# Visible-Light-Driven Benzylation of *In Situ*-Formed Imines Using Toluenes and Acridine Photocatalysis

**DOI:** 10.1021/acscatal.5c07891

**Published:** 2026-02-03

**Authors:** Beatriz Quevedo-Flores, Mario Martinez-Lopez, Loris Laze, Manuel A. Ortuño, Irene Bosque, Jose C. Gonzalez-Gomez

**Affiliations:** † Instituto de Síntesis Orgánica (ISO) and Departamento de Química Orgánica, 16718 Universidad de Alicante, Apdo. 99, 03080 Alicante, Spain; ‡ Departamento de Química Física, Universidad de Alicante, 03080 Alicante, Spain

**Keywords:** acridine photocatalysis, benzylic functionalization, three-component reaction, Porta-like reaction, 1,2-diphenylamines

## Abstract

The selective formation of benzyl radicals through the homogeneous photooxidation of toluene derivatives, followed by deprotonation, is difficult to implement when the involved chemical species have low oxidation potentials. Here, we present a successful application of this approach for the modular construction of biologically important 1,2-diarylethylamines from toluene derivatives, aldehydes, and anilines. This three-component reaction is driven by acridine photocatalysis under visible light, with trifluoroacetic acid (TFA) (or *p*-TsOH) serving as an acid additive that plays a triple role. The method is reliable, easy to use, metal-free, compatible with a wide range of functional groups, and more efficient under flow conditions. Unlike previous methods, no cocatalysts are required for the turnover of the acridine photocatalyst.

## Graphical abstract

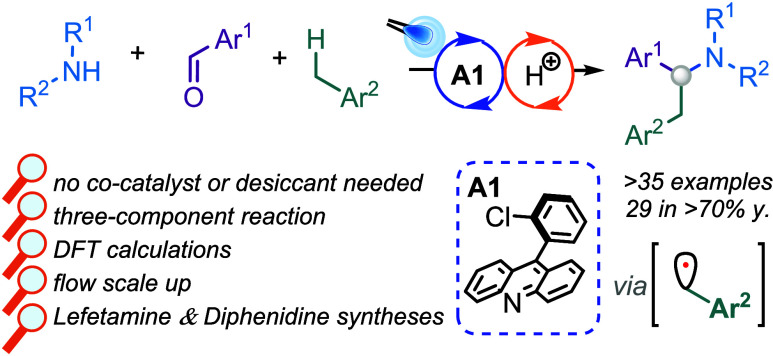

## Introduction

Several compounds featuring the 1,2-diarylethylamine scaffold display a wide range of bioactivities, including anticonvulsant, analgesic, neuroprotective, sympathomimetic, and bronchodilator effects.^1,2^ Notably, this pharmacophore is commonly found in drugs that act as antagonists of the *N*-methyl-*D*-aspartate (NMDA) receptor—a key player in processes such as learning and memory—as well as in the pathophysiology of epilepsy and neurodegenerative diseases.^3^ Representative members of this family of psychoactive substances, used both medically and recreationally, include DPPy, diphenidine, ephenidine, isophenidine, lefetamine, and remacemide (Scheme 1a).^4–8^

**Scheme 1:**
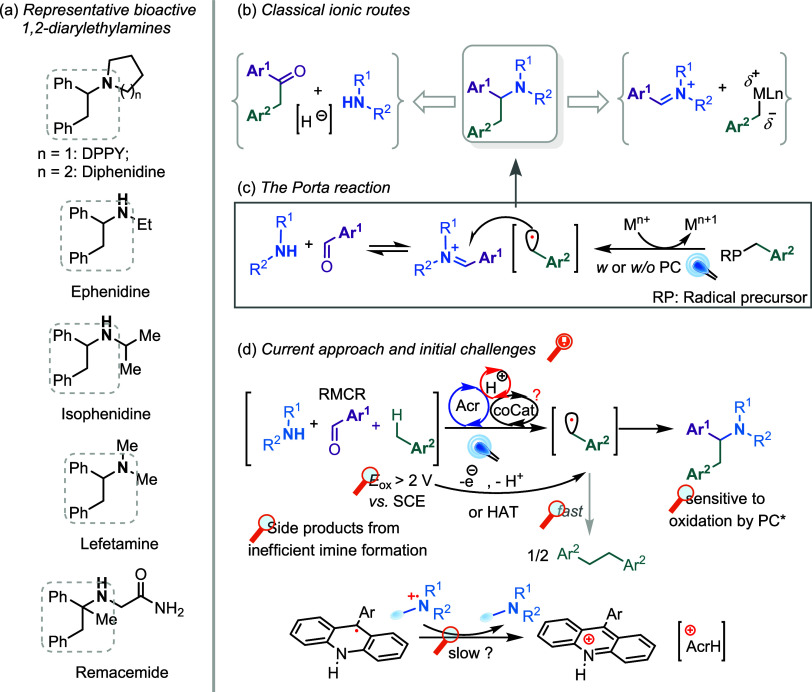
Background of Current Work: (a) Representative Bioactive 1,2-Diarylethylamines, (b) Classical Ionic Routes, (c) The Porta Reaction, and (d) Current Approach and Initial Challenges

Given the occurrence of diphenylethylamines in potent drugs and pharmaceuticals, considerable efforts have been devoted to enabling rapid access to diverse derivatives. Traditionally, this scaffold is accessed primarily via reductive amination or organometallic addition (Scheme 1b).^9^ Although recent protocols have introduced elegant and efficient ionic strategies,^10,11^ these often demand the prior synthesis of ketone or imine precursors and tend to struggle with base-sensitive functional groups. The radical addition to *in situ*-generated imines was pioneered by Clerici and Porta in 1990, leveraging the accelerated reactivity under acidic conditions—akin to the Minisci reaction.^12^ The same group subsequently developed various radical multicomponent reactions (RMCRs), expanding the repertoire of radical precursors and introducing innovative reaction conditions (Scheme 1c).^13–15^ The advent of visible-light photoredox catalysis^16–19^ has further advanced RMCRs, offering enhanced molecular complexity with high modularity, chemoselectivity, step economy, and energy efficiency.^20^ In this context, photoinduced multicomponent couplings of aldehydes, amines, and diverse radical precursors, including trifluoroborates, alkyl halides, alkyldihydropyridines, and organo­(tristrimethylsilyl)­silanes, have been recently reported. Considering the abundance and structural diversity of carboxylic acids from both industrial and natural sources, they are excellent feedstocks for synthetic applications. Building on this, the groups of Larionov^21^ and Dilman^22^ independently developed direct multicomponent decarboxylative couplings of carboxylic acids with aldehydes and amines. These protocols rely on light-driven proton-coupled electron transfer within hydrogen-bonded complexes formed between carboxylic acids and acridine photocatalysts. To facilitate acridine photocatalyst turnover—particularly involving cleavage of strong N–H bonds, Larionov et al. employed Co­(I) salts as single-electron transfer (SET) cocatalysts, while Dilman’s group utilized tetrabutylammonium decatungstate as a hydrogen atom transfer (HAT) cocatalyst.

Toluene derivatives produced on an industrial scale serve as convenient feedstocks for synthesizing high-value organic molecules.^23^ Thus, a multicomponent coupling of toluene derivatives with aldehydes and amines would provide modular access to the 1,2-diarylethylamine pharmacophore with H_2_O as the only byproduct and great atom economy. Despite some remarkable precedents in the benzyl radical addition to preformed imines,^24–26^ very few examples of RMCRs of toluene derivatives with aldehydes and amines have been reported.^27,28^ Additionally, a recent study described the photoinduced (467 nm) intramolecular addition of benzyl radicals to preformed imines, yielding morpholine derivatives, using a highly oxidizing pyrylium photocatalyst (TPP·BF_4_, *E*_red_* + 2.39 V vs SCE in MeCN).^29^

Prompted by these precedents, we set out to investigate the multicomponent coupling of toluene derivatives with aldehydes and amines under acridine photocatalysis. At the outset of this project, we identified the following key challenges (Scheme 1d): (a) the high oxidation potentials of toluene derivatives^30^ might hinder their activation by photoexcited acridinium species;^29^ (b) a cocatalyst might be required to promote the HAT from weak benzylic C–H bonds or to support the slow acridine turnover;^21,22^ (c) efficient *in situ* imine formation is essential to prevent aminal formation, homolytic cleavage of the C­(O)–H bond, and other side reactions; (d) benzyl radicals are prone to dimerization, as observed in classical Kolbe decarboxylation;^31^ and (e) the final amines are susceptible to oxidation by photoexcited acridines unless sufficiently protected by protonation.

## Results and Discussion

### Reaction Development

We selected the three-component coupling of benzaldehyde, aniline, and toluene as a model reaction, employing neutral acridine **A1** as the photocatalyst in the presence of stoichiometric amounts of trifluoroacetic acid (TFA) and pyridine *N*-oxide as a HAT cocatalyst, conditions derived from previous studies by our research group.^32,33^

Initial optimization studies were conducted at room temperature under 450 nm LED irradiation, with varying reagent stoichiometry, **A1** loading, solvent, and reaction time. We were pleased to observe full conversion to the desired product, *even in the absence of pyridine N-oxide* (Scheme 2a).^34^ The reaction works smoothly in pure hexafluoroisopropyl alcohol (HFIP), but better results were obtained in a mixture with dichloroethane (DCE), with optimal results for a 7:3 DCE/HFIP ratio (Table S1). It is worth noting that HFIP is a polar, non-nucleophilic, and strongly hydrogen-bond-donating solvent that can facilitate proton transfer, solvate anions, and stabilize cations.^35^ Control reaction experiments showed that TFA, HFIP, **A1**, and light were essential for productive reactivity. Interestingly, the presence of air had no significant impact on the reaction outcome. In contrast, under 405 nm irradiation, the reaction performed poorly without a photocatalyst. Classical *N*-alkyl­(aryl) acridinium photocatalysts (**A2**: Fukuzumi catalyst; and **A3**) were markedly less efficient, underscoring the superior performance of readily prepared acridine **A1**. Remarkably, the reaction worked well at room temperature without the need for desiccants to remove water, an inert atmosphere, or a large amount of catalyst (**A1**). Unlike previous methods, it did not need any cocatalysts, making the process very simple, easy to use, and scientifically intriguing.

**Scheme 2:**
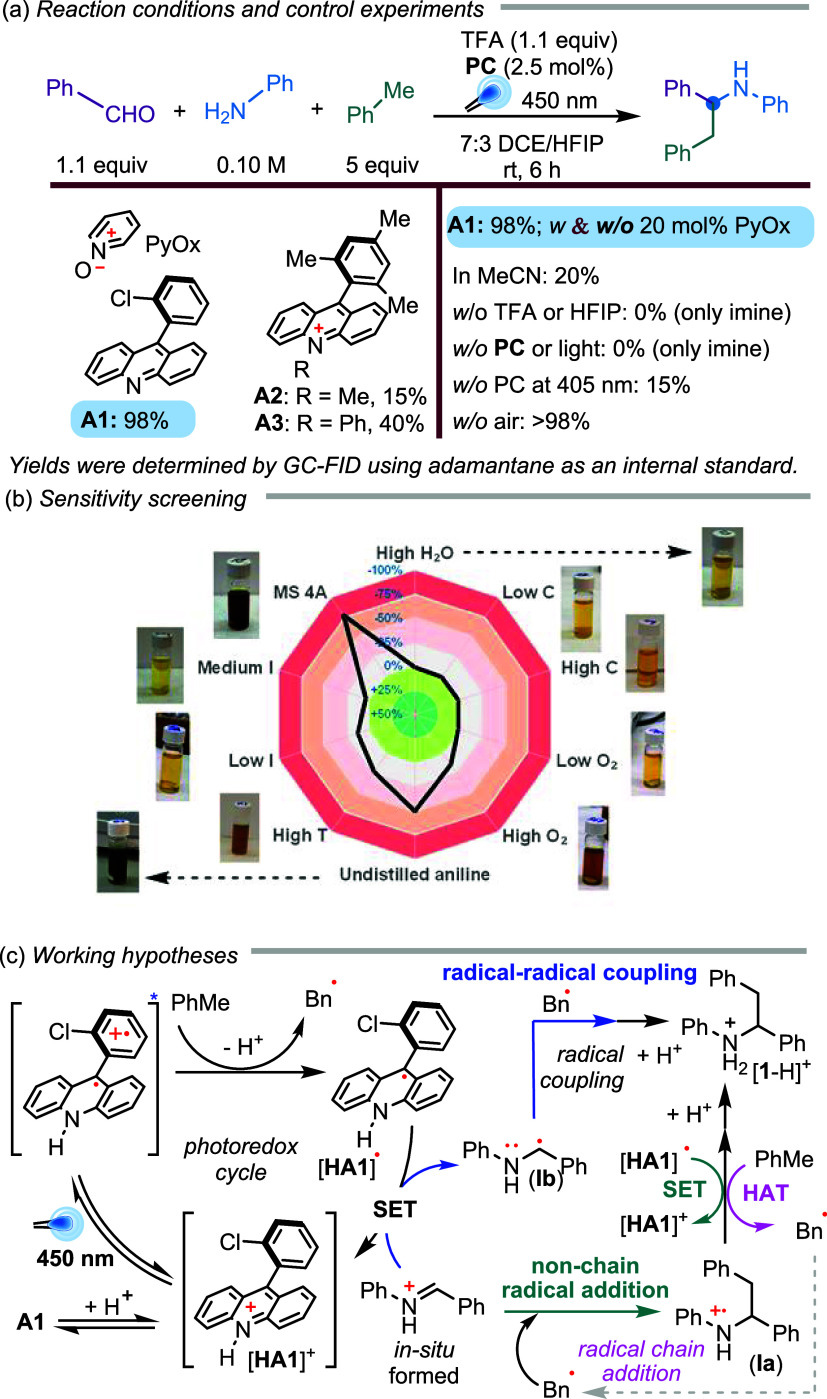
Optimizing the Three-Component Coupling and Working Hypotheses: (a) Reaction Conditions and Control Experiments, (b) Sensitivity Screening, and (c) Working Hypotheses

We next examined the reaction sensitivity to external parameters that might affect this photoinduced reaction using the methodology proposed by Glorius and co-workers (Scheme 2b).^36^ As observed during the optimization, the reaction was poorly sensitive to moderate levels of dissolved O_2_, observing a decrease of only 14% yield after bubbling air in the reaction mixture for 1 min before closing the vial (named high O_2_ in the diagram). The irradiance at different positions in the photoreactor (Figure S2) after 6 h or a moderate change in the reagent concentration had little impact on the reaction outcome. Notably, the reaction was not affected by the addition of H_2_O; however, molecular sieves significantly impacted the reaction outcome (likely due to reduced light penetration in the reaction mixture). This result suggests that the equilibrium in imine formation is shifted by its conversion to the final product, an intrinsic advantage of this three-component reaction. Remarkably, we found that the reaction is highly sensitive to aniline purity, requiring distilled aniline to obtain reproducible results. Light-induced degradation of aniline was observed under the reaction conditions, particularly in the presence of air, likely catalyzed by trace metals in undistilled aniline (see SI). Finally, a moderate increase in the temperature (from 30 to 45 °C) negatively impacted the reaction yield. A detailed explanation of these experiments is provided in the Supporting Information (Table S2). Interestingly, a light-yellow reaction mixture at the end indicated a good result, while dark reaction mixtures were obtained when poor reactivity was achieved (see the pictures in Scheme 2b).

After obtaining these results, we considered different possible mechanistic scenarios. Our previous studies have shown that when acridine **A1** reacts with TFA, the corresponding acridinium compound becomes photoactive upon irradiation at 450 nm. We hypothesized that this photoexcited acridinium could oxidize toluene via single-electron transfer (SET), generating a benzyl radical (Bn^•^) after deprotonation. This nucleophilic radical would rapidly add to the *in situ*-generated iminium, forming a radical-cation intermediate (**Ia**) that could regenerate the photocatalyst, ultimately yielding the protonated product under the acidic conditions used (a nonchain radical addition pathway similar to the one proposed by Porta and co-workers in their seminal studies^12^). Alternatively, the photocatalyst turnover might occur via reduction by the iminium, forming a stabilized α-amino benzylic radical (**Ib**),^37–39^ which could then couple with Bn^•^, a process likely influenced by the persistent radical effect (radical–radical coupling).^40^ This pathway is less likely with aliphatic aldehydes since the benzylic stabilization disappears from the corresponding intermediate **Ib**. A third possibility involves a chain mechanism, where radical cation **Ia** abstracts a hydrogen atom from toluene (radical chain addition). In all scenarios, TFA plays a triple role: it promotes the photoactivation of **A1**; protonates the intermediate imine, thereby improving its reactivity; and protonates the final product, preventing its photooxidation. Moreover, protonation of the *in situ*-formed imine accelerates the addition of Bn^•^ or facilitates iminium reduction.

### Substrate Scope

Having identified the optimal conditions, we next explored the scope of toluenes in reactions with benzaldehyde and aniline (Scheme 3). Toluene derivatives bearing additional methyl groups or chloro-substituents delivered the desired products (**1**-**4**) in excellent yields. Alkyl benzenes such as ethyl benzene, indane, and functionalized 4-phenyl-1-butene afforded products **5**-**7** as diastereomeric mixtures, also in very good yields. Notably, isochromane reacted regioselectively at the benzylic position adjacent to the oxygen atom, furnishing product **8** in good yield as a diastereomeric mixture. Remarkably, the reaction of (3-chloropropyl) benzene proceeded smoothly, followed by intramolecular cyclization to obtain diastereomeric pyrrolidines **9** in excellent yield. A variety of aromatic aldehydes was also examined under these conditions. Halogen- and alkyl-substituted benzaldehydes were excellent substrates, affording products **10**-**13**. Electron-withdrawing groups, such as trifluoromethyl and cyano, were also well tolerated in the aromatic aldehyde substrates, although the corresponding products (**14**, **15**) were obtained in moderate-to-good yields. A 4-chloropyridine moiety was tolerated, albeit in lower yields (**16**). Importantly, aliphatic aldehydes, including tertiary, secondary, and primary types, were suitable coupling partners, yielding products **17**-**19**. It is worth noting that imines derived from secondary and primary aliphatic aldehydes exist in equilibrium with their corresponding enamine; nevertheless, the reaction proceeds effectively with these substrates. Finally, we found that these reaction conditions were compatible with a broad range of anilines. As shown by products **20**-**30**, diverse functionalities were well tolerated, including the secondary aniline, which gave product **26** in excellent yield, and a 3-aminothiophene derivative (**29**) in moderate yield. A notable limitation of this protocol was found with *p*-methoxyaniline, which failed to produce amine **30**. This result was particularly concerning given the well-documented ease of removal of the *p*-methoxyphenyl (PMP) group,^41^ a key step in accessing bioactive diphenylethylamines. Additional substrates that were incompatible with these reaction conditions are listed in Figure S32.

**Scheme 3:**
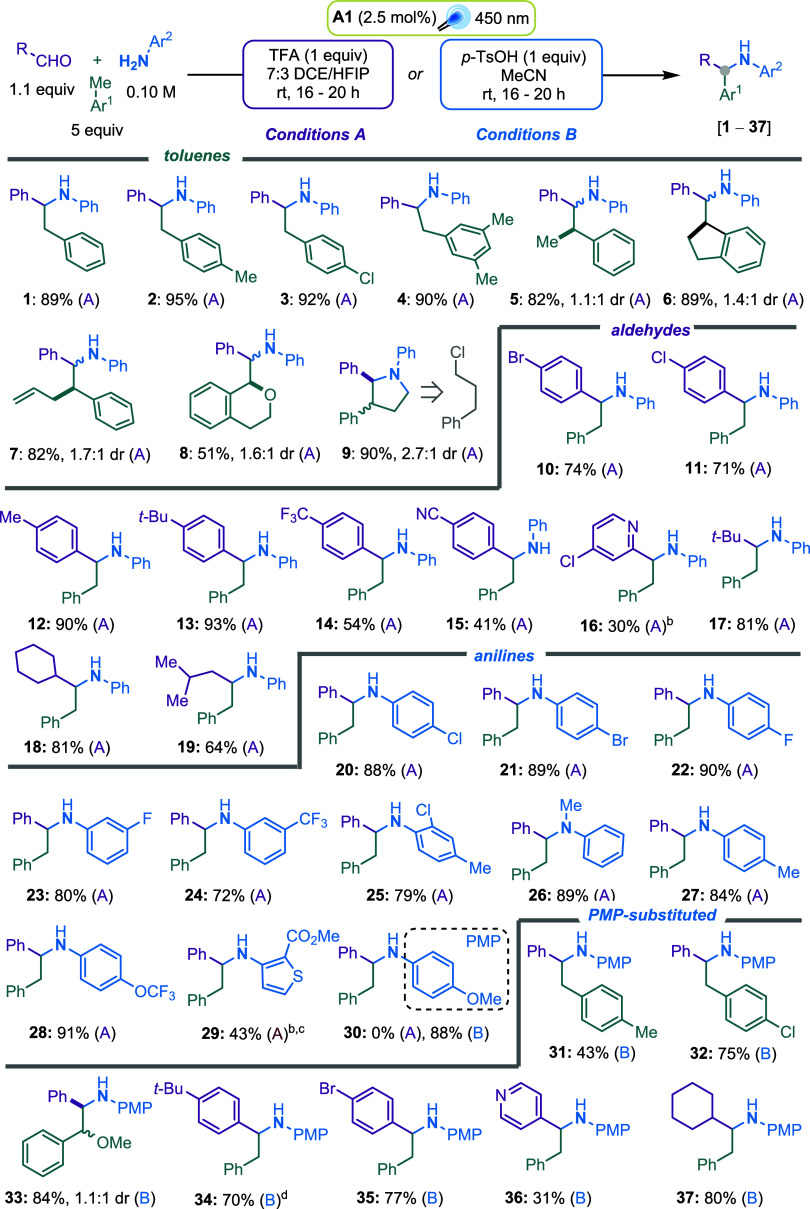
Substrate Scope^d^

a
*Reaction conditions*: **GPA**: aniline (0.30 mmol), aldehyde (0.33 mmol), toluene derivative (1.50 mmol), **A1** (0.0075 mmol), TFA (0.33 mmol), DCE (2.1 mL), HFIP (0.9 mL), LED light (450 nm), 16–20 h at RT. *Reaction conditions*: **GPB**: *p*-TsOH (0.33 mmol) was used as acidic additive and MeCN (3 mL) as solvent. Yields are reported for isolated pure compounds.
b Two equiv TFA.
c 48 h
d Five mol % of **A1** was used over 48 h.

### Reaction Development for PMP-Aniline

Our interest in using *p*-methoxyaniline for further removal of the PMP group prompted us to examine new reaction conditions. A detailed analysis of the UV–vis spectra of the iminium ion formed from benzaldehyde and aniline in the reaction medium (7:3 DCE/HFIP and TFA) revealed minimal absorbance at 450 nm. This enables efficient photoactivation of the acridinium photocatalyst under blue light. In contrast, the iminium ion derived from *p*-methoxyaniline absorbs much more than the acridinium photocatalyst at 450 nm, under similar conditions, preventing the photoactivation of the acridinium (Figure S9). We hypothesized that this “shadow effect” could be mitigated using a reaction medium in which the *p*-methoxyaniline-derived iminium ion is poorly soluble. To test our hypothesis, we explored alternative reaction conditions to prepare PMP-protected amine **30** (Table S3). Eventually, we found that the desired product was obtained in excellent yield using MeCN as the solvent and *p*-TsOH as the acid mediator (entry 4). Interestingly, a precipitate appeared within minutes of mixing the reagents and disappeared by the end of the reaction (Figure S8). This result is consistent with a very low concentration of this iminium in the reaction medium, which is too low to inhibit photocatalyst activation but sufficient to drive the reaction forward (Figure S10). We also observed that stoichiometric amounts of *p*-TsOH and the presence of photocatalyst **A1** are essential for the reaction to run to completion (Table S3, entries 5 and 6).

We therefore investigated the reactivity of PMPNH_2_ with other toluene derivatives and aldehydes under the newly developed reaction conditions (General Procedure B: GPB). Toluene derivatives bearing additional methyl groups or chloro-substituents were well tolerated, affording products **31** and **32** in good to moderate yields. Notably, methyl benzyl ether—previously a poor substrate under conditions A—performed well using this protocol, delivering diastereoisomers **33** in excellent yield. A range of aromatic aldehydes also served as effective coupling partners under these conditions, affording products **34**-**36**, including heteroaromatic aldehyde. Due to the poor solubility of iminium, a larger **A1** load and a longer reaction time were used for product **34**. Importantly, an aliphatic aldehyde provided product **37** in a very good yield. Additional substrates that were incompatible with these conditions are listed in Figure S33.

### Synthetic Applications

To explore the applications of the developed synthetic protocols, we first demonstrated that each protocol could be easily scaled up 10-fold (to 3 mmol) under batch conditions, although with a moderate decrease in the reaction yield (Scheme 4a). We speculated that this decrease in reaction yield could be attributed to the inefficient control of reaction temperature at this scale. To address this issue, flow chemistry offers a practical approach for scaling up the process, enabling precise temperature control, efficient heat dissipation, and improved light penetration across larger reaction volumes.^42^ After some experimentation with a PFA microreactor (1.6 mm ID, *V*_R_ = 2 mL), we obtained the best results with a retention time of 20 min (Table S5). Under these conditions, the reaction with 3 mmol of aniline was complete after 300 min, yielding 96% of product **1**, thereby increasing both yield and productivity to 13.8 mmol/day (vs 2 mmol/day in batch conditions).

**Scheme 4:**
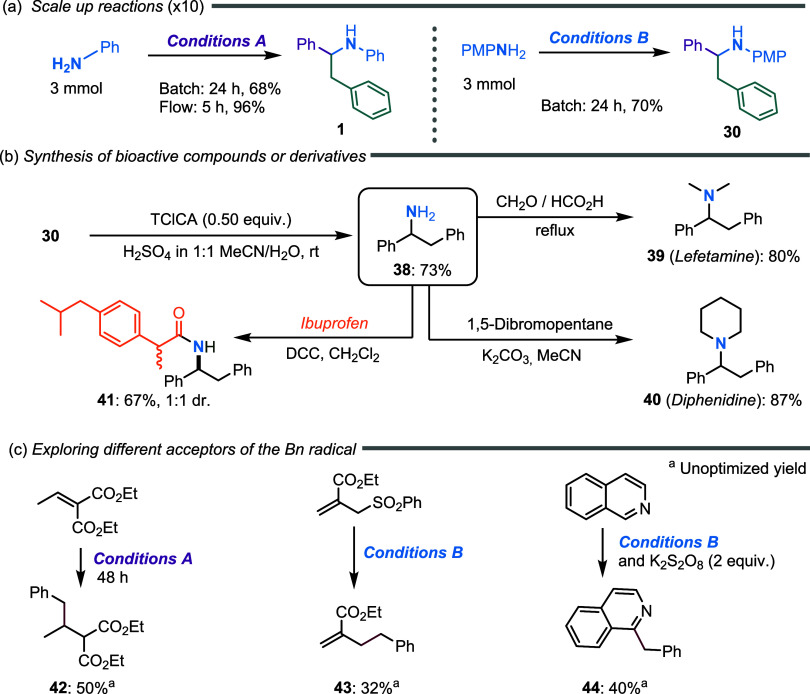
Synthetic Applications of the Developed Protocols: (a) Scale-Up Reactions (×10), (b) Synthesis of Bioactive Compounds or Derivatives, and (c) Exploring Different Acceptors of the Bn Radical

The synthetic potential of GPB was validated by synthesizing different valuable bioactive compounds. The PMP group was removed from compound **30** using trichloroisocyanuric acid as the oxidant under mild conditions to obtain the parent 1,2-diphenylamine **38** in good yield (Scheme 4b). The reductive alkylation of this amine allowed the straightforward preparation of bioactive lefetamine (**39**) and diphenidine (**40**), both in excellent yields. On the other hand, the same synthetic intermediate was coupled with Ibuprofen under mild and simple conditions, providing **41**. We also demonstrated that the tolyl radicals generated by visible-light-driven acridine photocatalysis from toluene could be trapped by radical acceptors other than iminiums, expanding the chemical space covered by this innovation. For example, diethyl ethylidenemalonate reacted smoothly as a Giese acceptor of the tolyl radical using GPA to obtain product **42** with moderate yield. On the other hand, addition/elimination to an electron-poor allylsulfone proceeded smoothly to afford product **43** using GPB.^43^ Moreover, when isoquinoline was used as the radical acceptor with GPB, but in the presence of K_2_S_2_O_8_ as the terminal oxidant, a Minisci-like reaction took place to obtain product **44**.^44^

### Mechanistic Investigations

We confirmed by cyclic voltammetry (CV) experiments that toluene starts its oxidation around +1.51 V (vs SCE) in 7:3 DCE/HFIP and about +1.71 V in MeCN (Figures S20 and S21), which means that photoexcited protonated **A1** is oxidant enough for this single-electron oxidation (*E*_p/2_ + 2.08 V in 7:3 DCE/HFIP, and +2.24 V in MeCN, Figures S12–S15). After deprotonation of the radical cation, the generated benzyl radical may follow different pathways, which were initially examined by DFT (Scheme 5a). Evaluation of the nonchain radical addition path (Porta-like mechanism) revealed an exergonic addition of the benzyl radical to the *in situ*-formed iminium through a low activation barrier of 10.4 kcal·mol^–1^ (**TS1**), followed by an almost barrierless highly thermodynamically favorable SET of 0.2 kcal·mol^–1^ (**SET1**) from acridinyl radical to obtain a product that is protonated in the acidic media, preventing its overoxidation (for CVs, see Figures S26 and S27). On the other hand, recycling the acridinium catalyst by SET from the acridinyl radical to the iminium ion is slightly endergonic but proceeds through a low estimated barrier of 11.8 kcal·mol^–1^ (**SET2**). The coupling of radical **Ib** (likely a persistent radical) with the benzyl radical is highly exergonic and must be extremely fast, affording product **1** via a reasonable radical–radical coupling pathway. Finally, hydrogen atom transfer (HAT) from toluene to radical cation **Ia** was thermodynamically disfavored by 10.9 kcal·mol^–1^. Comparing that step with almost barrierless **SET1**, we can rule out this radical chain addition. In summary, DFT calculations for product **1** indicate that nonchain radical addition and radical–radical coupling pathways can be operative, likely for aromatic aldehydes. Both routes involve rapid turnover of the acridinium photocatalyst without requiring cocatalysts, a question we had at the outset of the work, given the precedents in this field (Scheme 1). Importantly, similar DFT calculations for pivaldehyde (Figure S28) showed that the reduction of the iminium is highly endergonic and occurs significantly more slowly than the addition of the benzyl radical to the iminium intermediate. This conclusion can be reasonably extended to other aliphatic aldehydes, for which the nonchain radical addition is the preferred pathway. In addition, for prochiral benzylic substrates, the addition of the corresponding benzyl radical to the iminium is the stereodetermining step in the nonchain radical addition pathway. The approach from each face of a planar benzyl radical to the same face of a flat (*E*)-iminium intermediate looks very similar, which is in accordance with the poor diastereoselectivity obtained for products **5**, **6**, **7**, **8**, **9**, and **33**. DFT calculations conducted for product **6** revealed very low energy differences between the transition states leading to each diastereoisomer (Figure S29).

**Scheme 5:**
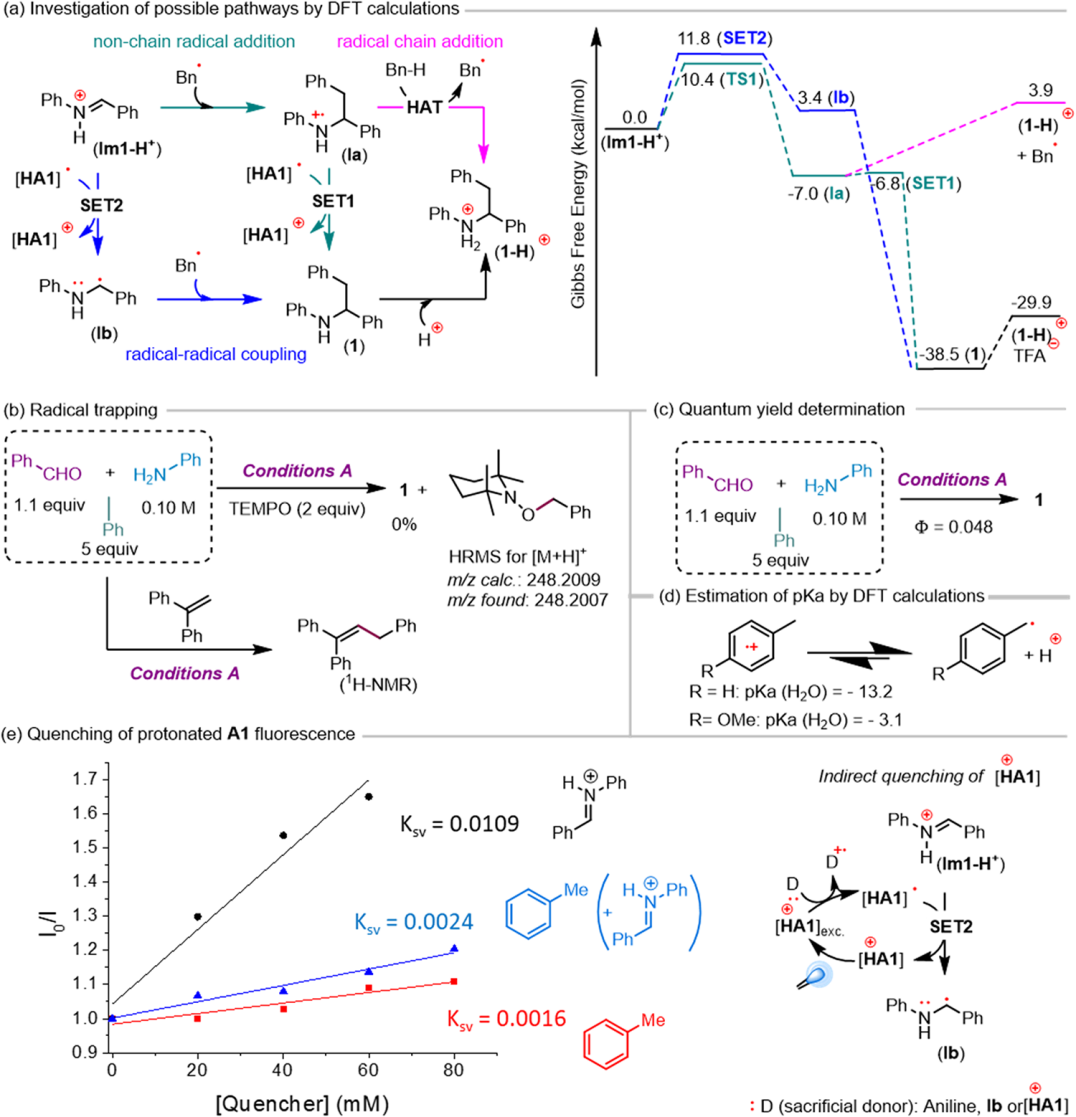
Mechanistic Studies: (a) Investigation of Possible Pathways by DFT Calculations, (b) Radical Trapping, (c) Quantum Yield Determination, (d) Estimation of p*K*_a_ by DFT Calculations, and (e) Quenching of Protonated A1 Fluorescence

We also conducted experiments to shed light on the reaction mechanism. The use of TEMPO or 1,1-diphenylethylene in GPA clearly supported the intermediacy of the benzyl radical (Scheme 5b). The quantum yield of the reaction to obtain product **1** was also measured, obtaining a value significantly lower than 1, suggesting a closed photoredox cycle and consistent with DFT calculations that rule out a radical chain mechanism. On the other hand, we were surprised that *p*-methoxytoluene, which is much easier to oxidize than toluene (Figures S22 and S23), failed to yield the desired product under either reaction condition (GPA or GPB). Thus, we estimated the p*K*_a_ of the corresponding radical cation by DFT, finding it to be 10 p*K*_a_ units higher than that from toluene (Scheme 5d; details in SI). Although H_2_O was assumed as the medium for these calculations, the trend is clear, and *p*-methoxytoluene should be much less acidic than toluene in other media. Therefore, considering that the reaction is performed under highly acidic conditions (TFA or *p*-TsOH), the deprotonation of the corresponding radical cation is likely the cause of the failure of this electron-rich toluene derivative, establishing some limitations for this methodology of generating benzyl radicals. Finally, we examined the fluorescence quenching of photoexcited protonated **A1** by different reaction mixture components. As observed in Scheme 5e, toluene was indeed a quencher, but this quenching was more pronounced in the presence of the imine. However, to our surprise, the best quencher was the *in situ*-formed iminium. Since the iminium is an oxidant, we hypothesize that a sacrificial donor (D: traces of aniline, intermediate **Ib** or [**HA1**]^+^) reduces the photoexcited catalyst to produce [HA1]^•^. Therefore, the oxidation of this species by the iminium to obtain intermediate **Ib** should be responsible for the pronounced quenching of the fluorescence obtained. According to DFT calculations, this reaction is expected to be slightly endergonic but fast (Scheme 5a, **SET2**). This evidence supports radical–radical coupling as a competent pathway for aromatic aldehydes.

## Conclusions

We have established a robust and modular three-component synthesis of biologically relevant 1,2-diarylethylamines directly from toluene derivatives, anilines, and aromatic aldehydes. This transformation is enabled by acridine photocatalysis under blue light irradiation (450 nm) in the presence of an acidic additive (TFA or *p*-TsOH), proceeding under metal-free conditions without the need for any cocatalyst. The protocol uses a low amount of a readily available acridine photocatalyst and shows good tolerance to a wide range of functional groups. Mechanistic investigations suggest that a radical chain is unlikely. Instead, plausible pathways include the nonchain radical addition (Porta-like mechanism) or radical–radical coupling, both of which are consistent with effective catalyst turnover when aromatic aldehydes are used. These studies also support the formation of benzyl radicals through the oxidation of the aromatic ring, followed by deprotonation, which enables the selective activation of benzylic C–H bonds. The acidic additive (TFA or *p*-TsOH) plays multiple roles: activating the photocatalyst, promoting *in situ* formation of a more reactive iminium intermediate, and preventing product oxidation. This methodology has proven effective for synthesizing a variety of bioactive 1,2-diphenylamines, and it is compatible with a range of benzyl radical acceptors, thereby expanding the scope of potential reactivity.

## Methods

### General Procedure A (GPA) for the Amino-Benzylation of Aldehydes with Toluenes and Anilines

In a two-dram vial equipped with a magnetic stirring bar, distilled aniline (0.30 mmol, 27 μL), distilled benzaldehyde (1.1 equiv, 0.33 mmol, 34 μL), and toluene (5 equiv, 1.50 mmol, 158 μL) were added, followed by 9-(2-chlorophenyl)­acridine (**A1**, 2.5 mol %, 2.2 mg, 0.0075 mmol). A mixture of 1,2-dichloroethane (DCE, 2.1 mL) and 1,1,1,3,3,3-hexafluoro-2-propanol (HFIP, 0.9 mL) was then added to the vial, followed by trifluoroacetic acid (TFA, 1.1 equiv, 0.33 mmol, 26 μL). The vial was sealed and placed in a PhotoRedOx Box Duo photoreactor. The reaction mixture was irradiated with blue LEDs (λ = 450 nm) for 6 h at room temperature (approximately 25–30 °C, controlled by a fan). After completion, the reaction mixture was concentrated under reduced pressure, and the resulting residue was dissolved in ethyl acetate (EtOAc). The mixture was then quenched by adding K_2_CO_3_ (approximately 40 mg), stirred for 30 min, and filtered. The crude mixture was concentrated under reduced pressure. The resulting residue was purified by flash column chromatography (silica gel, using Hexane as eluent) to afford the desired product.

### General Procedure B (GPB) for the Use of 4-Methoxyaniline (Including Synthesis of **30**)

In a two-dram vial equipped with a magnetic stirring bar, 4-methoxyaniline (0.30 mmol, 37 mg), distilled benzaldehyde (1.1 equiv, 0.33 mmol, 34 μL), and toluene (5 equiv, 1.50 mmol, 158 μL) were added, followed by 9-(2-chlorophenyl)­acridine (**A1**, 2.5 mol %, 2.2 mg, 0.0075 mmol). Acetonitrile (3 mL) was then added to the vial, followed by *p*-Toluenesulfonic acid (*p*-TsOH,1.1 equiv, 0.33 mmol, 56 mg). The vial was sealed and placed in the PhotoRedOx Box Duo photoreactor. The reaction mixture was irradiated with blue LEDs (λ = 450 nm) for 16 h at room temperature (approximately 25–30 °C, controlled by a fan). Once finished, EtOAc (10 mL) was added to the reaction mixture, which was then washed with sat. K_2_CO_3_ (3 × 5 mL). The combined organic phases were concentrated under reduced pressure, and the resulting residue was purified by flash column chromatography (silica gel, using Hexane as an eluent) to afford the desired product.

## Supplementary Material





## Notes

NMR data for all purified compounds (PDF)
Experimental procedures, materials, and methods, including photographs of experimental setup and computational details (PDF)
