# Supplementary material for: Visible-Light-Driven Benzylation of In Situ-Formed Imines Using Toluenes and Acridine Photocatalysis
Source: ACS Catal. 2026 Feb 3;16(4):3590–9. doi: 10.1021/acscatal.5c07891 (PMC12930518; doi:10.1021/acscatal.5c07891)

# *Supporting Information (NMR spectra)*

# *for*

## **Visible-light-driven benzylation of *in situ*-formed imines using toluenes and acridine photocatalysis**

Beatriz Quevedo-Flores[a], Mario Martinez-Lopez[a], Loris Laze[a], Manuel A. Ortuño[b], Irene Bosque[a],* and Jose C. Gonzalez-Gomez[a]*

[a]*Instituto de Síntesis Orgánica (ISO) and Departamento de Química Orgánica, Universidad de Alicante, Apdo. 99, 03080 Alicante, Spain*

[b]*Departamento de Química Física, Universidad de Alicante, 03080 Alicante, Spain*

*Email: josecarlos.gonzalez@ua.es

*Email: irene.bosque@ua.es

$^{1}$H NMR (400 MHz, $CDCl_3$) **1**

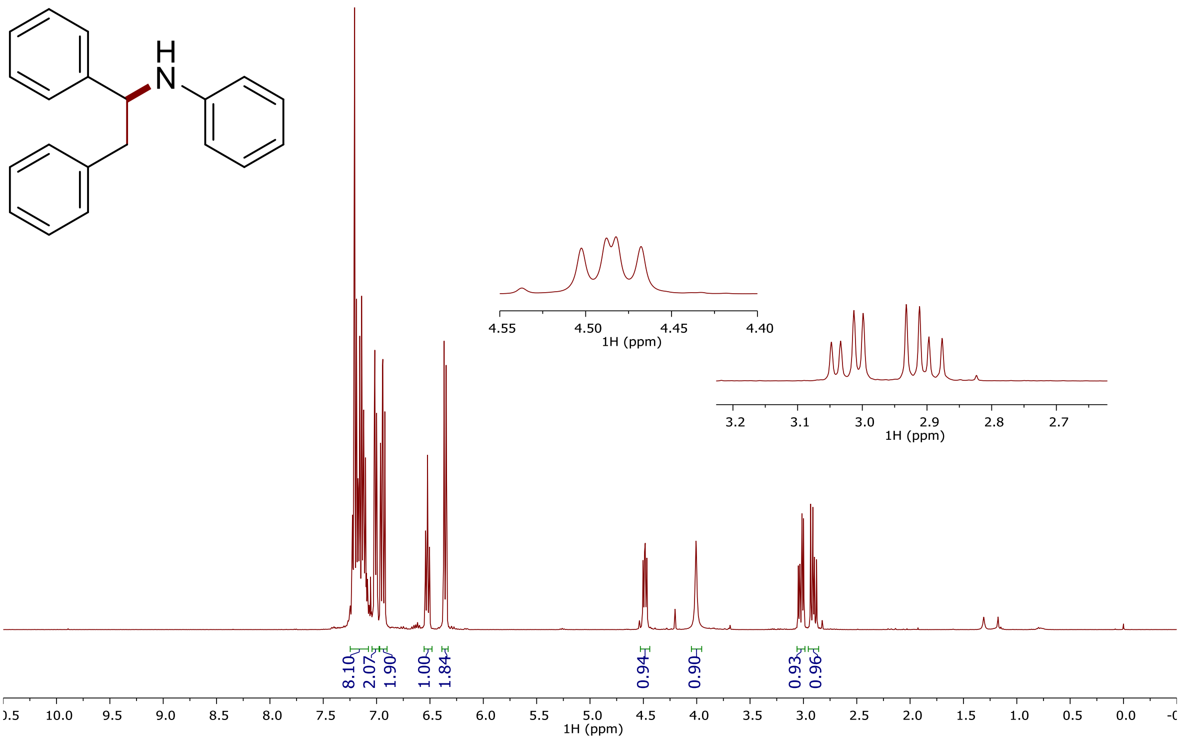


$^{13}$C NMR(101MHz,$CDCl_3$) **1**

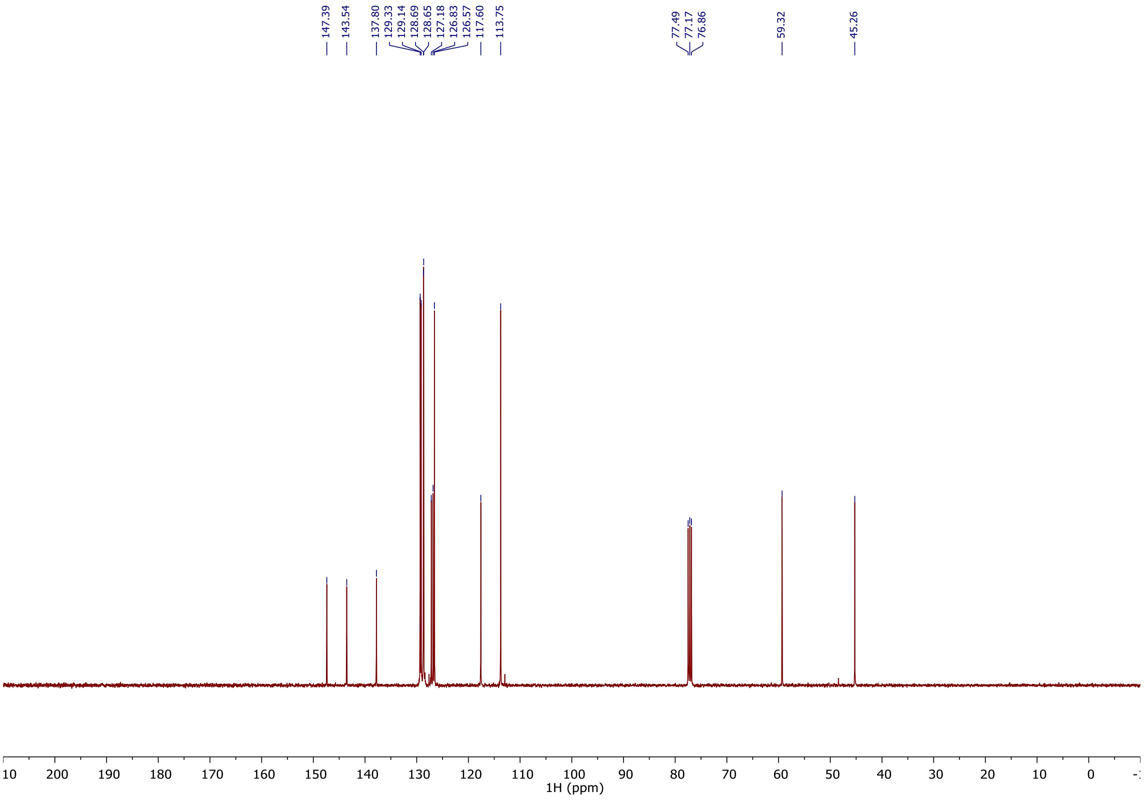

## $^{1}H$ NMR (400 MHz, $CDCl_3$) **2**

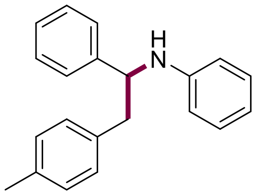

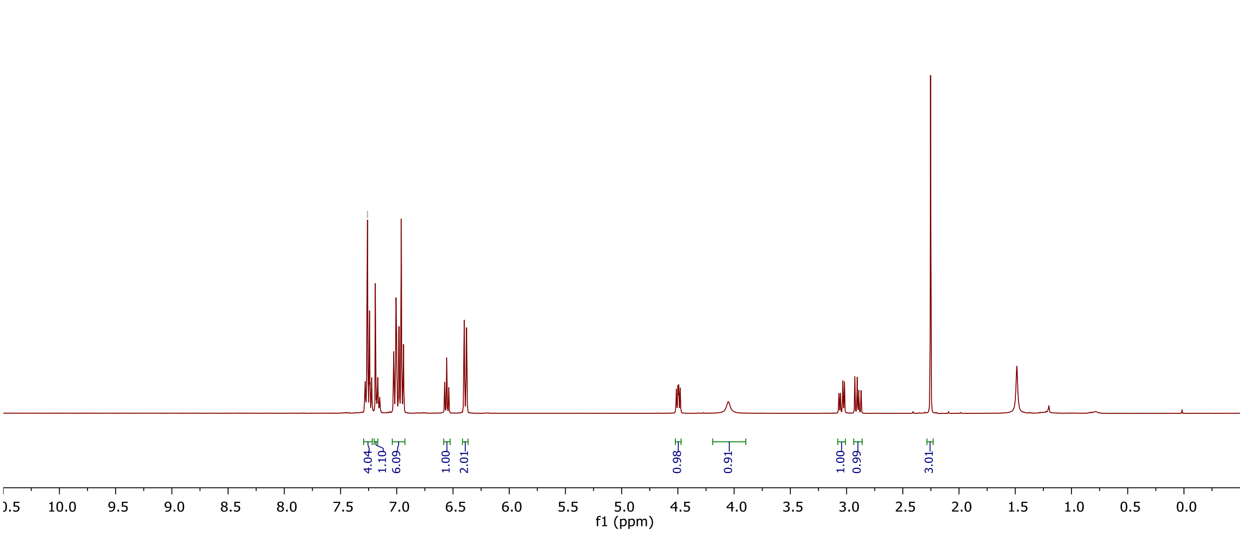


## $^{13}C$ NMR(101MHz,$CDCl_3$) **2**

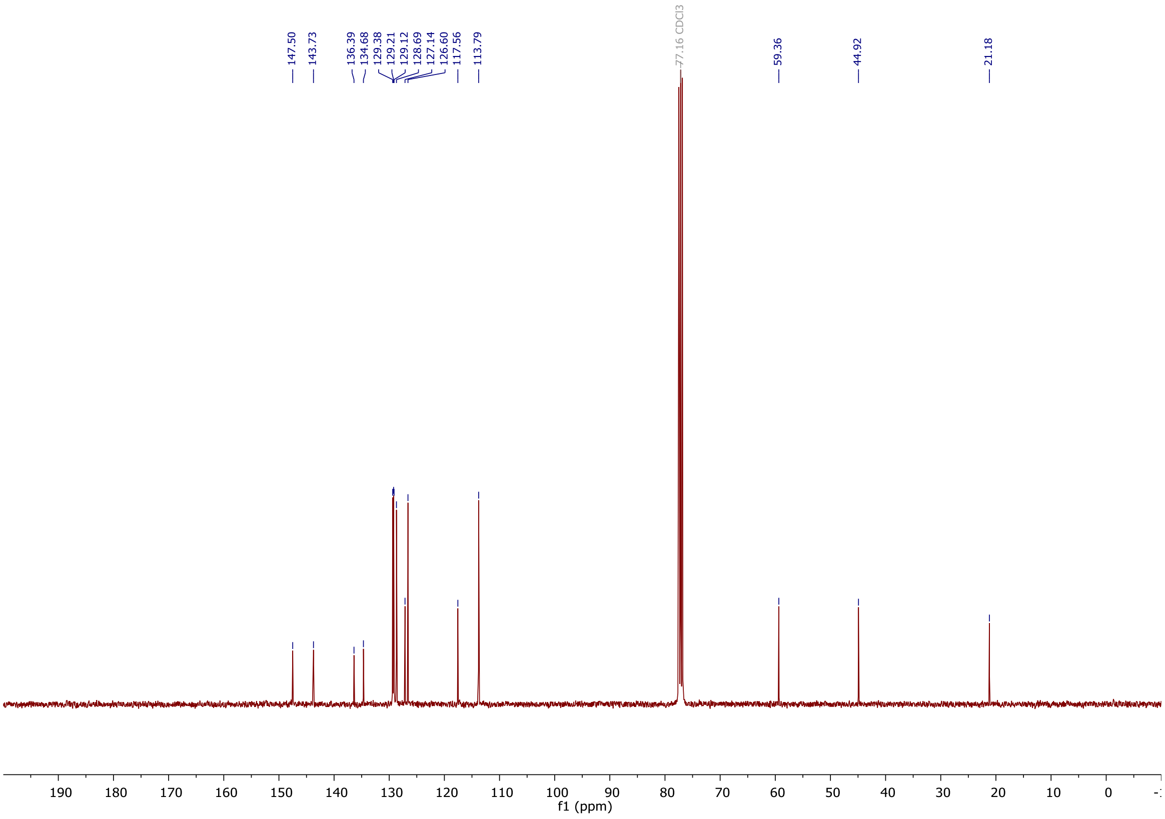

$^{1}$H NMR  (400 MHz, $CDCl_3$) **3**

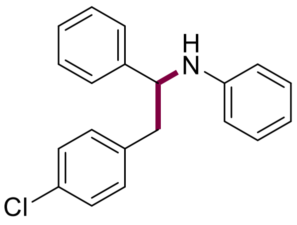


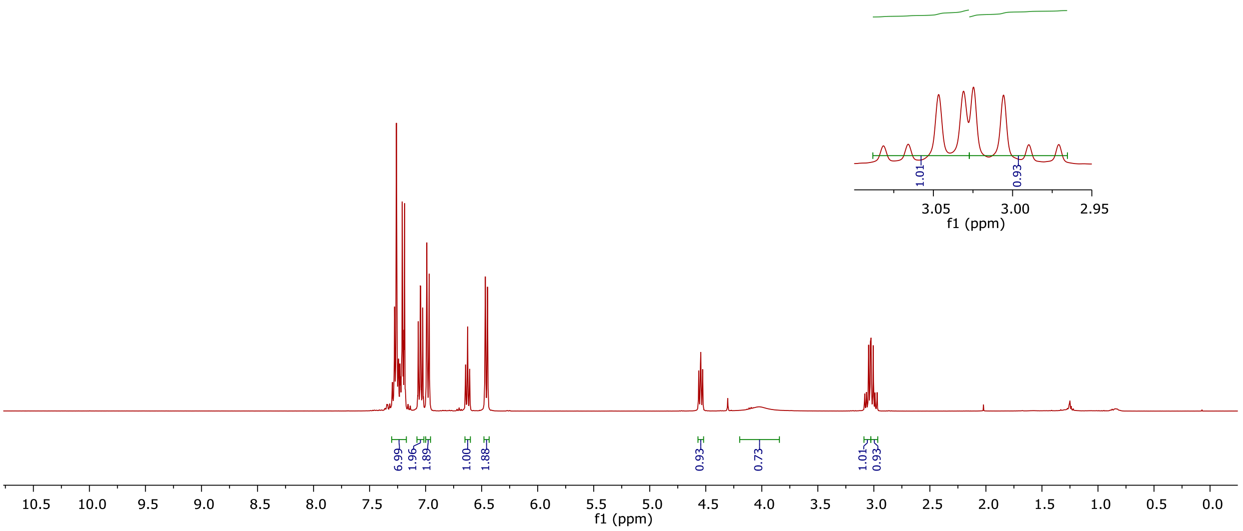


$^{13}$C NMR(101MHz,$CDCl_3$) **3**

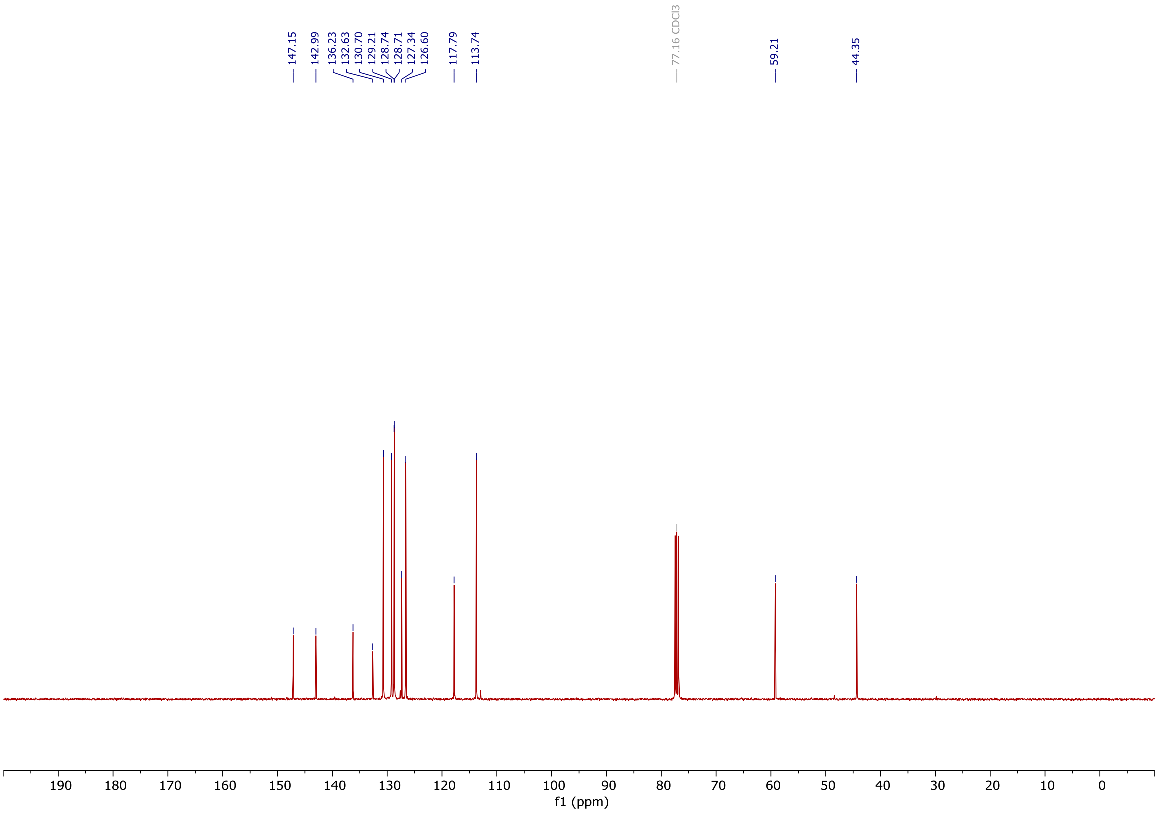

$^{1}H$ NMR (400 MHz, $CDCl_3$) **4**

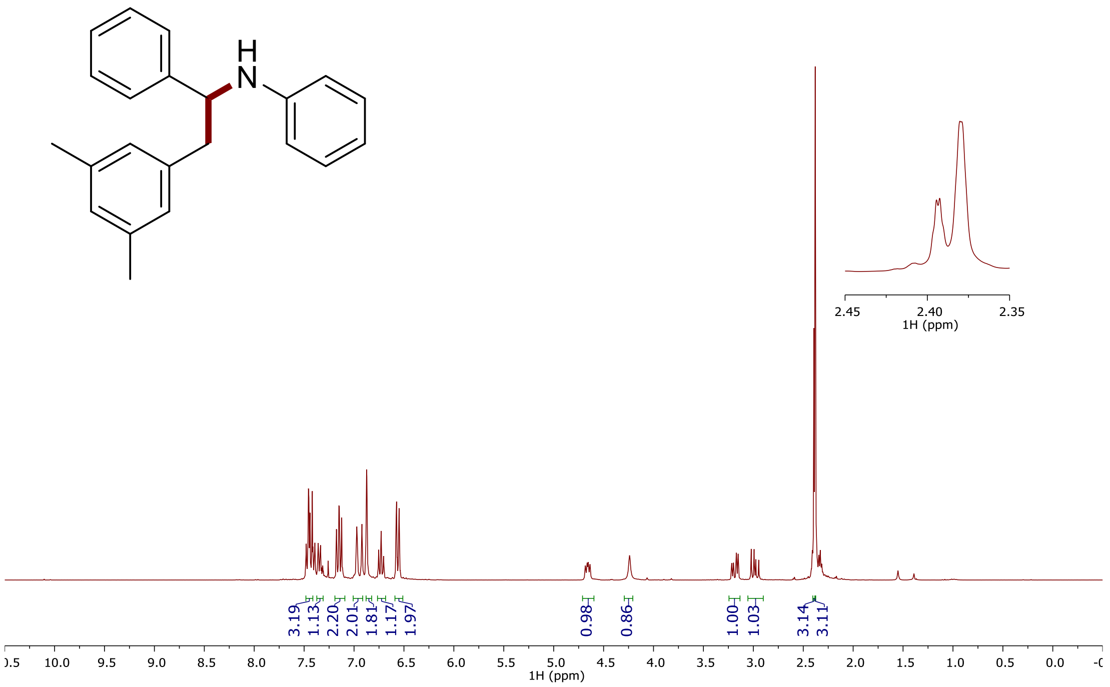


$^{13}C$ NMR(101MHz,$CDCl_3$) **4**

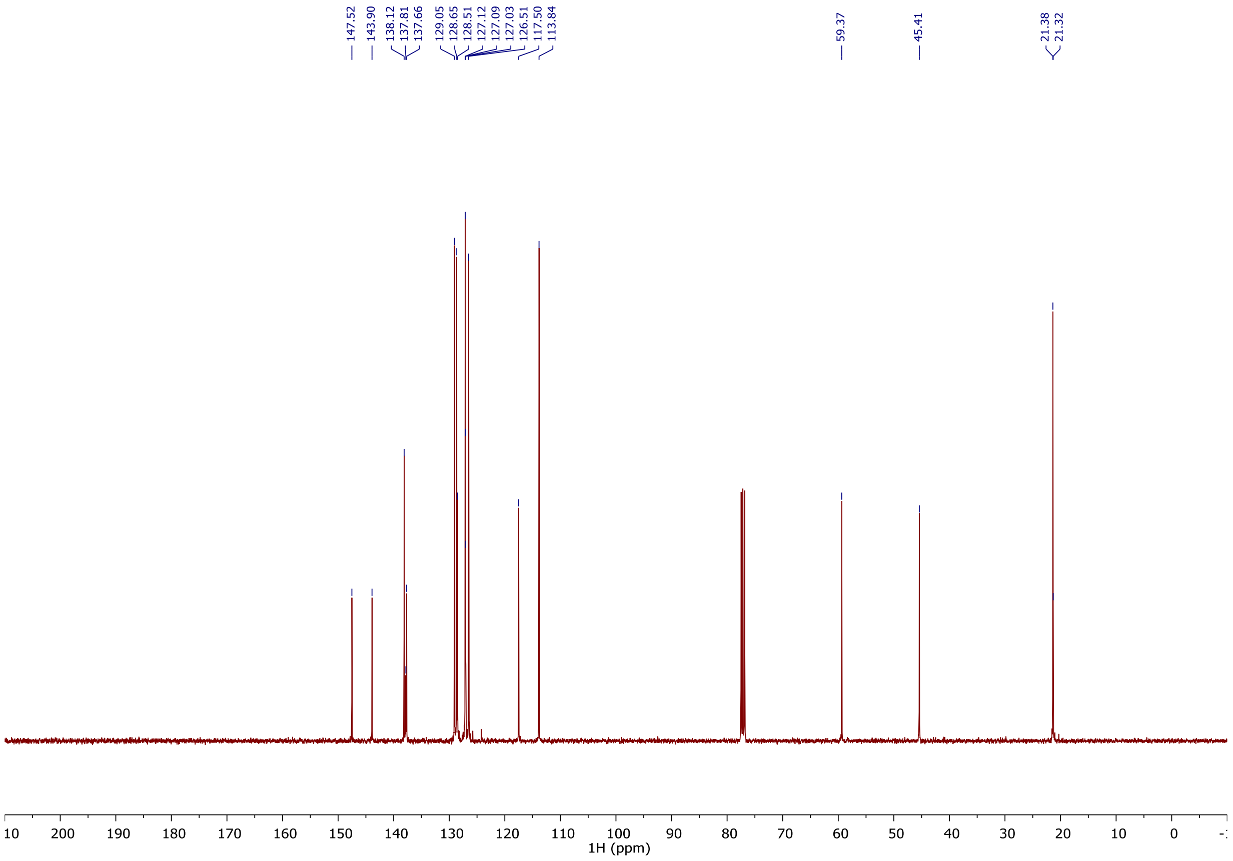

$^1$H NMR (400 MHz, CDCl$_3$) **5**

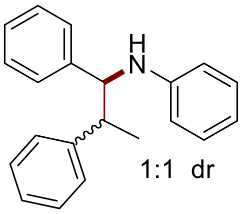


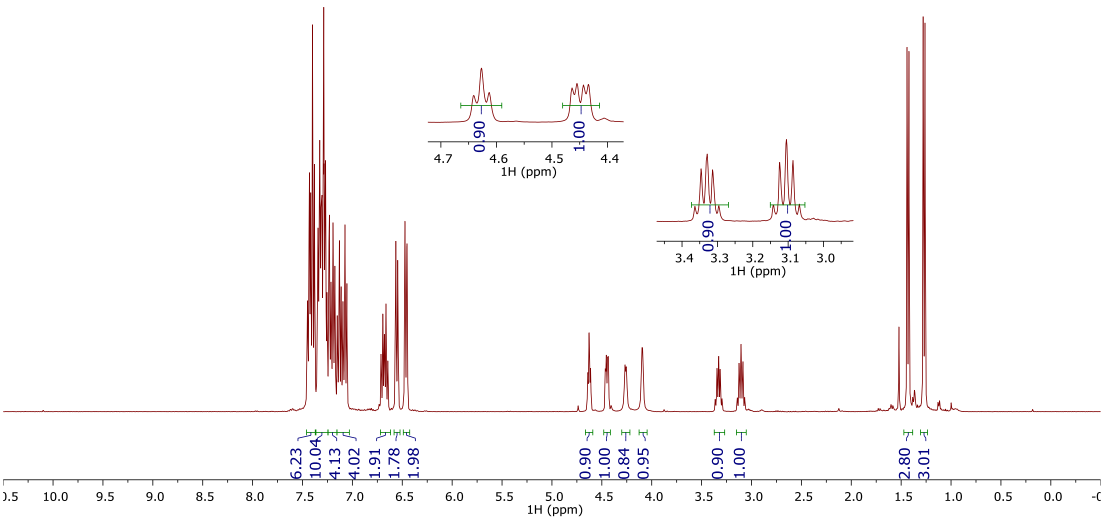


$^{13}$C NMR (101MHz,CDCl$_3$) **5**

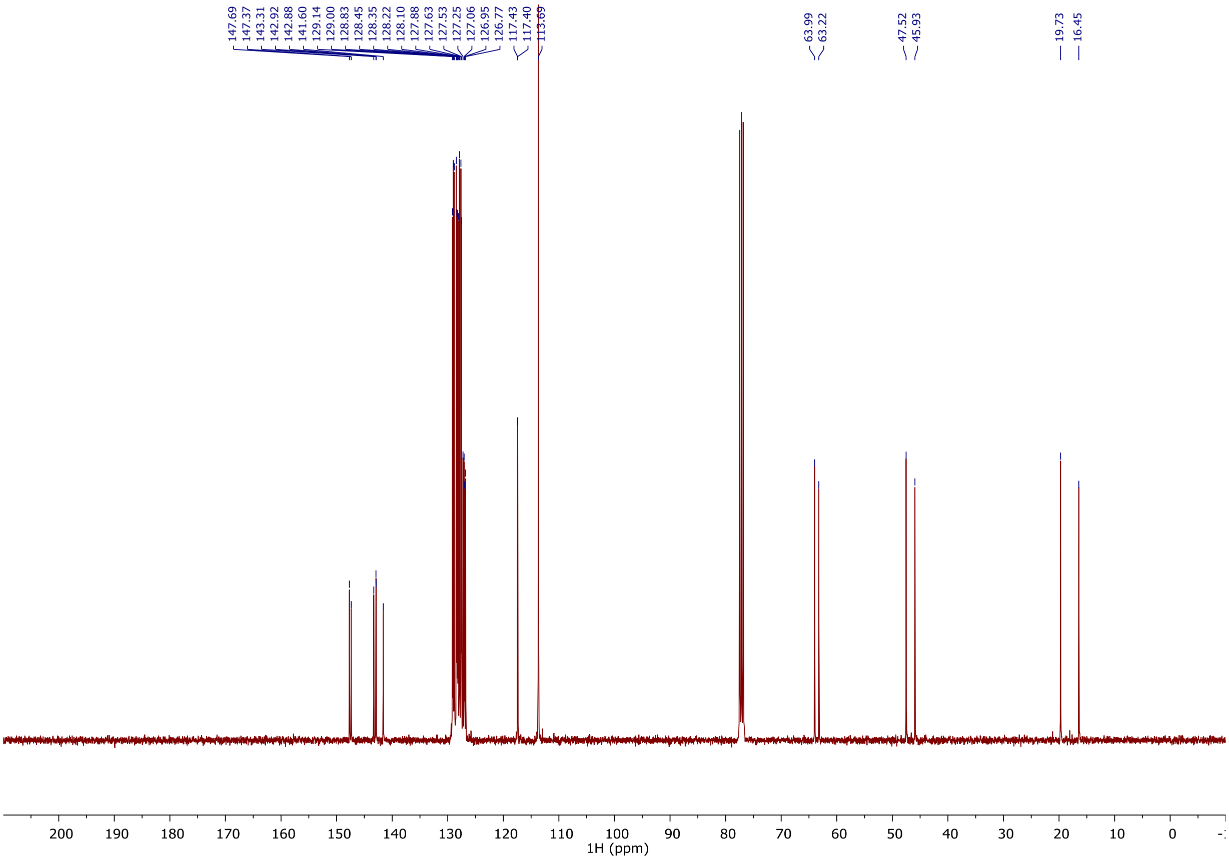

$^{1}$H NMR (400 MHz, $CDCl_3$) **6**

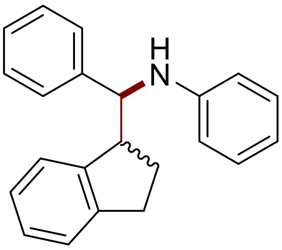


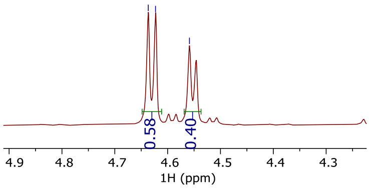


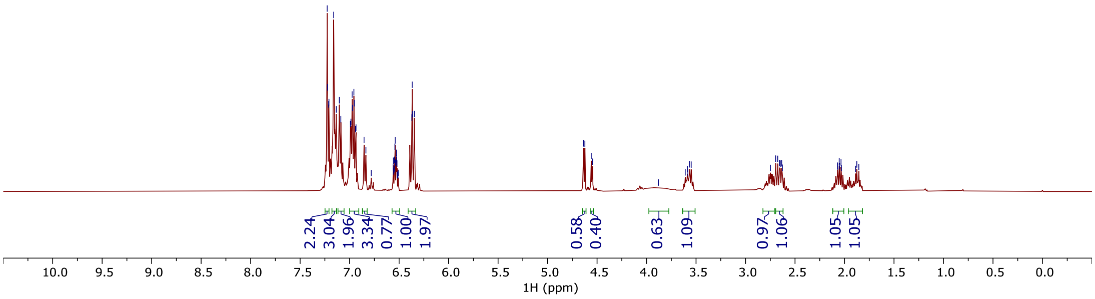


$^{13}$C NMR (101MHz,$CDCl_3$) **6**

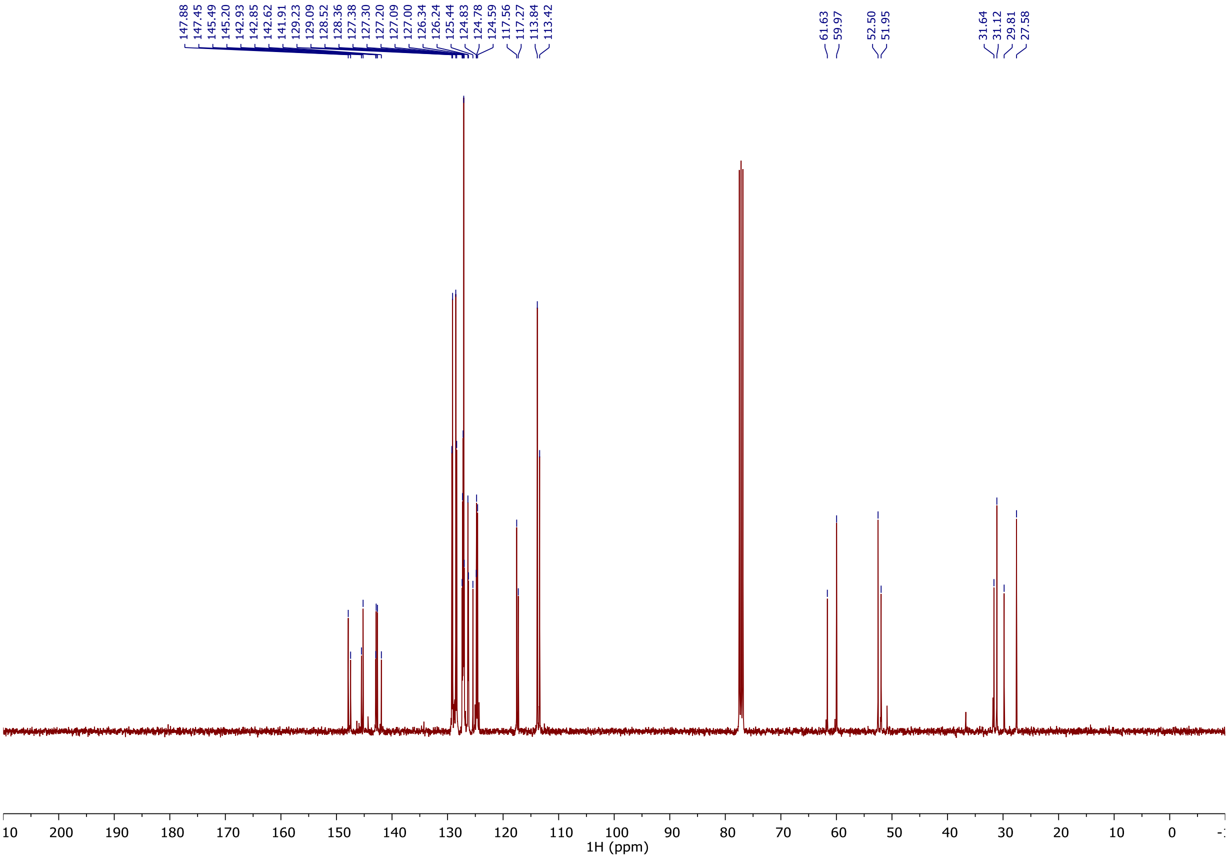

## $^{1}$H NMR (400 MHz, $CDCl_3$) **7**

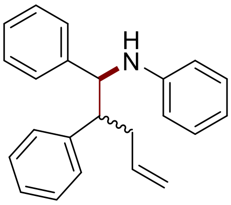


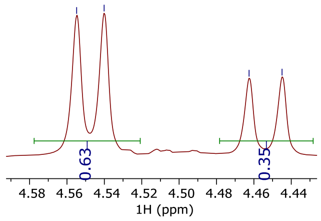


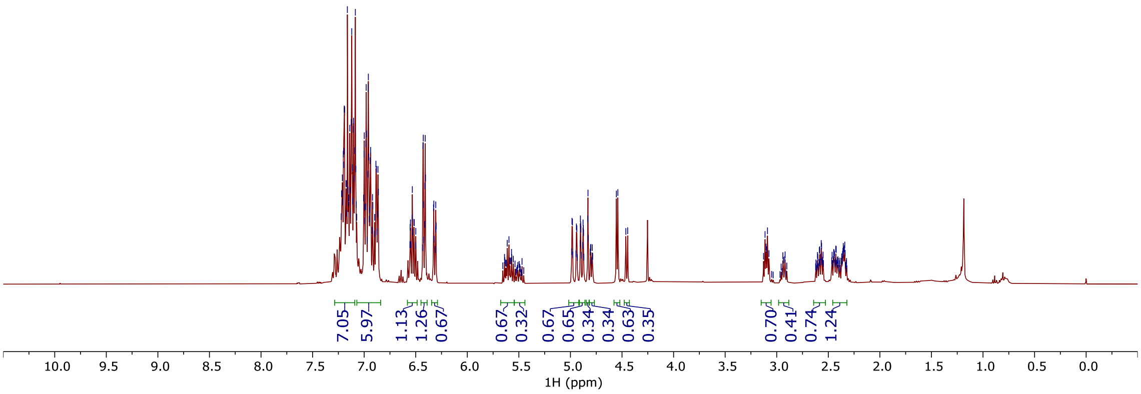


## $^{13}$C NMR(101MHz,$CDCl_3$) **7**

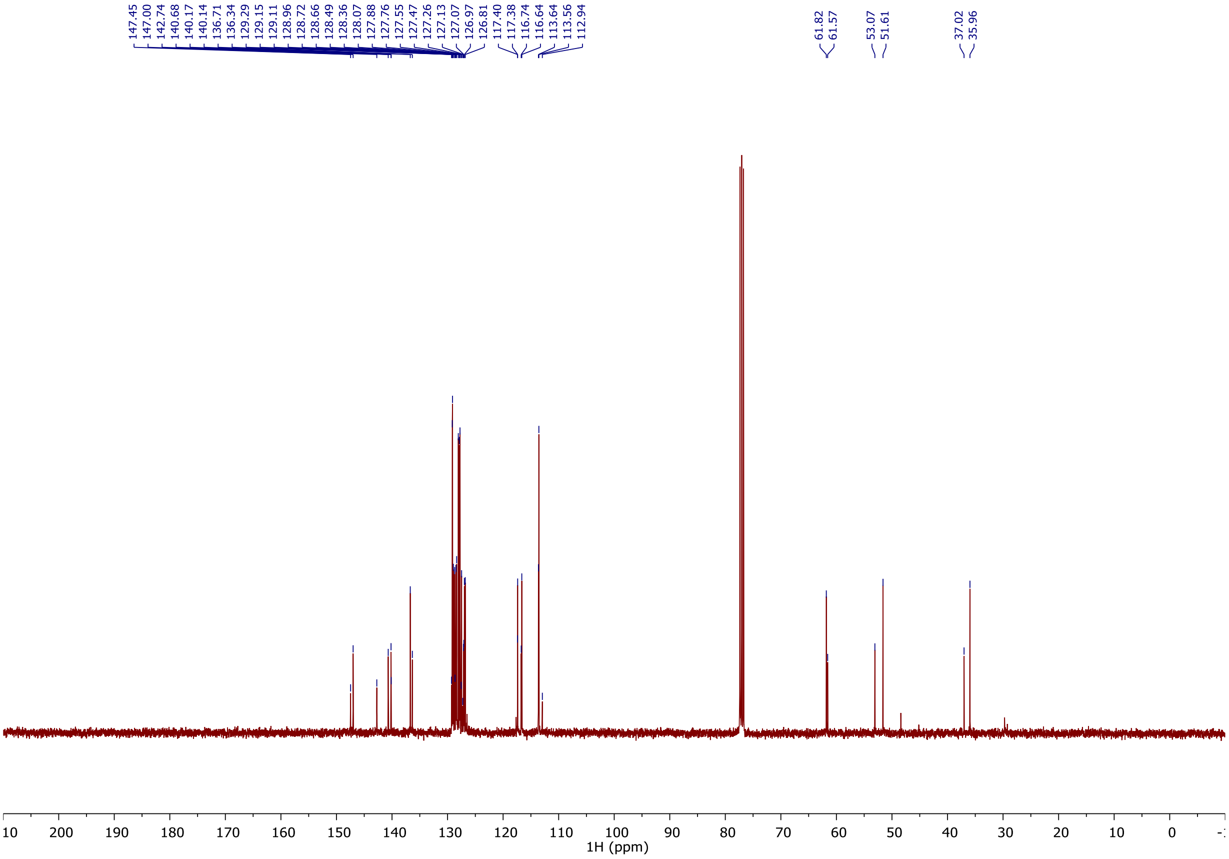

## $^{1}H$ NMR (400 MHz, $CDCl_3$) **8**

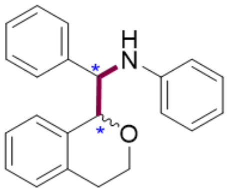

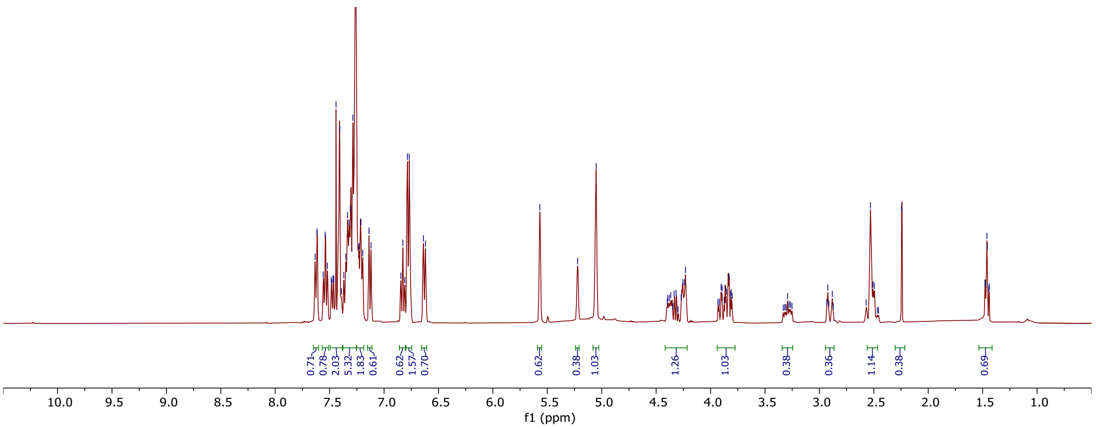


## $^{13}C$ NMR(101MHz,$CDCl_3$) **8**

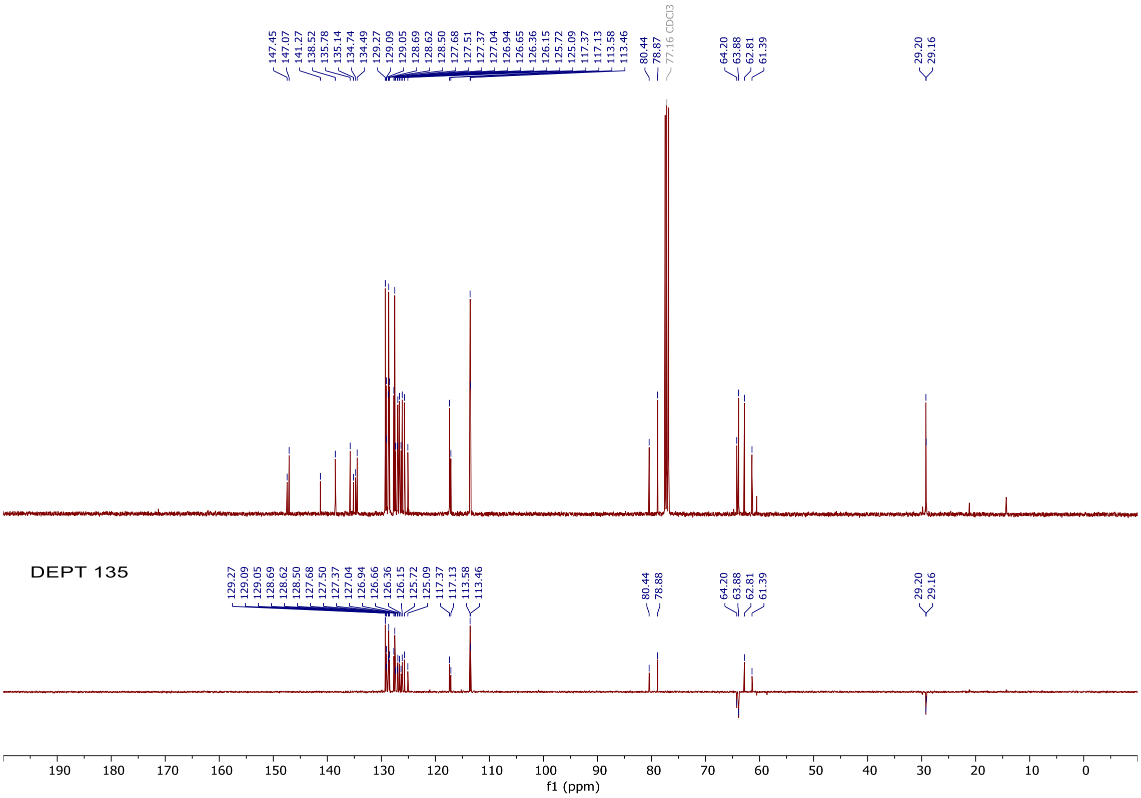

$^{1}H$ NMR (400 MHz, $CDCl_3$) **9**

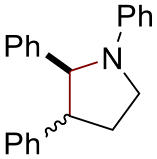

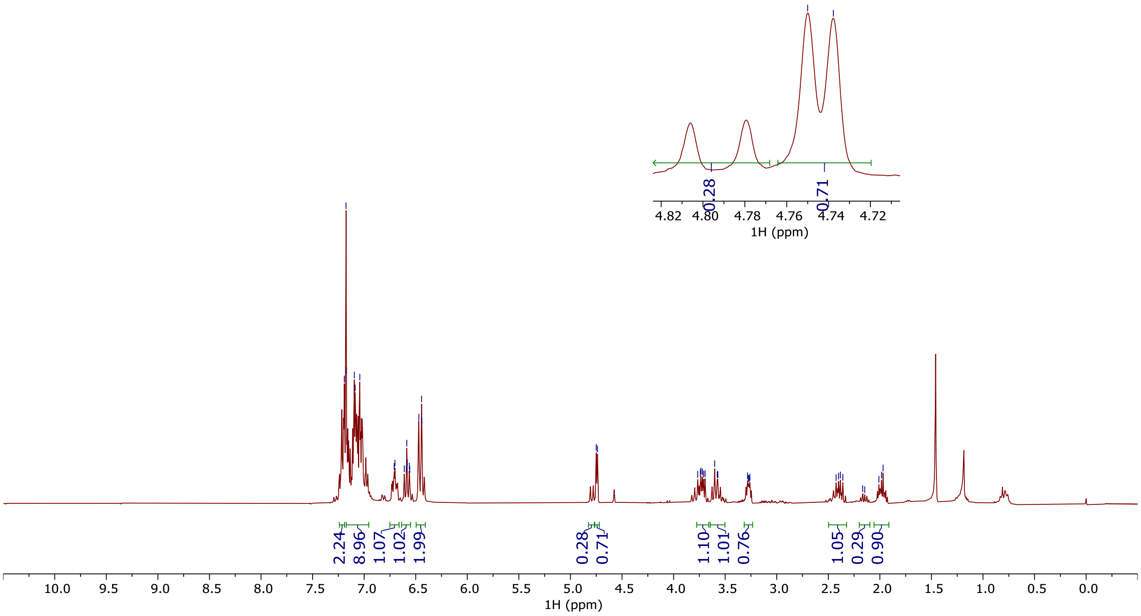

$^{13}C$ NMR(101MHz,$CDCl_3$) **9**

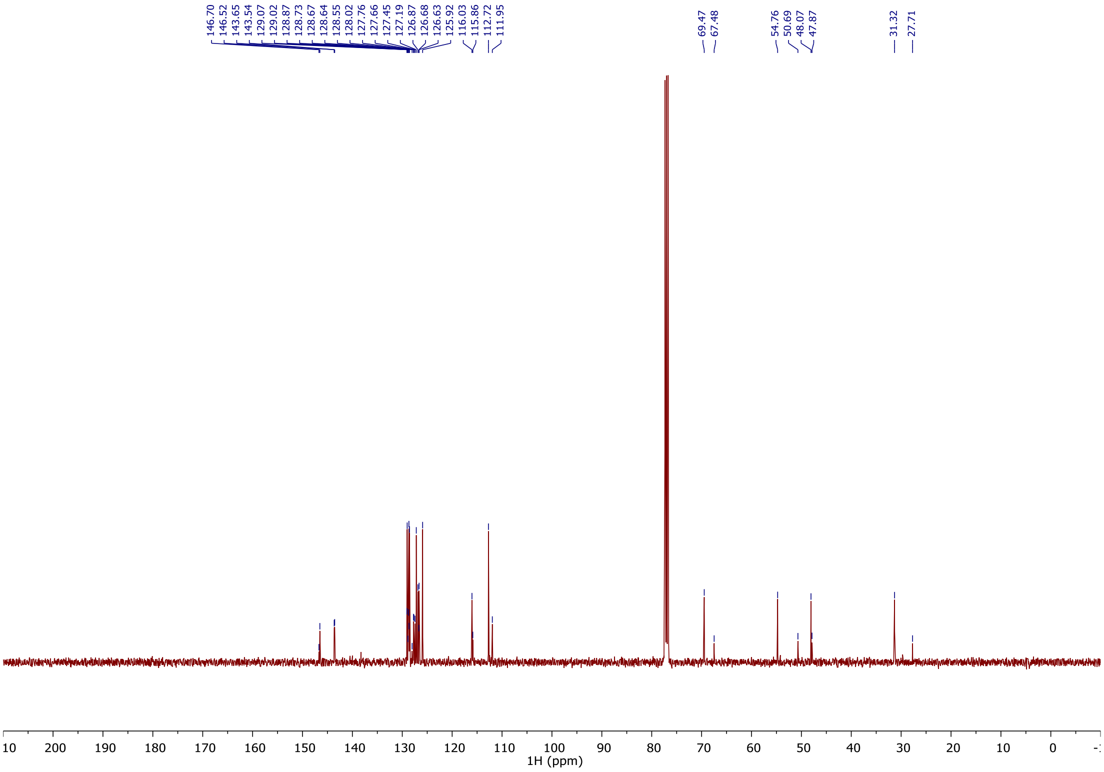

$^{1}H$ NMR (400 MHz, $CDCl_3$) **10**

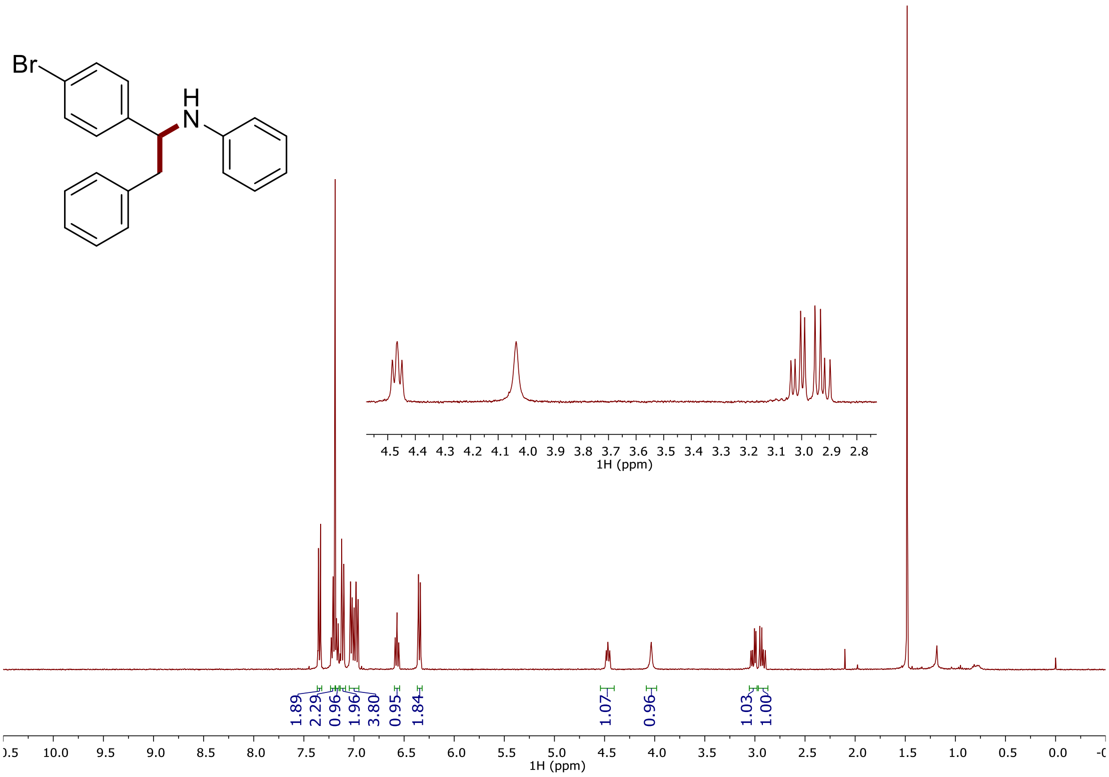


$^{13}C$ NMR(101MHz,$CDCl_3$) **10**

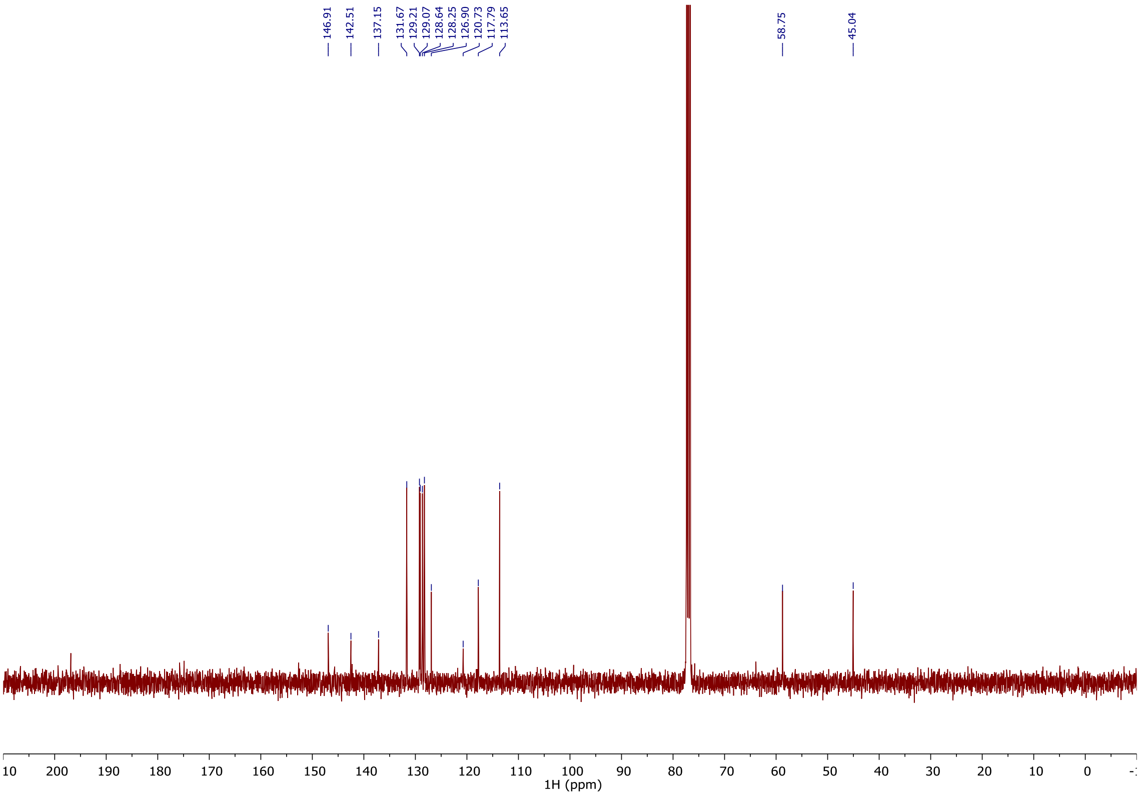

$^{1}$H NMR (400 MHz, $CDCl_3$) **11**

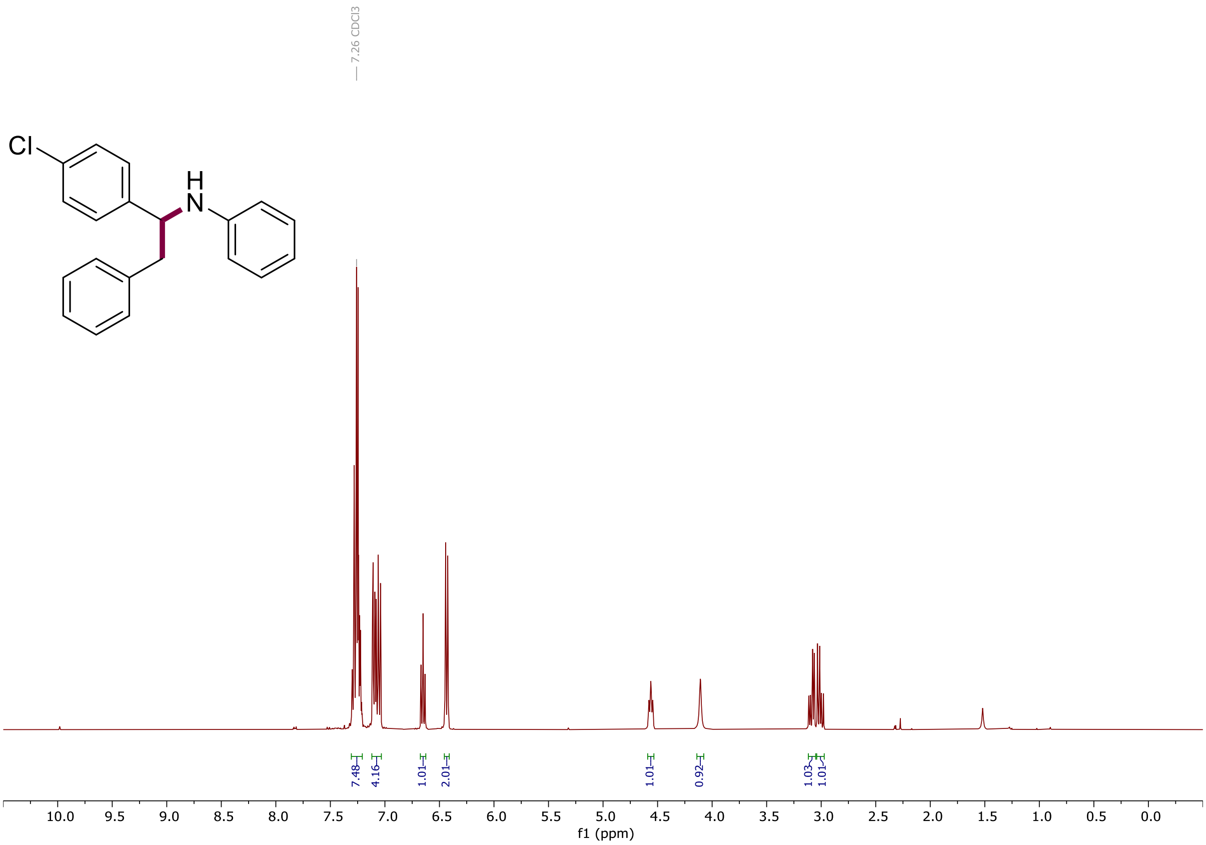


$^{13}$C NMR (101 MHz, $CDCl_3$) **11**

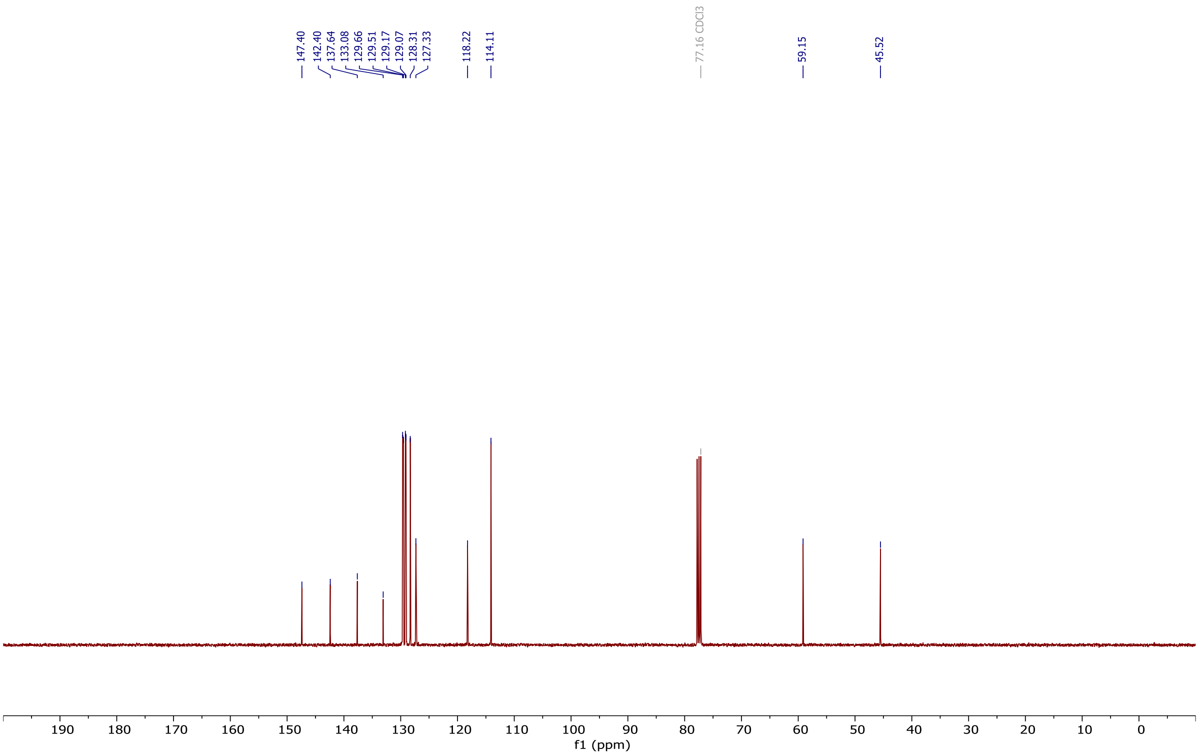

$^{1}H$ NMR (400 MHz, $CDCl_3$) **12**

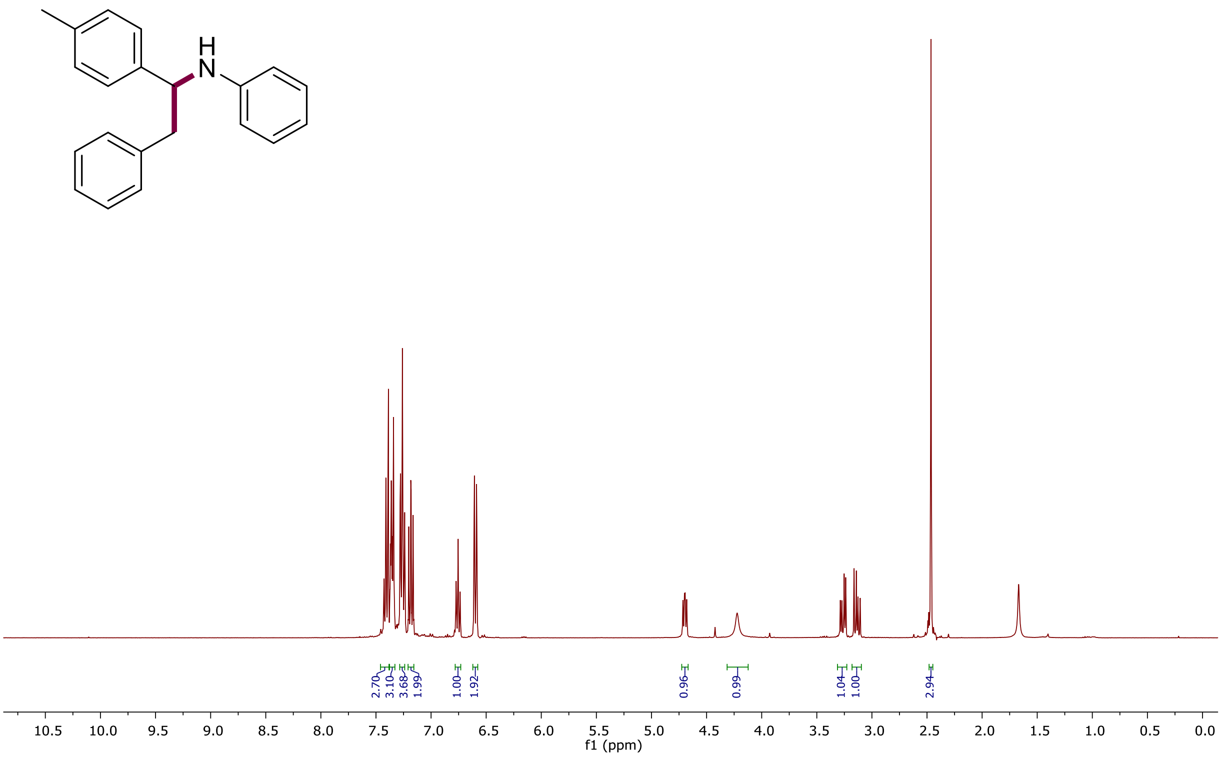


$^{13}C$ NMR (101 MHz, $CDCl_3$) **12**

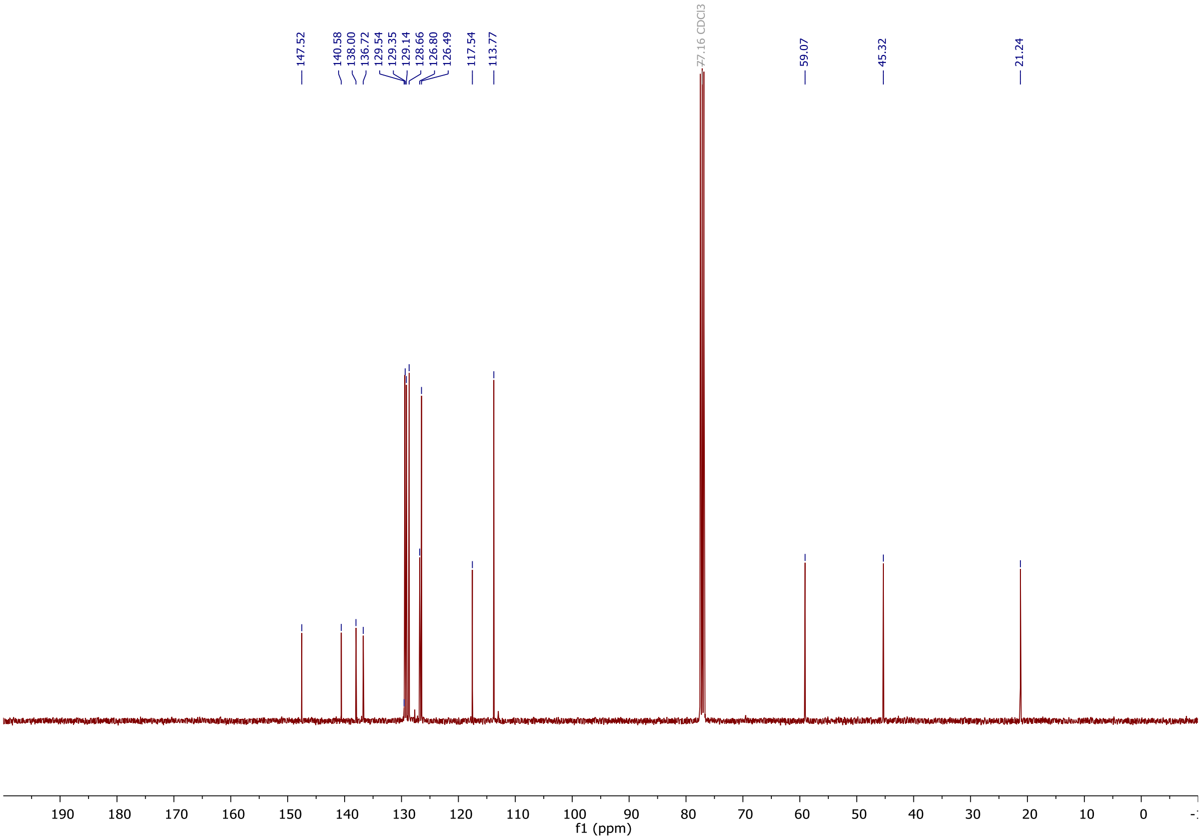

## $^{1}H$ NMR (400 MHz, $CDCl_3$) **13**

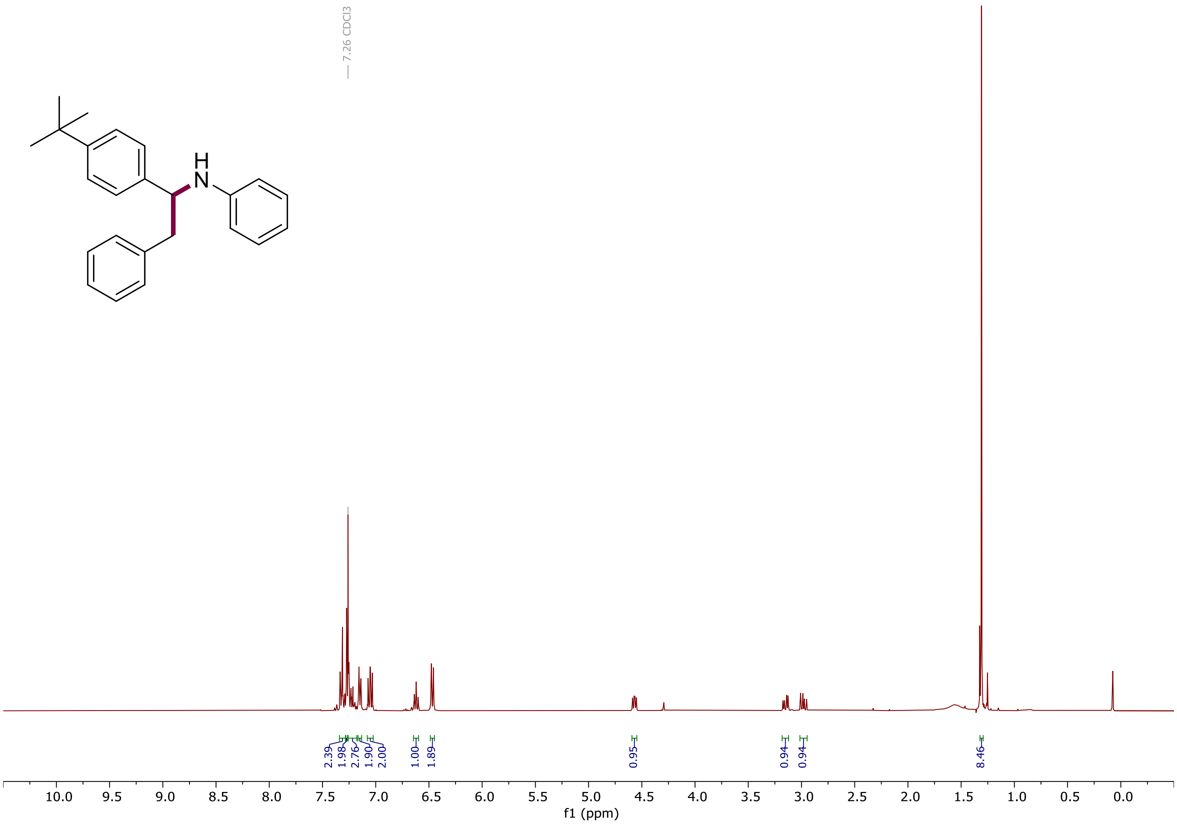


## $^{13}C$ NMR (101 MHz, $CDCl_3$) **13**

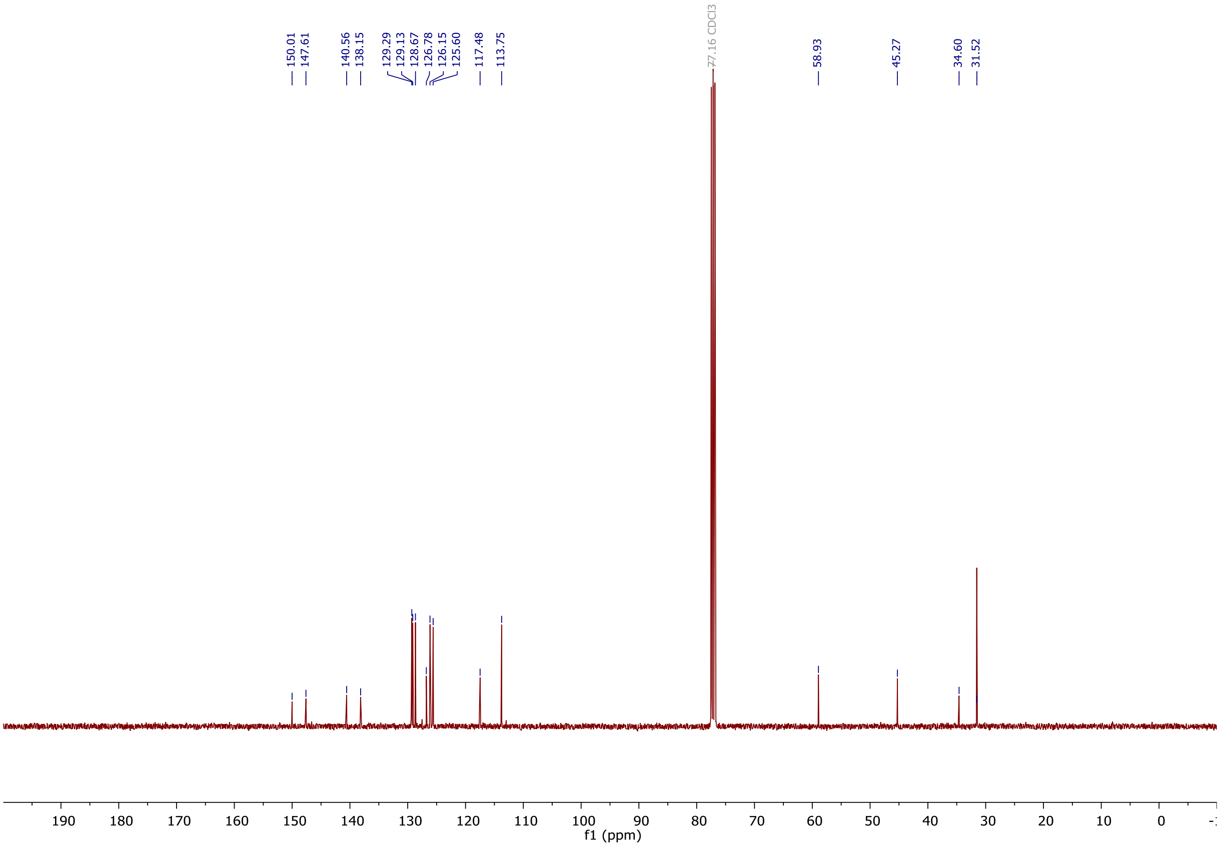

$^{1}H$ NMR (400 MHz, $CDCl_3$) **14**

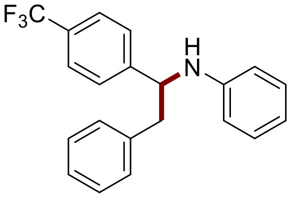


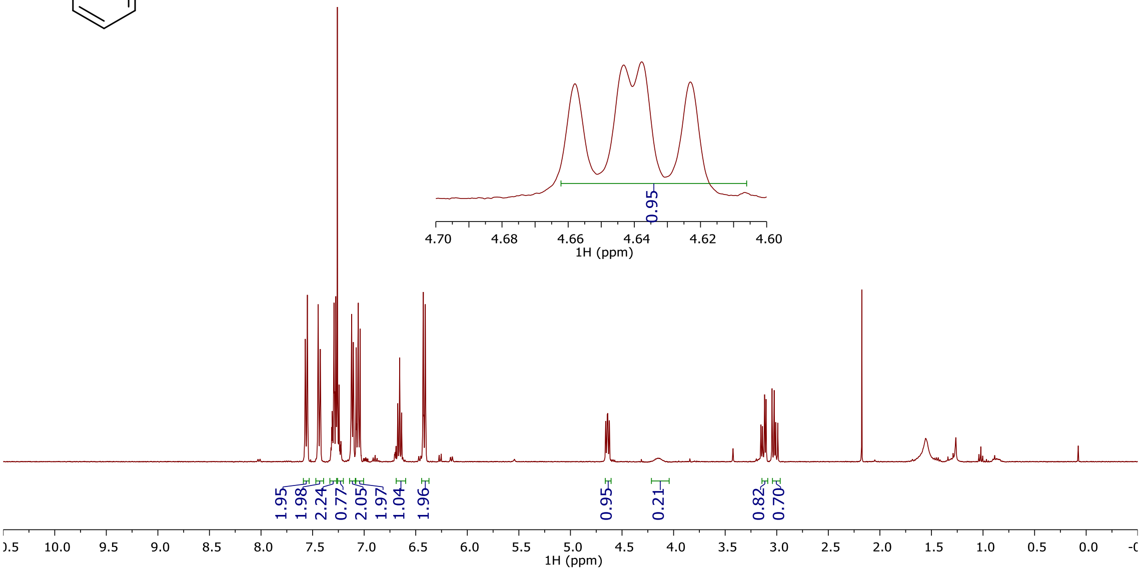

$^{13}C$ NMR(101MHz,$CDCl_3$) **14**

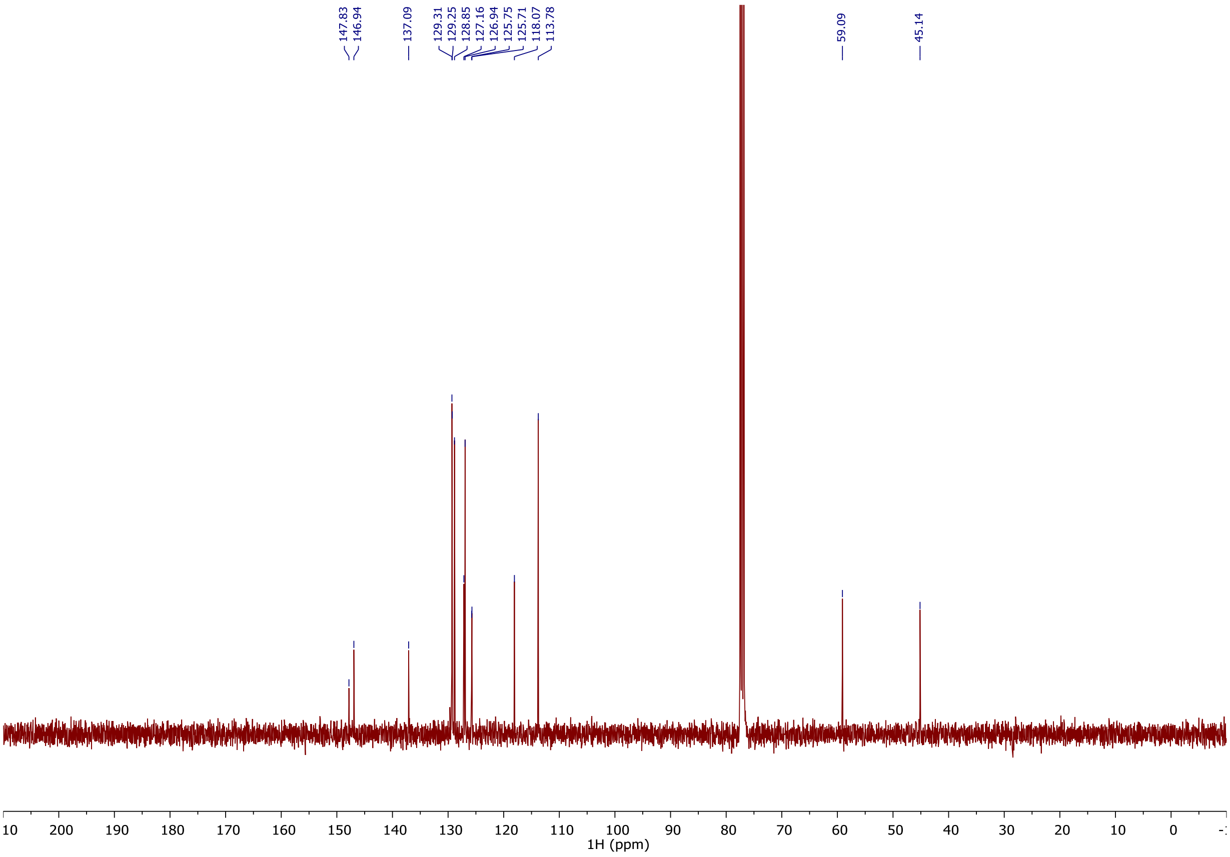

$^{19}$F NMR (282 MHz, $CDCl_3$) **14**

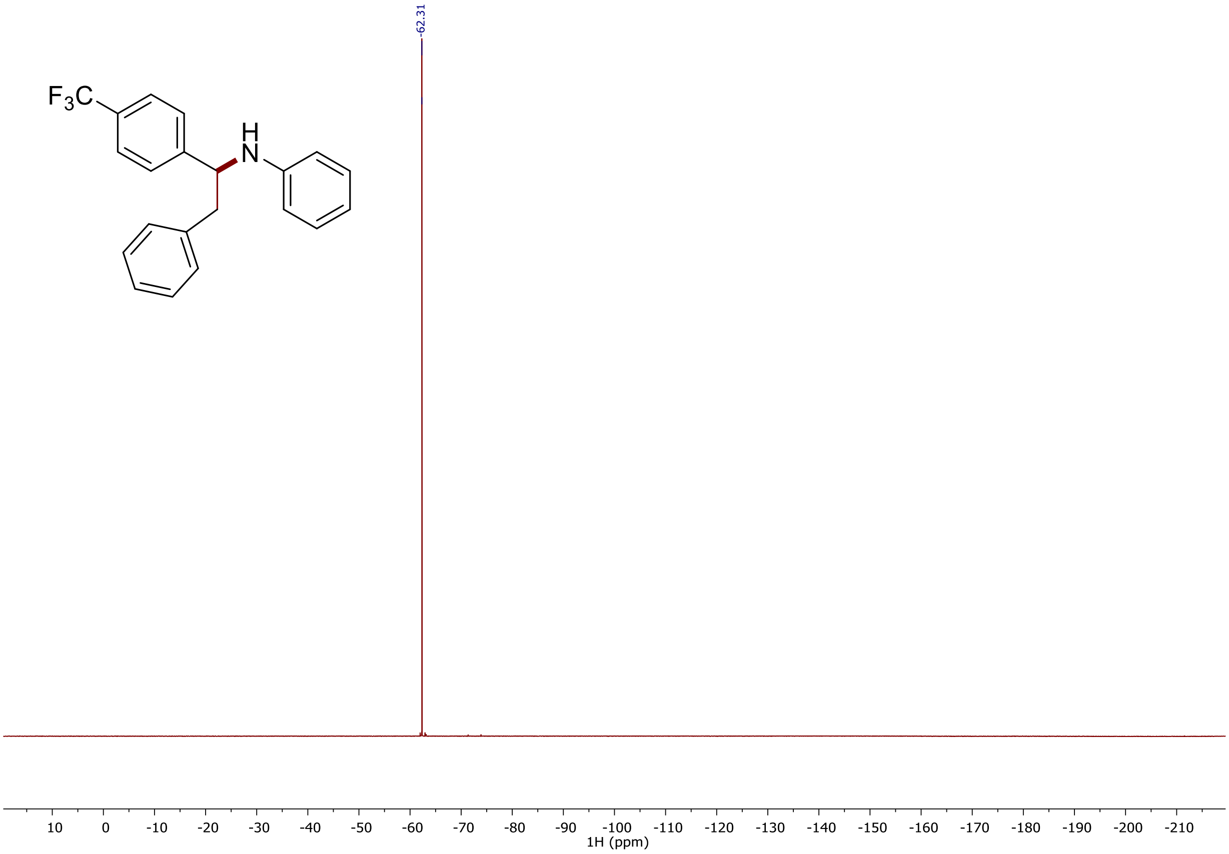

$^1$H NMR (400 MHz, $CDCl_3$) **15**

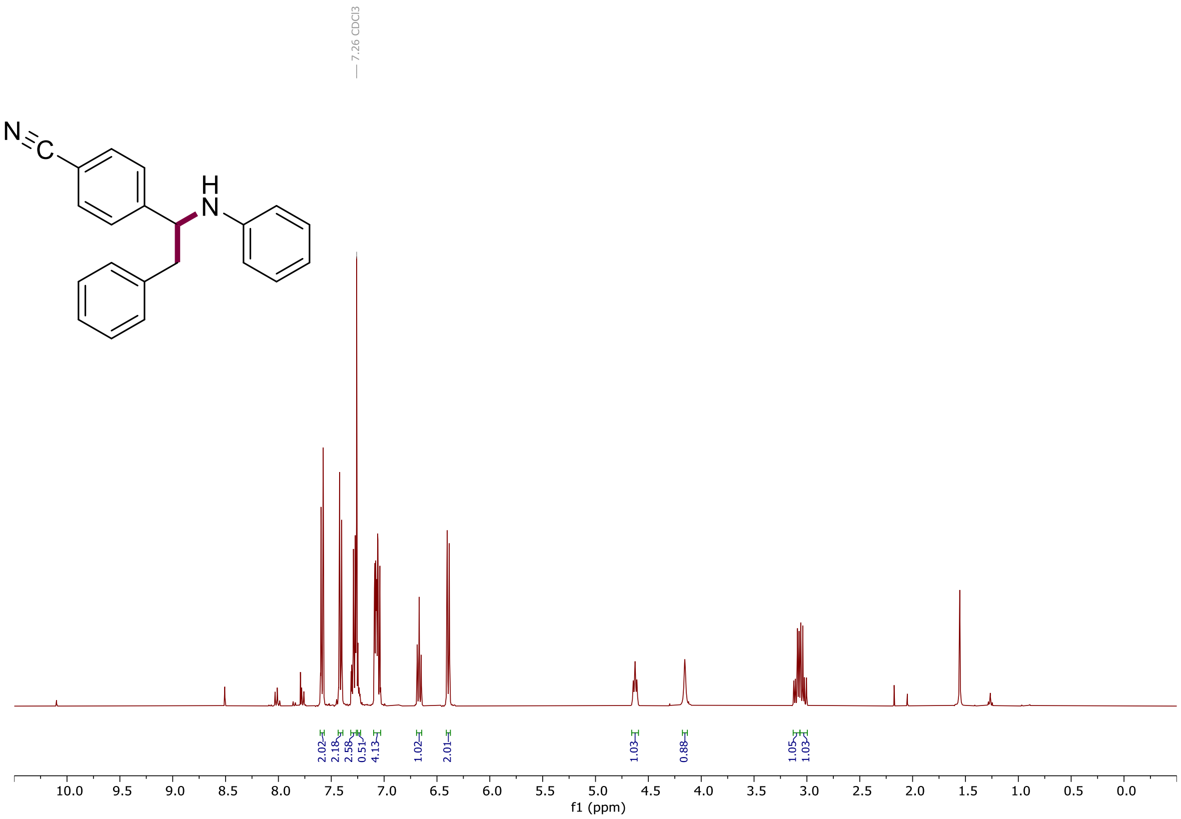


$^{13}$C NMR (101 MHz, $CDCl_3$) **15**

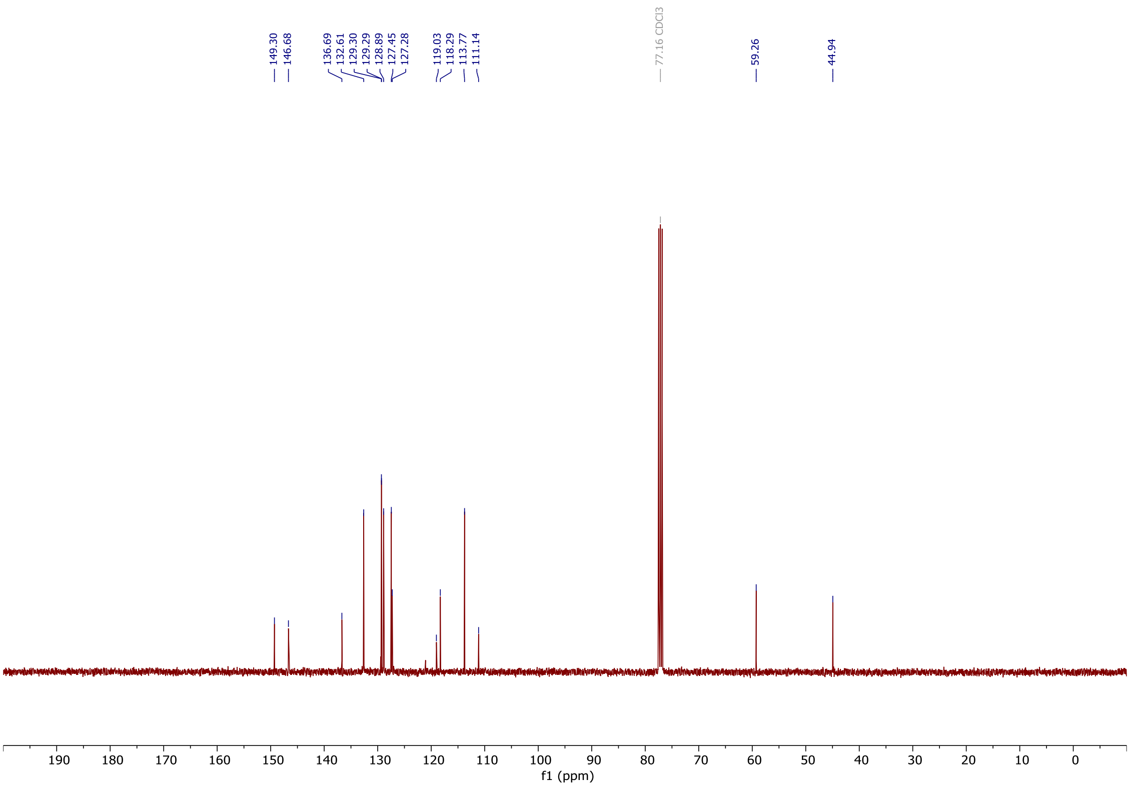

$^{1}H$ NMR (400 MHz, $CDCl_3$) **16**

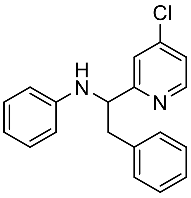

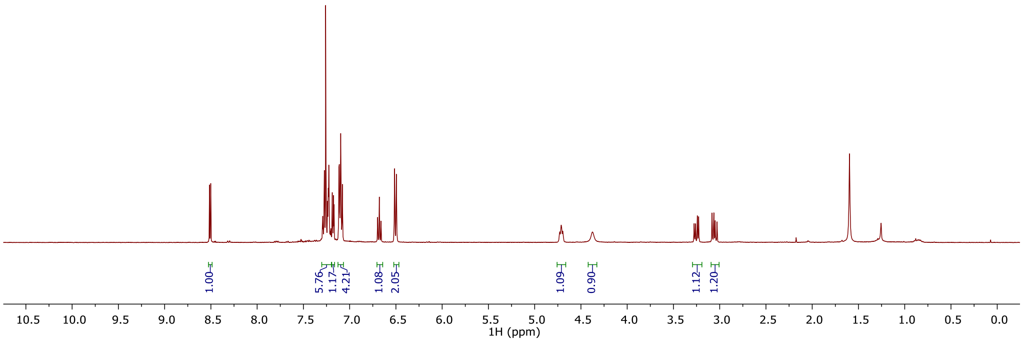


$^{13}C$ NMR(101MHz,$CDCl_3$) **16**

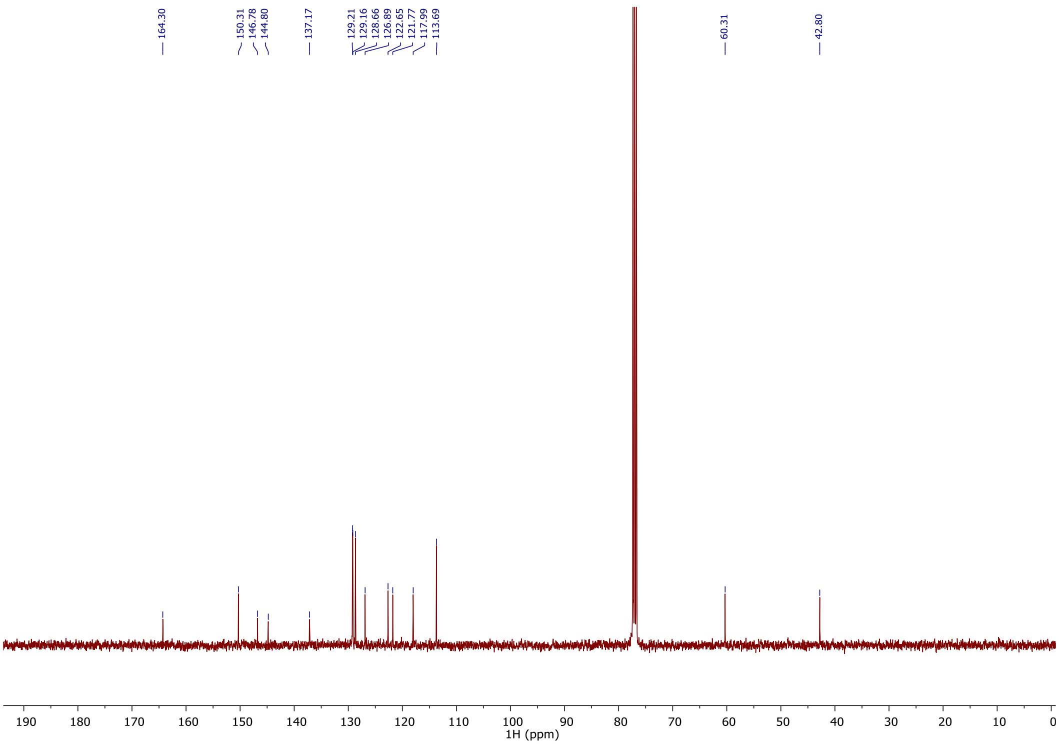

$^{1}$H NMR (400 MHz, $CDCl_3$) **17**

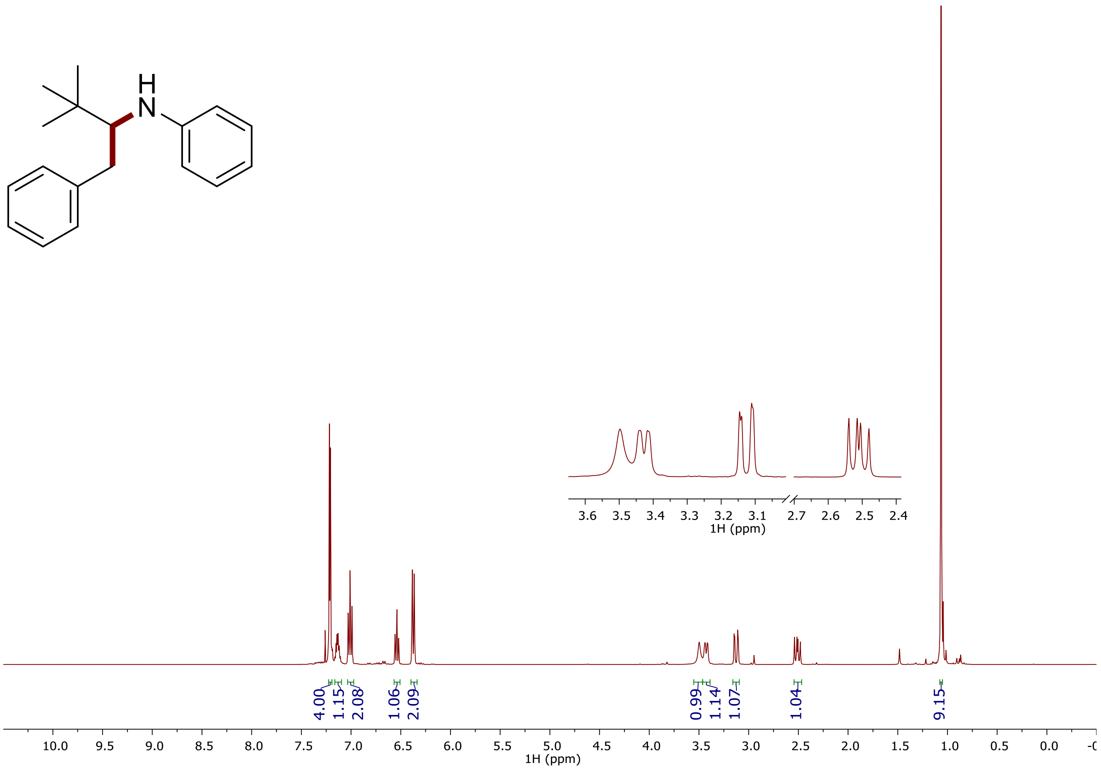


$^{13}$C NMR(101MHz,$CDCl_3$) **17**

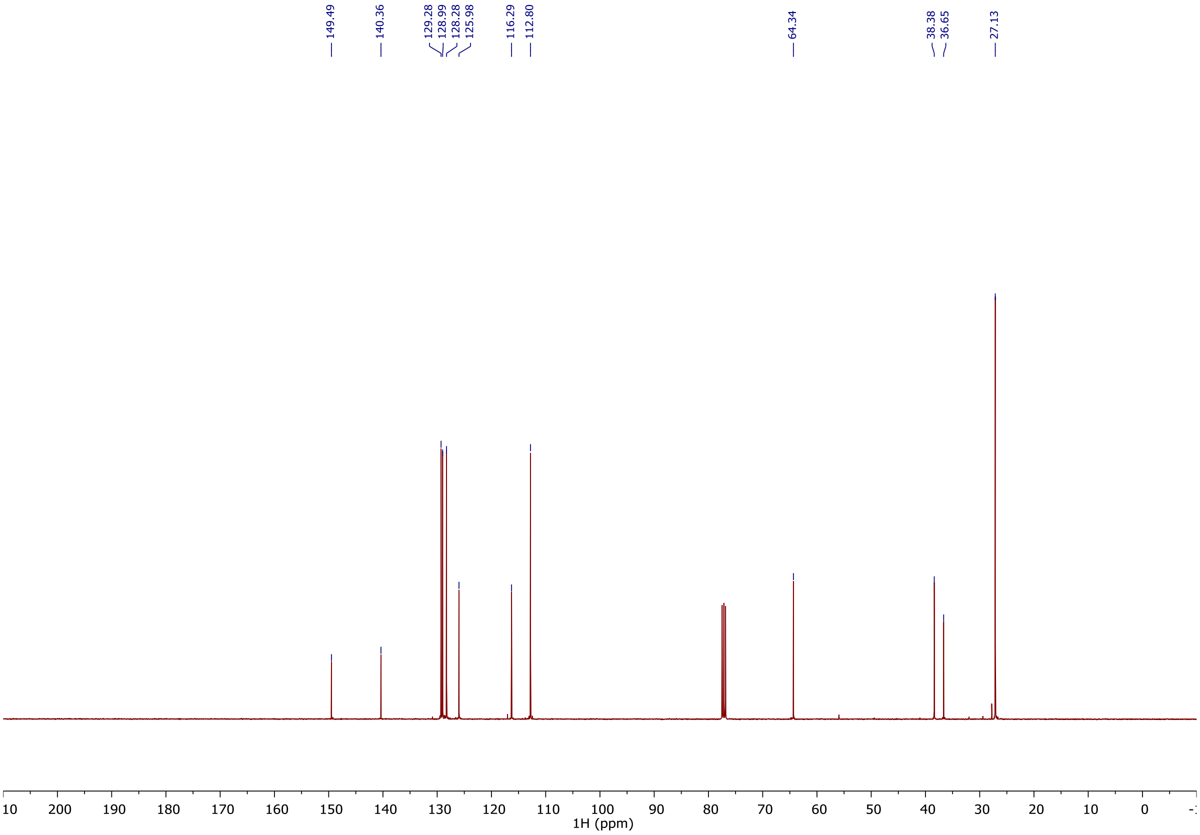

$^{1}$H NMR (400 MHz, $CDCl_3$) **18**

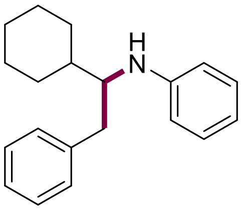


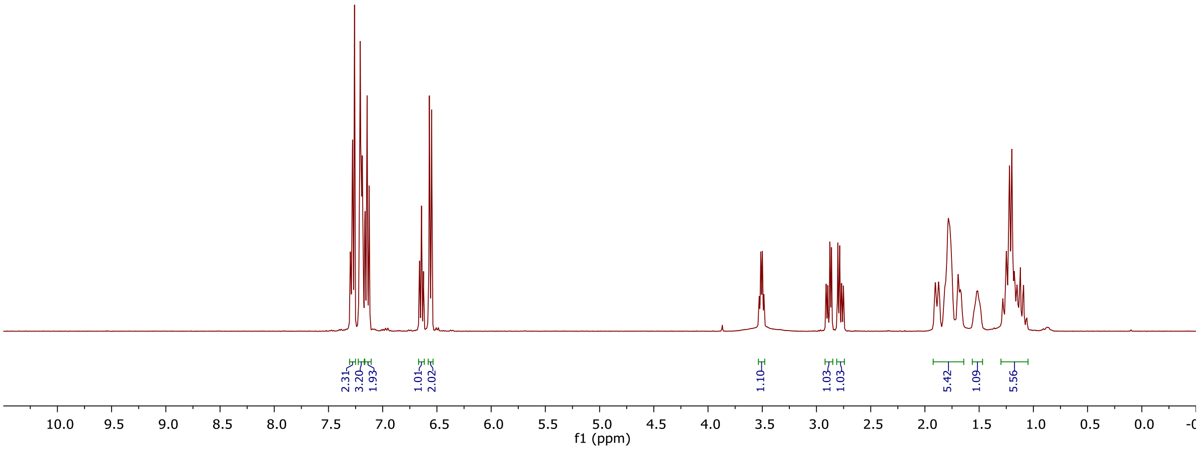


$^{13}$C NMR (101 MHz, $CDCl_3$) **18**

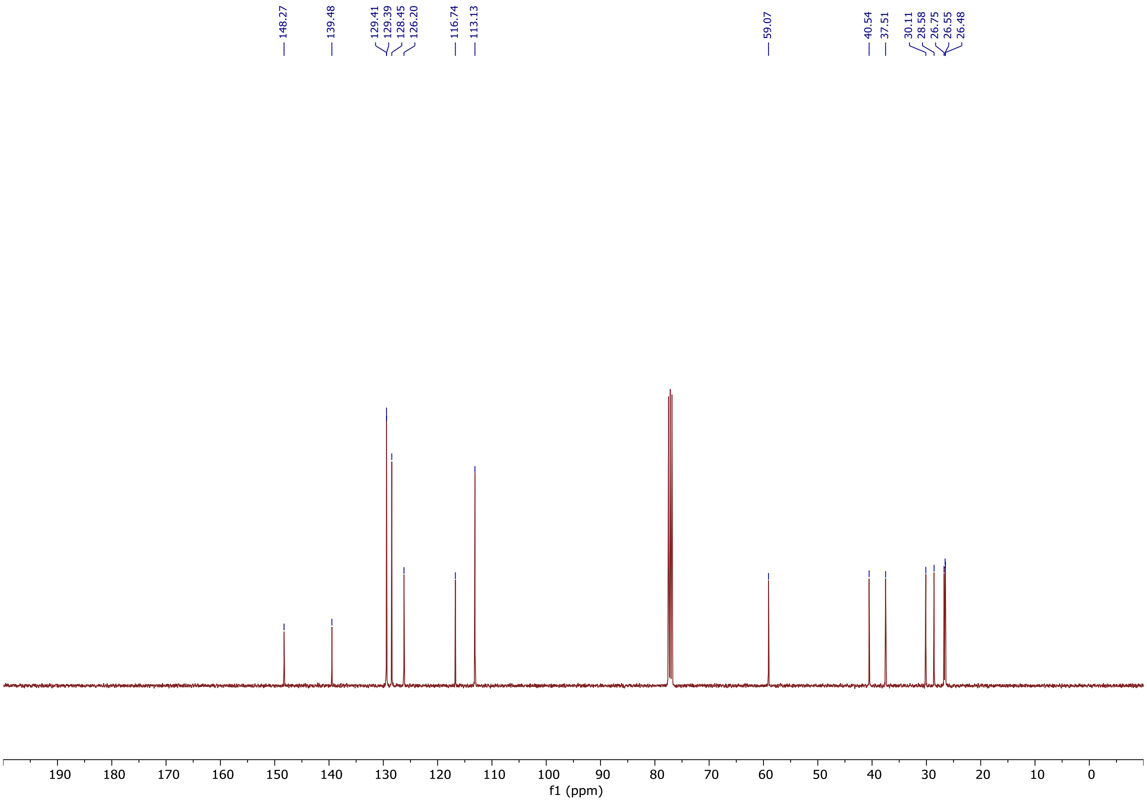

$^{1}$H NMR (400 MHz, $CDCl_3$) **19**

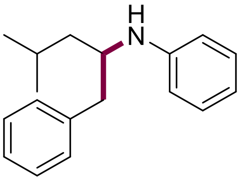


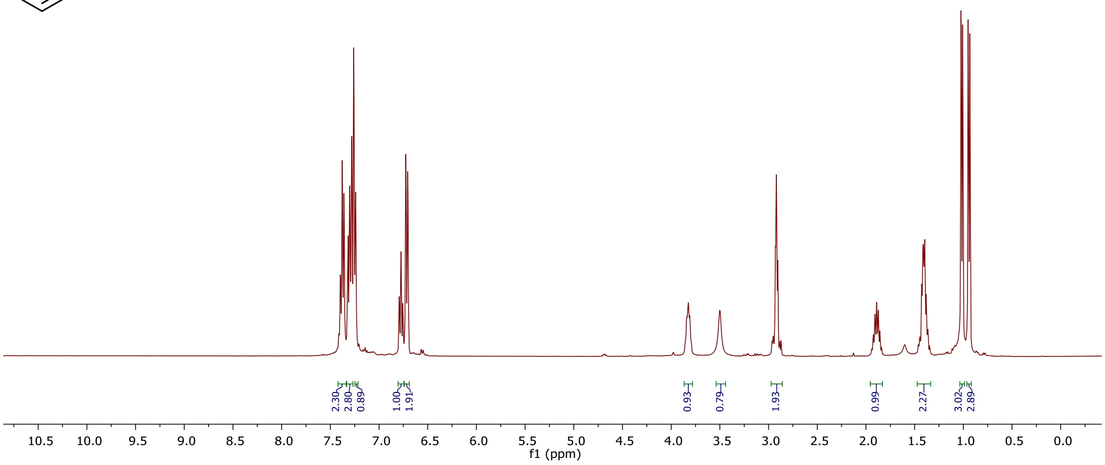


$^{13}$C NMR (101 MHz, $CDCl_3$) **19**

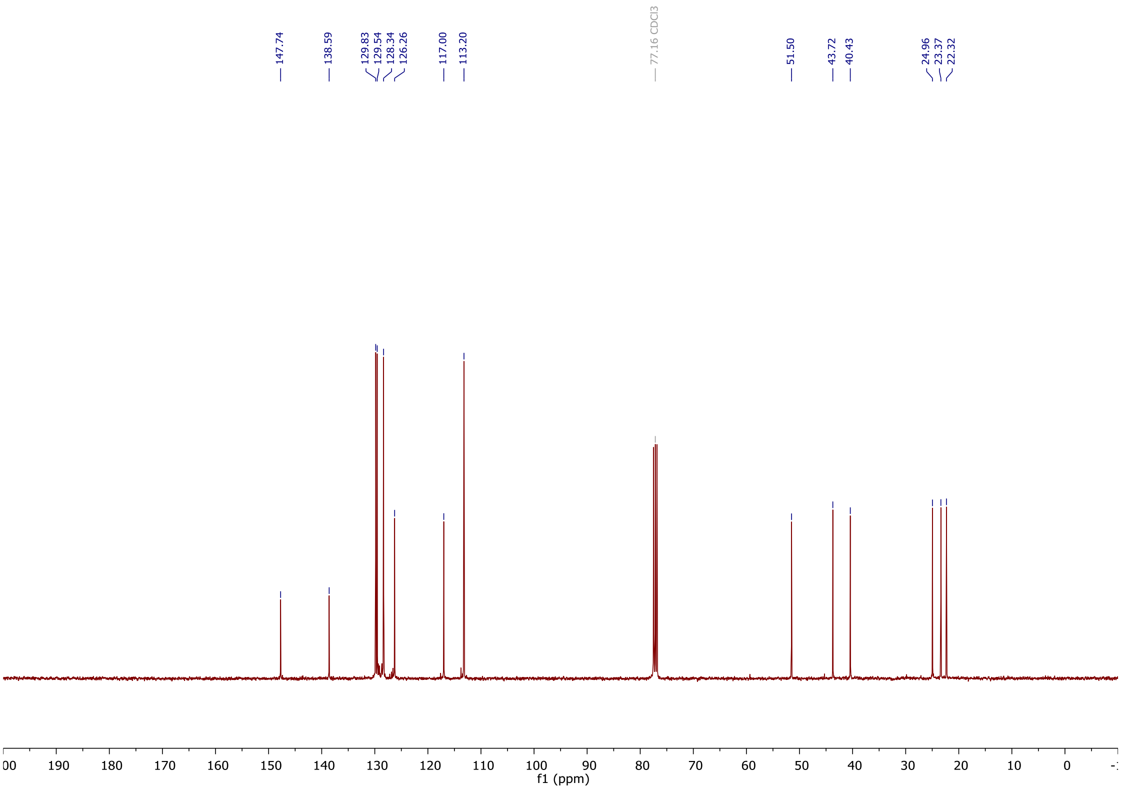

$^{1}$H NMR (400 MHz, $CDCl_3$) **20**

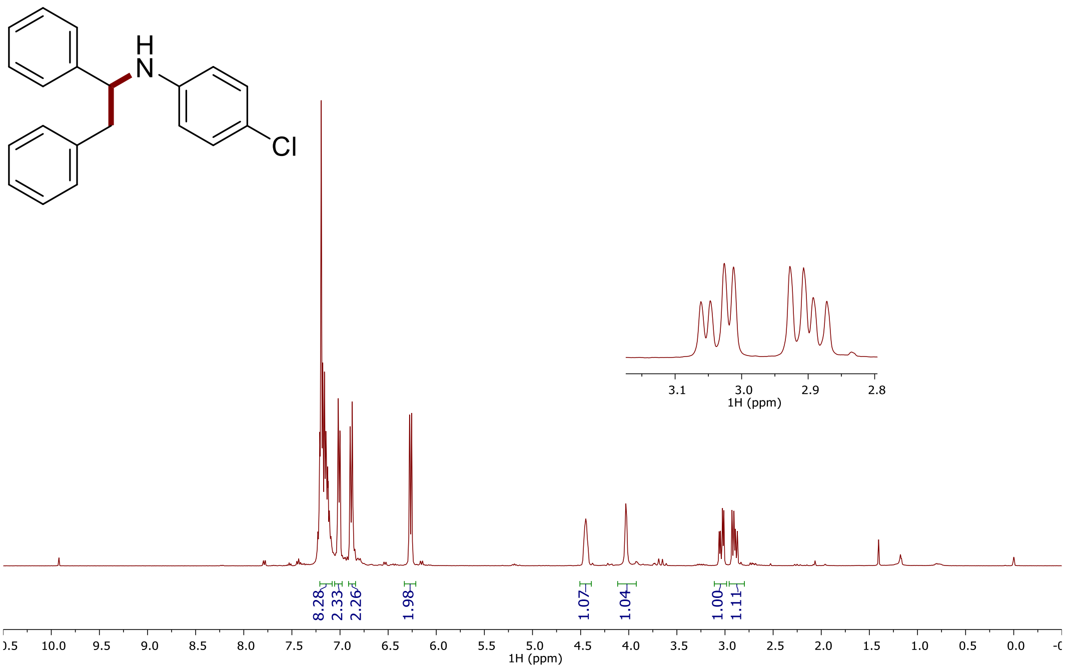


$^{13}$C NMR(101MHz,$CDCl_3$) **20**

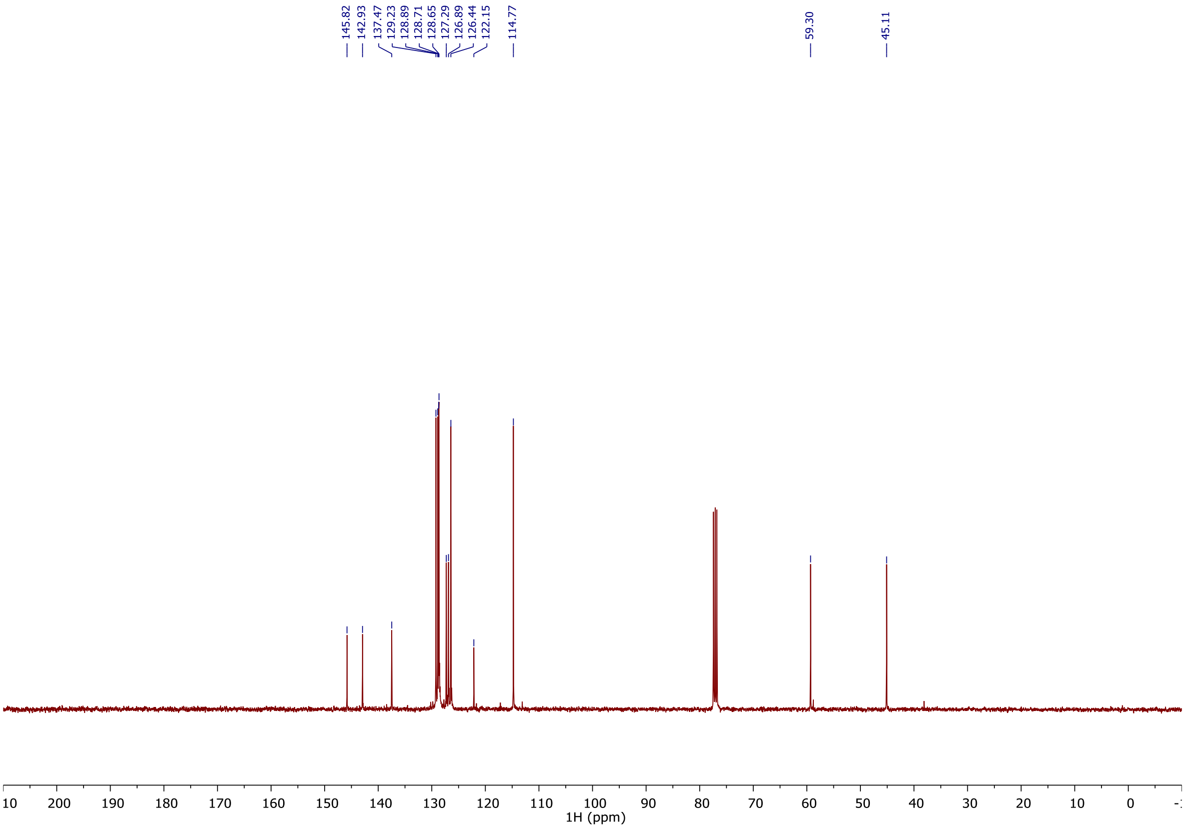

$^{1}H$ NMR (400 MHz, $CDCl_3$) **21**

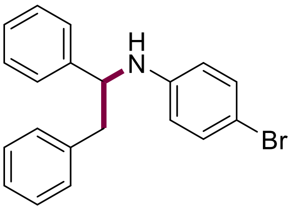


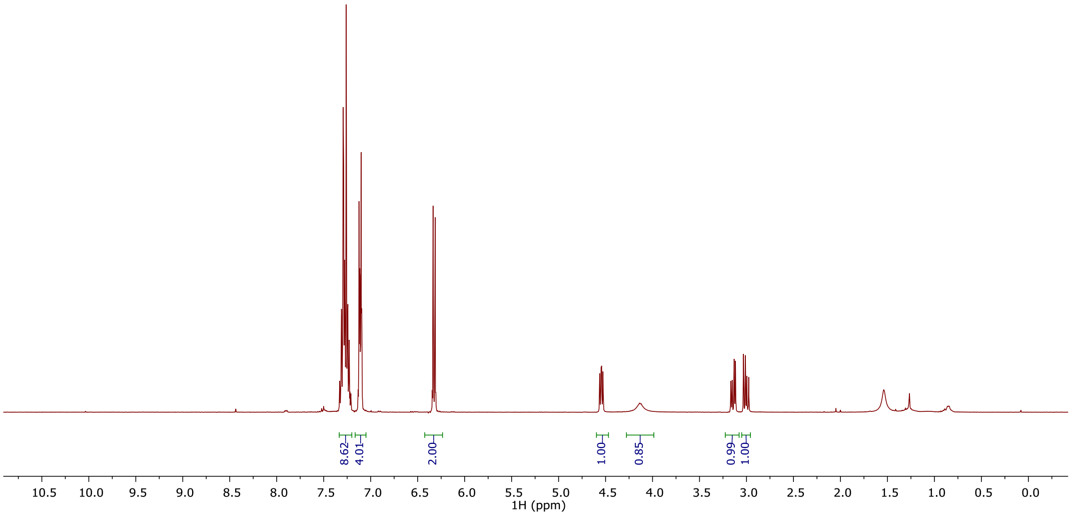


$^{13}C$ NMR(101MHz,$CDCl_3$) **21**

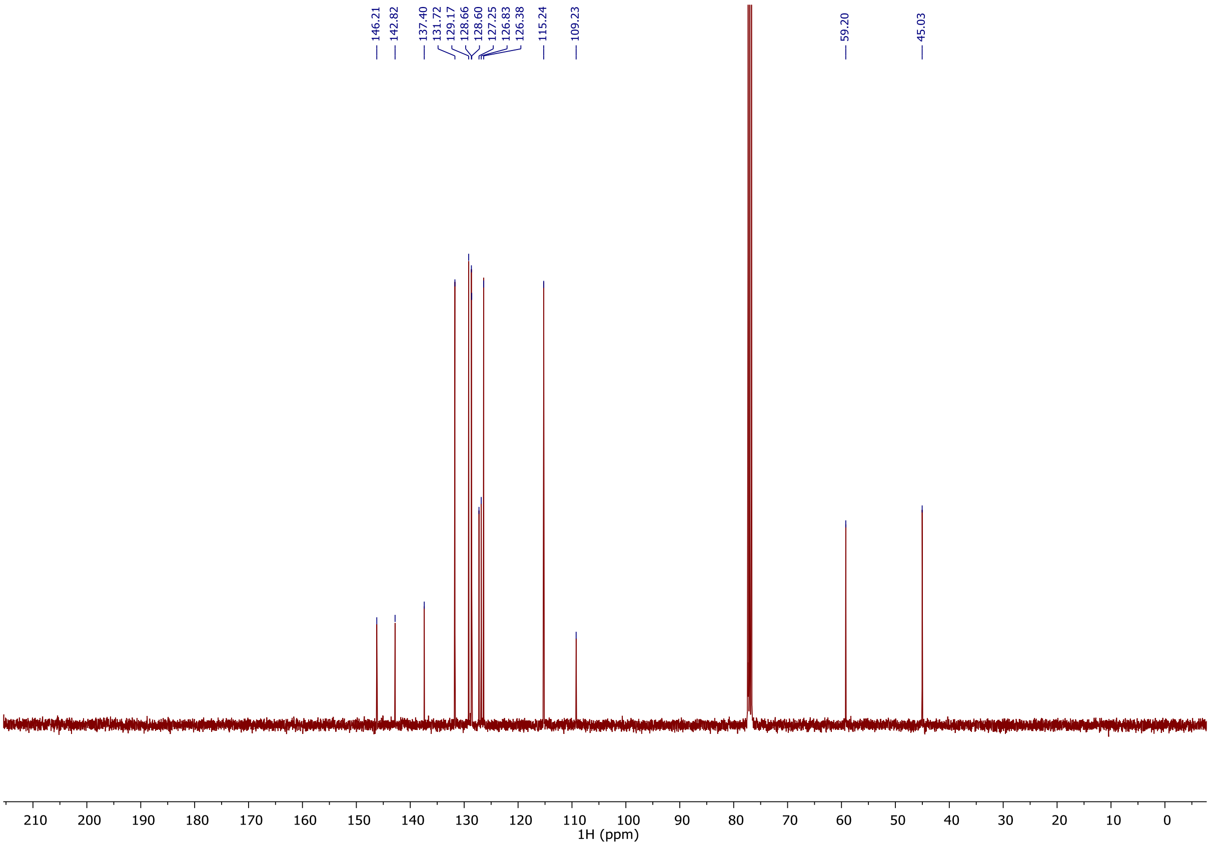

$^{1}$H NMR (400 MHz, $CDCl_3$) **22**

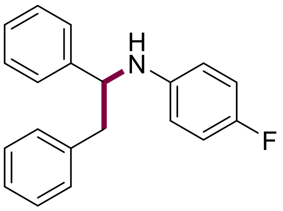


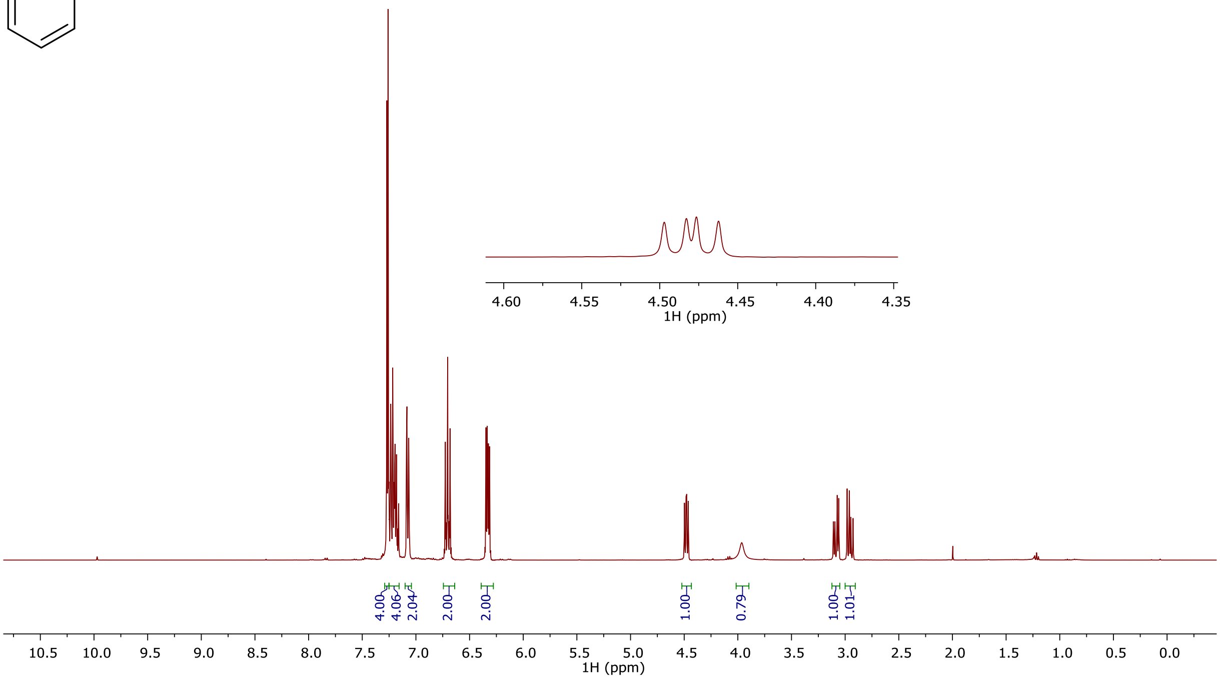


$^{13}$C NMR(101MHz,$CDCl_3$) **22**

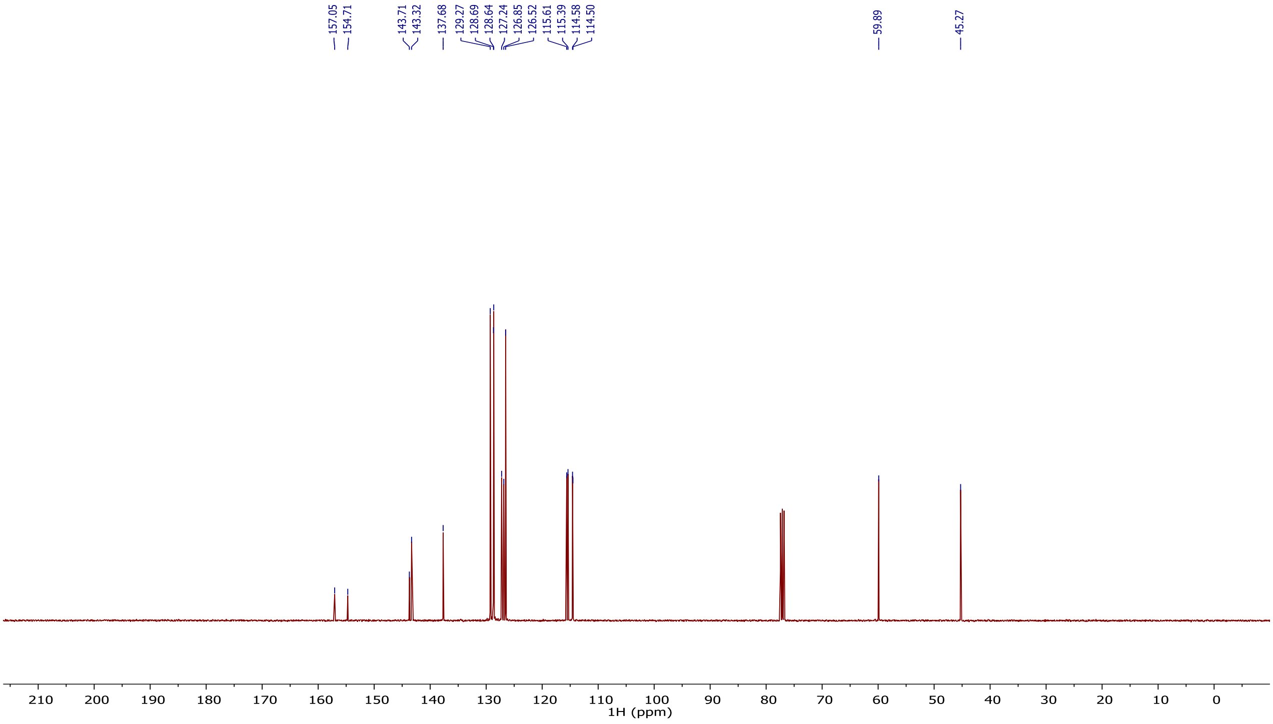

$^{19}$F NMR(377 MHz,CDCl$_3$) **22**

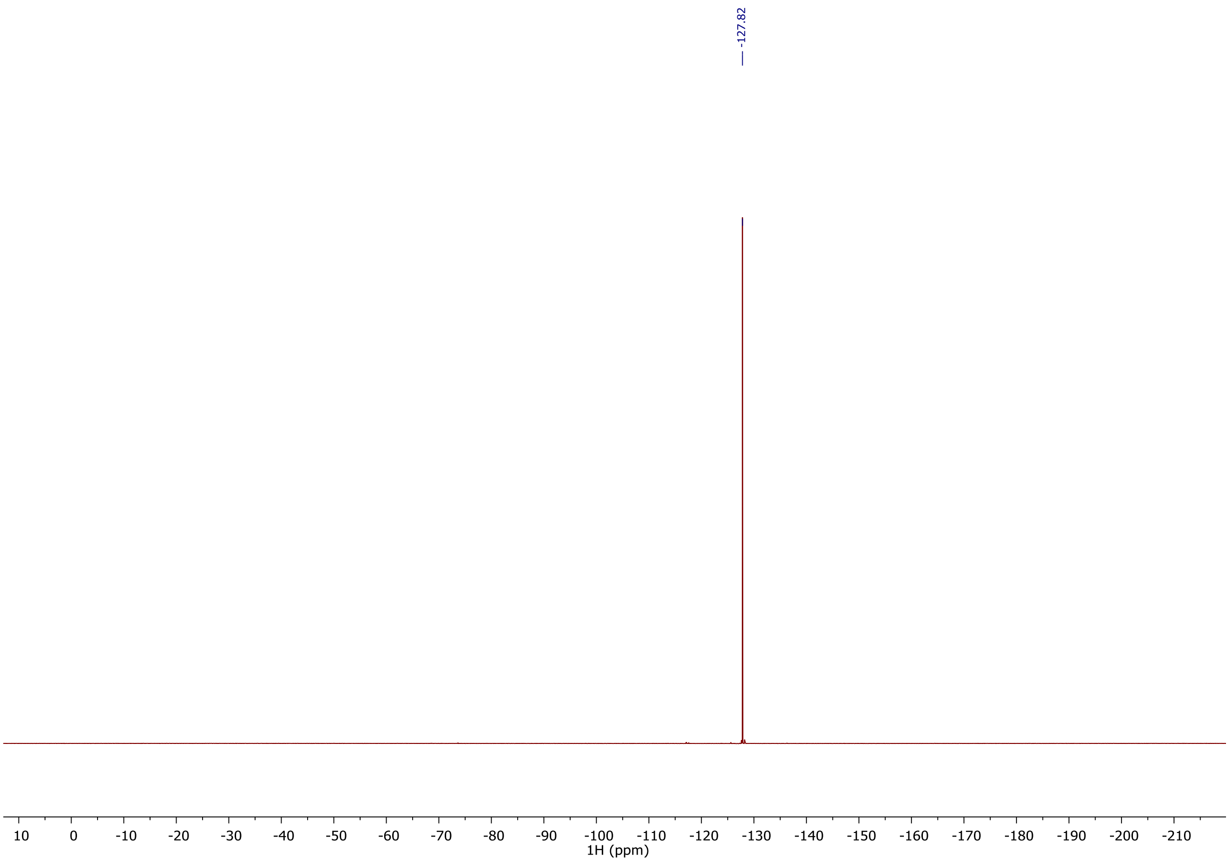

$^{1}$H NMR (400 MHz, $CDCl_3$) **23**

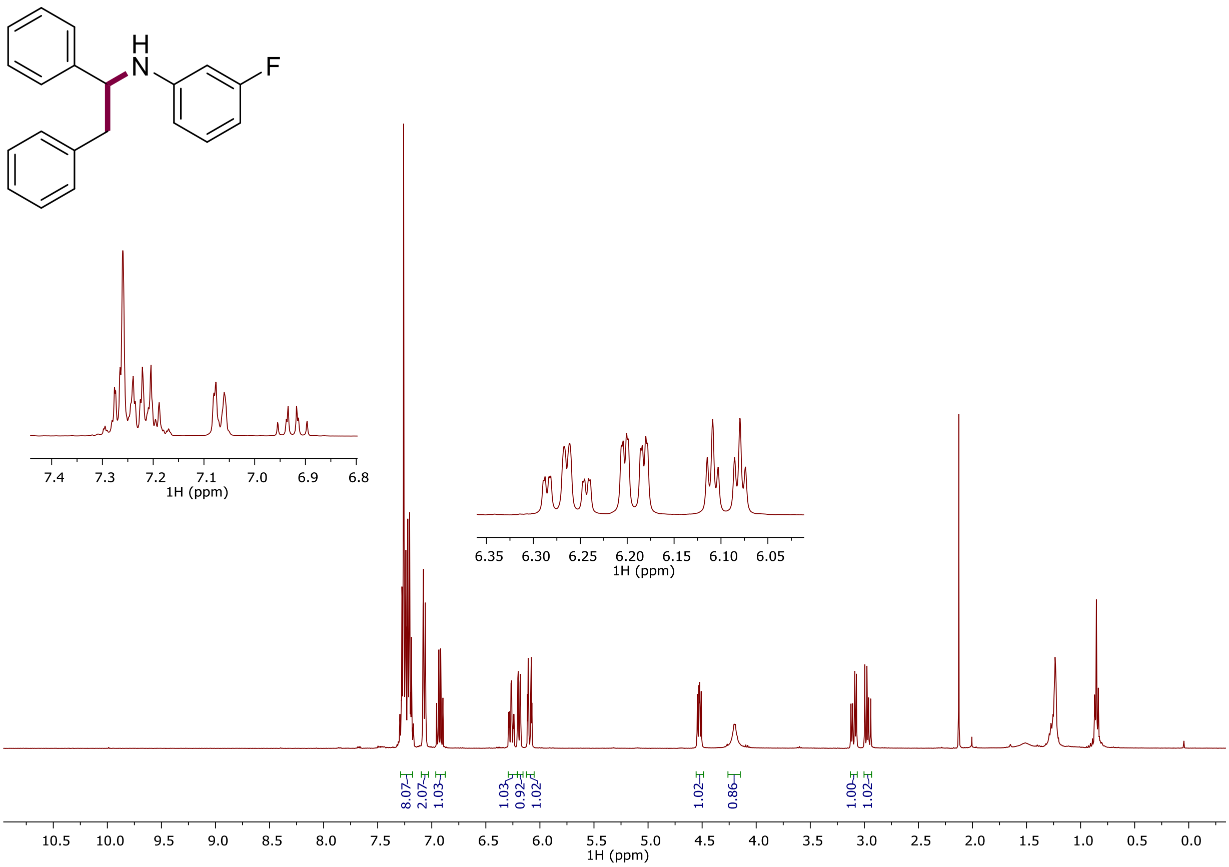


$^{13}$C NMR(101MHz,$CDCl_3$) **23**

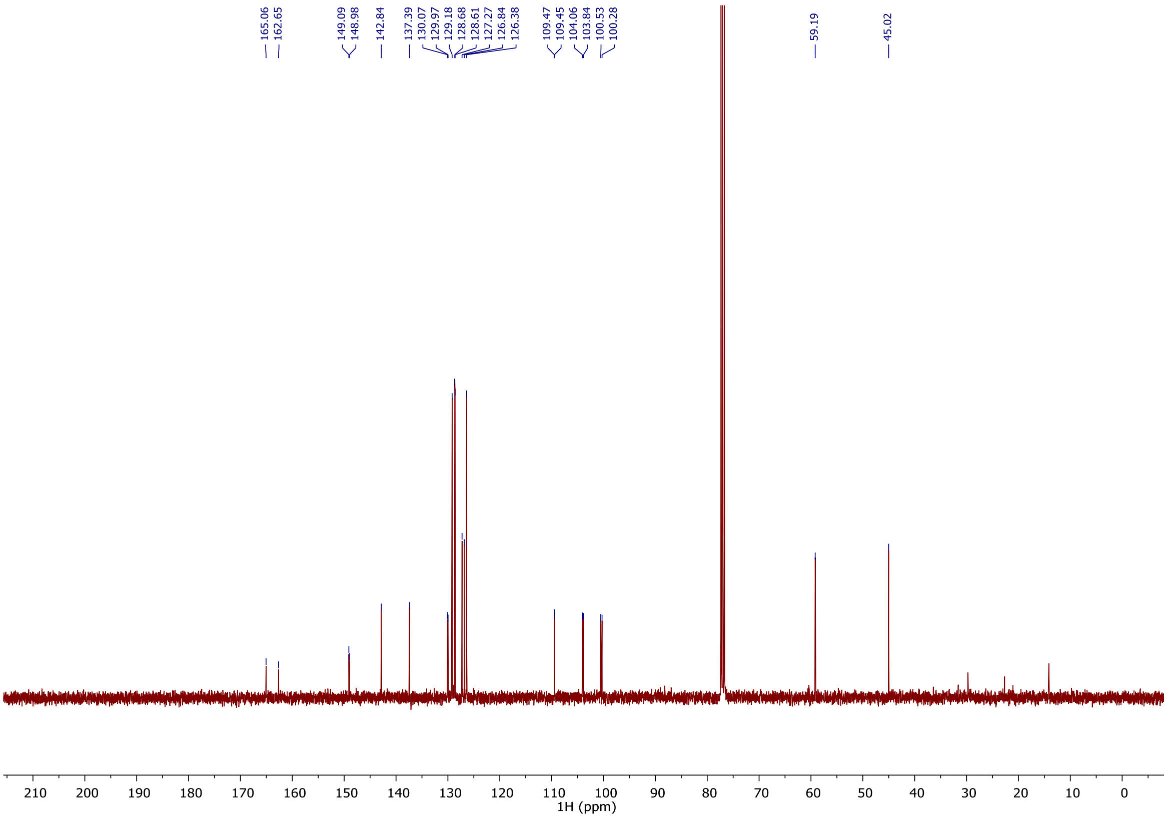

$^{19}$F NMR(377 MHz,$CDCl_3$) **23**

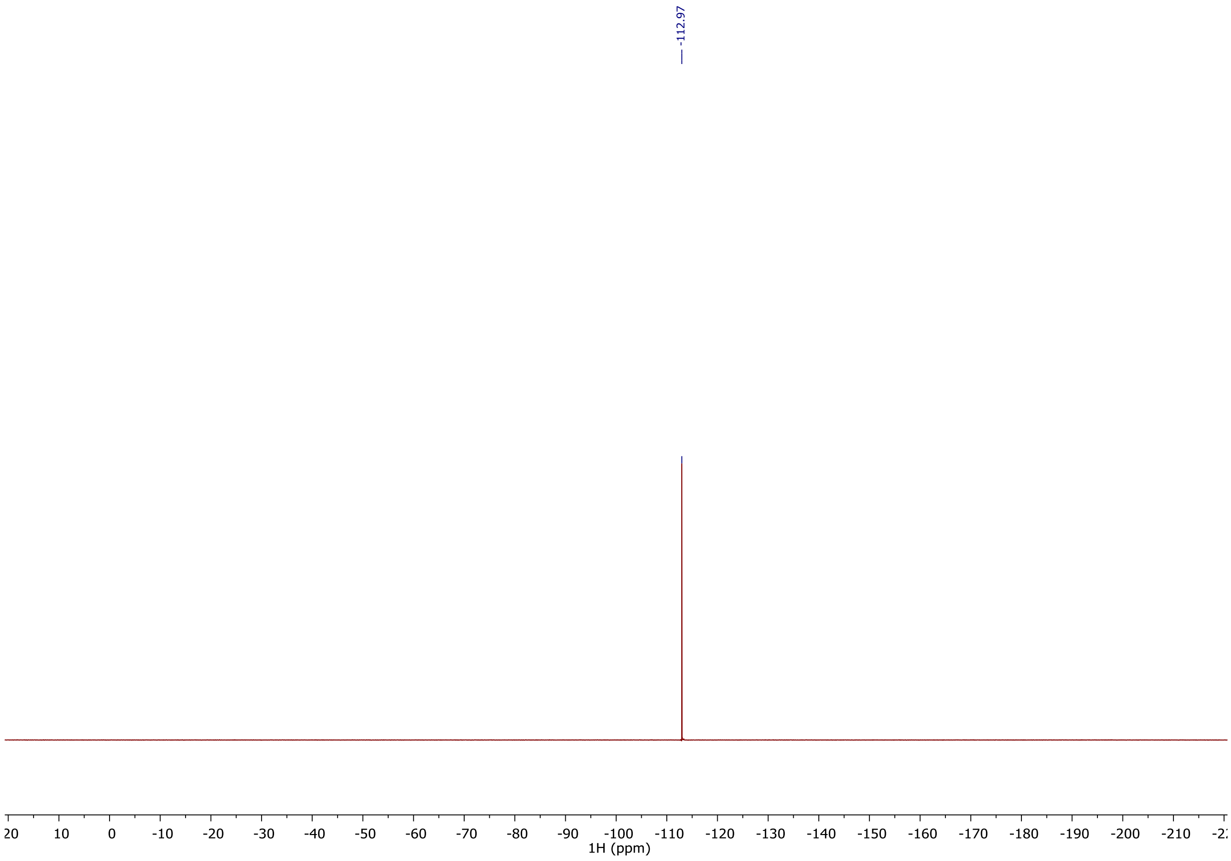

$^{1}$H NMR (400 MHz, $CDCl_3$) **24**

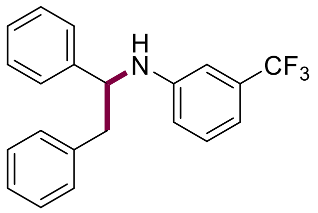


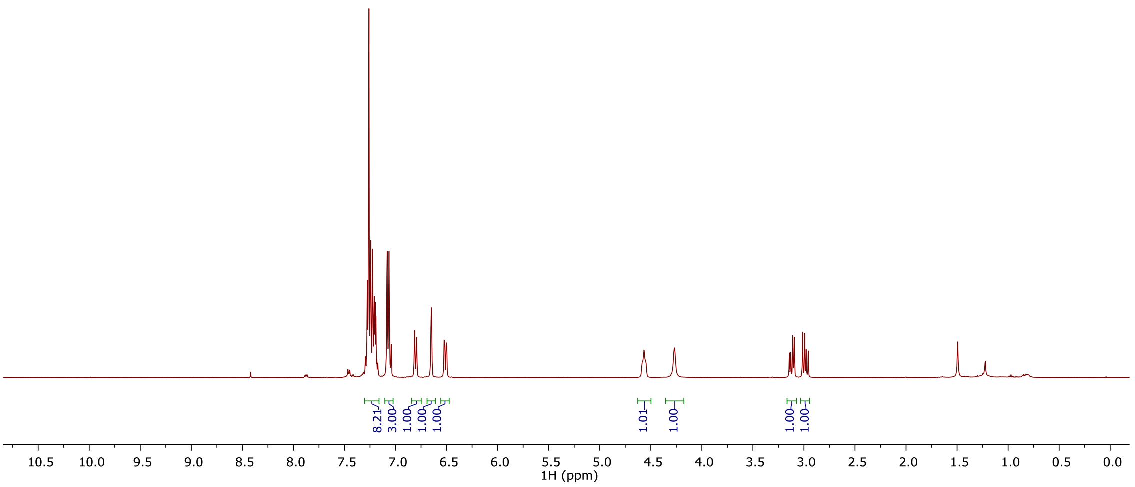


$^{13}$C NMR(101MHz,$CDCl_3$) **24**

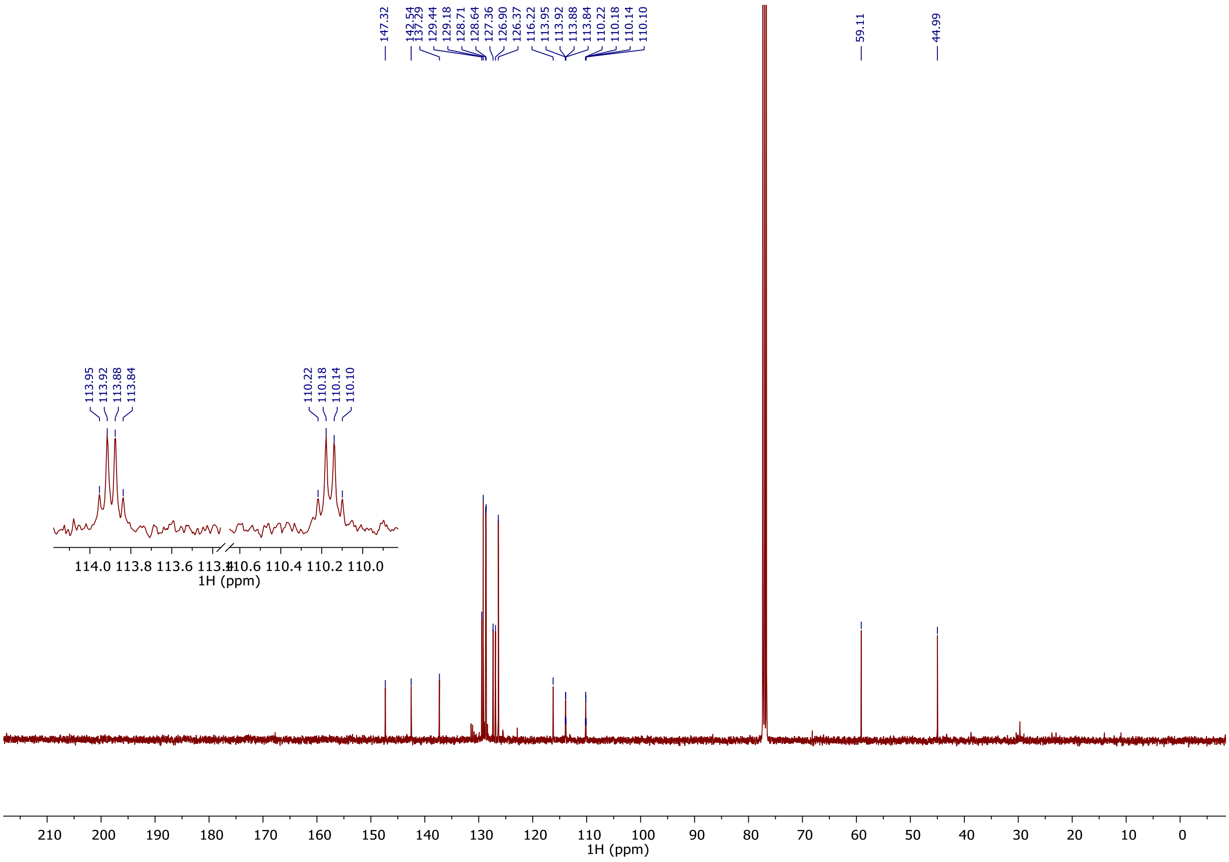

$^{19}$F NMR(377 MHz,$CDCl_3$) **24**

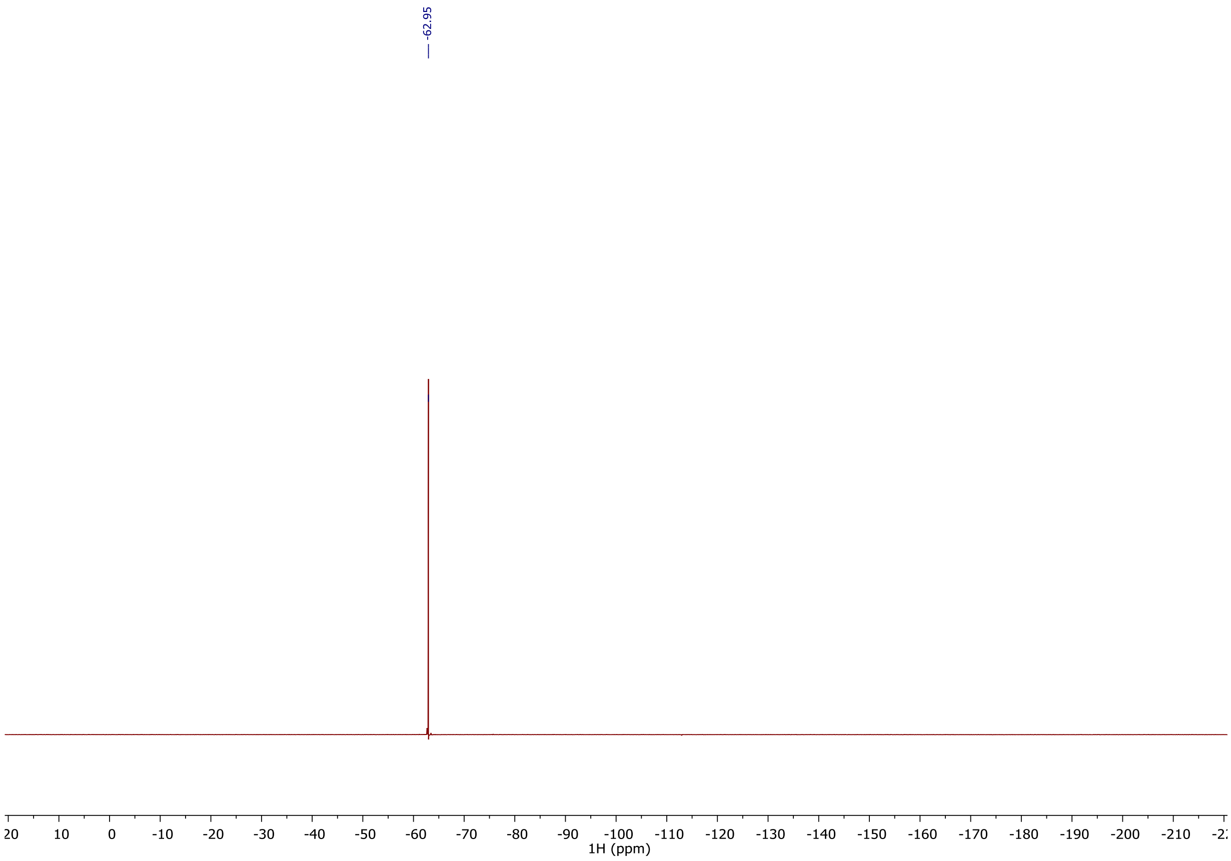

$^{1}$H NMR (400 MHz, $CDCl_3$) **25**

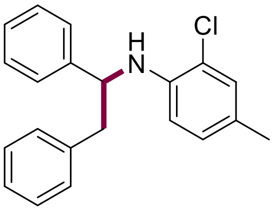


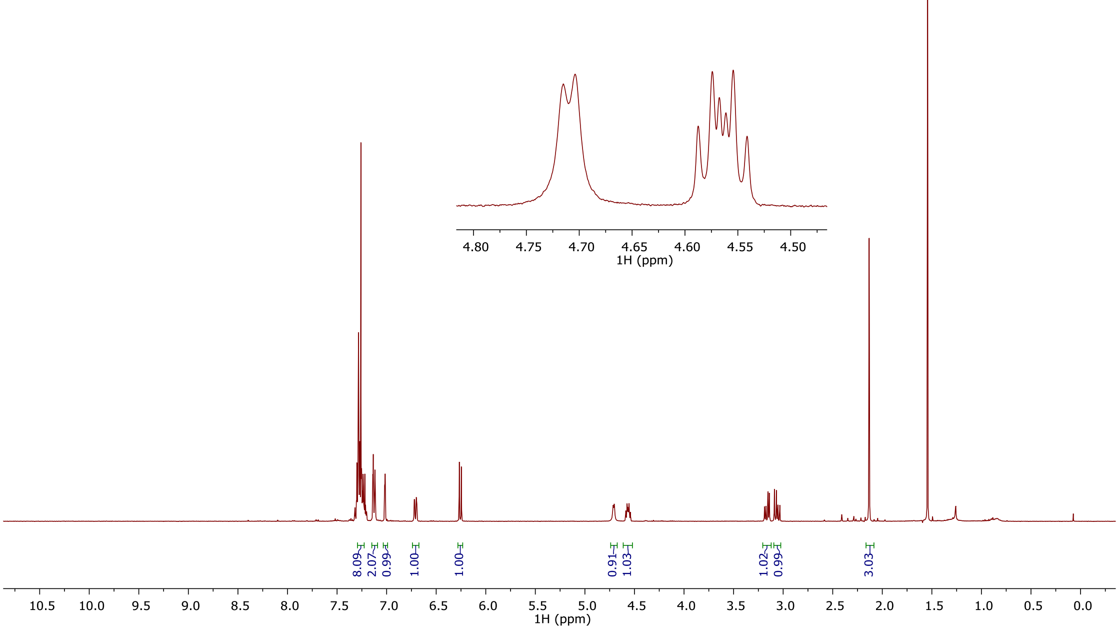


$^{13}$C NMR(101MHz,$CDCl_3$) **25**

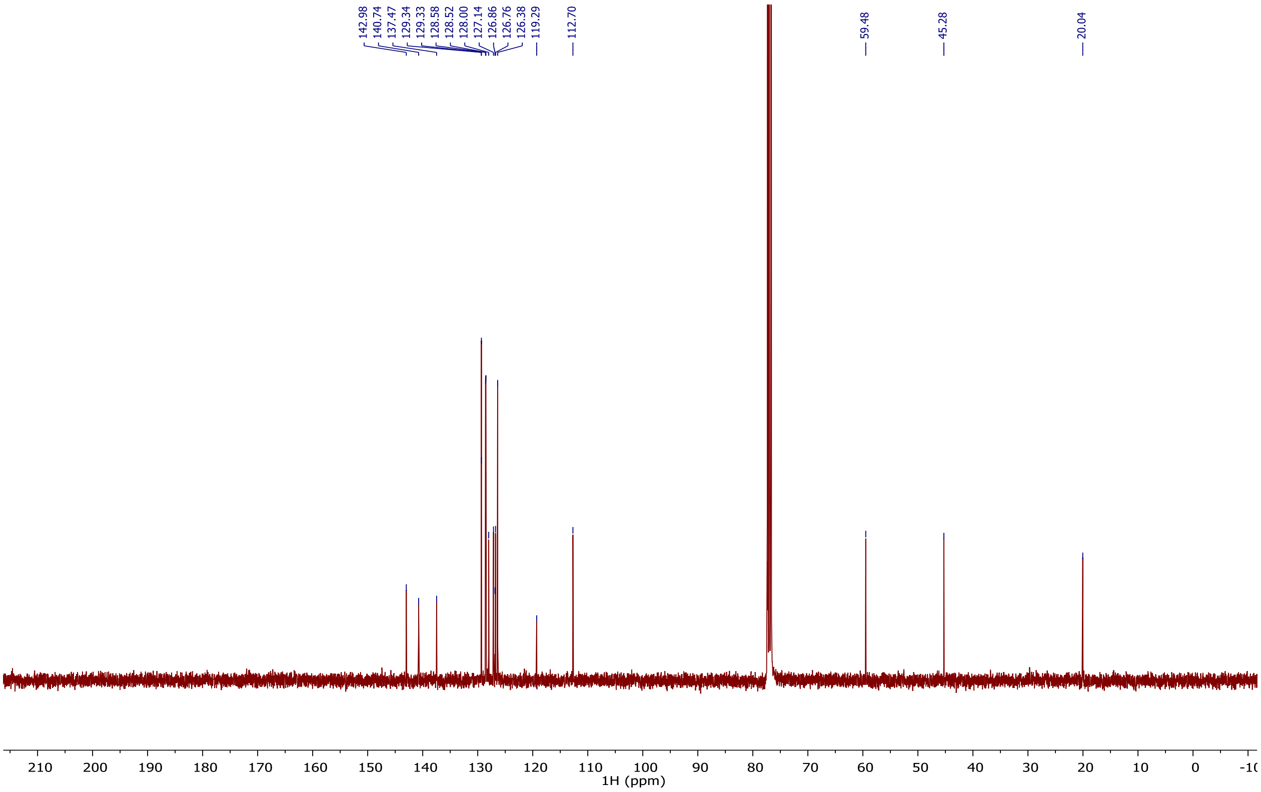

$^{1}H$ NMR (400 MHz, $CDCl_3$) **26**

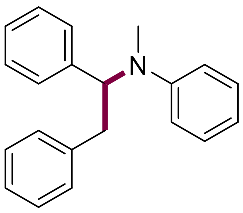

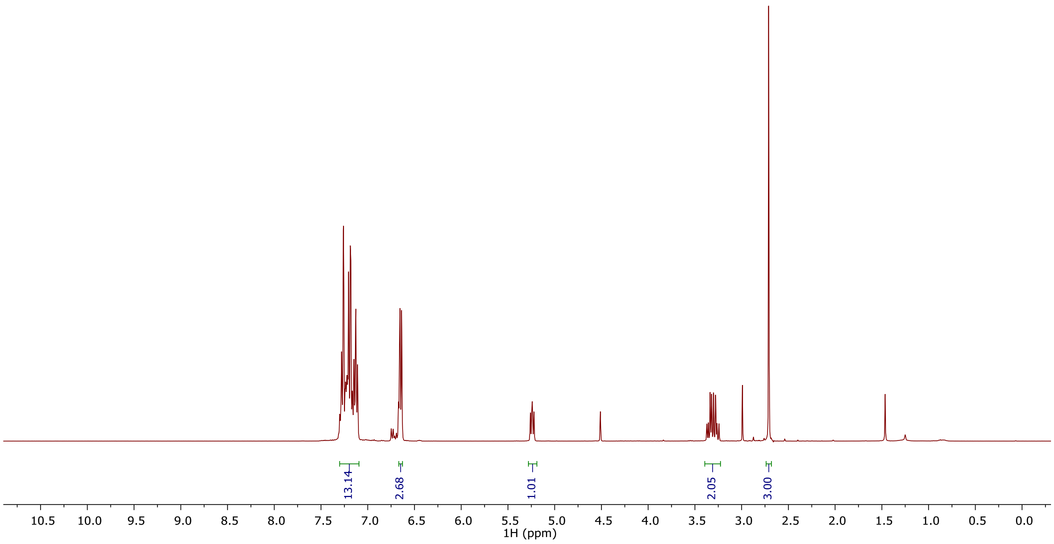


$^{13}C$ NMR(101MHz,$CDCl_3$) **26**

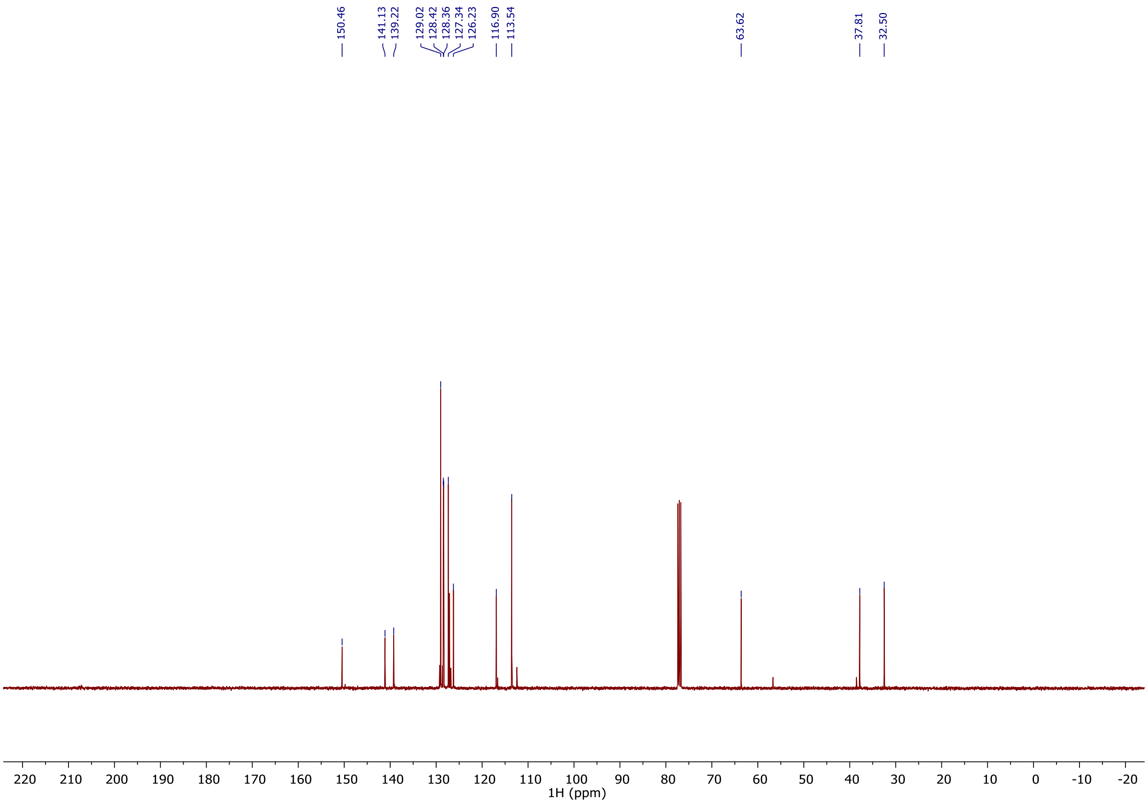

$^{1}$H NMR (400 MHz, $CDCl_3$) **27**

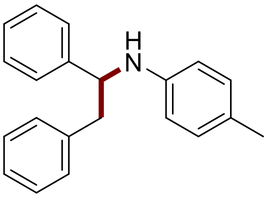

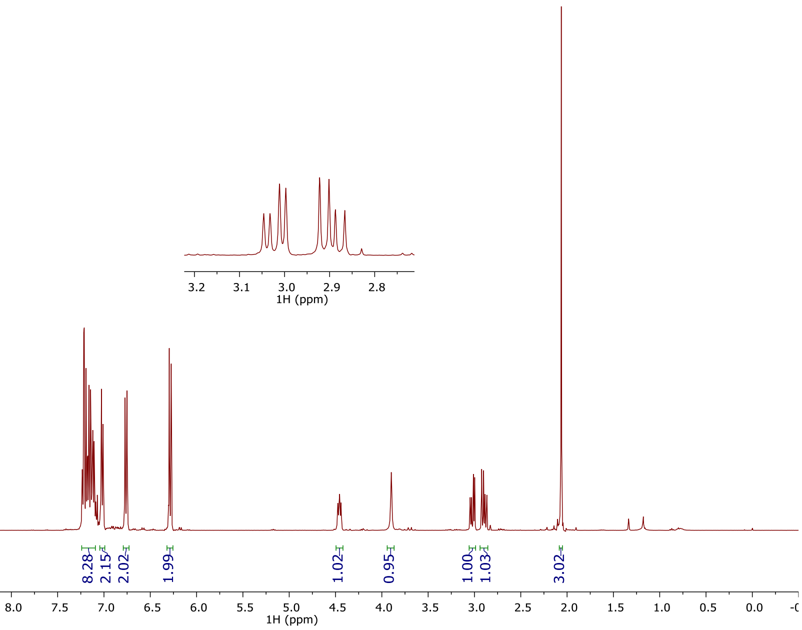


$^{13}$C NMR(101MHz,$CDCl_3$) **27**

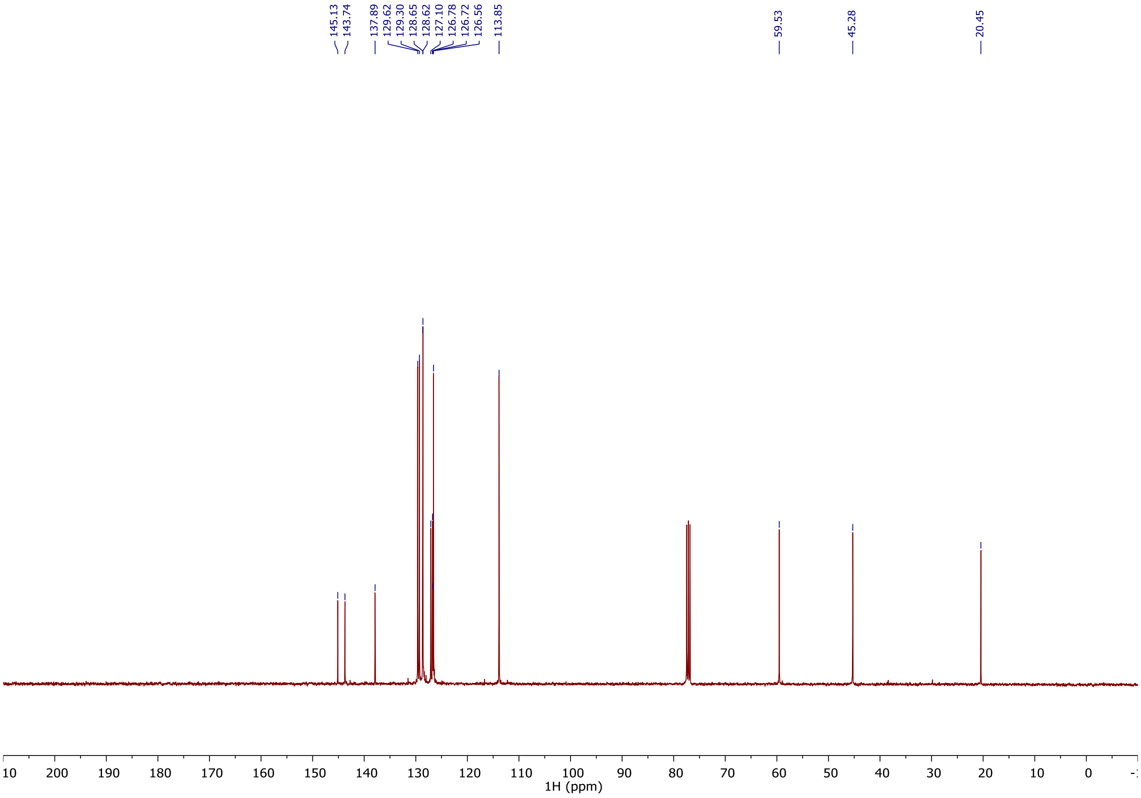

$^{1}$H NMR (400 MHz, $CDCl_3$) **28**

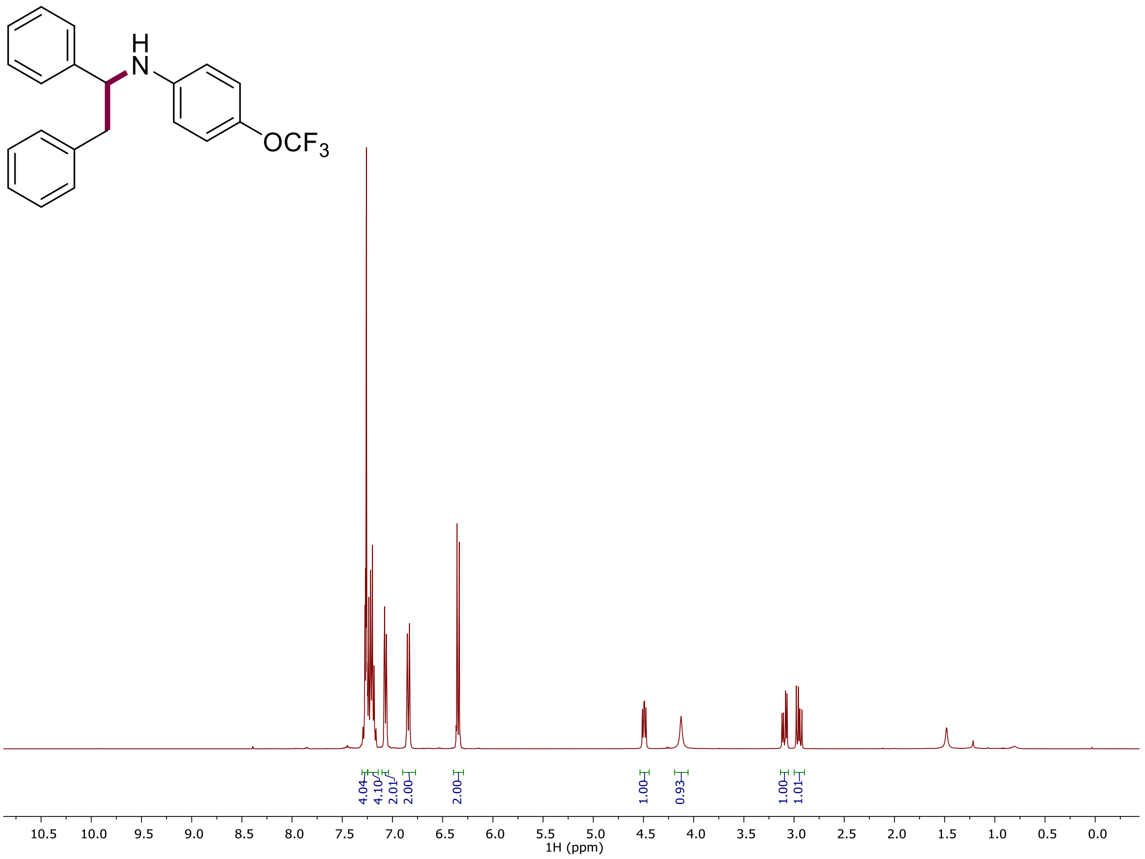


$^{13}$C NMR(101MHz,$CDCl_3$) **28**

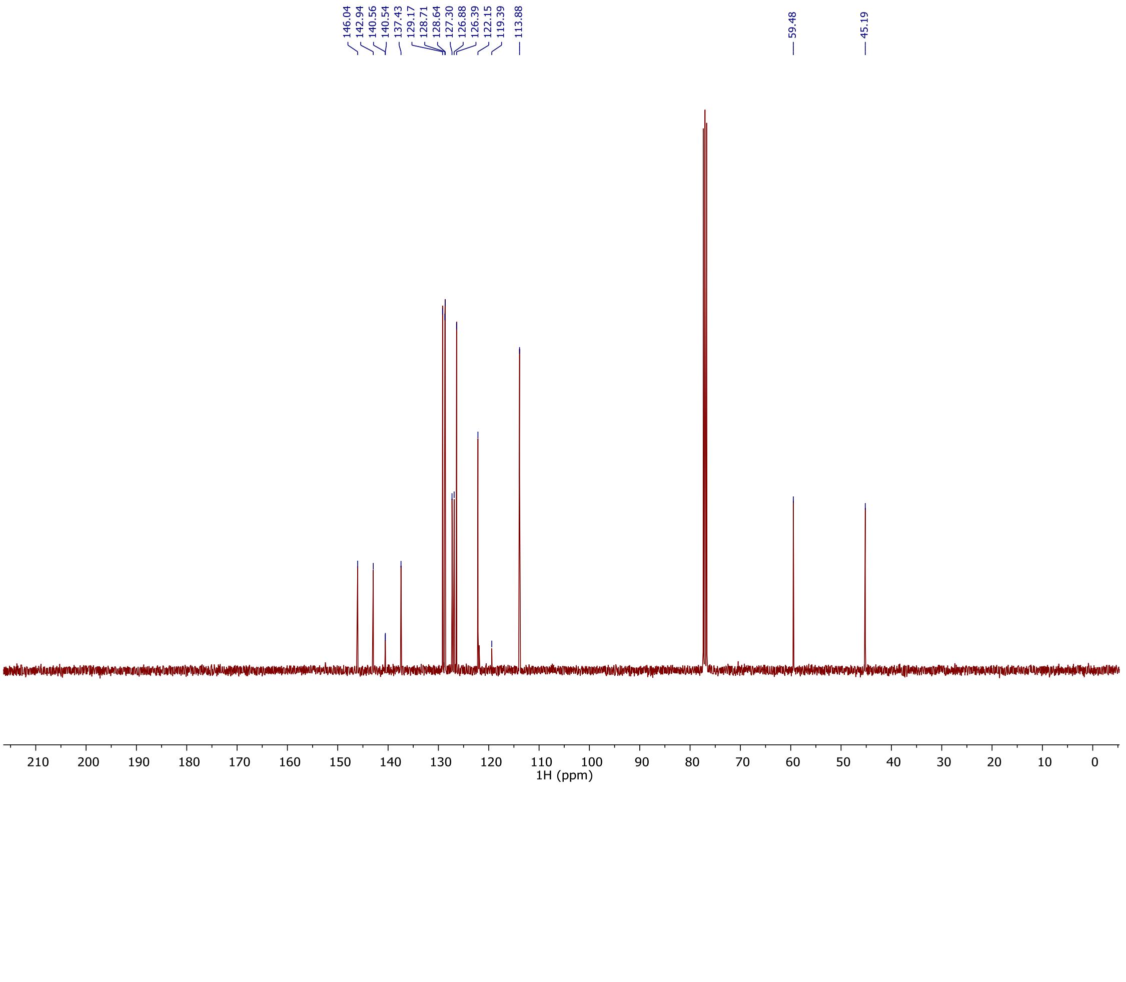

$^{19}$F NMR(377 MHz,CDCl$_3$) **28**

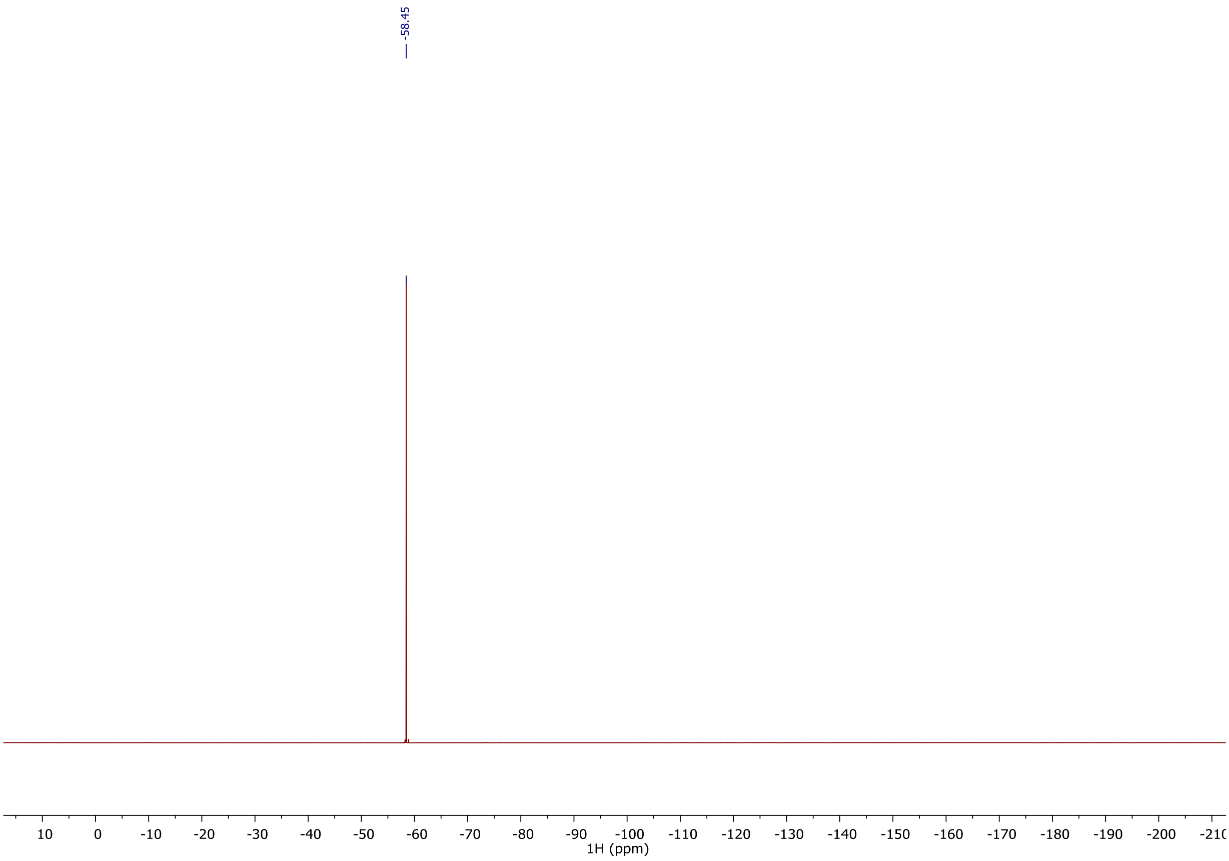

$^{1}$H NMR (400 MHz, $CDCl_3$) **29**

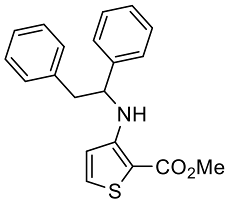


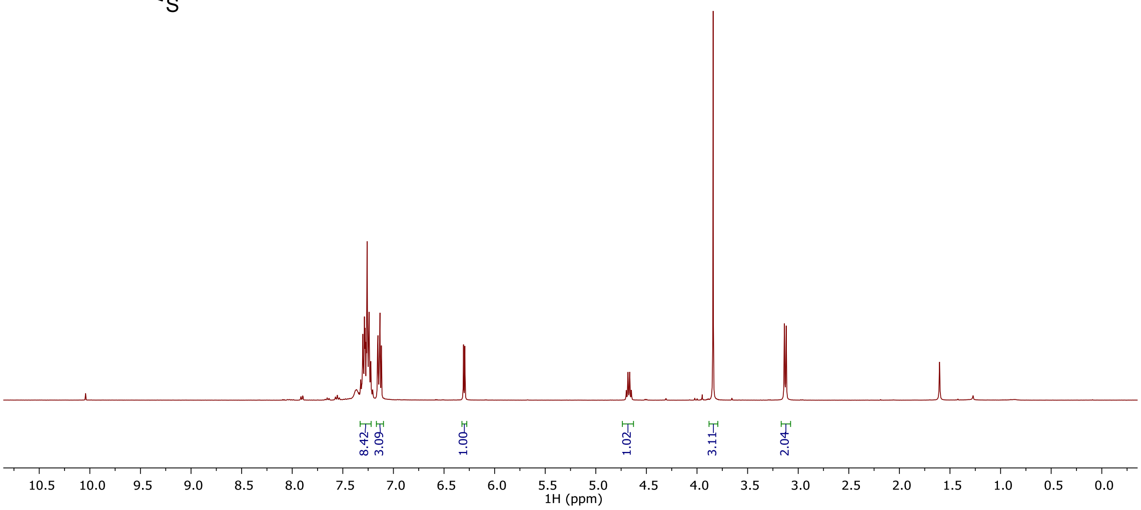


$^{13}$C NMR(101MHz,$CDCl_3$) **29**

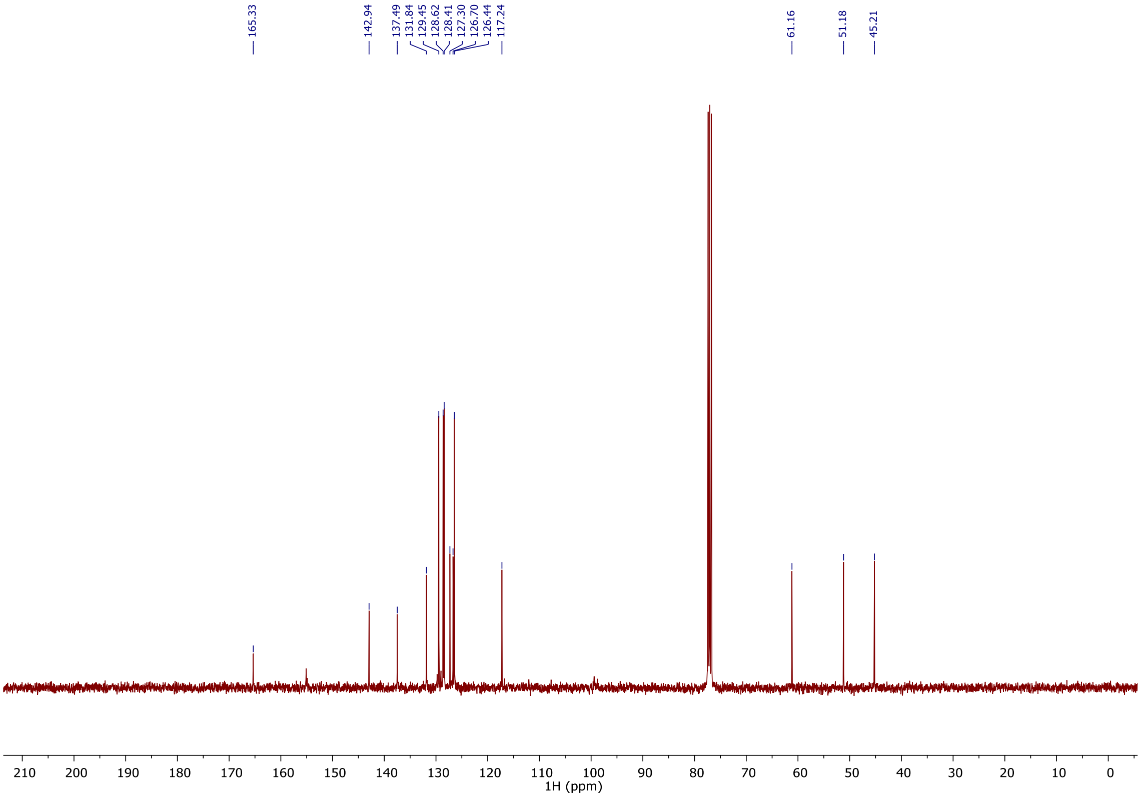

$^{1}H$ NMR (400 MHz, $CDCl_3$) **30**

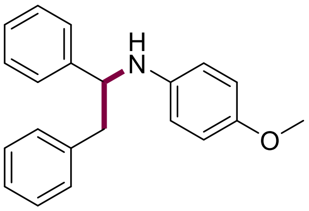


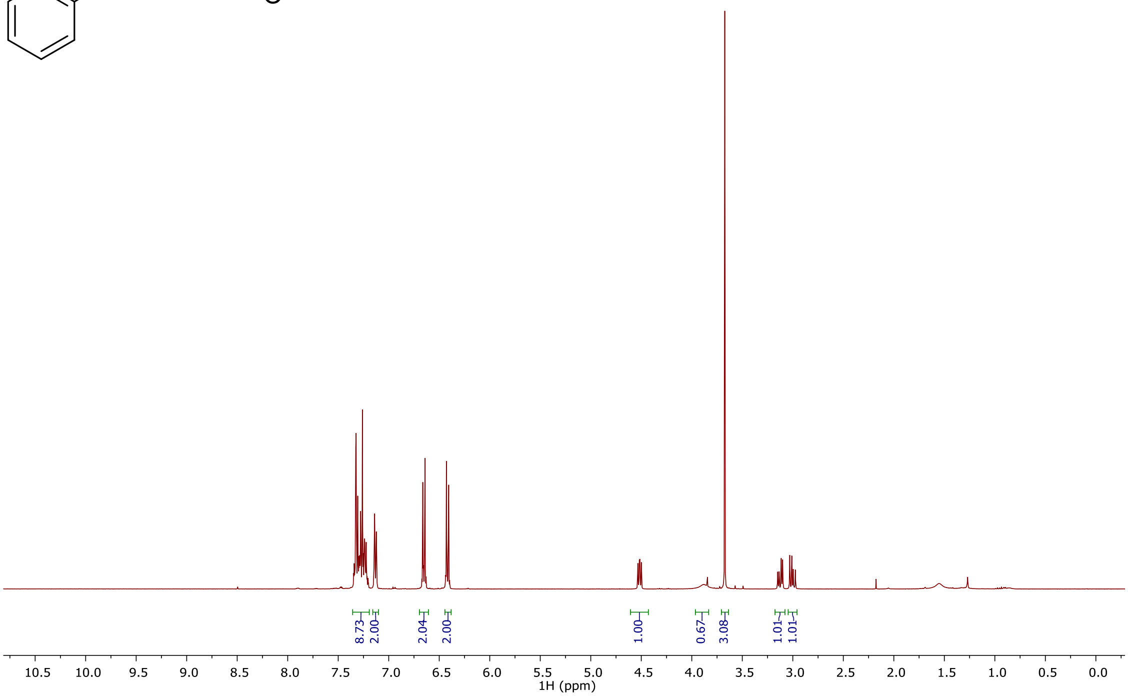


$^{13}C$ NMR(101MHz,$CDCl_3$) **30**

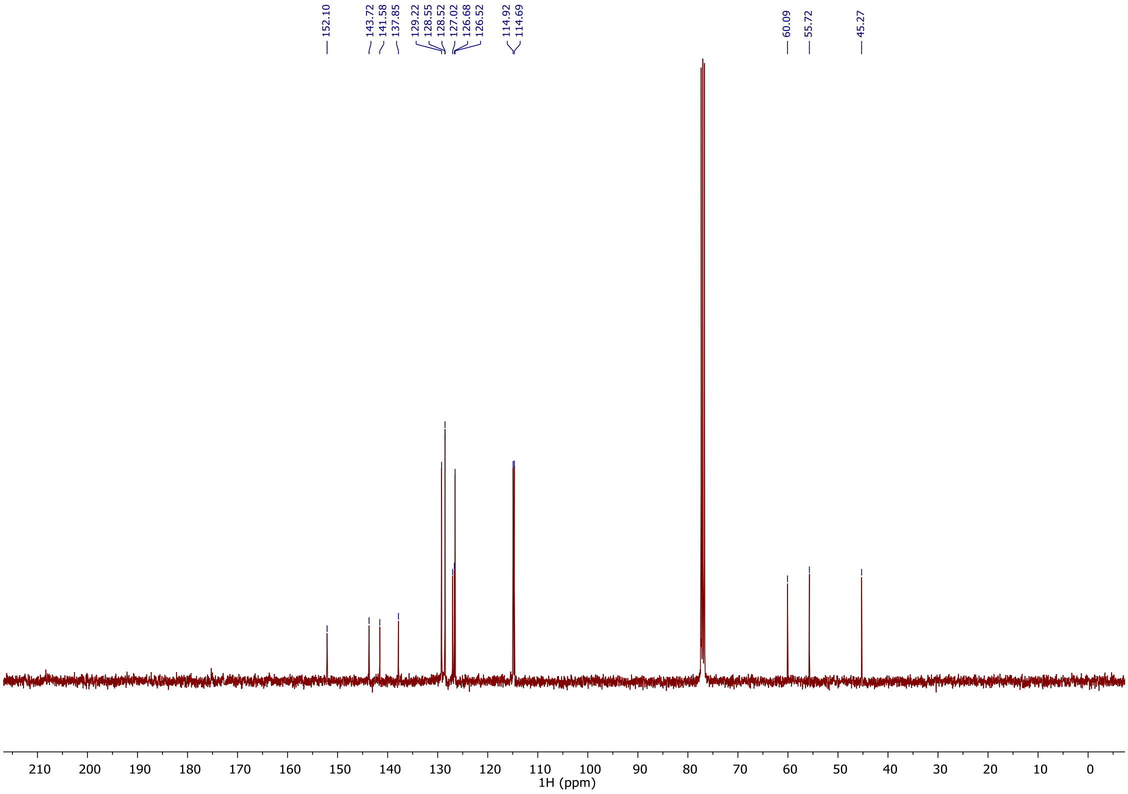

$^{1}$H NMR (400 MHz, $CDCl_3$) **31**

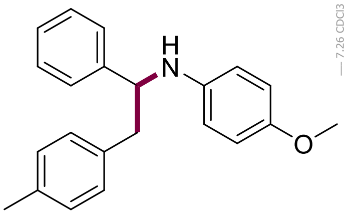

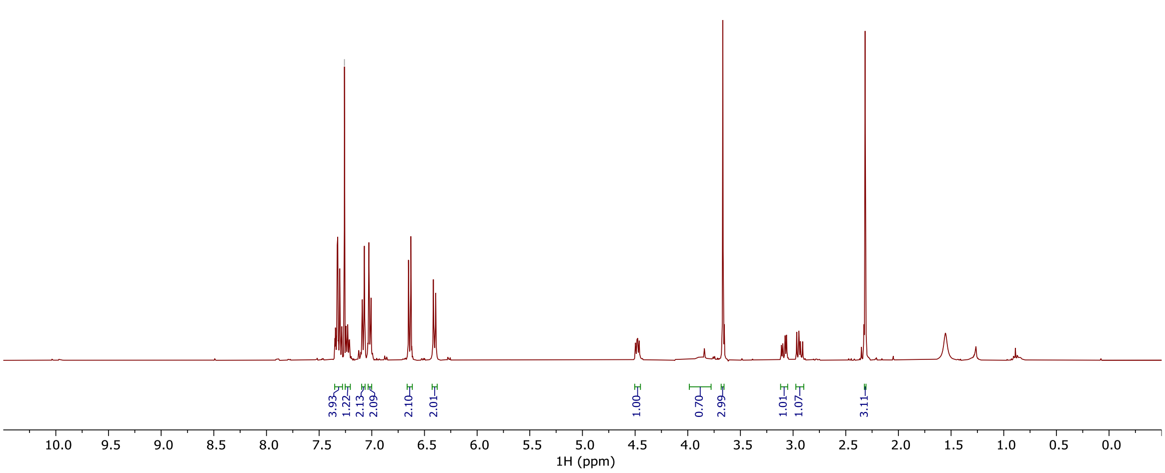


$^{13}$C NMR(101MHz,$CDCl_3$) **31**

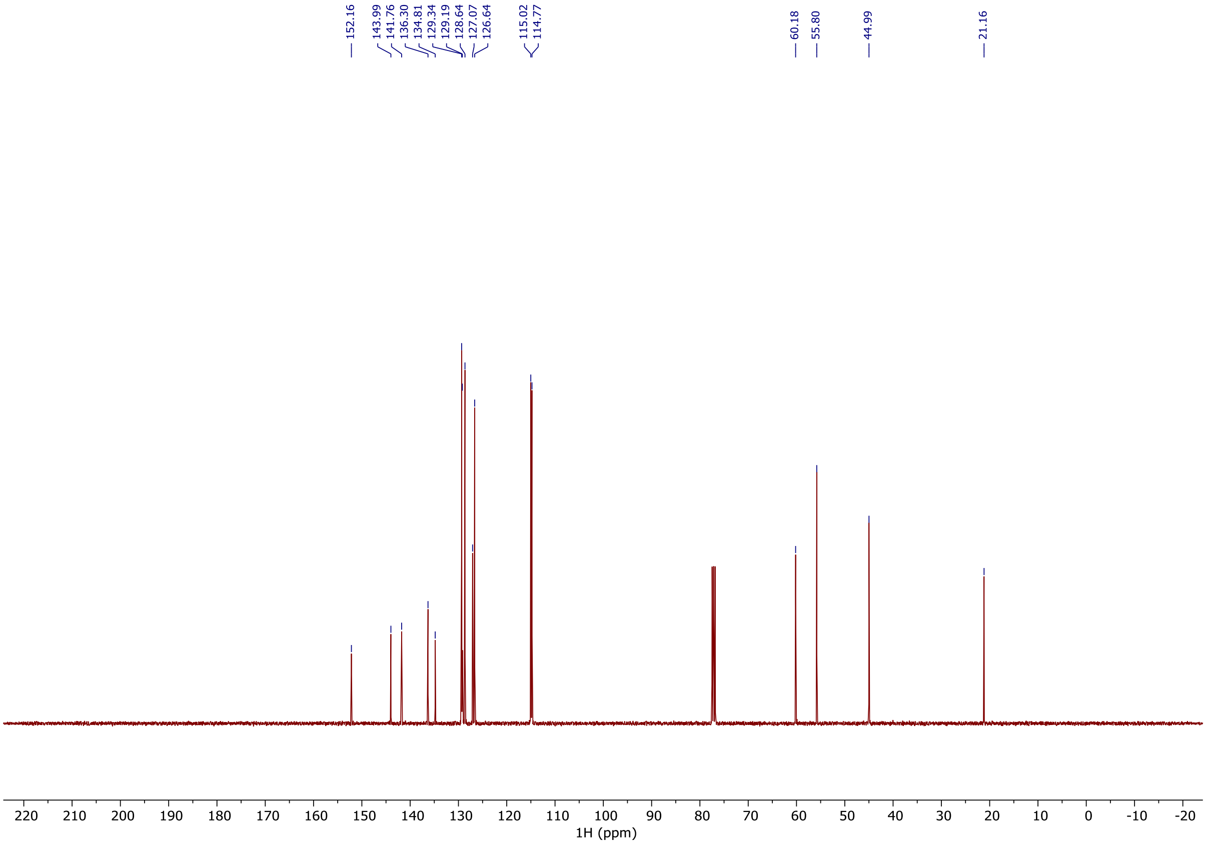

$^{1}$H NMR (400 MHz, $CDCl_3$) **32**

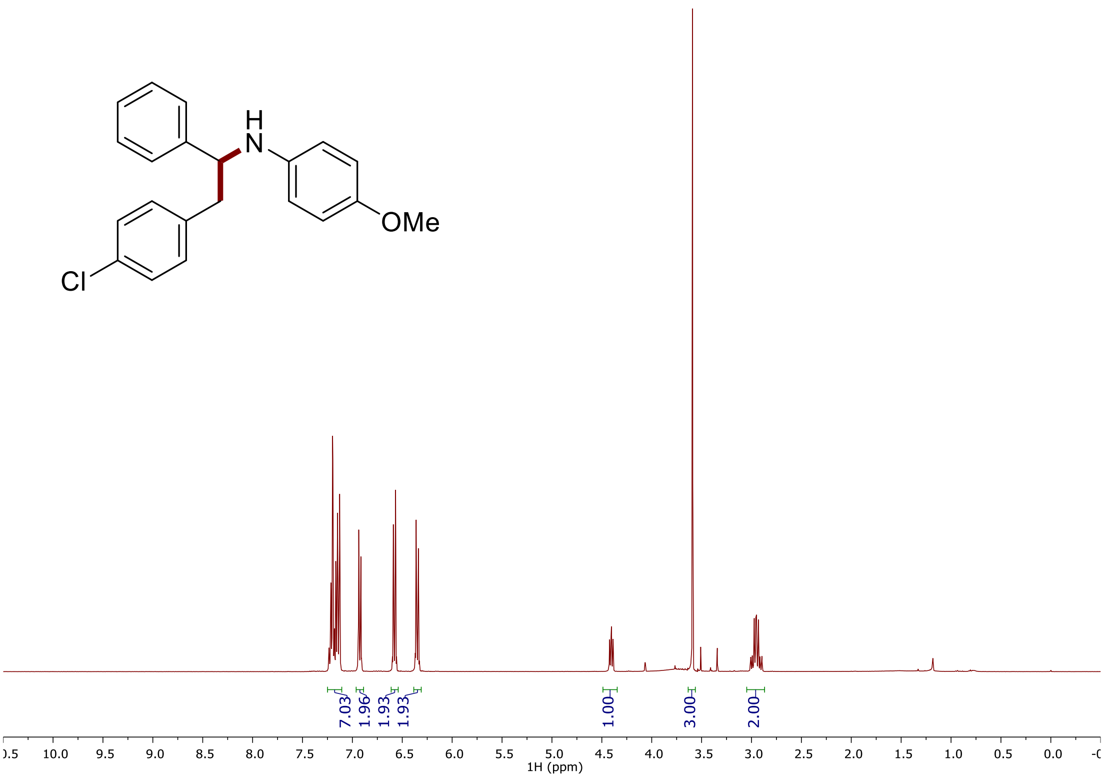


$^{13}$C NMR(101MHz,$CDCl_3$) **32**

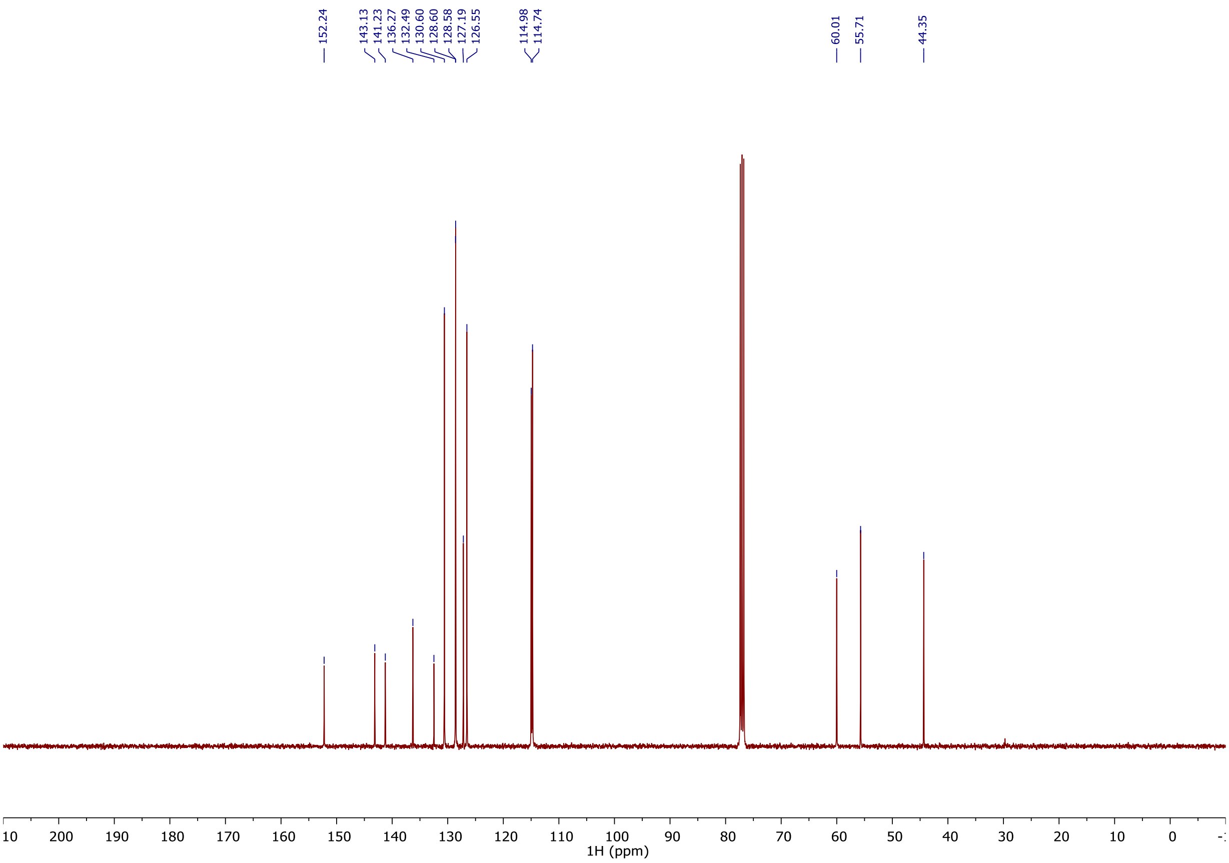

$^{1}H$ NMR (400 MHz, $CDCl_3$) **33**

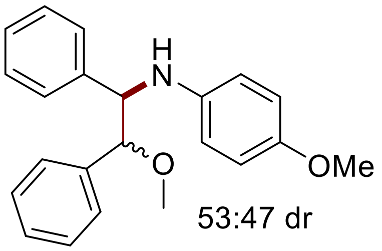


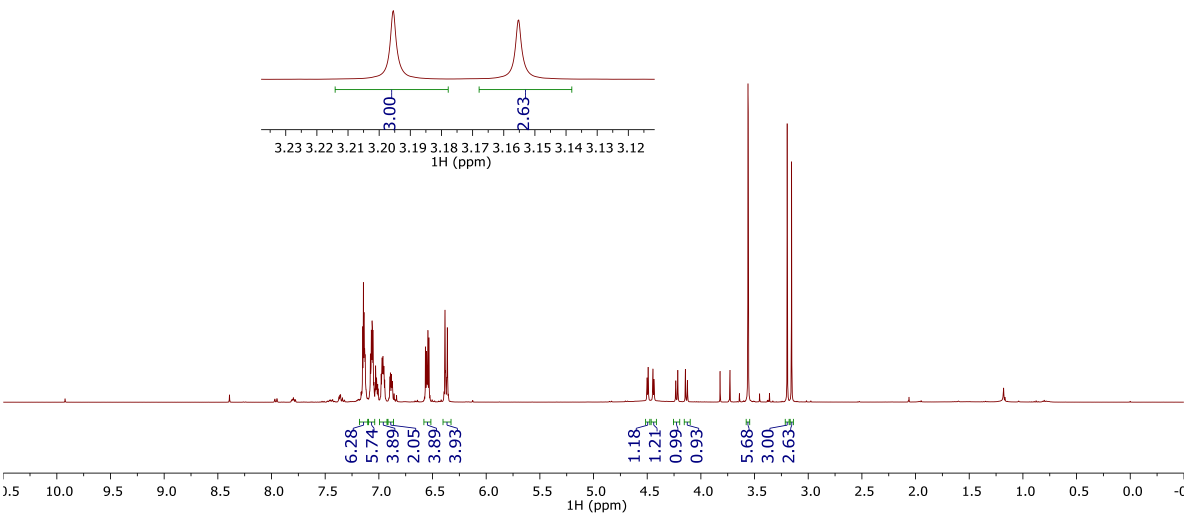

$^{13}C$ NMR(101MHz,$CDCl_3$) **33**

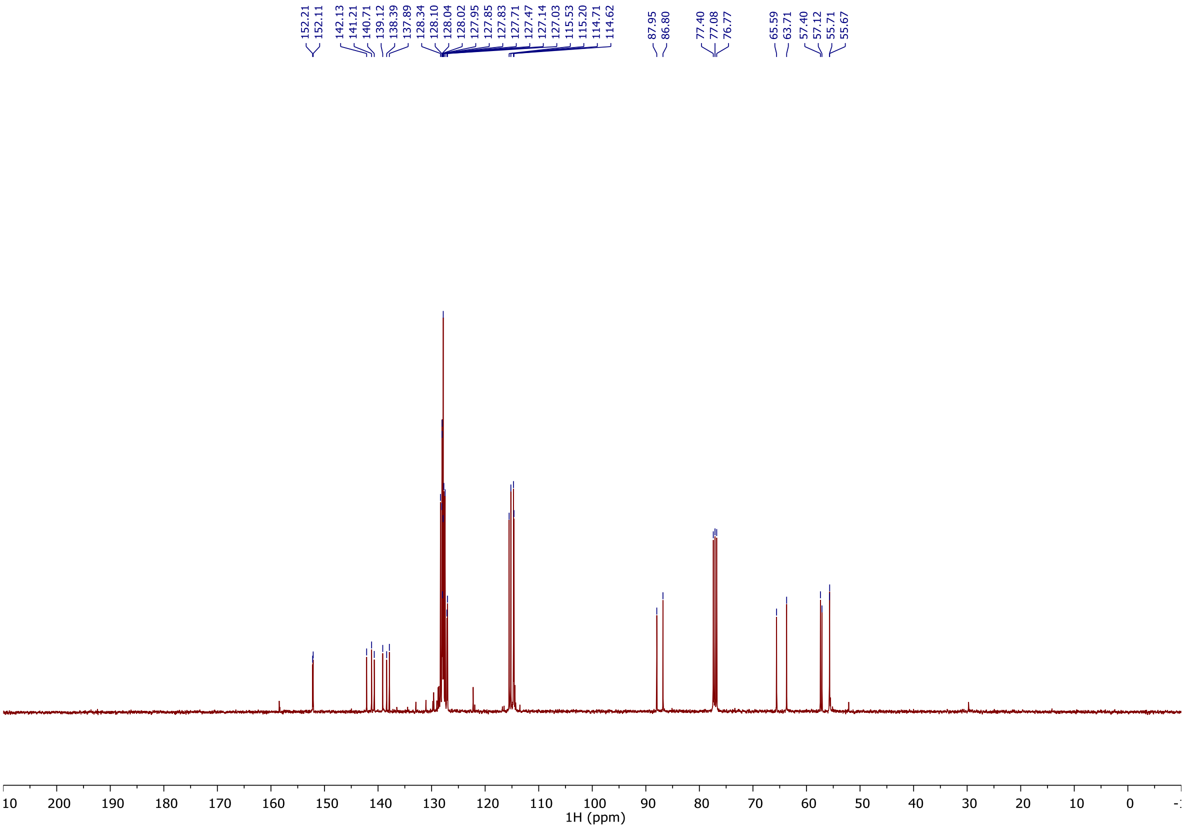

$^{1}$H NMR (400 MHz, $CDCl_3$) **34**

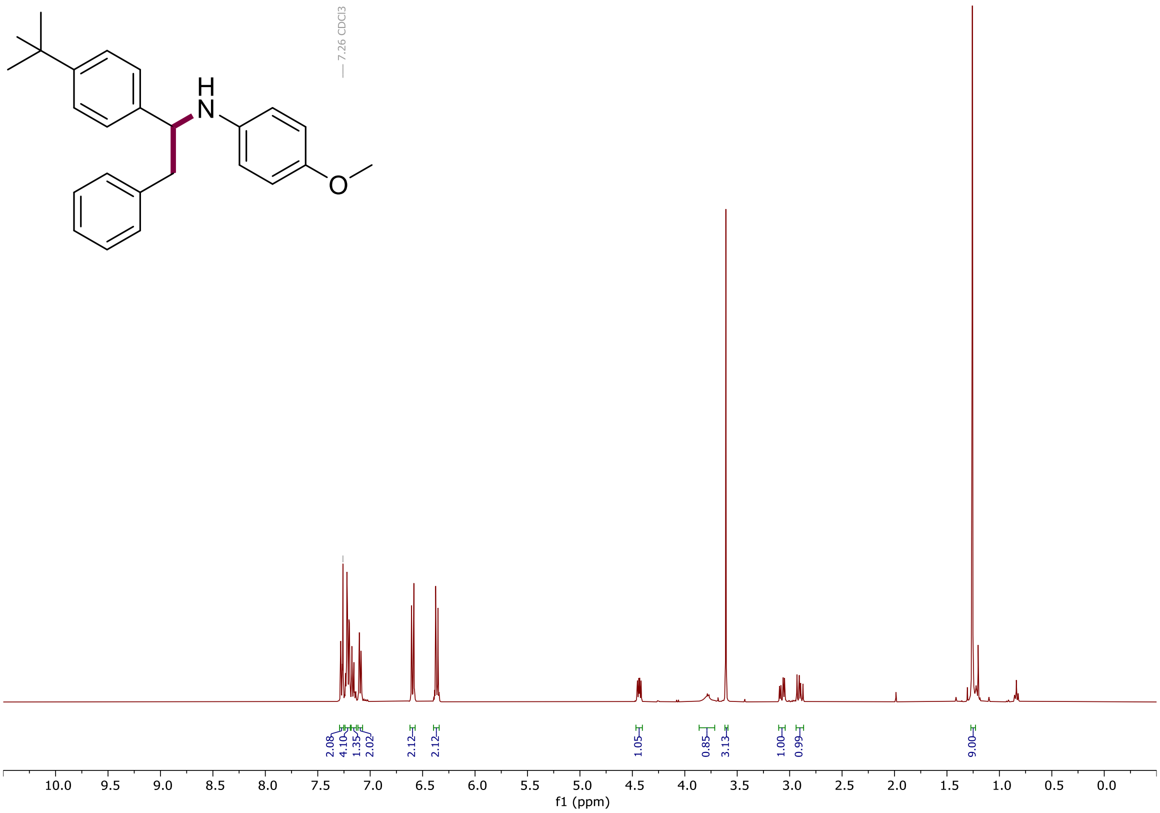


$^{13}$C NMR(101MHz,$CDCl_3$) **34**

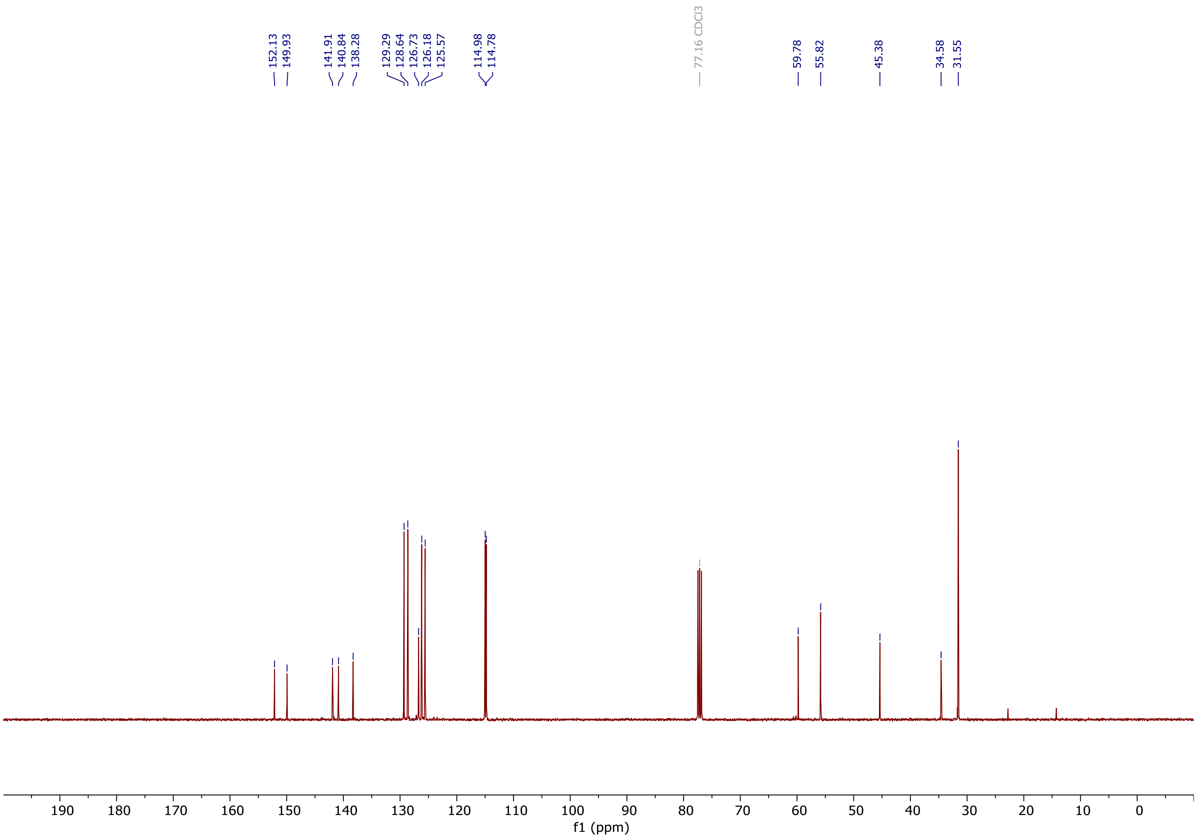

$^{1}H$ NMR (400 MHz, $CDCl_3$) **35**

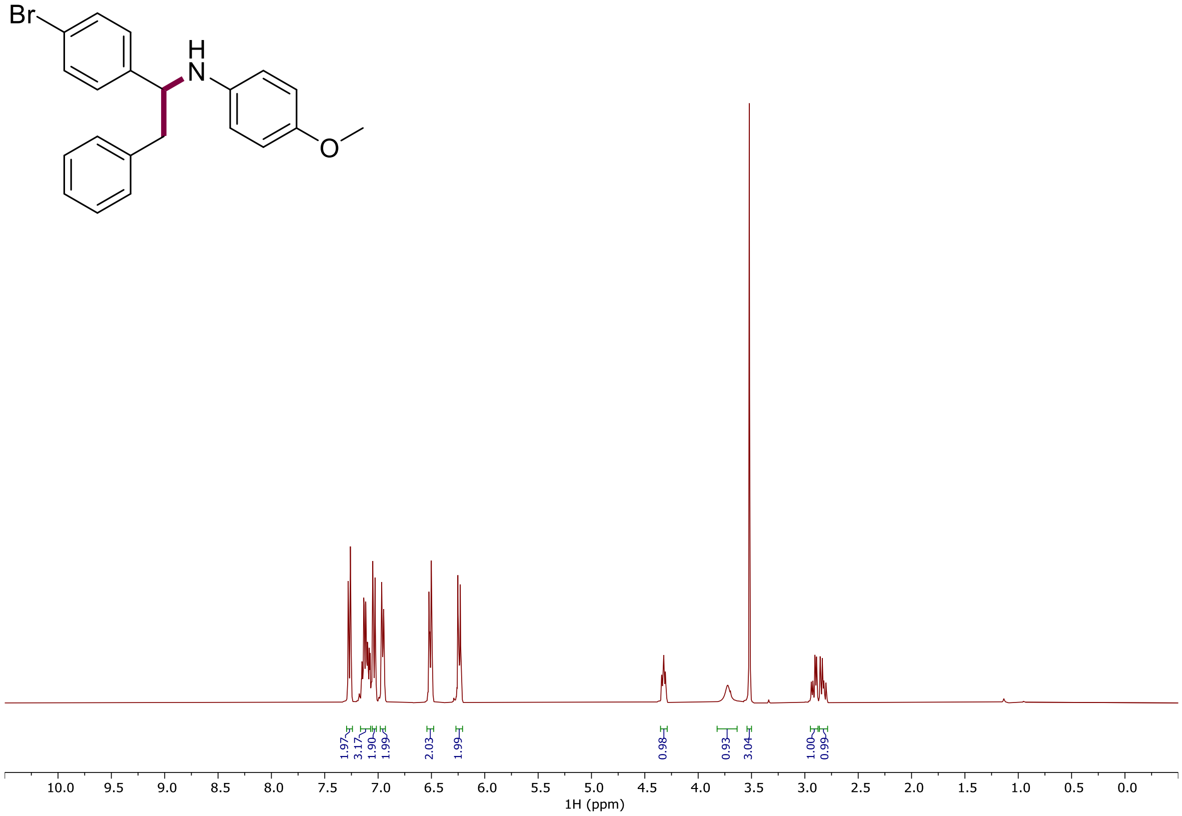

$^{13}C$ NMR(101MHz,$CDCl_3$) **35**

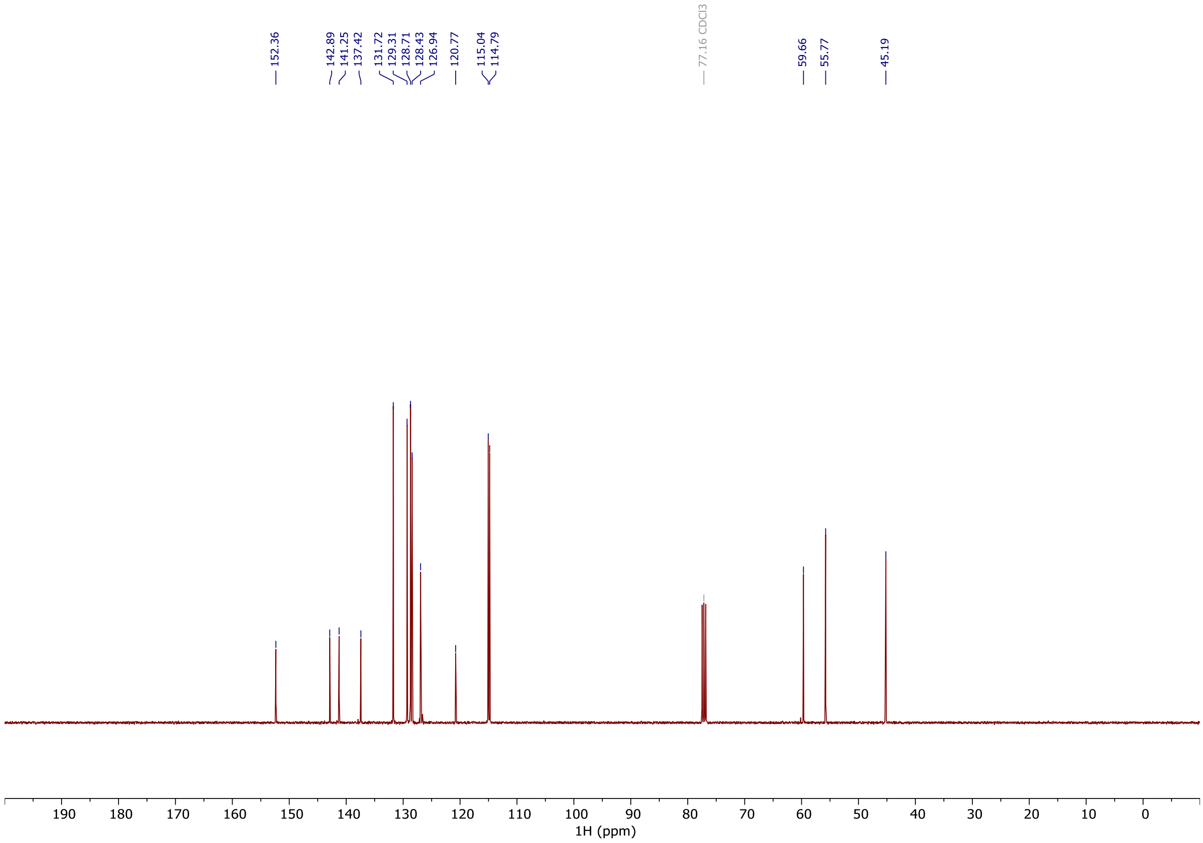

$^{1}H$ NMR (400 MHz, $CDCl_3$) **36**

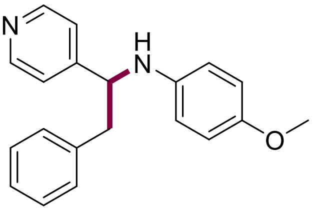


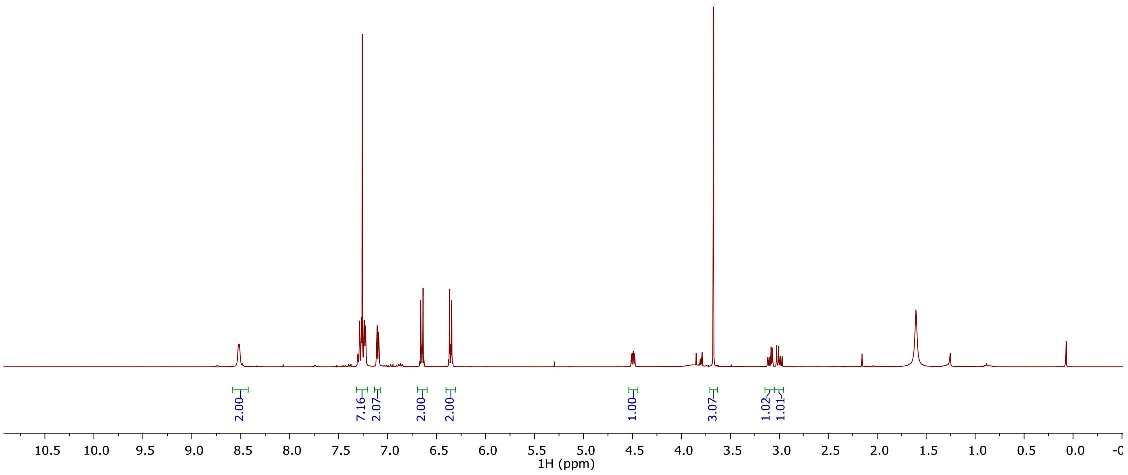


$^{13}C$ NMR(101MHz,$CDCl_3$) **36**

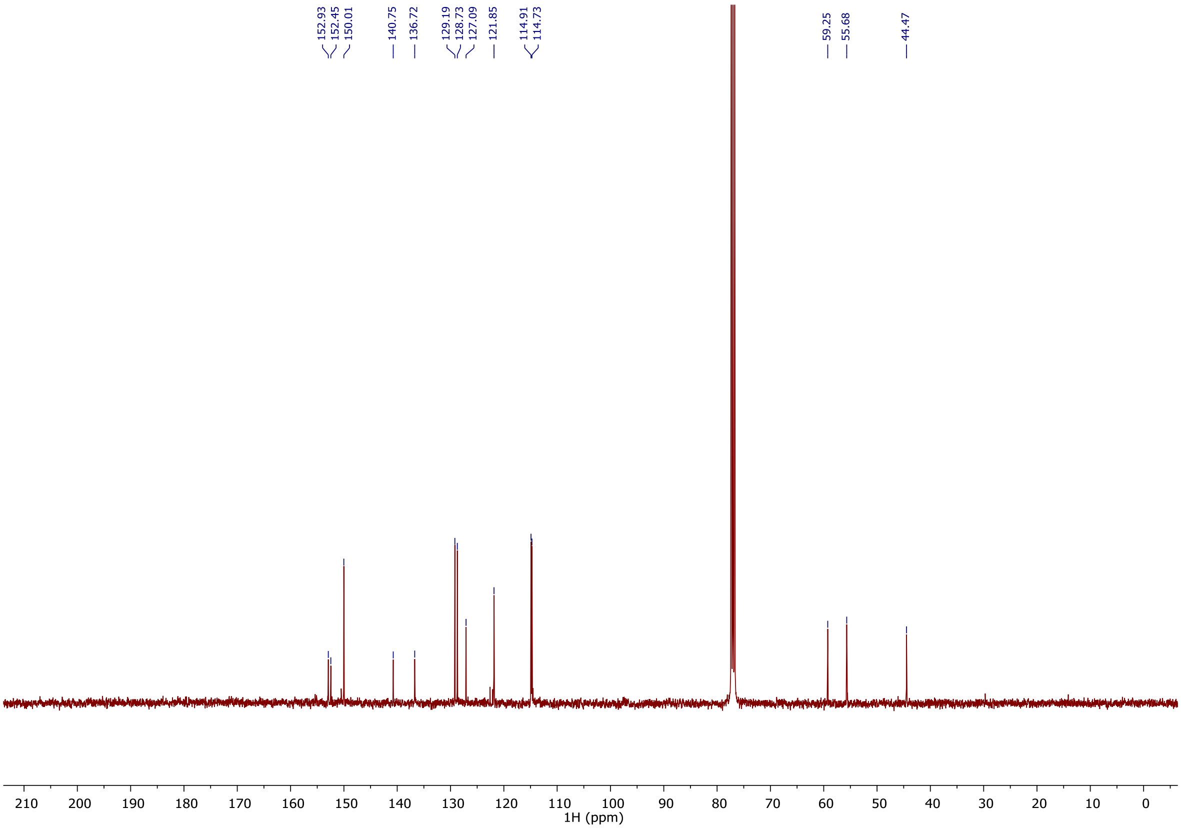

$^{1}$H NMR (400 MHz, $CDCl_3$) **37**

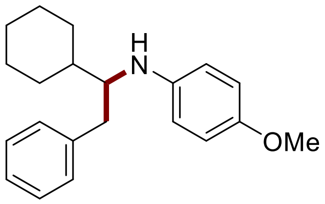


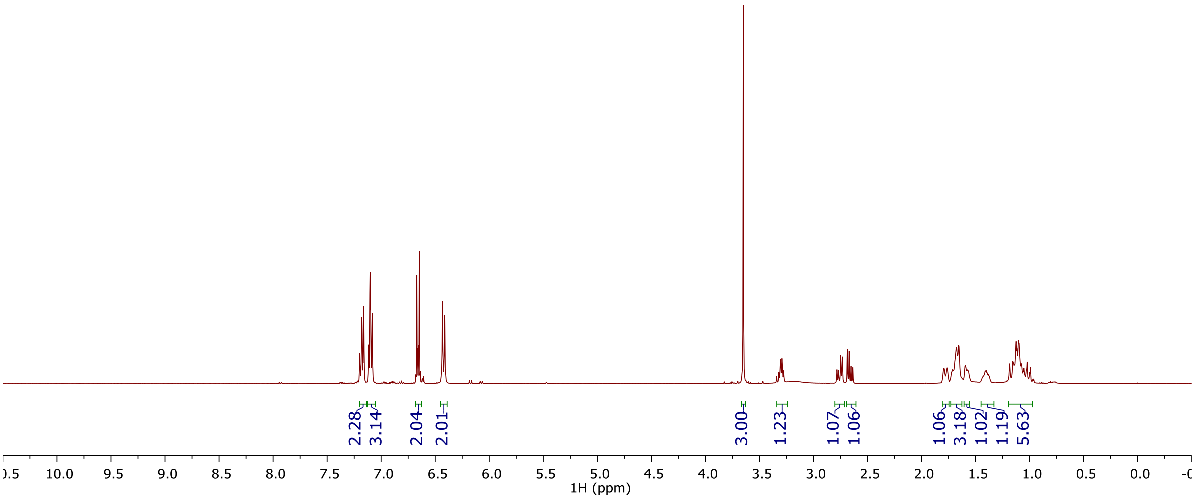


$^{13}$C NMR(101MHz,$CDCl_3$) **37**

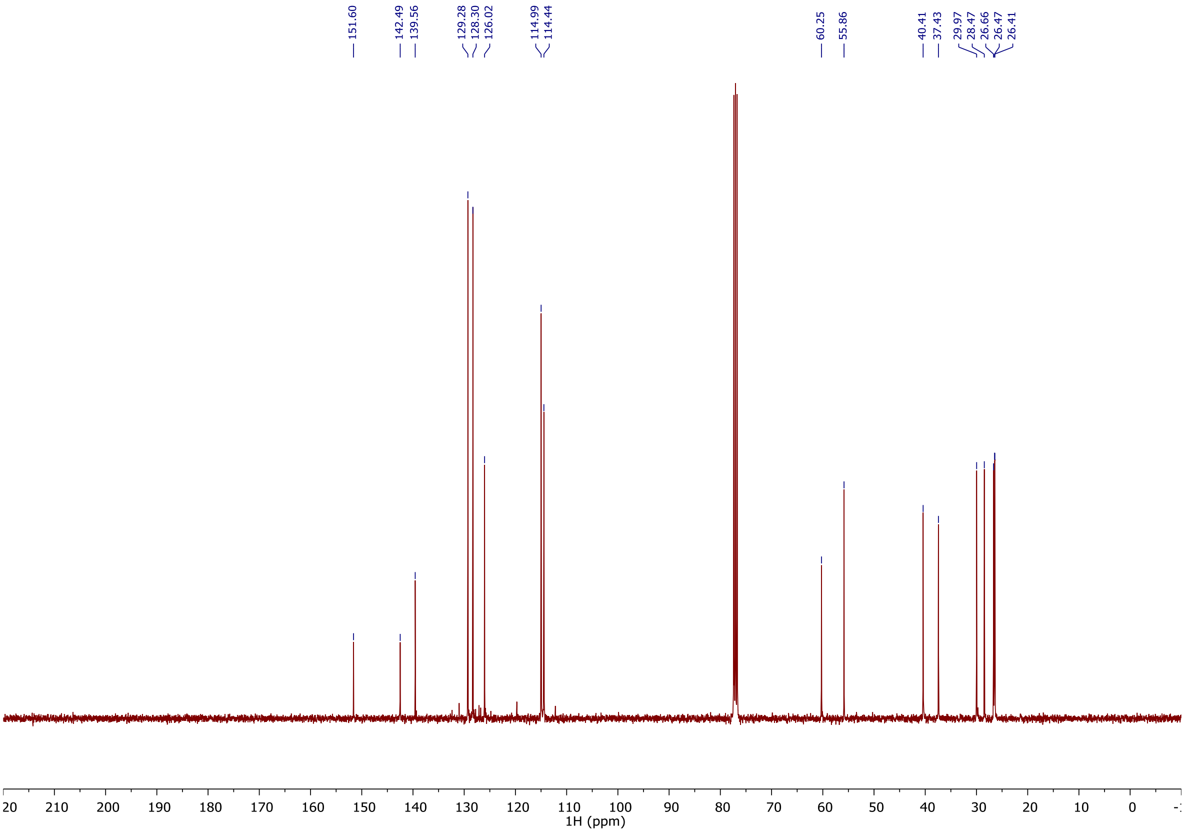

$^{1}$H NMR (400 MHz, $CDCl_3$) **38**

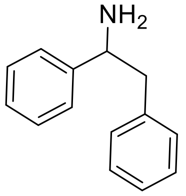


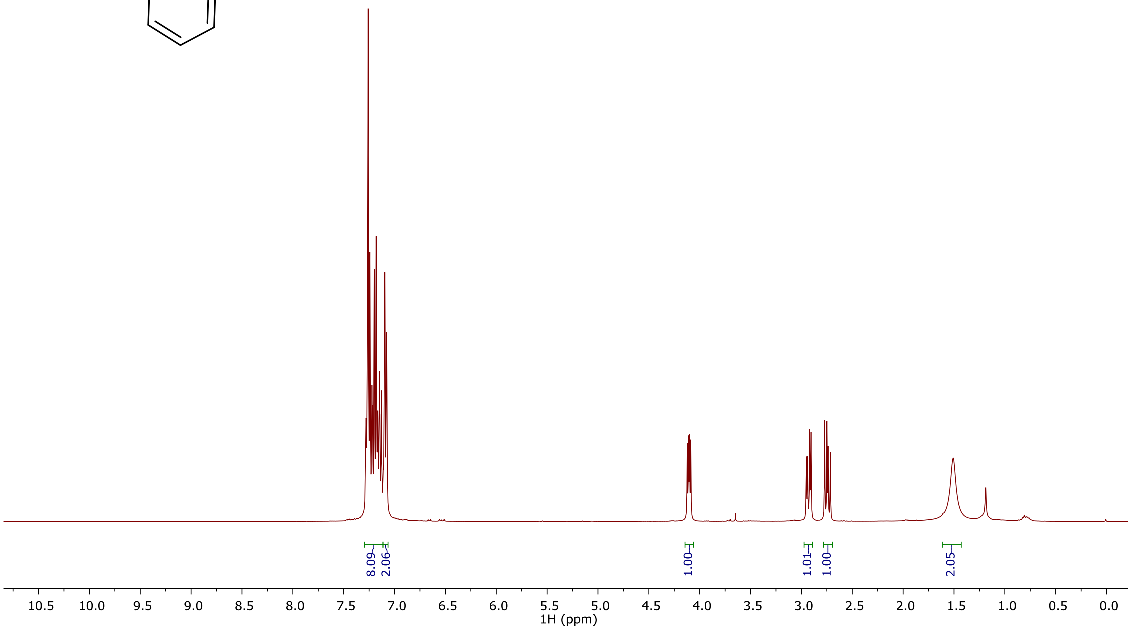


$^{13}$C NMR(101MHz,$CDCl_3$) **38**

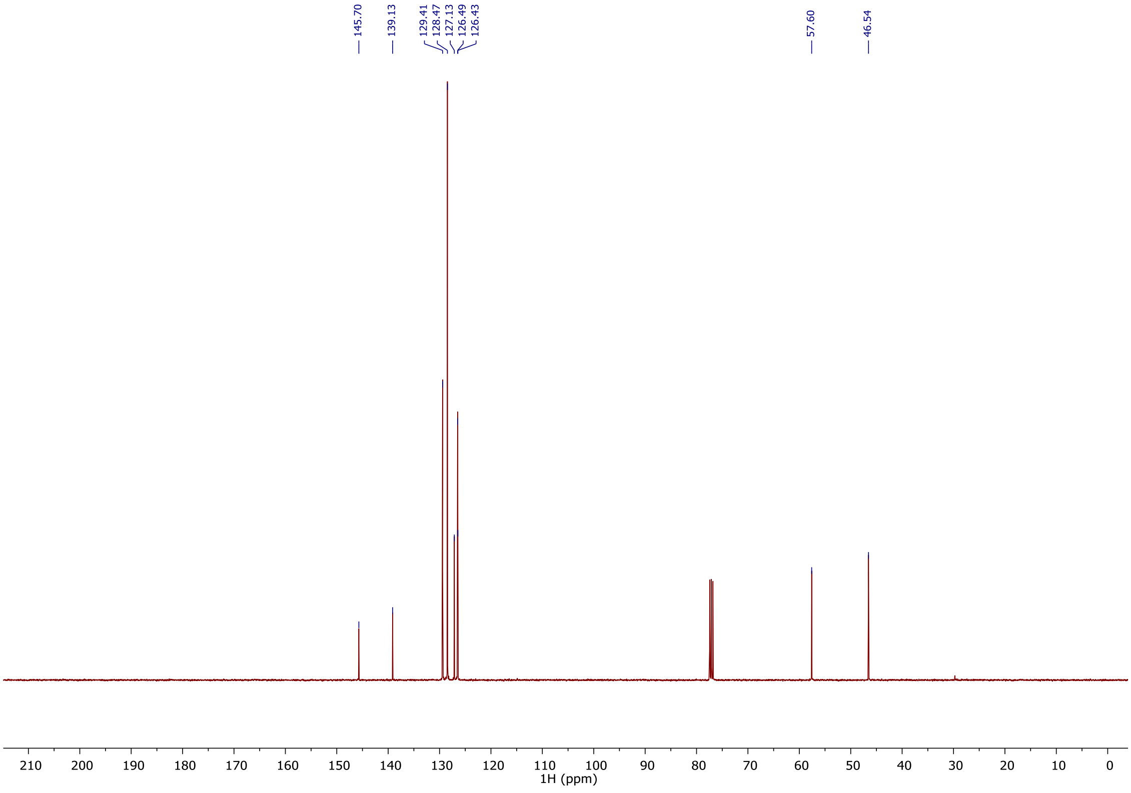

$^{1}$H NMR (400 MHz, $CDCl_3$) **39**

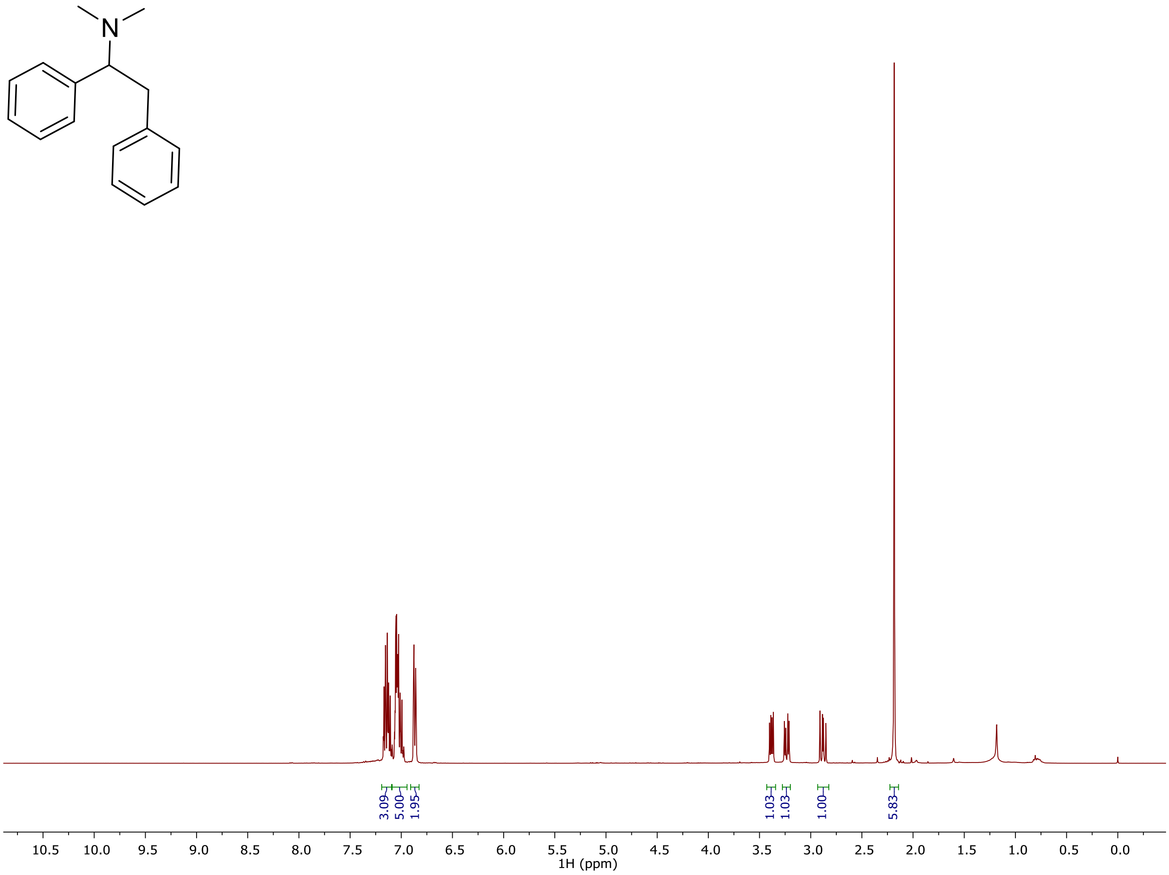


$^{13}$C NMR(101MHz,$CDCl_3$) **39**

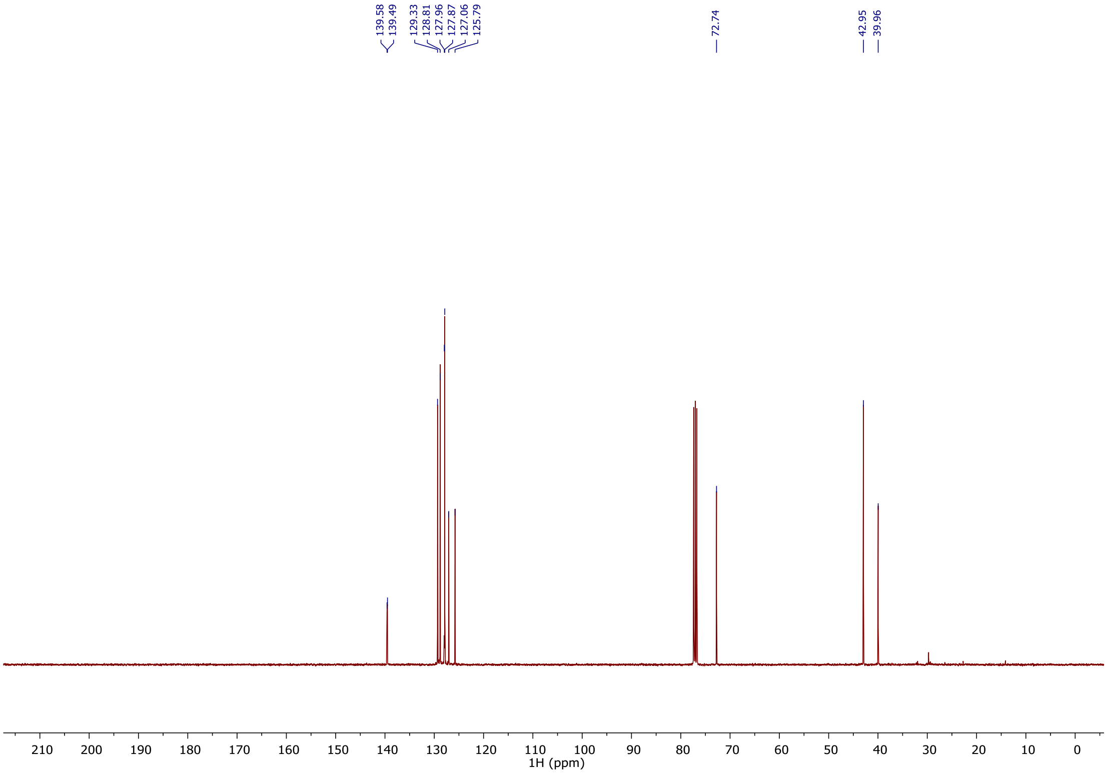

$^{1}$H NMR (400 MHz, $CDCl_3$) **40**

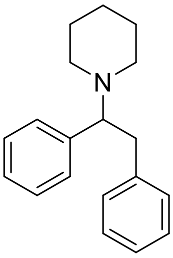


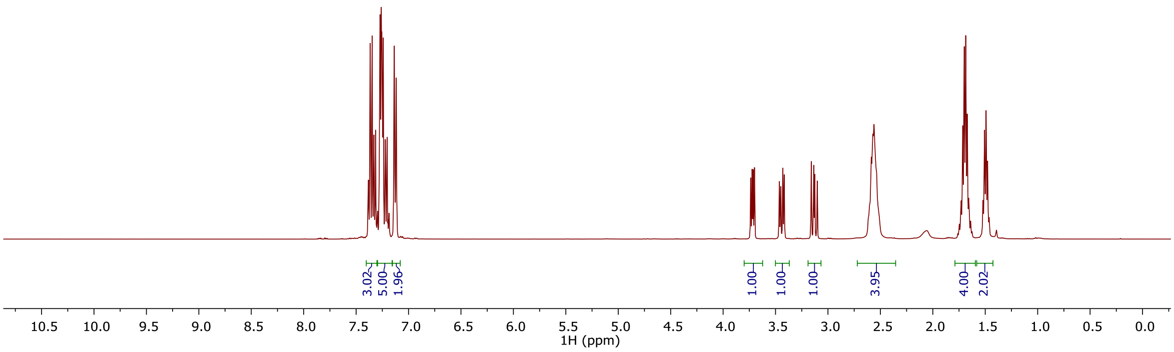


$^{13}$C NMR(101MHz,$CDl_3$) **40**

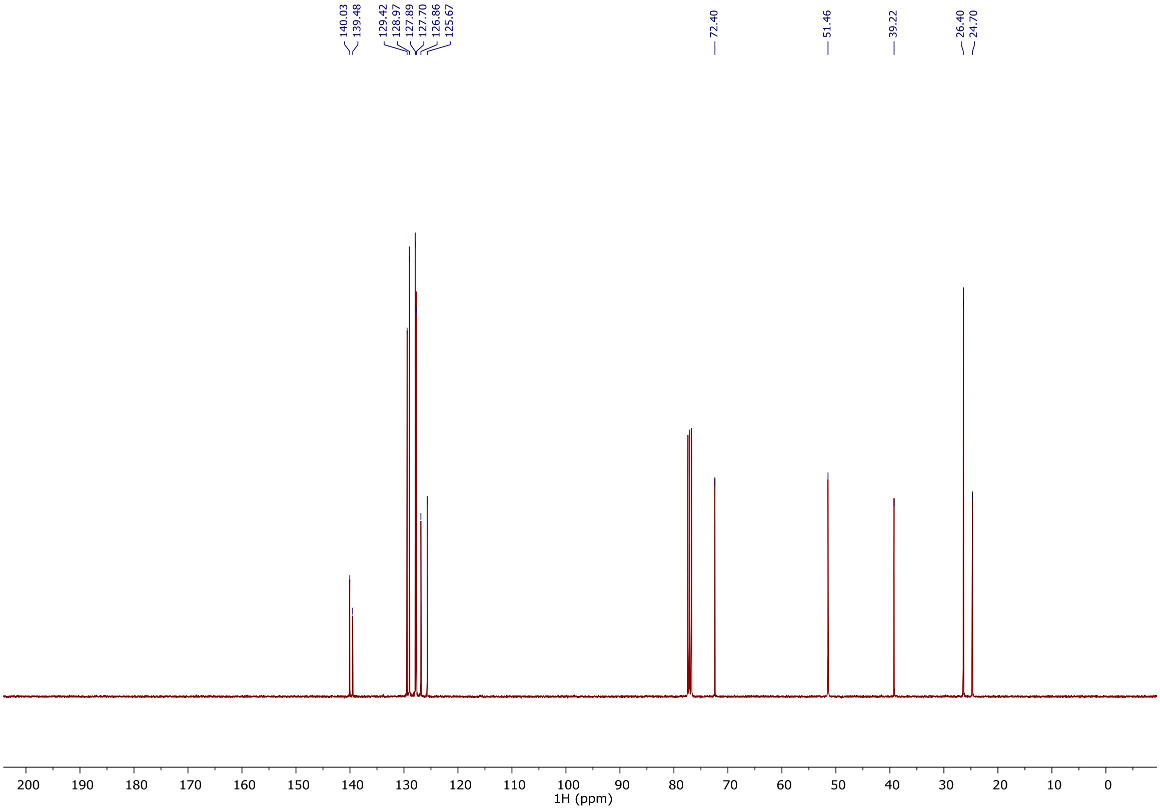

$^{1}$H NMR (400 MHz, $CDCl_3$) **41**

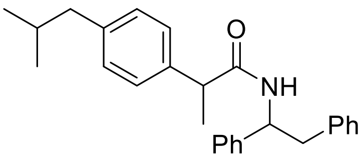


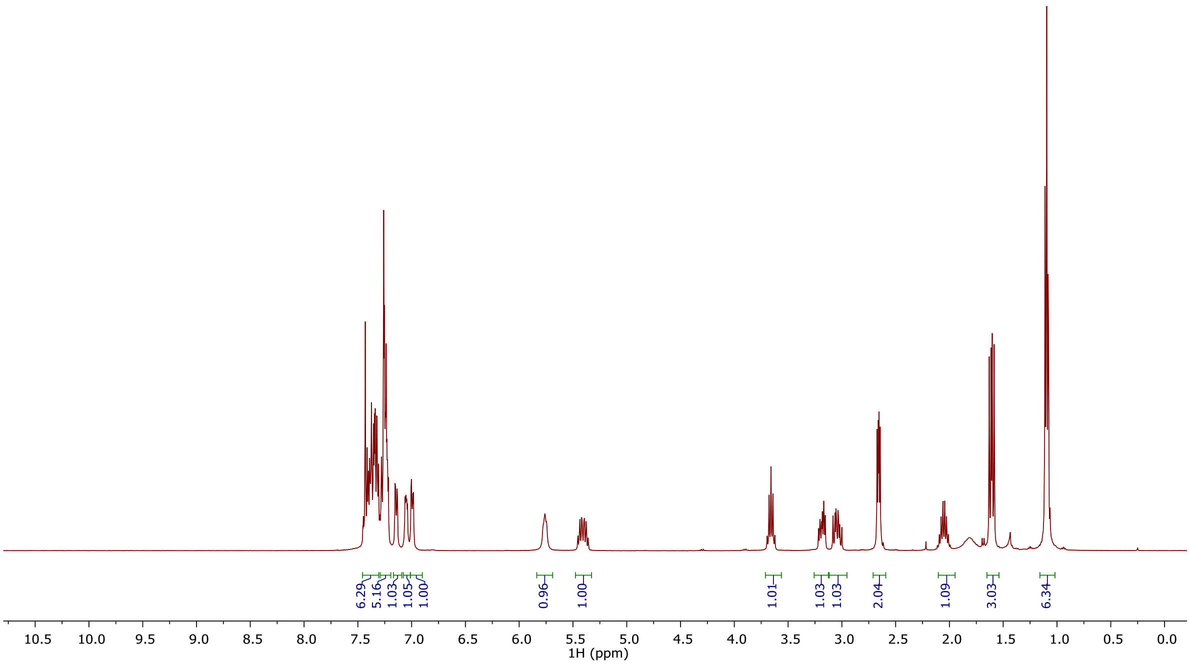


$^{13}$C NMR(101MHz,$CDl_3$) **41**

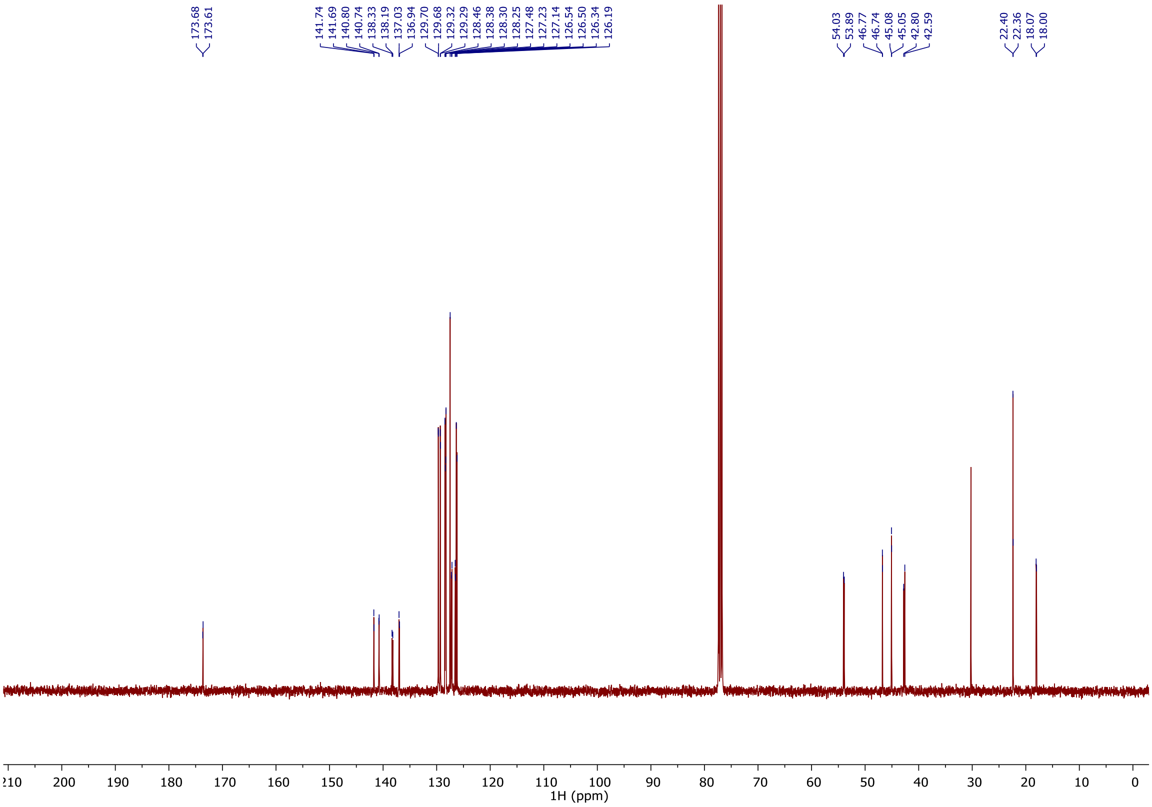

$^{1}H$ NMR (400 MHz, $CDCl_3$) **42**

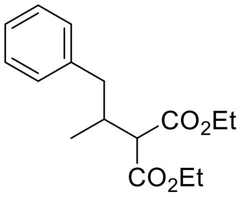


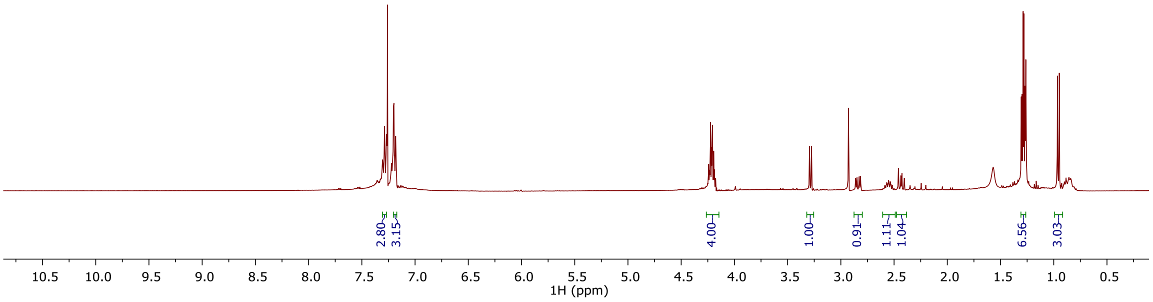


$^{13}C$ NMR(101MHz,$CDCl_3$) **42**

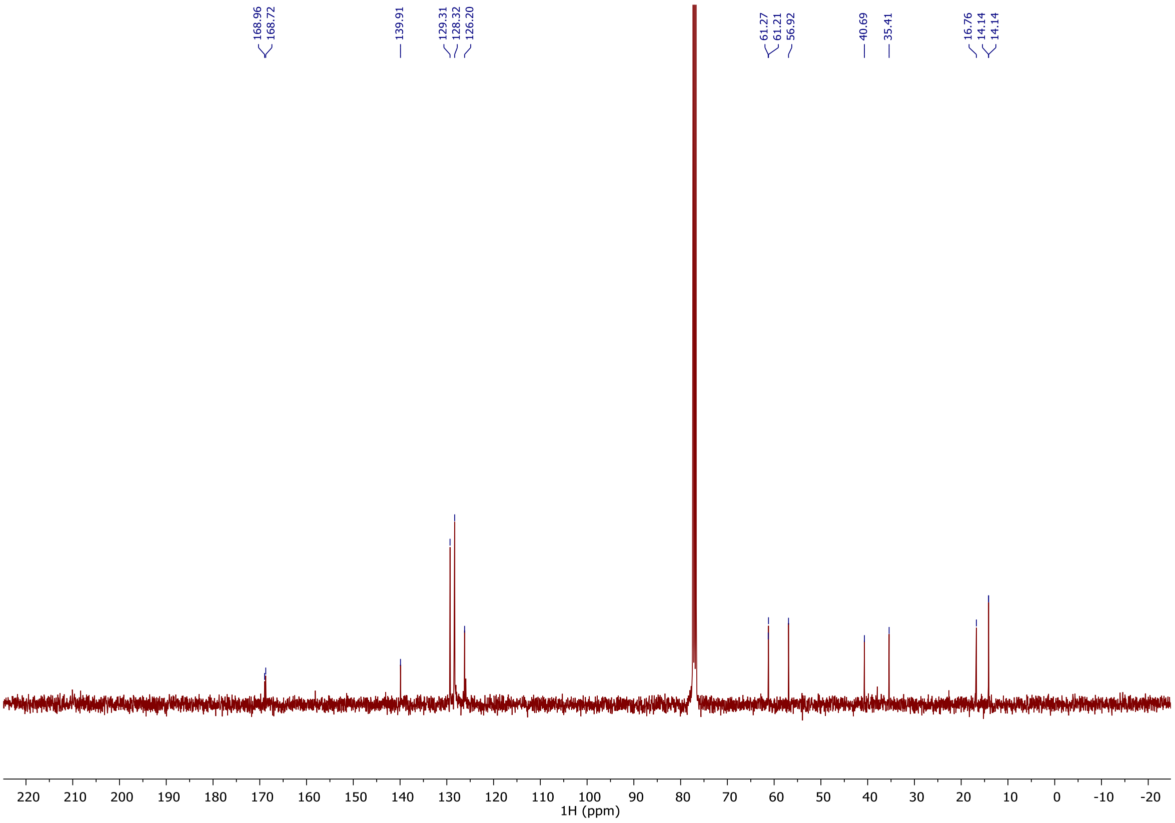

$^{1}H$ NMR (400 MHz, $CDCl_3$) **43**

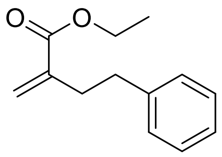

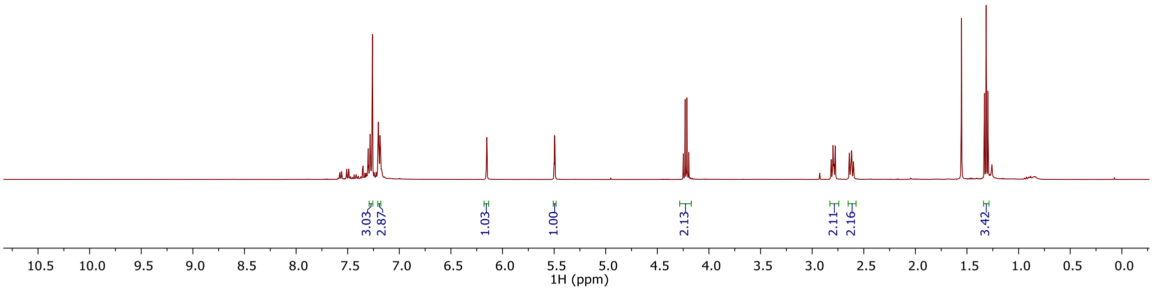


$^{13}C$ NMR(101MHz,$CDCl_3$) **43**

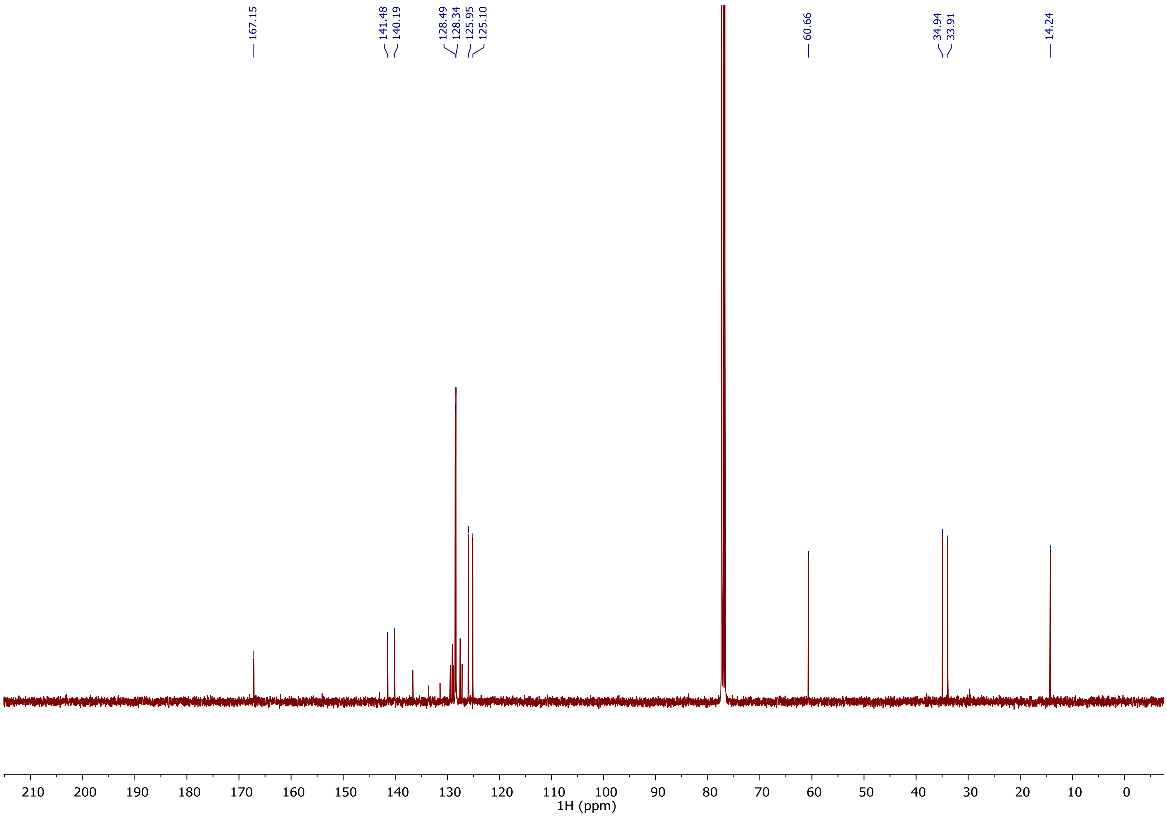

$^{1}H$ NMR (400 MHz, $CDCl_3$) **44**

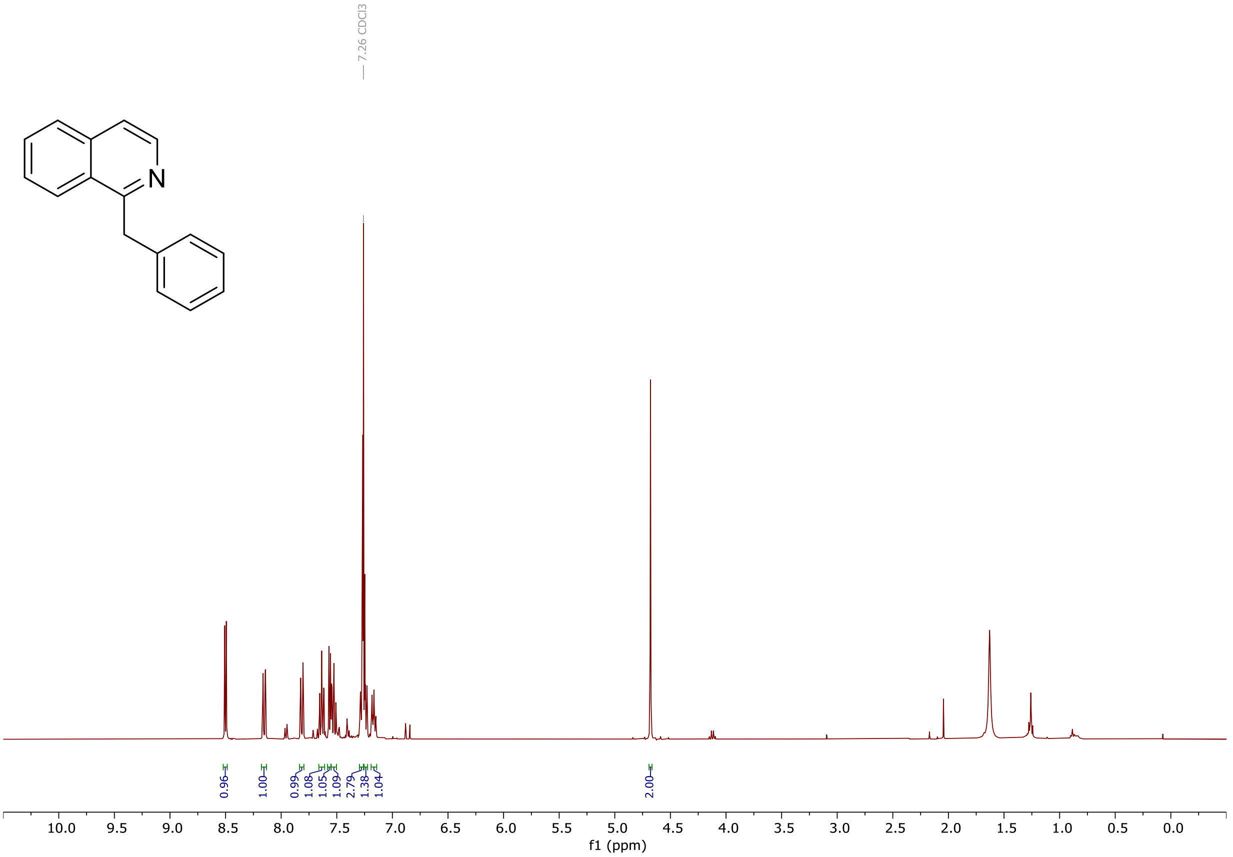


$^{13}C$ NMR(101MHz,$CDCl_3$) **44**

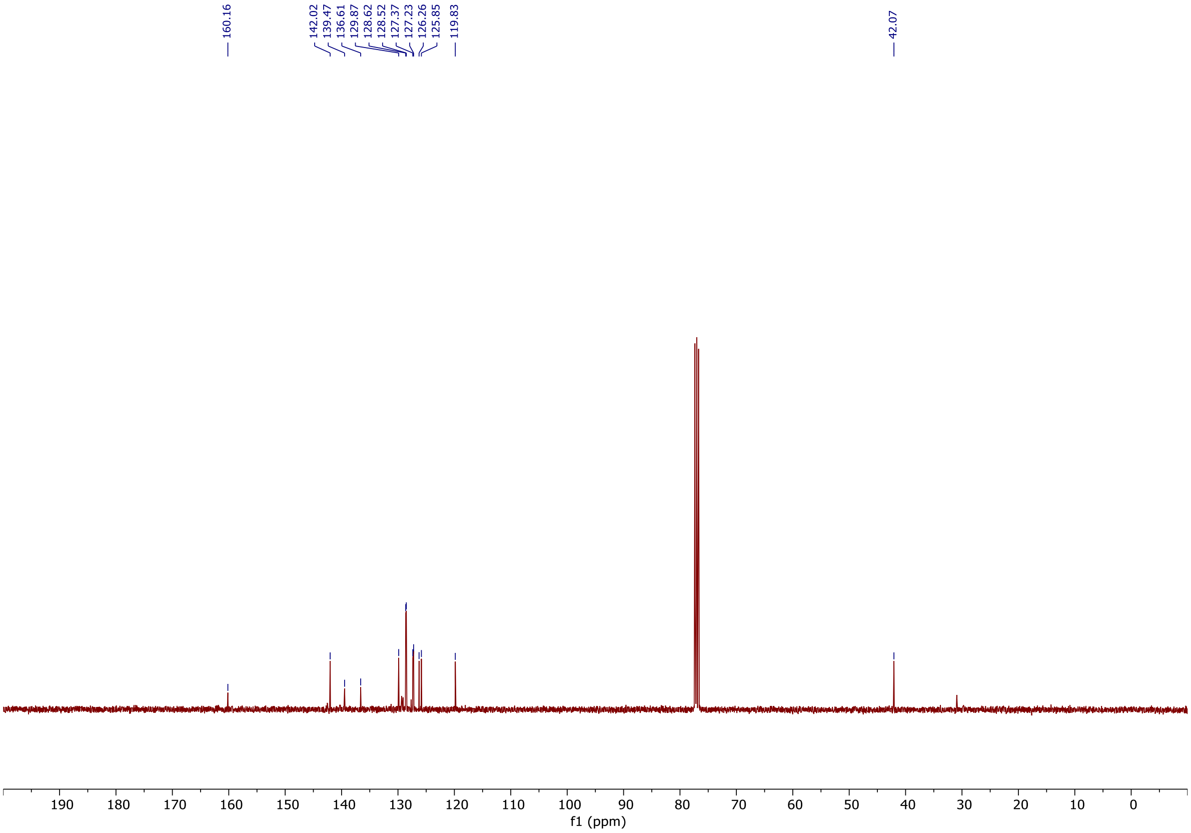

Supplement: Supplementary file 1 [file cs5c07891_si_001.pdf]
