# Supplementary material for: Visible-Light-Driven Benzylation of In Situ-Formed Imines Using Toluenes and Acridine Photocatalysis
Source: ACS Catal. 2026 Feb 3;16(4):3590–9. doi: 10.1021/acscatal.5c07891 (PMC12930518; doi:10.1021/acscatal.5c07891)
Supplement: Supplementary file 2 [file cs5c07891_si_002.pdf]

Supporting Information (protocols)

for

Visible-light-driven benzylation of *in situ*-formed imines using toluenes and acridine photocatalysis

Beatriz Quevedo-Flores[a], Mario Martinez-Lopez[a], Loris Laze[a], Manuel A. Ortuño[b], Irene Bosque[a],* and Jose C. Gonzalez-Gomez[a]*

[a]*Instituto de Síntesis Orgánica (ISO) and Departamento de Química Orgánica, Universidad de Alicante, Apdo. 99, 03080 Alicante, Spain*

[b]*Departamento de Química Física, Universidad de Alicante, 03080 Alicante, Spain*

*Email: josecarlos.gonzalez@ua.es

*Email: irene.bosque@ua.es

Table of contents

MATERIALS AND METHODS 2
Preparation of acridine catalysts; 9-(2-Chlorophenyl)acridine (**A1**): 5
Synthesis of starting material: Ethyl 2-((phenylsulfonyl)methyl)acrylate 5
OPTIMIZATION OF GENERAL PROCEDURE A 6
GENERAL PROCEDURE A 7
SENSITIVITY SCREEN 8
OPTIMIZATION OF GENERAL PROCEDURE B 10
GENERAL PROCEDURE B 11
CALIBRATION OF PRODUCT **1** IN GC-FID: 12
SCALE UP OF GENERAL PROCEDURE A AND B 13
UV-VIS ABSORPTION SPECTRA 18
CALCULATION OF THE OXIDATION POTENTIAL OF THE EXCITED STATE OF A1 22
QUENCHING EXPERIMENTS 25
CYCLIC VOLTAMMETRIES 28
QUANTUM YIELD MEASUREMENT 33
Determination of the photon flux: 33
Determination of the quantum yield of the reaction: 34
COMPUTATIONAL DETAILS 35
OTHER MECHANISTIC STUDIES 37
RADICAL TRAPS 37
TEMPO addition 37
1,1-Diphenylethylene addition 38
DETERMINATION of DIASTEREOMERIC RATIO FOR PRODUCTS **5-9** AND **33** 39
ANALYSIS OF UNDISTILLED ANILINE 45
UNSUCCESSFUL SUBSTRATES USING GENERAL PROCEDURES A AND B. 46
CHARACTERIZATION OF PRODUCTS 47
POST FUNCTIONALIZATION 63
DIFFERENT ACCEPTORS OF BENZYL RADICALS 66
REFERENCES 68

MATERIALS AND METHODS

Solvents and reagents: All solvents and commercially available reagents were purchased as reagent grades and were used without further purification unless otherwise stated.

General methods: All air- and moisture-insensitive reactions were carried out under ambient conditions and monitored by thin-layer chromatography (TLC) and gas chromatography-mass spectrometry (GC-MS). TLCs were performed on silica gel 60 F_{254} on aluminum plates and visualized under ultraviolet light. Flash column chromatography (FC) was performed using an automated flash chromatography system, Teledyne ISCO CombiFlash NEXTGEN 100 apparatus, using a Teledyne ISCO RediSep Bronze or Gold 4 g silica cartridge packed. HPLC-grade or distilled solvents were used for chromatography.

Photochemistry: The photochemical reactions were carried out in an Evoluchem PhotoRedOx Box Duo^{TM} reactor (for more details, see: https://www.hepatochem.com/photoreactors-leds-accessories/photoredox-duo/) equipped with two 18 W EvoluChem LEDs 450PF radiating at 450 nm with a total irradiance of about 30 W $\times m^{-2}$ (for light spectrum and other details, see: https://www.hepatochem.com/product/hck1012-xx-002/). Vials of borosilicate glass were placed at fixed positions of the vial holder designed for the Evoluchem photoreactor. With the internal fan on, the reaction temperature was determined to be between 25 °C and 30 °C. To ensure the reproducibility of the reactions, the light power received at each position of the photoreactor was measured using a StellarNet Inc. BLUE-Wave-VIS spectrometer, F600-VISNIR fiber optic cable, and RAD-CAL NIST traceable Radiant Flux calibration in Watts/nm.

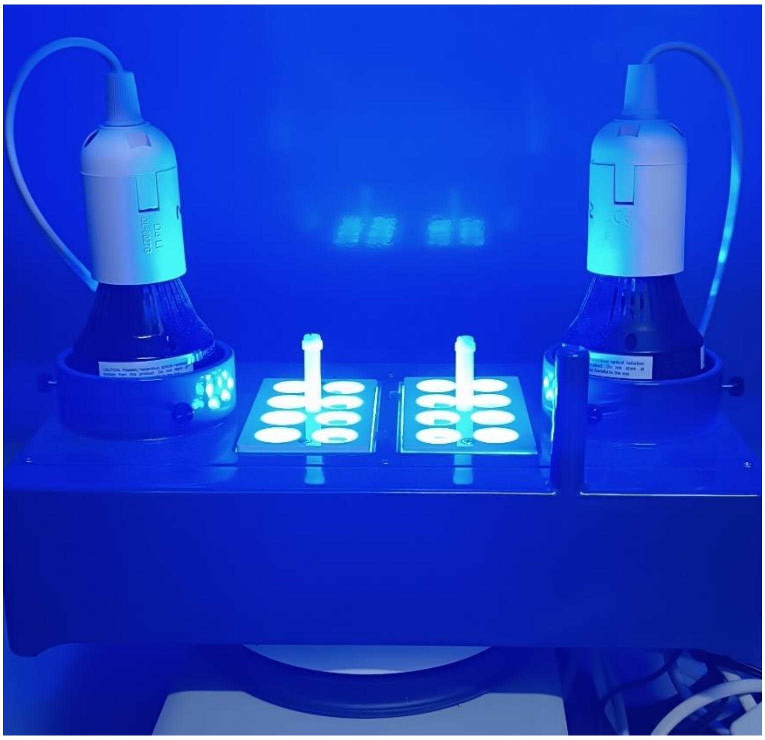

Figure S1. Experimental set-up for photochemical reactions.

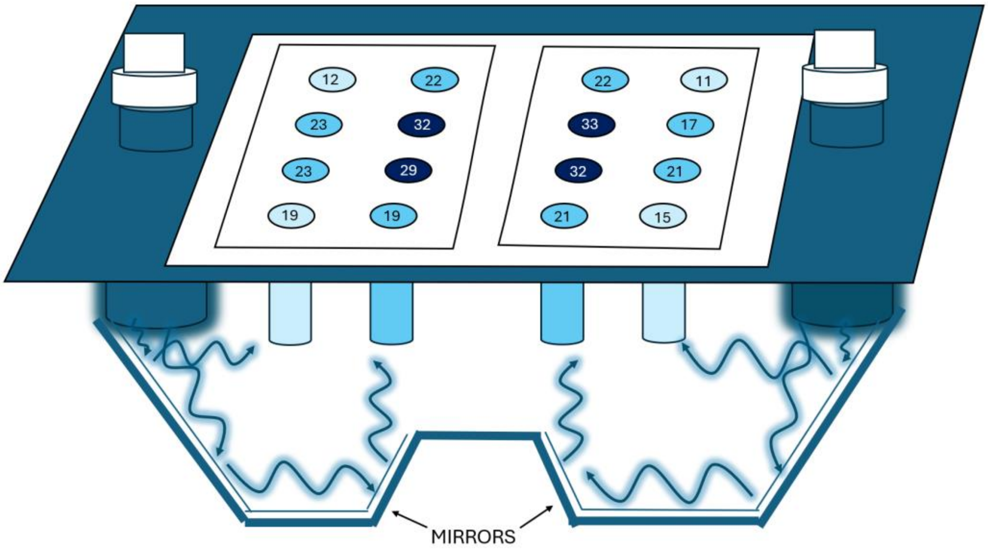

Figure S2. Scheme of photoreactor and values of irradiance in W/m^2.

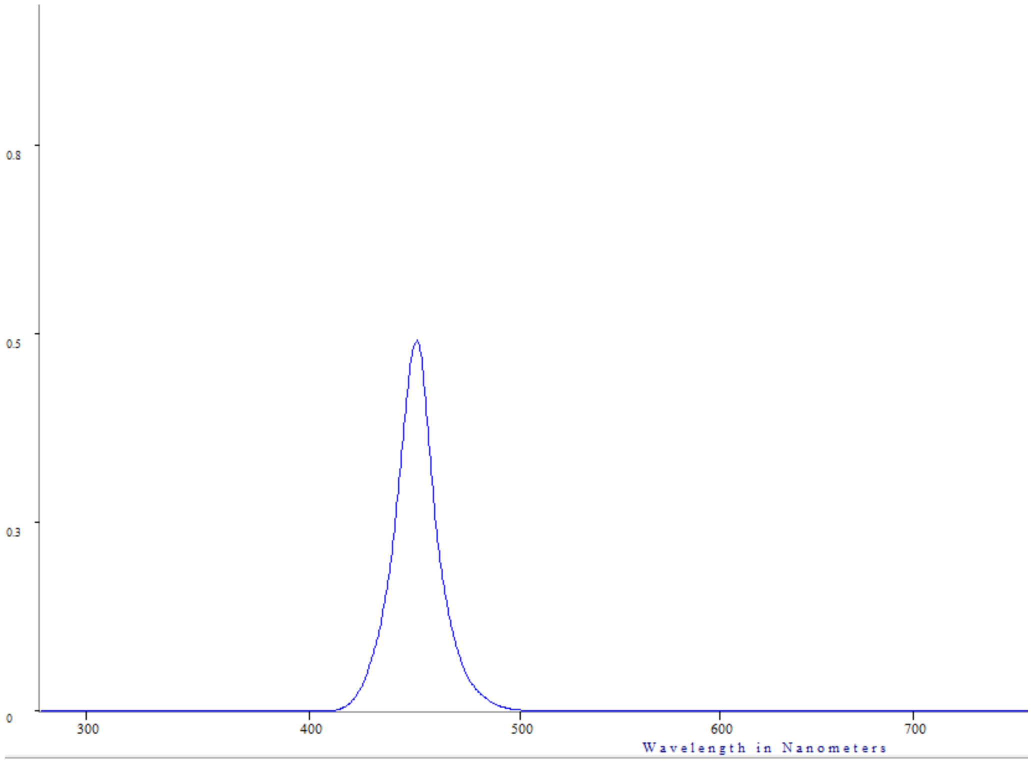

Figure S3. Emission spectra of the blue LED.

Analytical Information:

NMR spectra were recorded at 300 or 400 MHz for ^{1}H and 75 or 101 MHz for ^{13}C, in $CDCl_3$. For ^{1}H-NMR, the residual signal was used as the internal standard (7.26 ppm). Data are reported as (s = singlet, d = doublet, t = triplet, q = quartet, m = multiplet or unresolved, brs = broad signal, coupling constant(s) in Hz, integration). ^{13}C-NMR spectra were recorded with ^{1}H-decoupling at 101 MHz and referenced to $CDCl_3$ at 77.16 ppm.

LRMS were obtained using an Agilent 5977B mass spectrometer with a quadrupole analyzer, coupled to an Agilent 8890 gas chromatograph. The oven temperature was: 3 min at 80 ºC, then 20 ºC/min ramp until 300 ºC, then 3 min at 300 ºC.

HRMS analyses were carried out in electron impact (EI) mode at 70 eV using a quadrupole mass analyzer, or by Q-TOF in electrospray ionization (ESI) mode.

ICP-MS was obtained using an Agilent 7700x.

UV-vis spectra were recorded on a Horiba Duetta fluorescence and absorbance spectrometer, using quartz cuvettes with a beam path of 1 cm and a volume of 3 mL.

Quenching experiments were performed on a Horiba Duetta fluorescence and absorbance spectrometer, using quartz cuvettes with a 1 cm path length and a 3 mL volume. Monochromators were set with 3 nm of entrance and exit slit for excitation and emission monochromators, and an integration time of 0.05 s was selected.

GENERAL METHODS

Preparation of acridine catalysts; 9-(2-Chlorophenyl)acridine (**A1**):

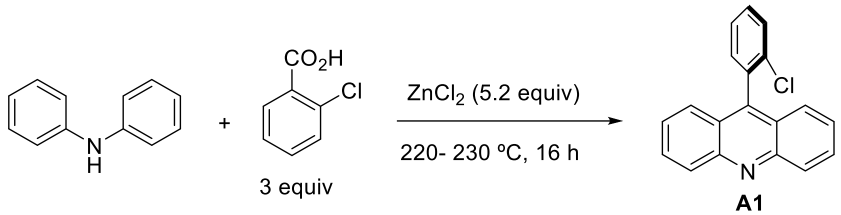

Following a reported protocol,[1] to a 20-mL pressure tube, diphenylamine (1.00 g, 5 mmol), 2-chlorobenzoic acid (2.40 g, 15 mmol, 3 equiv.), and zinc chloride (3.60 g, 26 mmol, 5.2 equiv.) were added. The tube was capped with a screw cap, and the reaction mixture was stirred at 220-230 °C in an oil bath for 16 h. After this time, the resulting thick green solid was treated with a saturated ammonium hydroxide solution (50 mL, 30% w/w), vigorously scratched with a spatula, and extracted with EtOAc (3 x 60 mL). The collected organic layers were washed with brine and dried over $MgSO_4$. Removal of the solvent and purification by FC eluting with a gradient from 0% to 20% EtOAc in *n*-hexane afforded acridine **A1** as a pure yellow solid (1.020 g¸70%). This solid could also be recrystallized from hot toluene to obtain crystalline **A1**. The spectroscopy data and NMR matched those previously reported.[1]

TLC: Rf = 0.29 (9:1 hexane/EtOAc, UV)

GC (T_i= 80 ºC): R_t 10.111 min.

MS: *m/z* (%) 291 (M^+, ^{37}Cl, 26), 289 (M^+, ^{35}Cl, 77) 254 (100), 126 (32), 291 (31), 253 (29).

Synthesis of starting material: Ethyl 2-((phenylsulfonyl)methyl)acrylate

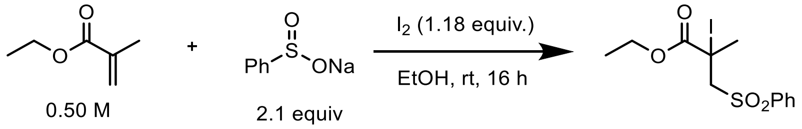

In a 25 mL round-bottom flask equipped with a magnetic stirring bar, ethyl methacrylate (2 mmol, 249 µL) and sodium benzenesulfinate (2.1 equiv., 4.2 mmol, 685 mg) were added in ethanol (0.05 M, 4 mL). Then, I_2 (1.18 equiv., 2.36 mmol, 598 mg) was added at 0 ºC. The reaction mixture was warmed to room temperature and stirred overnight under an argon atmosphere. Once finished, DCM (10 mL) was added to the reaction mixture, which was washed with H_2O (5 mL), $NaHCO_3$ (5 mL) and $Na_2S_2O_4$ (2 x 5 mL). The organic phase was concentrated under reduced pressure, and the resulting residue was used for the next step without further purification.

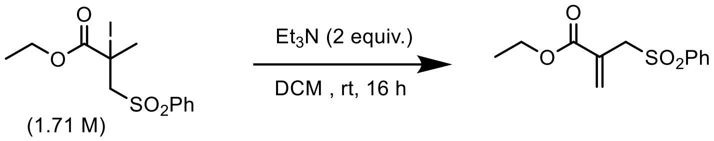

The crude product from the previous step was dissolved in dry DCM (1.5 mL), and Et_3N (2 equiv., 4 mmol, 556 µL) was added dropwise to the reaction. The mixture was under an argon atmosphere at room temperature for 9 h. Once finished, the reaction mixture was concentrated under reduced pressure. The product was obtained as a colorless oil (250 mg, 0.98 mmol, 50%, 2 steps) after FC using a gradient from 0% to 80% of EtOAc in *n*-hexane as the eluent. The spectroscopy data and NMR matched those previously reported in the literature.[2]

OPTIMIZATION OF GENERAL PROCEDURE A

Table S1: Screening of reaction conditions for product **1**.

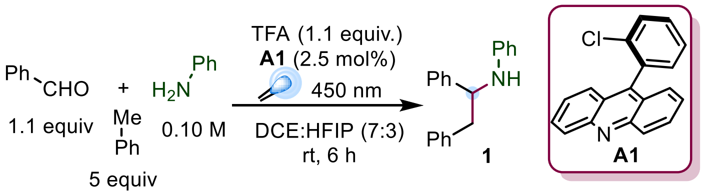

Entry	Conditions	%Yield[a]
1	MeCN as solvent	20
2	2 h	85
3	20 mol% PyOx 16 h	95
4	w/o PyOx	98
5	*w/o* PC at 450 nm	0
6	*w/o* PC at 405 nm	15
7	*w/o* TFA or HFIP	0
8	*w/o* air	>98
9	*w/o* light	0
10	DCE:HFIP (9:1)	72
11	DCE:HFIP (8:2)	92
12	HFIP as solvent	65

[a]Yield determined by ^{1}H-NMR with an internal standard.

GENERAL PROCEDURE A

General procedure A (GPA) for the amino-benzylation of aldehydes with toluenes and anilines:

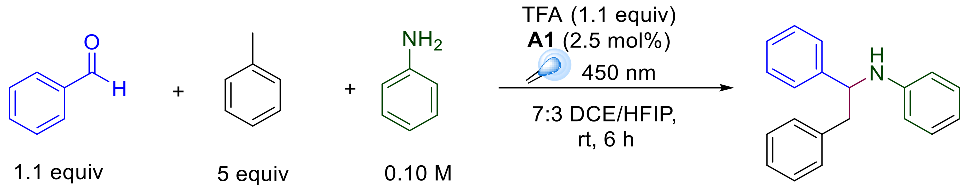

In a two-dram vial equipped with a magnetic stirring bar, distilled aniline[a] (0.30 mmol, 27 μL), distilled benzaldehyde (1.1 equiv, 0.33 mmol, 34 μL), and toluene (5 equiv, 1.50 mmol, 158 μL) were added, followed by 9-(2-chlorophenyl)acridine (**A1**, 2.5 mol%, 2.2 mg, 0.0075 mmol). A mixture of 1,2-dichloroethane (DCE, 2.1 mL) and 1,1,1,3,3,3-hexafluoro-2-propanol (HFIP, 0.9 mL) was then added to the vial, followed by trifluoroacetic acid (TFA, 1.1 equiv, 0.33 mmol, 26 μL). The vial was sealed and placed in the PhotoRedOx Box Duo photoreactor. The reaction mixture was irradiated with blue LEDs (λ = 450 nm) for 6 hours at room temperature (approximately 25–30 °C, controlled by a fan). After completion, the reaction mixture was concentrated under reduced pressure, and the resulting residue was dissolved in ethyl acetate (EtOAc). The mixture was then quenched by adding K_2CO_3 (approximately 40 mg), stirred for 30 minutes, and filtered. The crude mixture was concentrated under reduced pressure. The resulting residue was purified by flash column chromatography (silica gel, using Hexane as eluent) to afford the desired product.

[a] *We found that using distilled aniline is crucial for obtaining optimal, reproducible results, even when using a new bottle. Distilled aniline can be stored under an argon atmosphere at 0 – 5 ºC for over 1 month with similar results.*

SENSITIVITY SCREEN

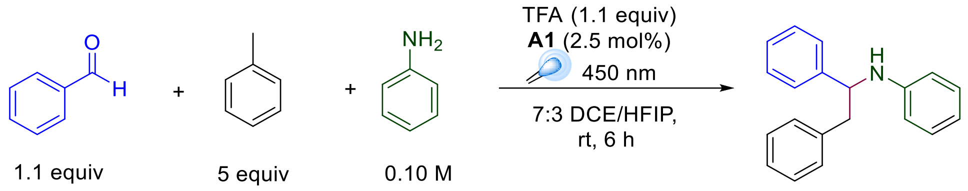

Performed according to a modified reported protocol.[3] All experiments were performed on a 0.30 mmol scale using the general procedure A, with different modifications given in the following table.
All the experiments were placed in the PhotoRedOx Box Duo photoreactor. The reactions were irradiated with blue LEDs (λ = 450 nm) for 6 hours at room temperature (approximately 25–30 °C), with the reaction temperature controlled by a fan (except entry 11). Once the reactions were finished, the reaction yields were obtained by GC-FID.

Table S2: Results of sensitivity screen.

Entry	Modification	Deviation from standard conditions	Yield % [GC][a]	Deviation from benchmark
1	None	General procedure A	98	–
2	Undistilled aniline	Undistilled aniline	48	-50
3	Low O_2	Mixture sparged with argon for over 60 s before closing the vial	100	+2
4	High O_2	Mixture sparged with air for over 60 s before closing the vial	84	-14
5	Low H_2O	Molecular Sieves 4 Å were added	21	-77
6	High H_2O	30 µL of H_2O was added	99	+1
7	Medium I[b]	$I \approx 20$ W/m^2 (Benchmark: $I \approx 30$ W/m^2)	94	-4
8	Low I[b]	$I \approx 10$ W/m^2 (Benchmark: $I \approx 30$ W/m^2)	85	-13
9	Higher concentration	[aniline] = 0.11 M	100	+2
10	Lower concentration	[aniline] = 0.090 M	100	+2
11	Higher temperature	Fan off (45ºC)	75	-23

[a] Yields were determined by GC analysis using adamantane as an internal standard.[b] Check Figure S2 to see the positions in the photoreactor where this irradiance was obtained.

The analysis of the experimental results indicates that the decrease in reaction efficiency is primarily associated with the purity of the aniline employed, demonstrating the necessity of distilling the reagent before its use. Moreover, the experiments conducted in the presence of molecular sieves or H_2O revealed that water does not have a detrimental effect on the reaction outcome. For the remaining experimental conditions, the reaction was sensitive to temperature increases and less sensitive to irradiance, with low sensitivity to other parameters (e.g., concentration), confirming the robustness and reproducibility of the protocol.

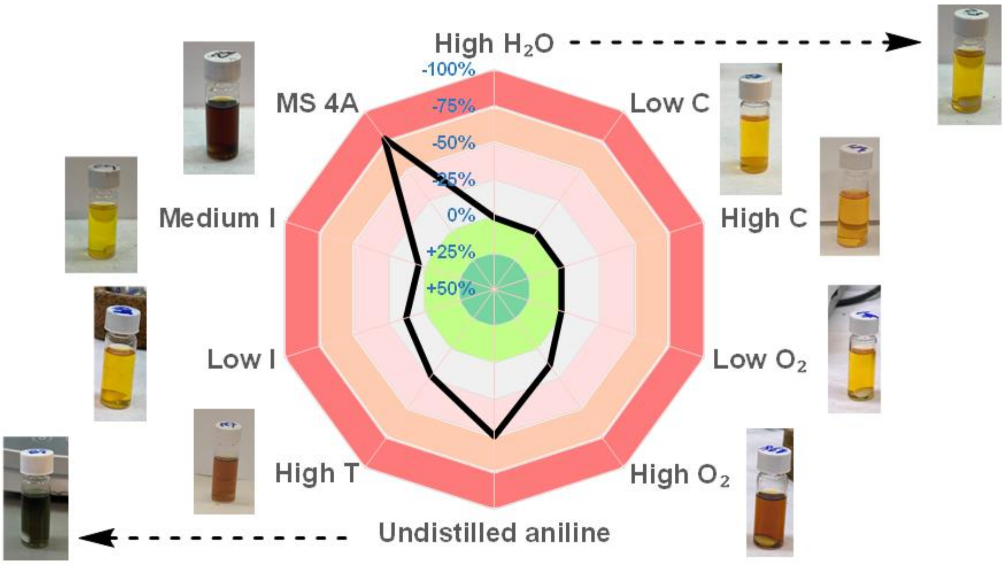

Figure S4: Radar diagram representation of the sensitivity screen.

OPTIMIZATION OF GENERAL PROCEDURE B

Optimization of reaction conditions (GPB) with *p*-methoxyphenylaniline ($PMPNH_2$):

Table S3: Screening of reaction conditions for product **30**.

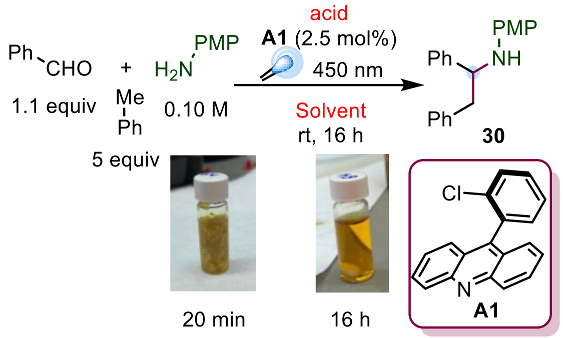

Entry	Conditions	%Yield[a]
1	TFA (1.1 equiv) in MeCN	0
2	TFA (1.1 equiv) in DCE	0
3	TsOH (1.1 equiv) in 7:3 DCE/HFIP	0
4	TsOH (1.1 equiv) in MeCN	91 (88)
5	TsOH (20 mol%) in MeCN	40
6	TsOH (1.1 equiv) in MeCN, ***w/o*** A1	0
7	TFA (1.1 equiv) in 7:3 DCE/HFIP, at 405 nm	0
8	TsOH (1.1 equiv) in MeCN, at 405 nm	80

[a]Yield determined by ^{1}H-NMR with an internal standard. In parentheses, the yield for the isolated pure product.

GENERAL PROCEDURE B

General procedure B (GPB) for the use of 4-methoxyaniline (including synthesis of **30**):

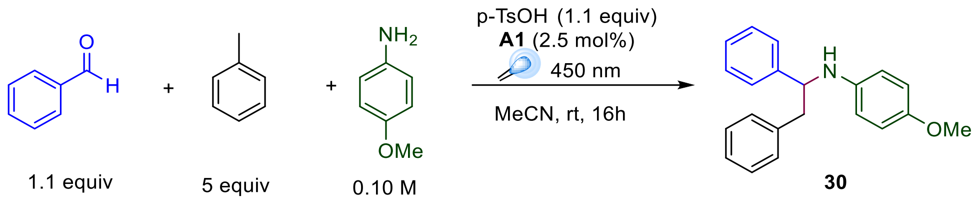

In a two-dram vial equipped with a magnetic stirring bar, 4-methoxyaniline (0.30 mmol, 37 mg), distilled benzaldehyde (1.1 equiv, 0.33 mmol, 34 µL), and toluene (5 equiv, 1.50 mmol, 158 µL) were added, followed by 9-(2-chlorophenyl)acridine (**A1**, 2.5 mol%, 2.2 mg, 0.0075 mmol). Acetonitrile (3 mL) was then added to the vial, followed by *p*-Toluenesulfonic acid (*p*-TsOH,1.1 equiv, 0.33 mmol, 56 mg). The vial was sealed and placed in the PhotoRedOx Box Duo photoreactor. The reaction mixture was irradiated with blue LEDs (λ = 450 nm) for 16 hours at room temperature (approximately 25–30 °C, controlled by a fan). Once finished, EtOAc (10 mL) was added to the reaction mixture, which was then washed with sat. aq. K_2CO_3 (3 x 5 mL). The combined organic phases were concentrated under reduced pressure, and the resulting residue was purified by flash column chromatography (silica gel, using Hexane as eluent) to afford the desired product.

CALIBRATION OF PRODUCT **1** IN GC-FID:

The formation of product **1** was quantified by GC-FID analysis using adamantane as an internal standard (IS). Two stock solutions of 0.100 M in EtOAc were prepared for **1** and IS. GC samples were prepared by adding 30, 40, 60, 80, or 100 µL of the compound **1** stock solution and mixing with 100 µL of the IS stock solution. A total volume of 1 mL was achieved using EtOAc. These prepared samples were directly injected into the GC [in the method used, retention times are: IS (6 min); **1** (18 min)].

Table S4: Calibration data obtained from direct integration of the GC peaks.

Table S4: Calibration data.

Volume IS + P (µL)	mmoles IS	mmoles P	Area P/Area IS	mmol P/mmol IS
100 + 30	0.01	0.003	0.311838306	0.300
100 + 40	0.01	0.004	0.467631379	0.400
100 +60	0.01	0.006	0.859838275	0.600
100 + 80	0.01	0.008	1.345945946	0.800
100 + 100	0.01	0.010	1.629560337	1.000

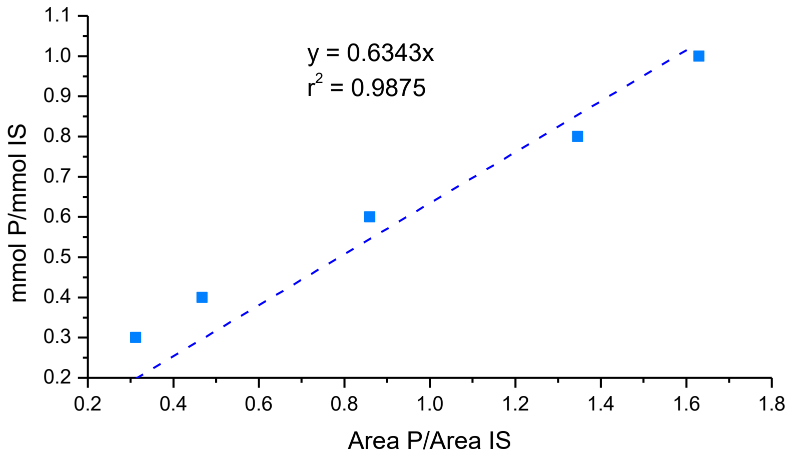

Therefore, for a 0.30 mmol scale, the yield can be determined using the following equation:

$$\% \, Yield \, (\mathbf{1}) = 0.6343 \, \frac{Area \, \mathbf{1}}{Area \, IS} \, x \, mmol \, IS \, x \, \frac{100}{0.3} \qquad \boldsymbol{Eq \, 1.1}$$

SCALE UP OF GENERAL PROCEDURE A AND B

Scale up of general procedure A

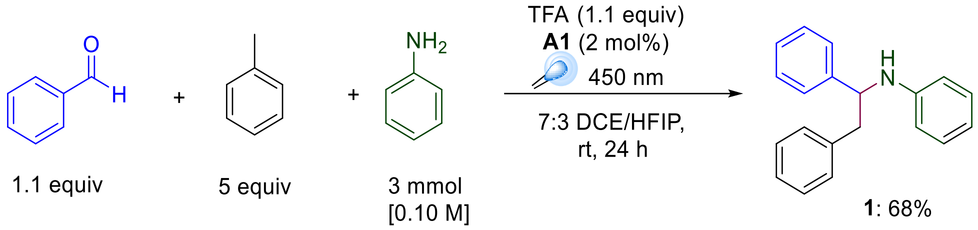

In a 100 mL Schlenk tube with a magnetic stirring bar, distilled aniline (3 mmol, 270 µL), distilled benzaldehyde (1.1 equiv., 3.3 mmol, 336 µL), and toluene (5 equiv., 15 mmol, 1.59 mL) were added, followed by **A1** (2 mol%, 17 mg). A mixture of 1,2-dichloroethane (DCE, 21 mL) and 1,1,1,3,3,3-hexafluoro-2-propanol (HFIP, 9 mL) was then added to the vial, followed by trifluoroacetic acid (TFA, 1.1 equiv., 3.3 mmol, 261 µL). The reaction mixture was irradiated with two blue LEDs (λ = 450 nm) at 5 cm for 24 hours at room temperature (approximately 40–45 °C, controlled by a fan). After completion, the reaction mixture was concentrated under reduced pressure, and the resulting residue was dissolved in ethyl acetate EtOAc (10 mL). The mixture was then quenched by adding K_2CO_3 (approximately 200 mg), stirred for 30 minutes, and filtered. The crude mixture was concentrated under reduced pressure, and the residue was purified by flash column chromatography using a gradient from 0% to 10% of EtOAc in *n*-hexane as the eluent. Product **1** was obtained as a colorless solid (552 mg, 2.02 mmol, 68%).

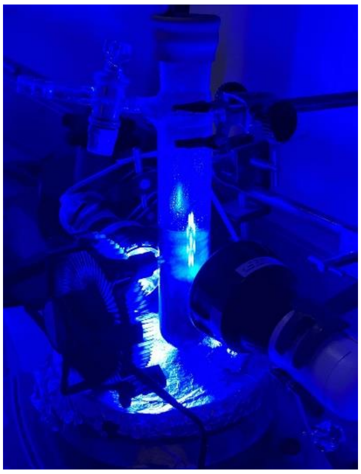

Figure S5: Set-up for general procedure A at 3-mmol scale.

Flow conditions of general procedure A for product 1

For a reaction scale of 3 mmol and a photoreactor volume of 2 mL, various flow rates were investigated to determine the optimal residence time.

Table S5: Screening flow conditions for product **1** under GPA.

Φ (mL/min)	t_R (min)	Yield GC (%)[a]
0.06	30	100%
0.10	20	96%
0.13	15	71%
0.20	10	43%

[a] Yields were determined by GC-FID analysis using adamantane as an internal standard.

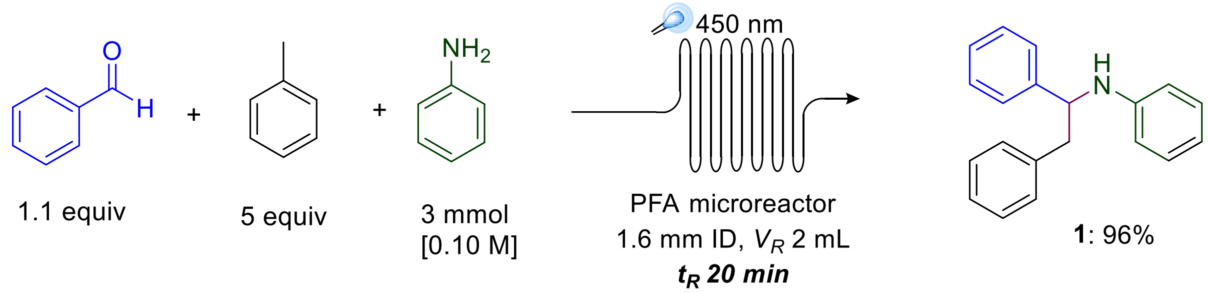

In a 100 mL flask, distilled aniline (3 mmol, 270 µL), distilled benzaldehyde (1.1 equiv., 3.3 mmol, 336 µL), and toluene (5 equiv., 15 mmol, 1.59 mL) were added, followed by **A1** (2 mol%, 17 mg). A mixture of 1,2-dichloroethane (DCE, 21 mL) and 1,1,1,3,3,3-hexafluoro-2-propanol (HFIP, 9 mL) was then added to the vial, followed by trifluoroacetic acid (TFA, 1.1 equiv., 3.3 mmol, 261 µL). The reaction mixture was injected into a 30 mL syringe, then pumped at 0.10 mL/min into the photobox, irradiated with two blue LEDs (450 nm), and recollected into a 100 mL round-bottom flask connected to an argon balloon. After time completion, the reaction mixture was concentrated under reduced pressure, and the resulting residue was dissolved in ethyl acetate EtOAc (10 mL). The mixture was then quenched by adding K_2CO_3 (approximately 200 mg), stirred for 30 minutes, and filtered. The crude mixture was concentrated under reduced pressure, and the residue was purified by flash column chromatography using a gradient from 0% to 10% of EtOAc in *n*-hexane as the eluent. Product **1** was obtained as a colorless solid (790 mg, 2.89 mmol, 96%).

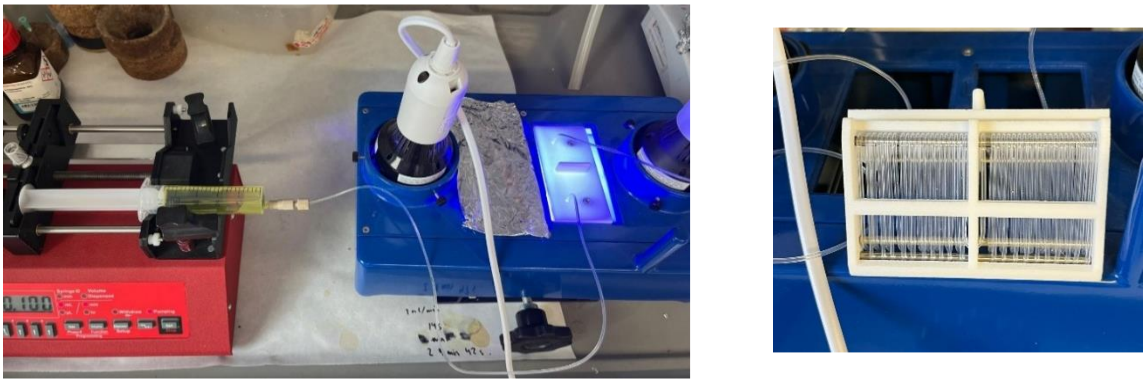

Figure S6: Set-up for flow reaction.

Scale up of general procedure B

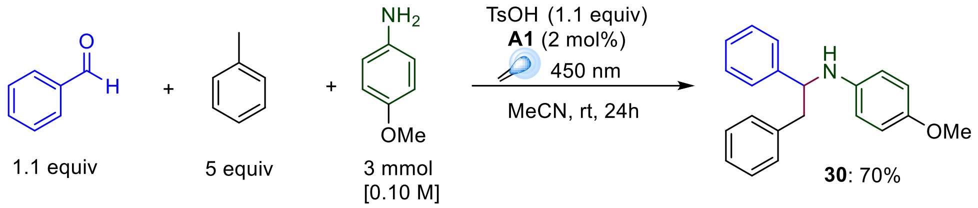

In a 100 mL Schlenk tube equipped with a magnetic stirring bar, 4-Methoxyaniline (369 mg, 3 mmol) was added to a reaction mixture consisting of distilled benzaldehyde (1.1 equiv., 3.3 mmol, 336 µL) and toluene (5 equiv., 15 mmol, 1.59 mL) in acetonitrile (30 mL). Then, **A1** (2 mol%, 17 mg) and TsOH (1.1 equiv., 3.3 mmol, 570 mg) were added, and a precipitate formed (*vide infra*, Fig. S8). The reaction mixture was irradiated with two blue LEDs (450 nm) at 5 cm and stirred at room temperature (approximately 40–45 °C, controlled by a fan) for 24 hours, during which the precipitate disappeared. The resulting mixture was concentrated under vacuum, and the residue was treated with sat. aq. K_2CO_3 (20 mL) and extracted with EtOAc (3 x 50 mL). The combined organic layers were concentrated, and the residue was purified by flash column chromatography using a gradient from 0% to 10% of EtOAc in *n*-hexane as the eluent. The product was obtained as a colorless oil (645 mg, 2.12 mmol, 70%).

(a) (b)

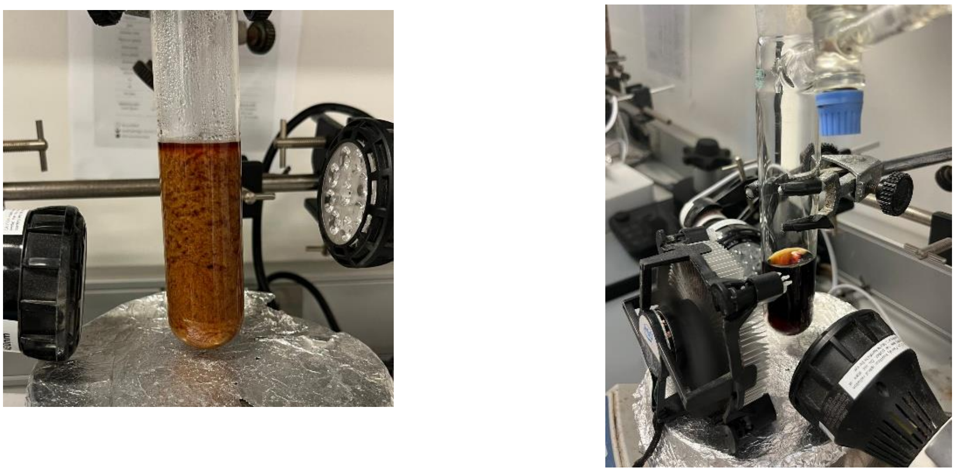

Figure S7: Set-up for GPB at 3-mmol scale. (a) At the beginning. (b) At the end.

The precipitate was isolated by filtration from the mixture. The remaining solid was distributed between EtOAc and sat. aq. K_2CO_3. The aqueous phase was extracted with EtOAc, and the collected organic layers were dried over $MgSO_4$. Upon concentration under reduced pressure, a yellowish solid was obtained, which was identified as the corresponding iminium and *p*-TsOK by NMR.

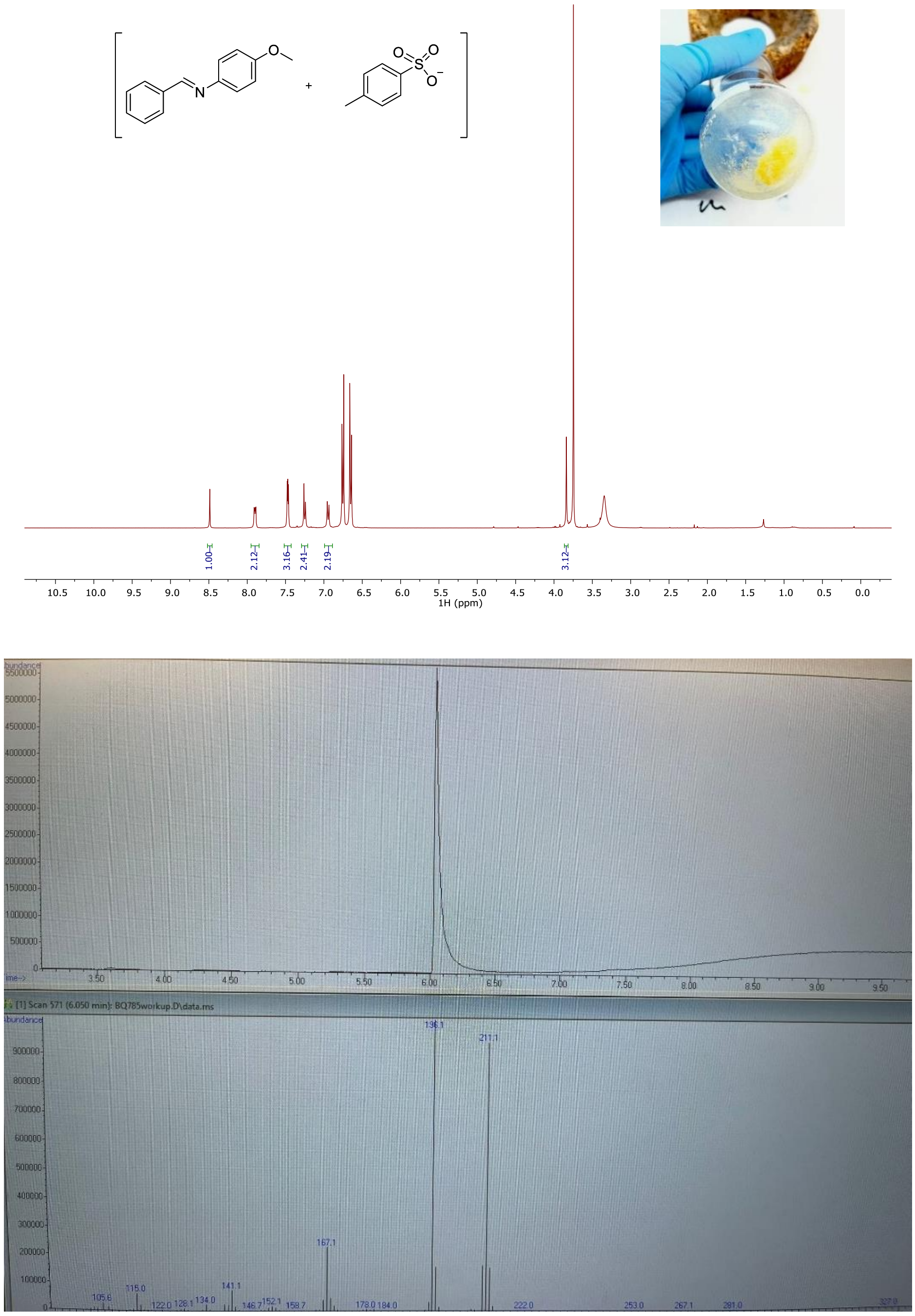

Figure S8: a) NMR and b) GC/MS of the precipitate.

UV-VIS ABSORPTION SPECTRA

Preparation of **A1**+TFA (**[HA1]**$^+$) stock solution (**stock solution A**): A 0.4 mM solution of **A1** was prepared by mixing 6 mg of **A1** in DCE: HPIF and 100 µL of TFA up to a final volume of 50 mL. From this solution, 1.5 mL (0.0006 mmol of **A1**) was taken and diluted to 3 mL with DCE: HFIP (7:3) or with quenchers, as appropriate. Thus, the final concentration of photocatalyst **[HA1]**$^+$ was always 0.2 mM. The same was done when the solution was prepared in MeCN or when TsOH was used instead of TFA.

Preparation of stock solution of iminium from benzaldehyde and aniline (**Im1-H**$^+$) (**Stock solution B**): A mixture of 82.5 µL of $PhNH_2$ (0.9 mmol), 100.8 µL of PhCHO (0.99 mmol, 1.3 equiv) and 300 µL of TFA (4 equiv) in 3 mL of DCE: HFIP (7:3) was mixed for 30 min at room temperature (30 ºC). The solution was greenish. The same was done when the solution was prepared in MeCN or using TsOH instead of TFA.

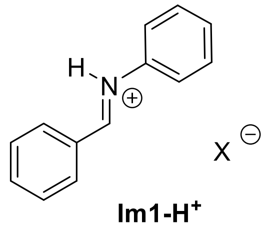

Im1-H$^+$

Preparation of stock solution of iminium from benzaldehyde and *p*-methoxyaniline (**Im30-H**$^+$) (**Stock solution C**): A mixture of 110.7 mg of $pOMeC_6H_4NH_2$ (0.9 mmol), 100.8 µL of PhCHO (0.99 mmol, 1.3 equiv) and 300 µL of TFA (4 equiv) in 3 mL of DCE: HFIP (7:3) was mixed for 30 min at room temperature (30 ºC). The solution was intensely yellow. The same was done when the solution was prepared in MeCN or when TsOH was used instead of TFA.

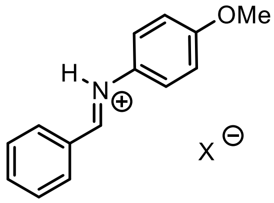

Im30-H$^+$

The absorbance of **[HA1]**$^+$ and iminium **Im1-H**$^+$ solutions were recorded, as well as the absorbance of the mixture: For **[HA1]**$^+$ solution, 1.5 mL of stock solution A were diluted up to 3 mL with DCE: HFIP (7:3). For the iminium **Im1-H**$^+$ solution, 200 µL of the stock solution B were taken and diluted up to 3 mL with DCE: HFIP (7:3). For the mixture, 1.5 mL of stock solution A were mixed with 200 µL of stock solution B and diluted up to 3 mL with DCE: HFIP (7:3).

The absorbance of the iminium **Im30-H**$^+$solution was recorded and added to the previous UV-visible spectra. For the iminium **Im30-H**$^+$ solution, 200 µL of stock solution C was diluted to 3 mL with DCE: HFIP (7:3).

Stock solutions B and C were prepared the same way, but using MeCN instead of DCE: HFIP. Then, 200 µL of the stock solutions (B and C in MeCN) were taken and diluted up to 3 mL with MeCN (7:3). Here, we observe that in DCE: HFIP, the absorbance is much more red-shifted than in MeCN for both iminium ions.

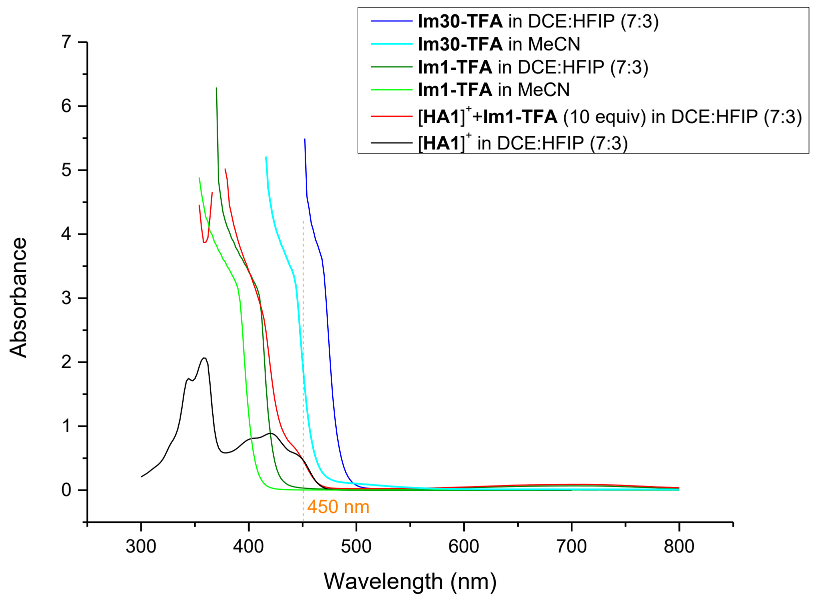

Figure S9: UV-Vis absorption spectra for iminium **Im1-H**$^+$ and **Im30-H**$^+$, both in DCE: HFIP (7:3) and MeCN, and for **A1** (stock solution A) and in the presence of 10 equivalents of **Im1-TFA**.

As observed in Figure S9, the absorbance of iminium **Im30-H**$^+$ is significantly higher than that of **A1** (there are 10 equiv of iminium with respect to the photocat, and in the real reaction, there are 40 equiv). That is why **A1** shows no light absorption at 450 nm when this iminium is present (shadow effect).

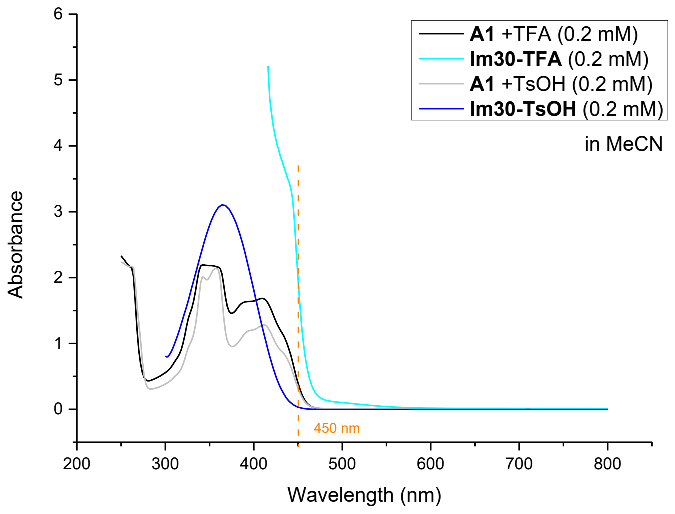

Figure S10: UV-Vis absorption spectra for **A1** (0.2 mM) (using TFA and *p*-TsOH acid), and for iminium **Im30** with both acids.

As observed in Figure S10, when iminium **Im30-H$^+$** is formed in MeCN, the same behavior occurs with TFA as before, and the iminium absorbs all light at 450 nm. However, when the acid is changed to TsOH, the iminium precipitates, which means that the amount in solution is much lower (around 2.4 µM, determined by preparing by mixing the reagents in MeCN and diluting until a clear solution stays for at least 1 h without any precipitation), and now light can penetrate and reach the photocatalyst at 450 nm. Under these conditions, the reaction proceeds, albeit much more slowly, probably due to the low iminium concentration. Also, light penetration in the presence of precipitated iminium is less efficient (decreased illuminated solution volume, increased reaction time).

Preparation of **A1**+ p-TsOH (**[HA1]$^+$**)

A 0.4 mM solution of **A1** was prepared by mixing 6 mg of **A1** in MeCN and 220 mg of p-TsOH up to a final volume of 50 mL. From this solution, 1.5 mL (0.0006 mmol of **A1**) was taken and diluted to 3 mL with MeCN. Thus, the final concentration of photocatalyst **[HA1]$^+$** was always 0.2 mM.

Preparation of (**Im30-H$^+$**)

A mixture of $pMeC_6H_4NH_2$ (0.3 mmol), PhCHO (0.33 mmol, 1.1 equiv) and 57 mg of p-TsOH (1 equiv) in 3 mL of MeCN was mixed for 1h min at room temperature (24 ºC). The solution was intensely yellow. Then, the precipitated was filtered, and from this solid was taken 6.3 mg and up to 10 mL of MeCN (0.003M).

The absorbance of **[HA1]$^+$** and iminium **Im30-H$^+$** solutions were recorded, as well as the absorbance of the mixture: For **[HA1]$^+$** and **Im30-H$^+$** solution, 0.3 mL of stock solution **[HA1]$^+$** were diluted up to 3 mL with MeCN. For the iminium **Im30-H$^+$** solution, 2.7 mL of the stock solution **Im30-H$^+$** were taken and diluted up to 3 mL with MeCN. For the mixture, 0.3 mL of stock solution **[HA1]$^+$** were mixed with 2.7 mL of stock solution **Im30-H$^+$**.

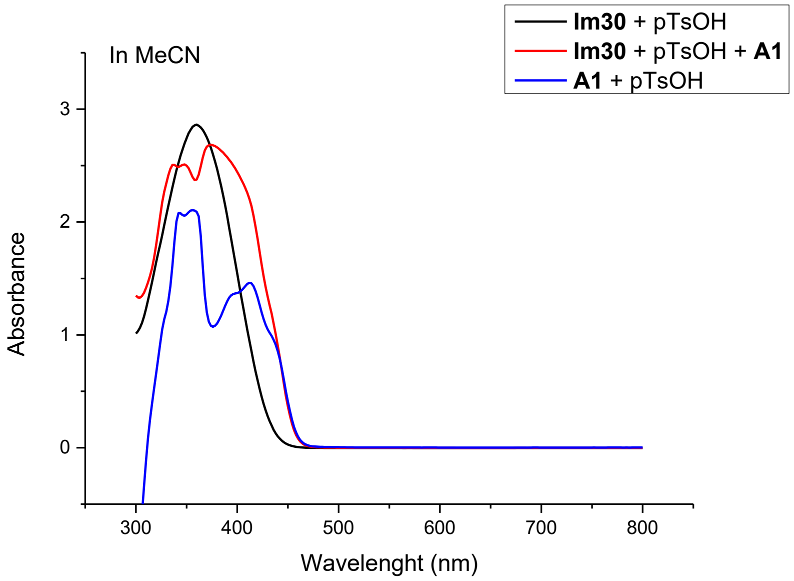

Figure S11: UV-Vis absorption spectra for **A1** (0.2 mM) (using *p*-TsOH acid), for iminium **Im30-**pTsOH and for the mixture.

CALCULATION OF THE OXIDATION POTENTIAL OF THE EXCITED STATE OF A1

Prepared by taking 1.5 mL of stock solution A (see UV-vis absorption spectra section) in DCE: HFIP (7:3) and 1.5 mL of DCE: HFIP (7:3). Excitation at 420 nm. The fluorescence curve was normalized so that the maximum fluorescence (498 nm) equals the absorbance at 420 nm.

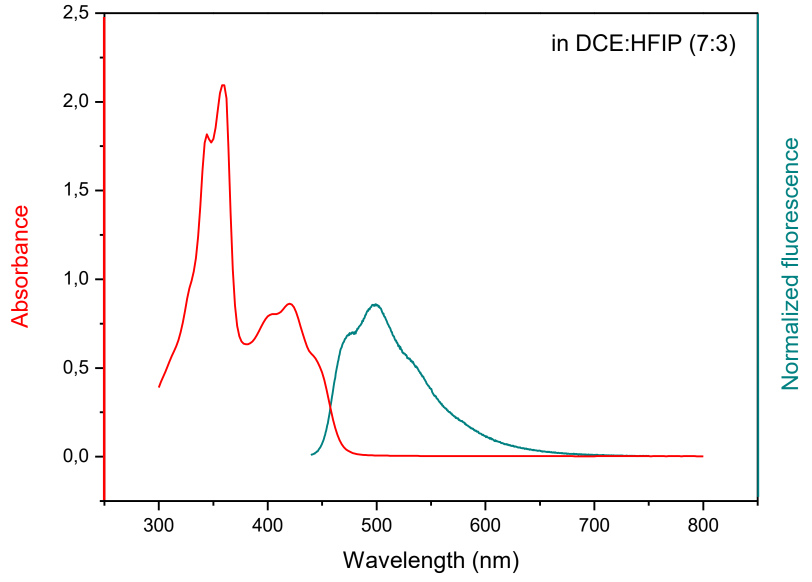

Figure S12: Energy of the wavelength at which UV-Vis absorption and emission spectra of **A1** overlap for the $E_{0,0}$ determination in DCE: HFIP (7:3).

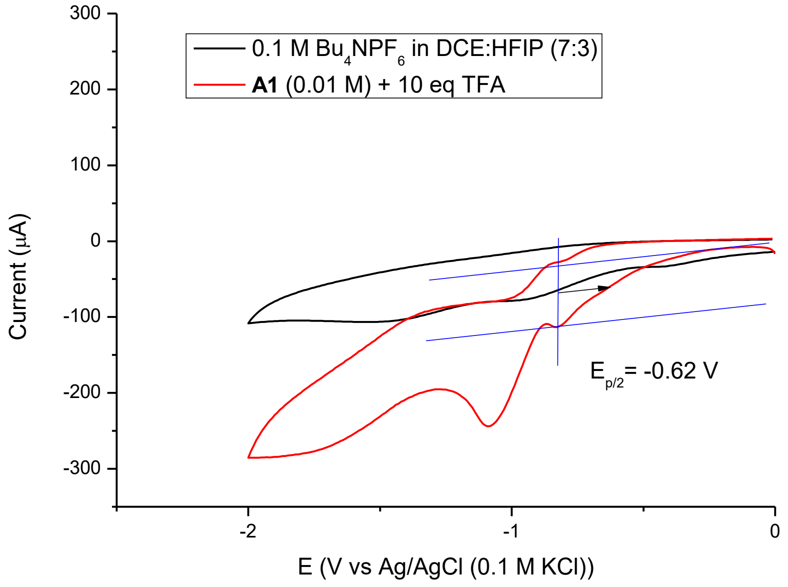

Figure S13: $E_{p/2}$ of **A1** after the addition of TFA in DCE: HFIP (7:3)

Prepared by taking 1.5 mL of stock solution A (see UV-vis absorption spectra section) in MeCN and 1.5 mL of **MeCN**. Excitation at 415 nm. The fluorescence curve was normalized so that the value of the maximum fluorescence (498 nm) is the same as the value of the absorbance at 415 nm.

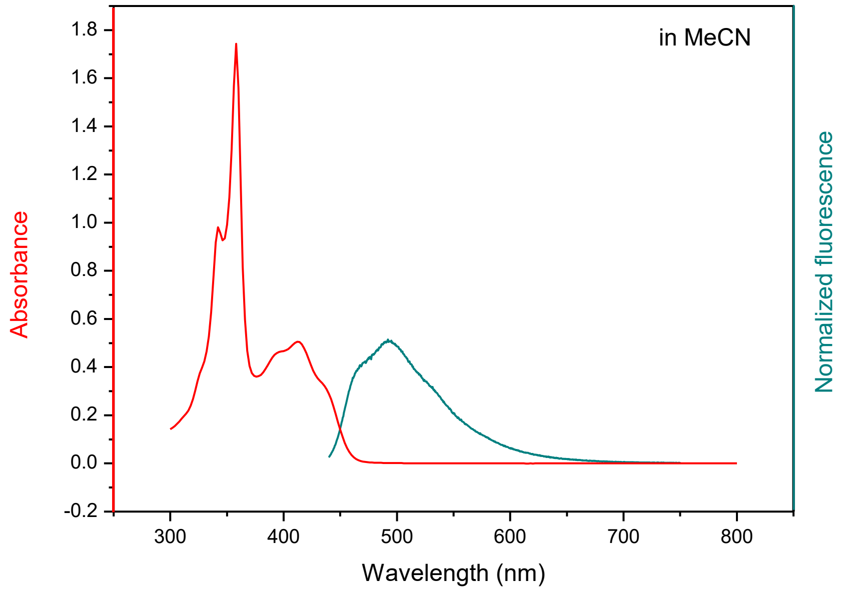

Figure S14: Energy of the wavelength at which UV-Vis absorption and emission spectra of **A1** overlap for the $E_{0,0}$ determination in MeCN.

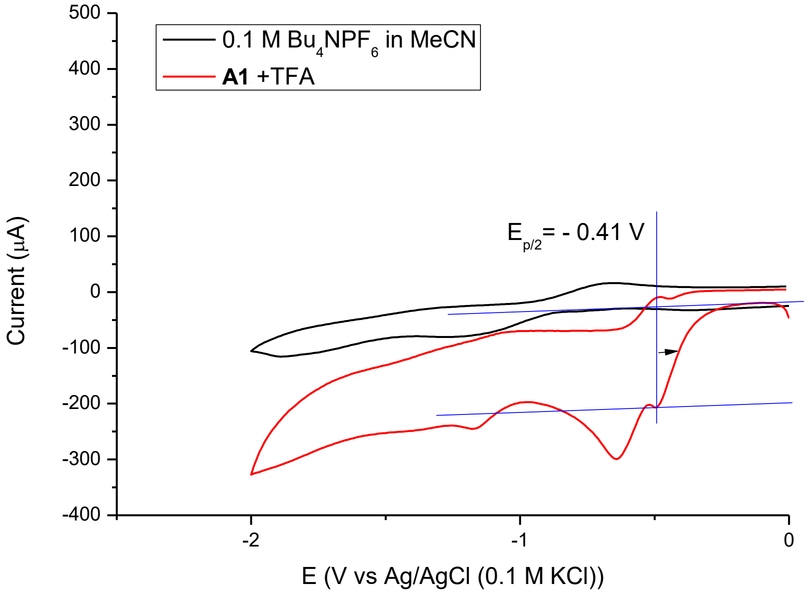

Figure S15: $E_{p/2}$ of **A1** after the addition of TFA in MeCN

Data from Figures S12 and S13 in DCE:HFIP (7:3):

Excitation wavelength: 420 nm

Fluorescence maximums 498 nm

Crossing wavelength: 458 nm → $E_{0,0}$ = 2.71 eV

From voltametric experiments: $E_{p/2}$ = -0.62 V vs Ag/AgCl (0.1 M)

Considering that E (Fc) *vs.* SCE is 0.38 V in MeCN and that our Fc measurement is 0.485 V vs Ag/AgCl (0.1 M) in MeCN and 0.375 V vs Ag/AgCl (0.1 M) in DCE: HFIP (7:3), that leaves a correction factor of (0.485 -0.375) + (0.485-0.38) = 0.215 V.

The correction of the ground state potential of **A1** would be:

$E_{p/2}$ (**A1**) = -0.410 - 0.215 = -0.625 V vs SCE (considering that E Fc vs SCE is 0.38 V)

Therefore:

$E^*_{p/2\ \text{in DCE/HFIP (7:3)}}$= 2.71 – 0.625 = 2.08 V (vs SCE with Fc correction)

Data from Figures S14 and S15 in MeCN:

Excitation wavelength: 415 nm

Fluorescence maximum: 492 nm

Crossing wavelength: 450 nm → $E_{0,0}$ = 2,76 eV

From voltametric experiments: $E_{p/2}$ = -0.410 V vs Ag/AgCl (0.1 M)

Considering that E (Fc) vs SCE is 0.38 V and that our Fc measurement is 0.485 V vs Ag/AgCl (0.1 M), that leaves a correction factor of -0.105 V.

The correction of the ground state potential of **A1** would be:

$E_{p/2}$ (**A1**) = -0.410 - 0.105 = -0.515 V vs SCE (considering that E Fc vs SCE is 0.38 V)

Therefore:

$E^*_{p/2\ \text{in MeCN}}$= 2.76 – 0.515 = 2.24 V (vs SCE with Fc correction)

Therefore, as a summary:

$E^*_{p/2\ \text{in MeCN}}$ = **2.24 V** vs SCE

$E^*_{p/2\ \text{in DCE/HFIP (7:3)}}$ = **2.08 V** vs SCE

QUENCHING EXPERIMENTS

The following experiments were performed exciting at 450 nm (slits 3, 0.05 s integration time).

1.5 mL of stock solution A was diluted to 3 mL with DCE: HFIP (7:3). Then, 4 solutions were prepared containing 1.5 mL of stock solution A, and 0, 200, 400 or 600 μL of stock solution B (0, 100, 200, 300 equiv), and diluting each solution up to 3 mL with DCE: HFIP (7:3). The fluorescence spectra were recorded:

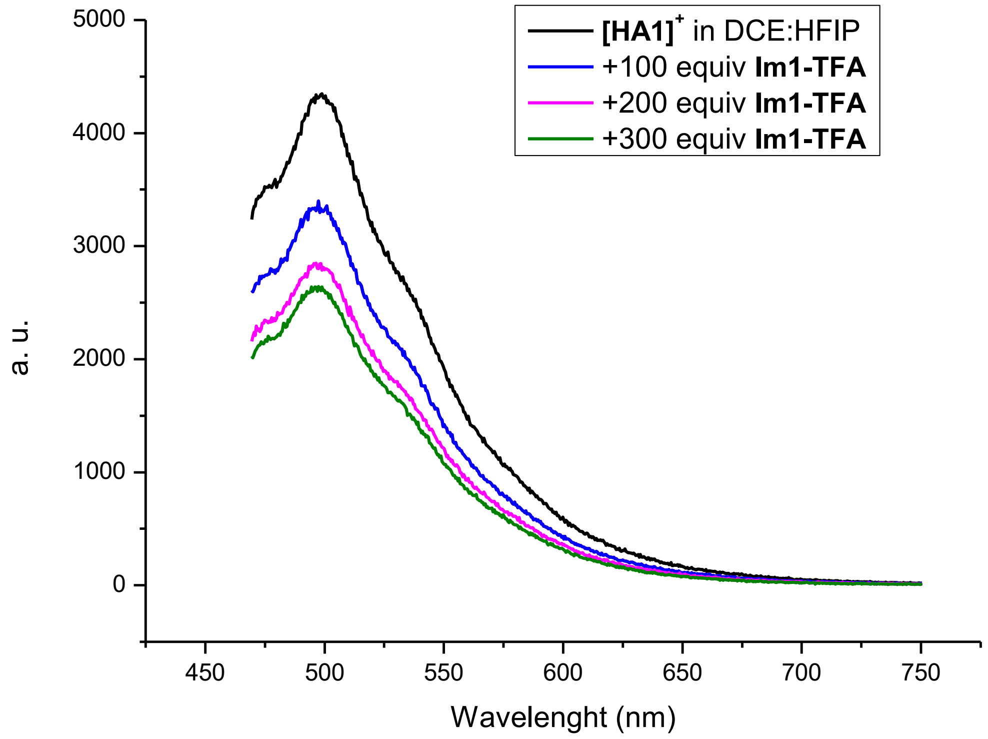

Figure S16: Fluorescence spectra of **A1**+TFA and quenching using different equivalents of **Im1-TFA** in DCE: HFIP (7:3).

The same measurements were performed using toluene instead of stock solution B. This way, 1.5 mL of stock solution A was diluted up to 3 mL with DCE: HFIP (7:3), followed by successive additions of 6.5 μL of pure toluene (0.06 mmol each addition, 100 equiv), making five measurements with 0, 100, 200, 300, and 400 equivalents of toluene with respect to **A1**. This data is shown:

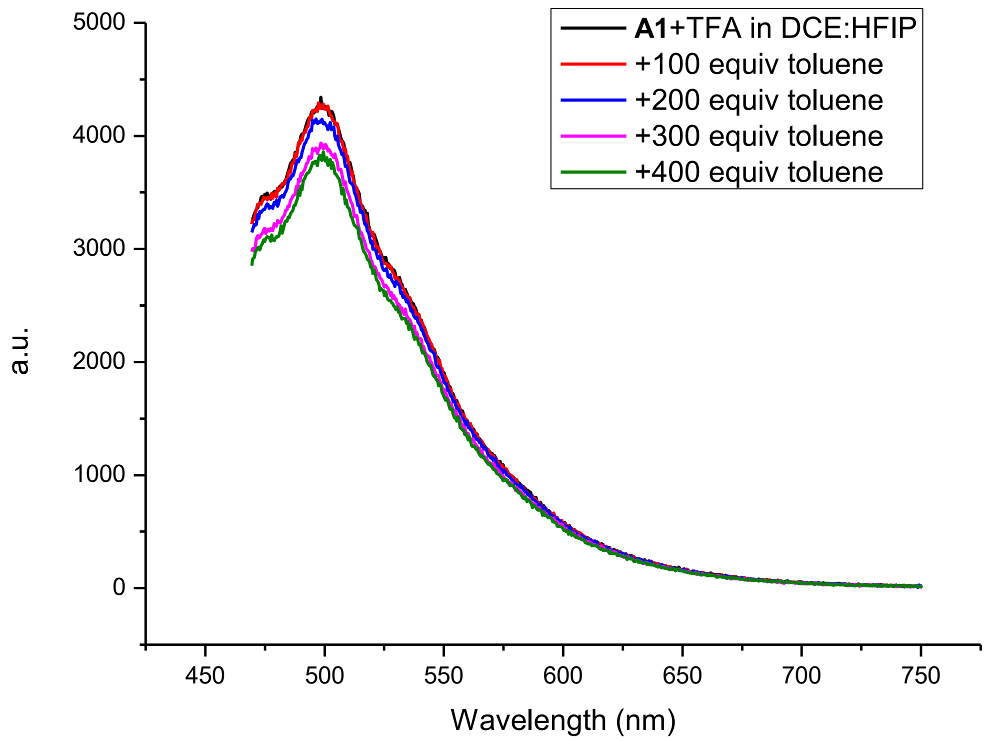

Figure S17: Fluorescence spectra of **A1**+TFA and quenching using different equivalents of toluene in DCE: HFIP (7:3).

1.5 mL of stock solution A were diluted up to 3 mL with DCE: HFIP (7:3). Then, a solution was prepared containing 1.5 mL of stock solution A, and 200 µL of stock solution B (100 equiv. of **Im1-TFA**), and diluting to 3 mL with DCE: HFIP (7:3). To this solution, 4 successive additions of 6.5 µL of toluene were added to obtain 100, 200, 300, 400 equivalents of toluene in solution. The fluorescence spectra of all solutions were recorded:

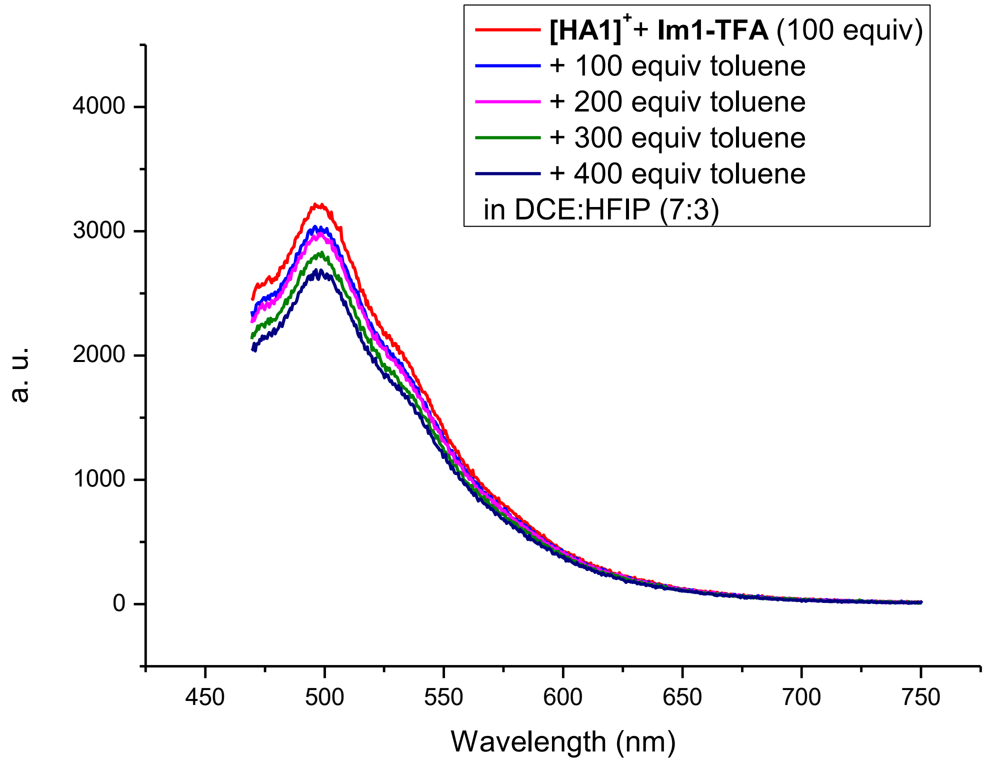

Figure S18: Fluorescence spectra of **A1**+TFA and **Im1-TFA**, and quenching using different equivalents of toluene in DCE: HFIP (7:3).

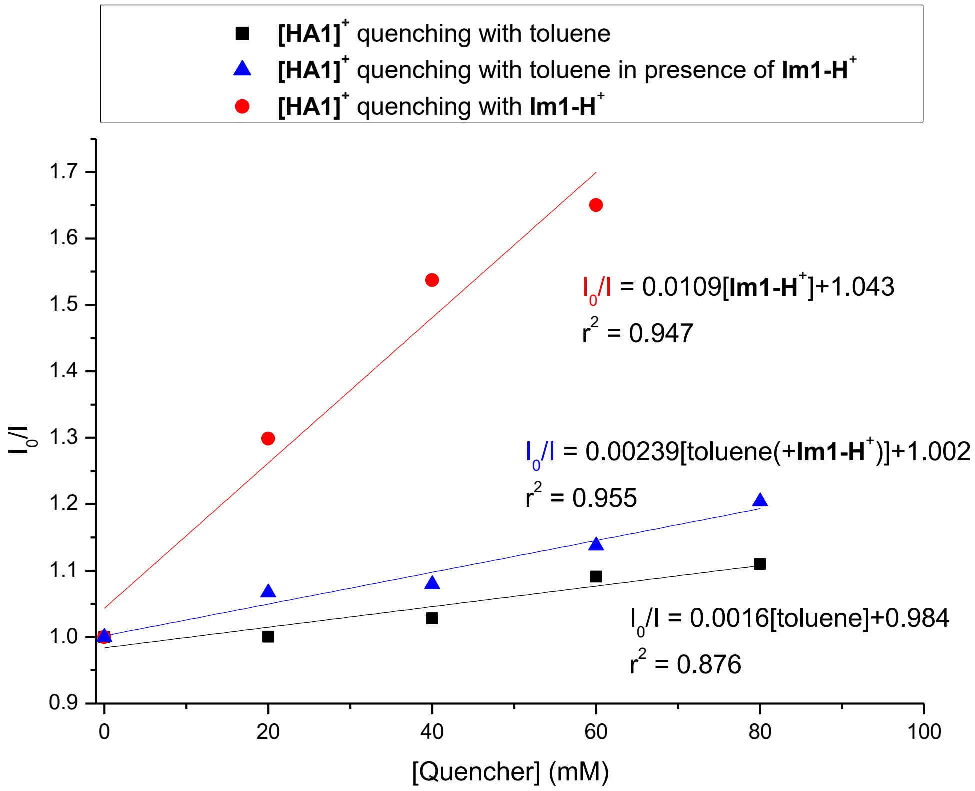

Figure S19: The Stern-Volmer plot of the quenching of **A1** with iminium **Im1-H**$^+$, toluene, and toluene in the presence of **Im1-H**$^+$. In DCE: HFIP (7:3).

CYCLIC VOLTAMMETRIES

Measurements were made using the EmStatblue+ potentiostat with a standard three-electrode setup: a 3 mm diameter vitreous carbon electrode as the working electrode, a platinum wire as the counter electrode, and an Ag/AgCl electrode (0.10 M aqueous KCl) as the reference electrode. The measurements were made on solutions of the substrates (0.1 mmol) in DCE: HFIP (7:3) or MeCN (3 mL each), using Bu_4NPF_6 as the supporting electrolyte (0.1 M). Solutions were bubbled with Ar for 5 min before each measurement. The scanning speed was 100 mV s^{-1}, and spectra were recorded from an initial potential of 0 V, scanning towards positive potentials to +2.5 V for the oxidation process and towards negative potentials to –2.5 V for the reduction process. The glassy carbon electrode was polished between every scan. Potentials were corrected vs. SCE using the correction factor obtained for A1, as described above. Data was analyzed using OriginLab software.

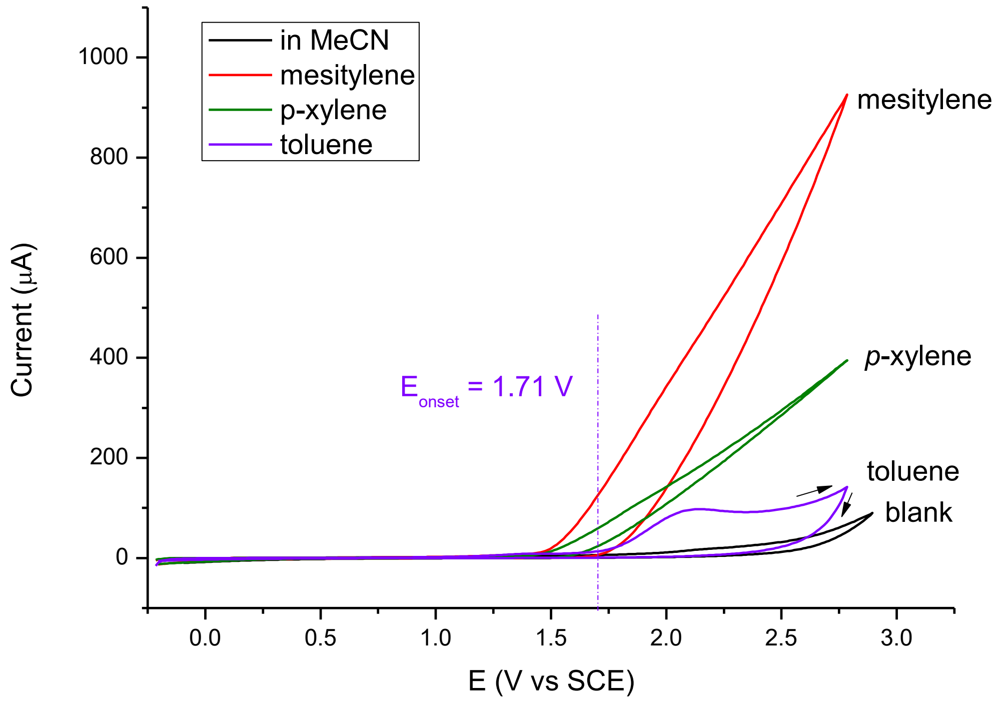

Figure S20: Cyclic voltammetries for toluene and toluene derivatives in MeCN.

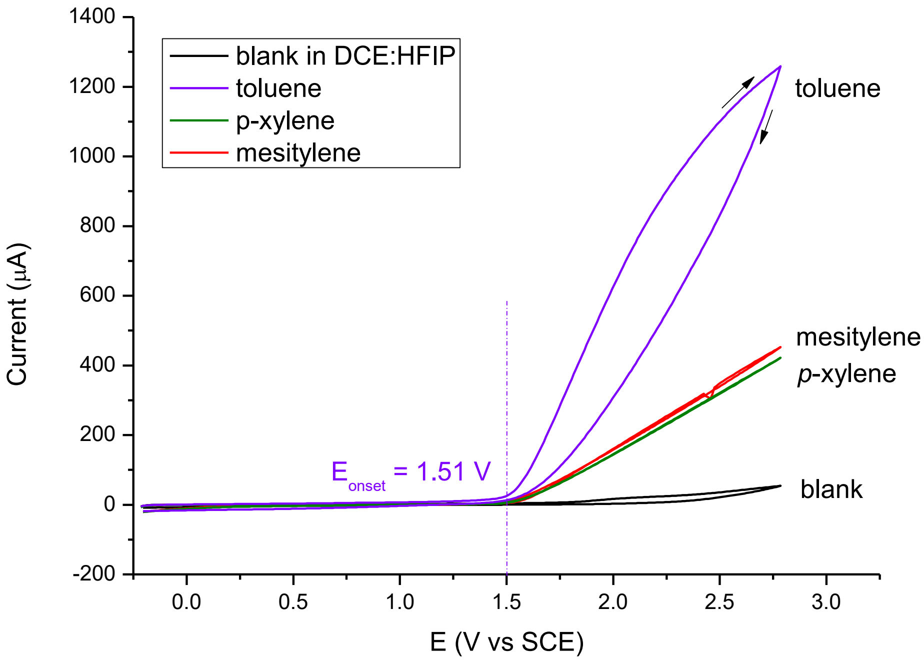

Figure S21: Cyclic voltammetries for toluene and toluene derivatives in DCE: HFIP (7:3).

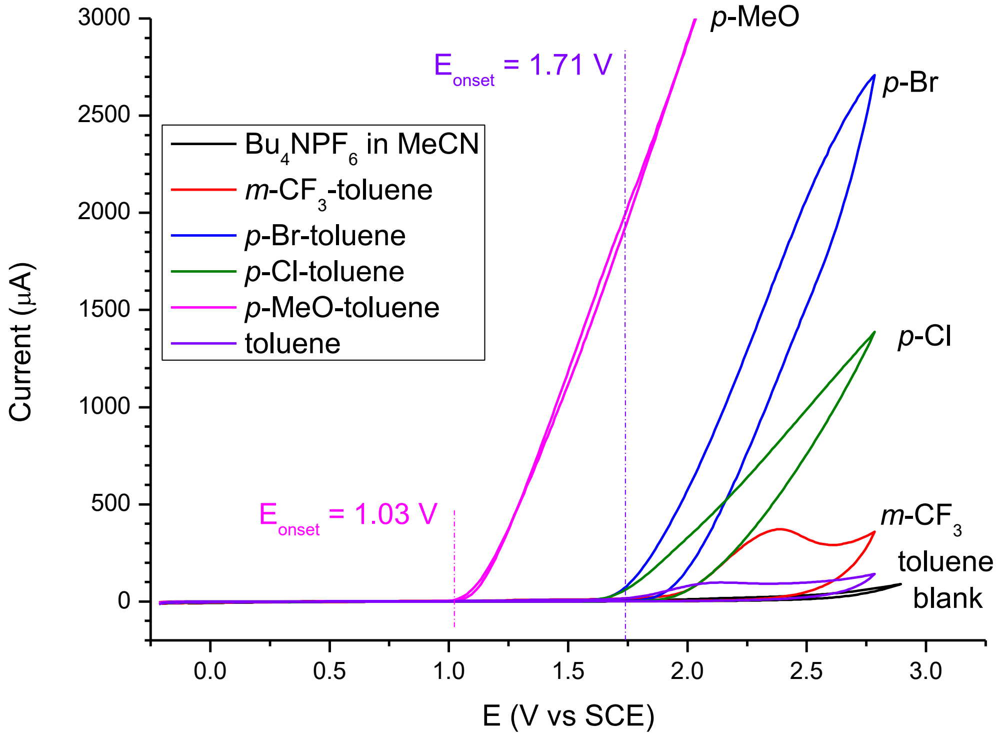

Figure S22: Cyclic voltammetries for toluene and toluene derivatives in MeCN.

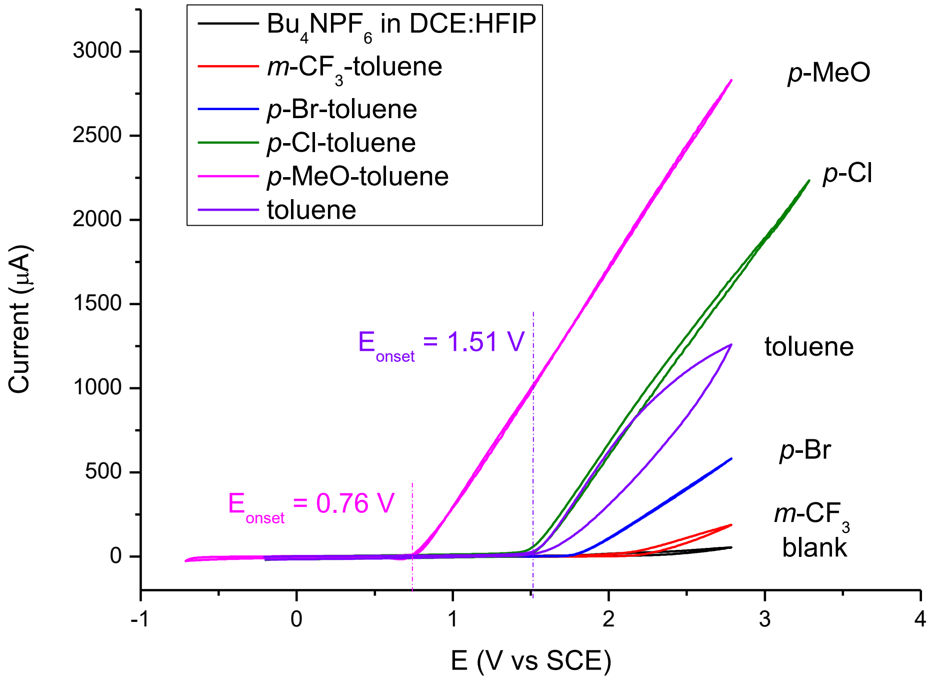

Figure S23: Cyclic voltammetries for toluene and toluene derivatives in DCE: HFIP (7:3).

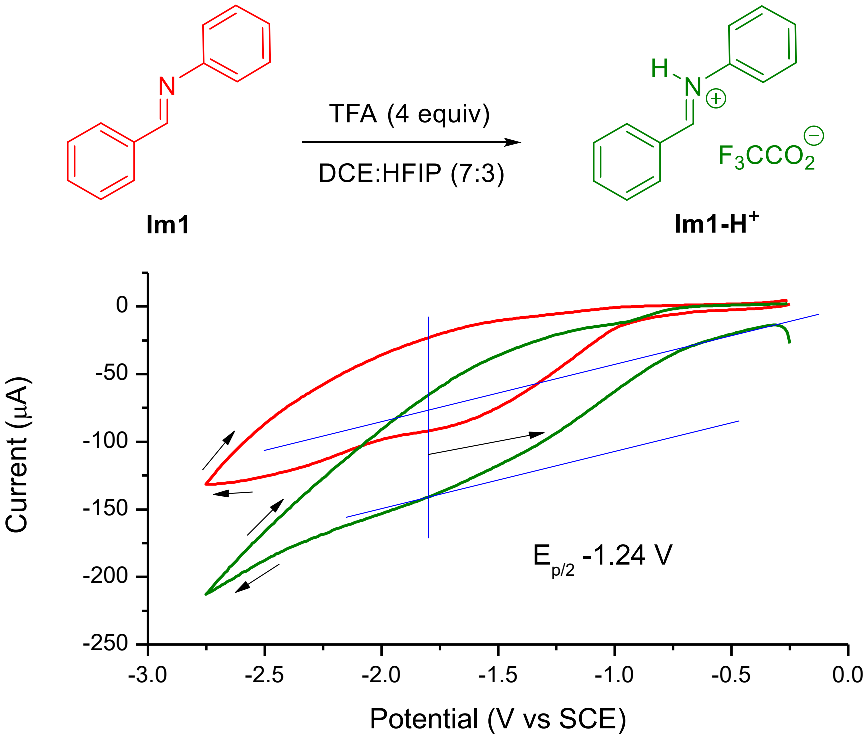

Figure S24: $E_{p/2}$ potential of iminium **Im1-H$^+$** in DCE: HFIP (7:3) after addition of 4 equivalents of TFA to **Im1** (0.1 mM).

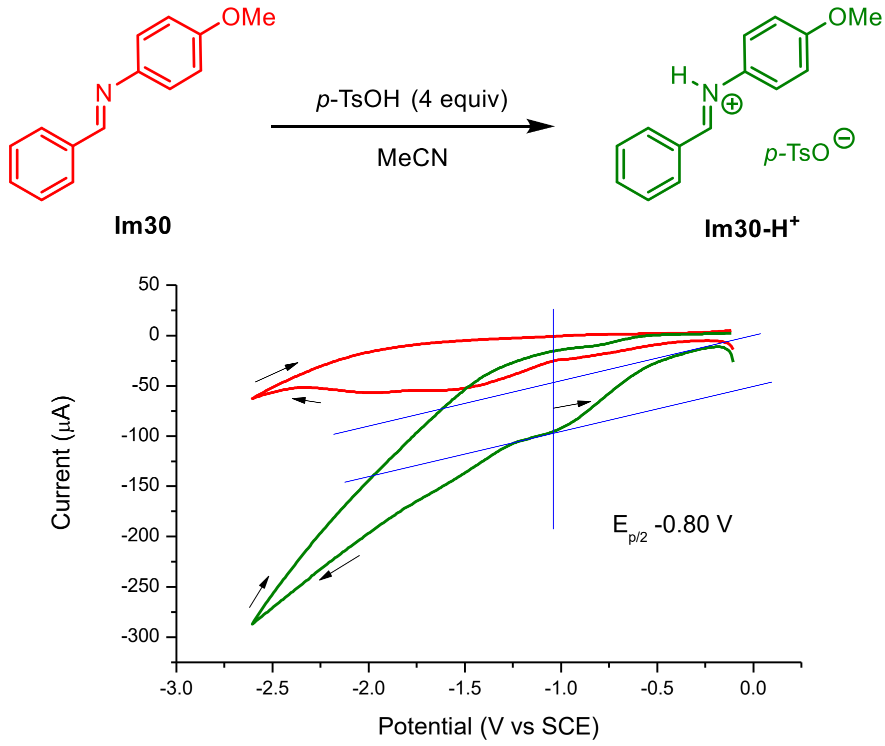

Figure S25: $E_{p/2}$ potential of iminium **Im30-H$^+$** in MeCN after addition of 4 equivalents of *p*-TsOH to **Im30** (0.1 mM).

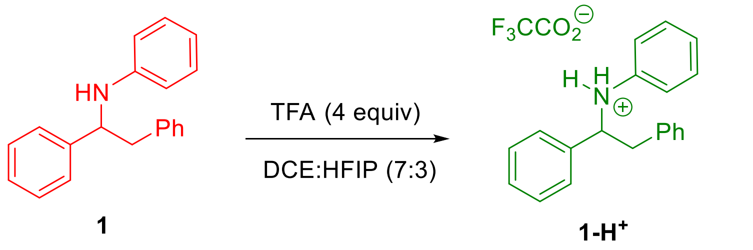

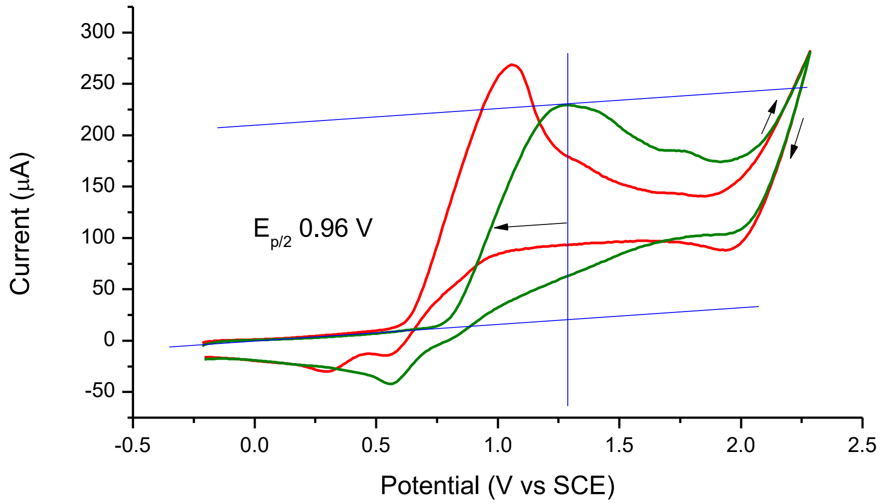

Figure S26: $E_{p/2}$ potential of iminium **1-H**$^+$ in DCE: HFIP (7:3) after addition of 4 equivalents of TFA to **Im1** (0.1 mM).

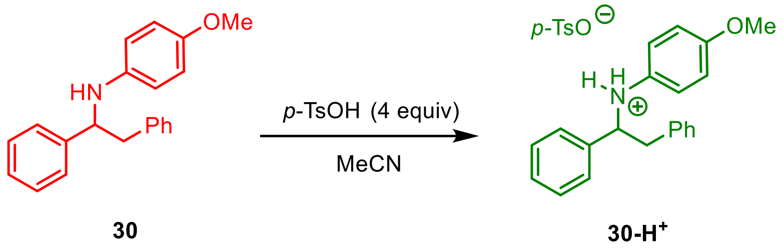

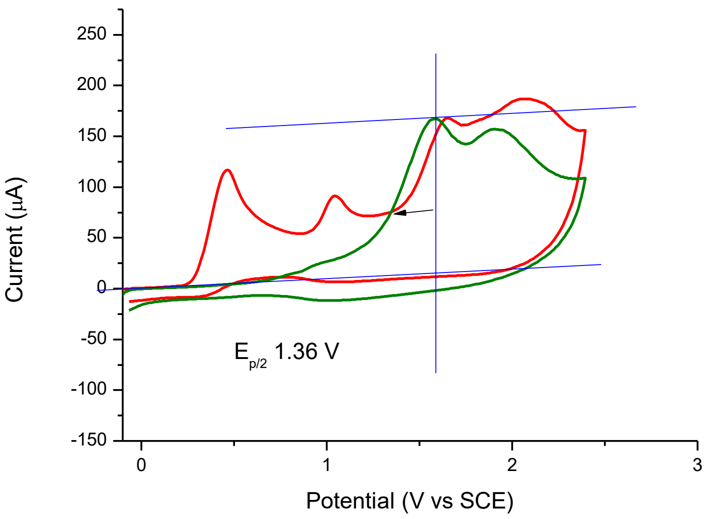

Figure S27: $E_{p/2}$ potential of **30-H**$^+$ in MeCN after addition of 4 equivalents of *p*-TsOH to **30** (0.1 mM).

QUANTUM YIELD MEASUREMENT

Determination of the photon flux:

The photon flux was measured for the conditions used in our photochemical setup using a two-dram vial in a PhotoRedOX Box Duo reactor (Evoluchem™) equipped with two 18 W EvoluChem lamps radiating at 450 nm. The photoreduction of Fe(III) to Fe(II) was monitored by determining the absorbance at 510 nm of the complex with 1,10-phenanthroline:

Actinometer solution (0.10 M): A 10 mL volumetric flask was charged with potassium ferrioxalate trihydrate (491,2 g, 1 mmol), distilled H_2O, and H_2SO_4 96% (0.2 mL, 3.7 mmol).

A buffered solution of phenanthroline (0.0055 M) was prepared in a 100 mL volumetric flask by dissolving phenanthroline (100 mg, 0.554 mmol), sodium acetate (11.25 g, 137 mmol), H_2SO_4 (96%, 1.3 mL, 25 mmol), and distilled water (pH 4).

A sample of the Actinometer solution (2 mL) is illuminated for a specific time (5 s, +5 s, +5 s). After each irradiation, an aliquot (50 μL) is transferred to a 10 mL volumetric flask, where 4 mL of phenanthroline solution is added, and the volume is raised to 10 mL. A blank sample was prepared using the same procedure, but keeping it in the dark.

To determine the fraction of light absorbed at 450 nm by the actinometer, a sample of this solution is measured at 450 nm (without dilution).

The absorbance of the solution was measured at 510 nm. A non-irradiated sample was also prepared, and the absorbance was measured at 510 nm. The formation of Fe(II) was calculated using Eq. 1.2.

- Quantum yield for the photoreduction of ferrioxalate at 450 nm is 1.1 mol× $einstein^{-1}$
- F is the fraction of light absorbed by the actinometer solution at 450 nm:

$$F = 1 - 10^{-A(450\,nm)}$$

- The reaction rate can be calculated with the data obtained at different irradiation times, after sequential transfer of samples

$$mol(Fe(II)) = \frac{\Delta A\ (510)}{\epsilon\ d}\ 2\ mL\ \frac{10\ mL}{0.05\ mL} 10^{\ -3} \qquad \boldsymbol{Eq\ 1.2}$$

$$mol(Fe(II)) = \frac{\Delta A\ (510)}{1.11x10^4\ M^{-1}cm^{-1}\ x\ 1cm}\ 2\ mL\ \frac{10\ mL}{0.05\ mL}\ 10^{\ -3} = 3.6036 \cdot 10^{-5} \cdot \Delta A\ (510) \qquad \boldsymbol{Eq\ 1.3}$$

$$Photon\ flux\ \left(\frac{einstein}{s}\right) = \frac{Reaction\ rate\ (\frac{mol}{s})}{Quantum\ yield\ (\frac{mol}{einstein}) \times F} \qquad \boldsymbol{Eq. 2.1}$$

$$Photon\ flux\ \left(\frac{einstein}{s}\right) = \frac{1.444 \cdot 10^{-6}\ (\frac{mol}{s})}{1.1\ (\frac{mol}{einstein}) \times 0.9858} = 1.3300 \cdot 10^{-6}\ Einstein \cdot s^{-1} \qquad \boldsymbol{Eq. 2.2}$$

Determination of the quantum yield of the reaction:

The quantum yield was calculated as follows:

$$\emptyset_{450\ nm}(\%) = \frac{mol\ of\ \mathbf{1}}{t\ (s) \times photon\ flux\ (einstein \bullet s^{-1}) \times F} \times 100 \qquad \textbf{\textit{Eq}. 3.1}$$

Where mol of product **1** represents the amount of product generated after the irradiation time (*t* in seconds). The fraction of light absorbed (F) by the protonated photocatalyst at 450 nm was 0.992334. The photon flux previously calculated in **Eq. 2.2** was $1.3300 \cdot 10^{-6}\ Einstein \cdot s^{-1}$. Thus, after three measurements, the data obtained were:

Table S6: Quantum yield data obtained.

Run	Irradiation time (s)	Mol of product	% Quantum yield
1	600	0.000048	6.05
2	1200	0.000060	3.78
3	1800	0.000111	4.67

$$\emptyset_{450\ nm}(\%) = \mathbf{4.83\ \%} \qquad \textbf{\textit{Eq}. 3.2}$$

COMPUTATIONAL DETAILS

Simulations were performed at the Density Functional Theory (DFT) level with the M06-2X density functional[4] as implemented in Gaussian 16.[5] N, C, and H atoms were described with the basis set def2-TZVP[6] and O and F atoms with def2-TZVPD.[7] Geometries were optimized in the gas phase, and solvation effects (dichloroethane) were included via single-point calculations using the SMD method.[8] Intermediates and transition states were confirmed to have none and one imaginary frequency, respectively. Frequencies below 50 cm^{-1} were shifted to 50 cm^{-1} when computing vibrational partition functions[9] with GoodVibes.[10] The computation of Gibbs energy barriers for single-electron transfer processes were estimated using Marcus theory[11,12] and following procedures previously described in literature.[13] All reported energies correspond with Gibbs energies in solution at 298 K and 1 M.

The computation of pK_a values was carried out using a discrete–continuum method,[14] which included ten explicit water molecules.[15] For this section, additional single-point calculations were performed with the larger basis set def2-QZVPD.[16]

Energies and geometries are fully available at the ioChem-BD open-access platform[17] through the following database: DOI 10.19061/iochem-bd-6-597 (https://iochem-bd.bsc.es/browse/handle/100/479621).

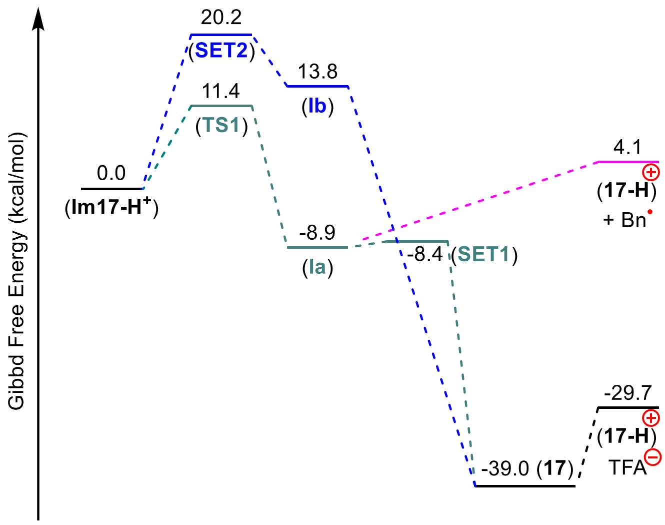

Figure S28: Summary of DFT calculations performed for compound **17** (from pivaldehyde)

A summary of the DFT calculations performed for the addition of the benzyl radical obtained from indan to **Im1**-H^+ is presented in Figure S28. Three conformers of the transition state leading to the *anti*-**6** diastereoisomer were found, and another three conformers leading to the *syn*-**6** diastereoisomer. Overall, *syn*-**6** is slightly favored; however, the energy differences are so small that they fall within the error of these calculations. Considering the contributions of each transition state based on their energies, the theoretical *syn/anti* ratio is about 57:43, whereas in practice it is 60:40. As mentioned before, the error of these calculations is considerable. However, a low diastereoselectivity is expected for product **6**, and presumably for the addition of other prochiral benzylic precursors.

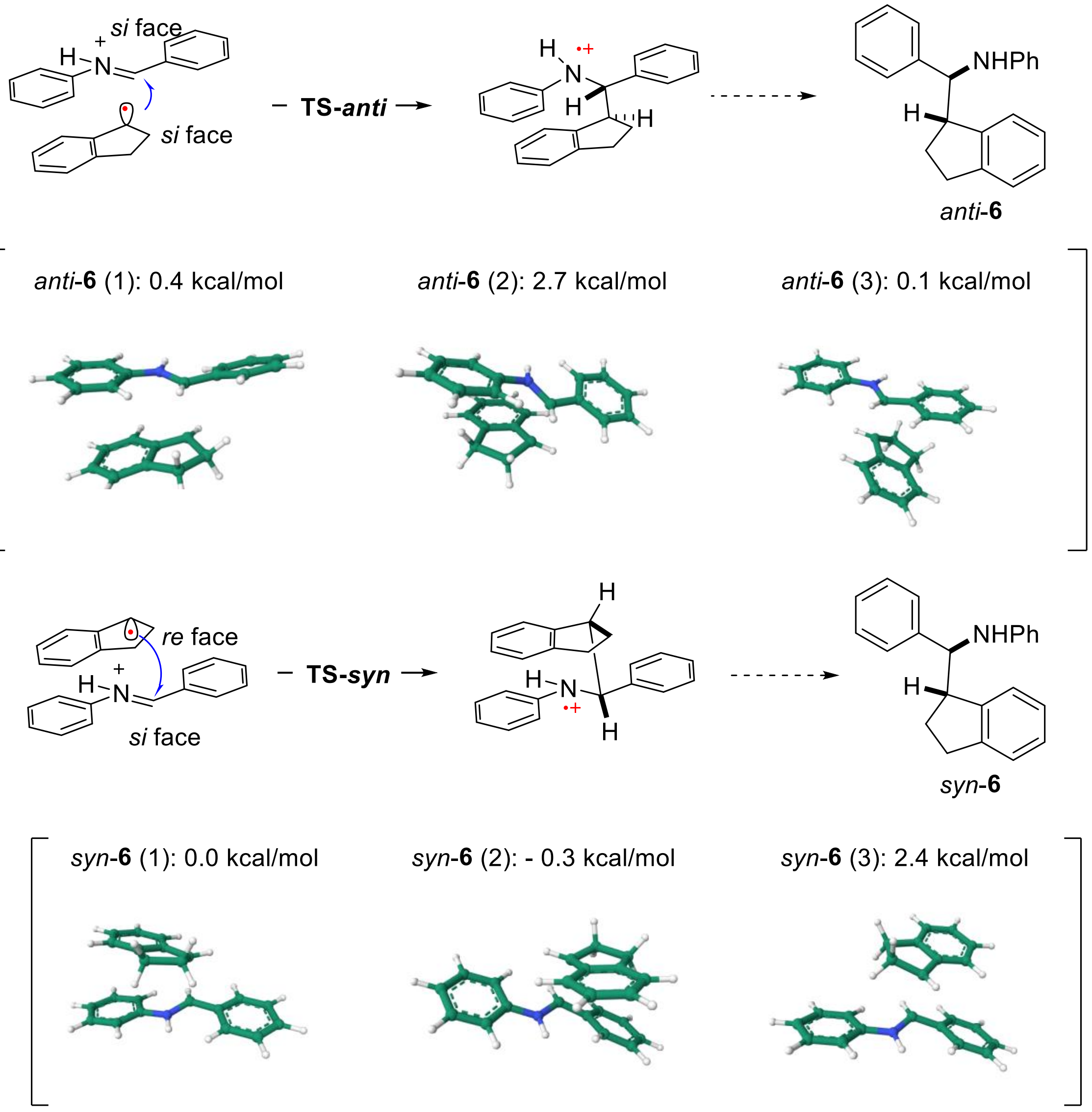

Figure S29: Summary of DFT calculations for the stereodetermining step of product **6**. The ΔΔG for each TS was calculated using *syn*-**6** (1) as an arbitrary reference (0.0 kcal/mol).

OTHER MECHANISTIC STUDIES

RADICAL TRAPS

TEMPO addition

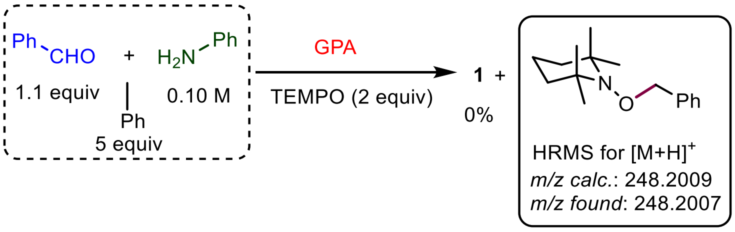

The general procedure A was followed, but TEMPO (94 mg, 0.6 mmol, 2 equiv) was added to the reaction mixture. Once the time elapsed, the reaction mixture was directly analyzed by HPLC-MS (ESI), revealing no formation of product **1**, but only the adduct of the bencyl radical with TEMPO.

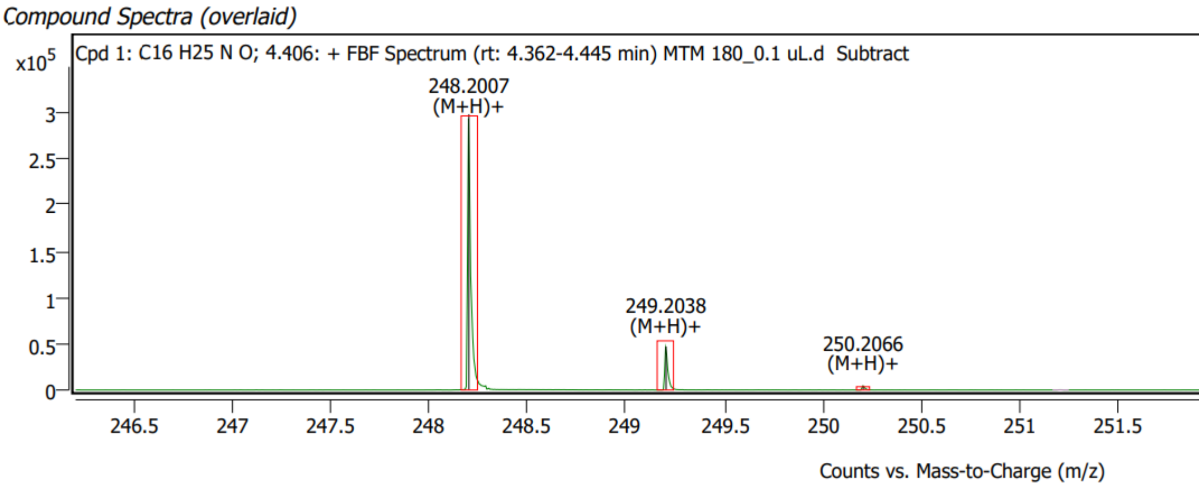

Figure S30: MS for the TEMPO-Tolyl radical adduct.

1,1-Diphenylethylene addition

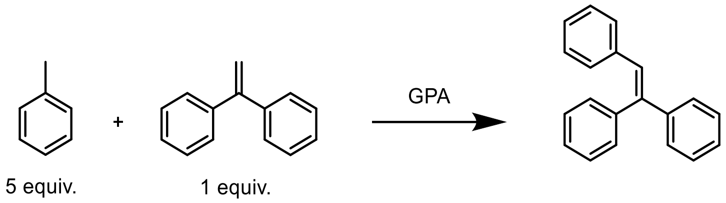

In a two-dram vial equipped with a magnetic stirring bar, 1,1-diphenylethylene (0.30 mmol, 54 μL) and toluene (5 equiv, 1.50 mmol, 158 μL) were added, followed by 9-(2-chlorophenyl)acridine (**A1**, 2.5 mol%, 2.2 mg). A mixture of 1,2-dichloroethane (DCE, 2.1 mL) and 1,1,1,3,3,3-hexafluoro-2-propanol (HFIP, 0.9 mL) was then added to the vial, followed by trifluoroacetic acid (TFA, 1.1 equiv, 0.33 mmol, 26 μL). The vial was sealed and placed in the PhotoRedOx Box Duo photoreactor. The reaction mixture was irradiated with blue LEDs (λ = 450 nm) for 16 hours at room temperature (approximately 25–30 °C, controlled by a fan). After completion, the reaction mixture was concentrated under reduced pressure, and the resulting residue was dissolved in ethyl acetate (EtOAc). The mixture was then quenched by adding K_2CO_3 (approximately 40 mg), stirred for 30 minutes, and filtered. The crude mixture was concentrated under reduced pressure. The resulting residue was purified by FC using a gradient from 0% to 2% EtOAc in n-hexane as the eluent, affording the desired product and other inseparable byproducts. The product was analyzed by ^{1}H-NMR using an internal standard to quantify the exact yield (62%).

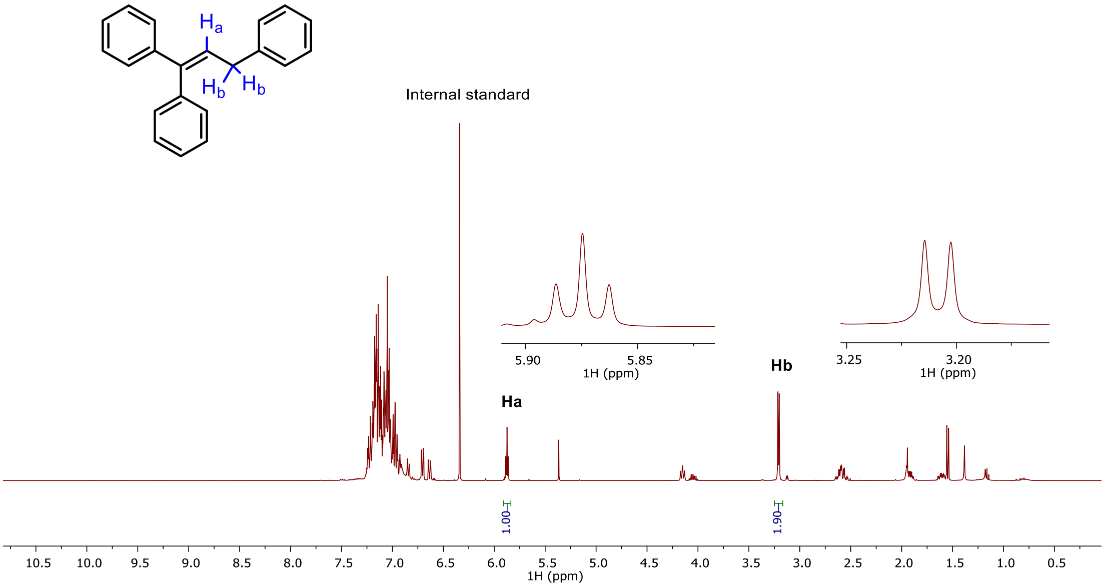

Figure S31: ^{1}H-NMR of the 1,1,2-Triphenylethene with the internal standard.

DETERMINATION of DIASTEREOMERIC RATIO FOR PRODUCTS **5-9** AND **33**

Product 5

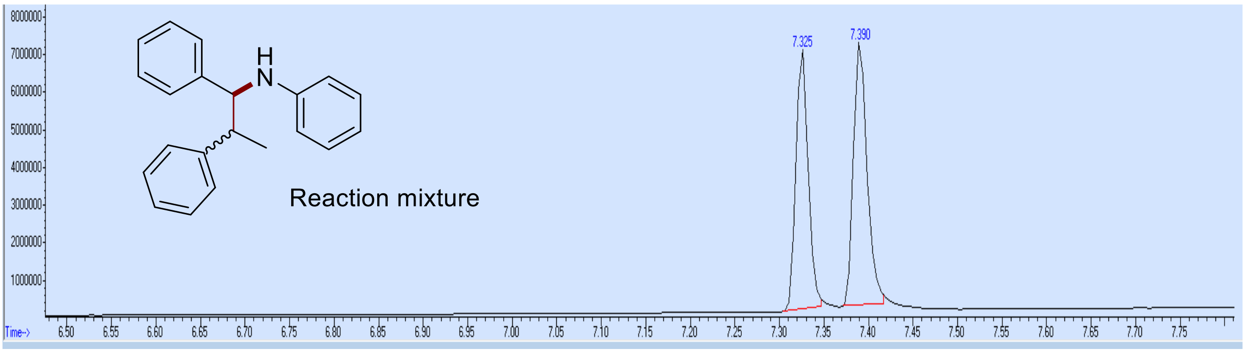

peak #	R.T. min	first scan	max scan	last scan	PK TY	peak height	corr. area	corr. % max.	% of total
1	7.325	813	817	820	M	6874981	61378149	87.23%	46.590%
2	7.390	826	829	833	M	6986149	70363410	100.00%	53.410%

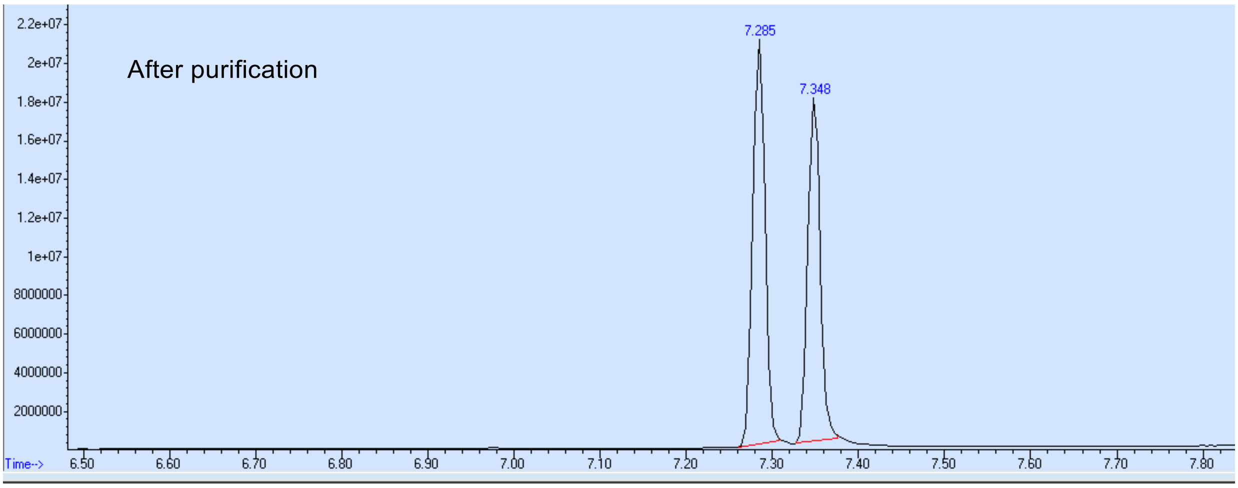

peak #	R.T. min	first scan	max scan	last scan	PK TY	peak height	corr. area	corr. % max.	% of total
1	7.285	804	809	813	M	20987323	203477256	100.00%	52.720%
2	7.348	817	821	826	M	17889342	182480335	89.68%	47.280%

Product 6

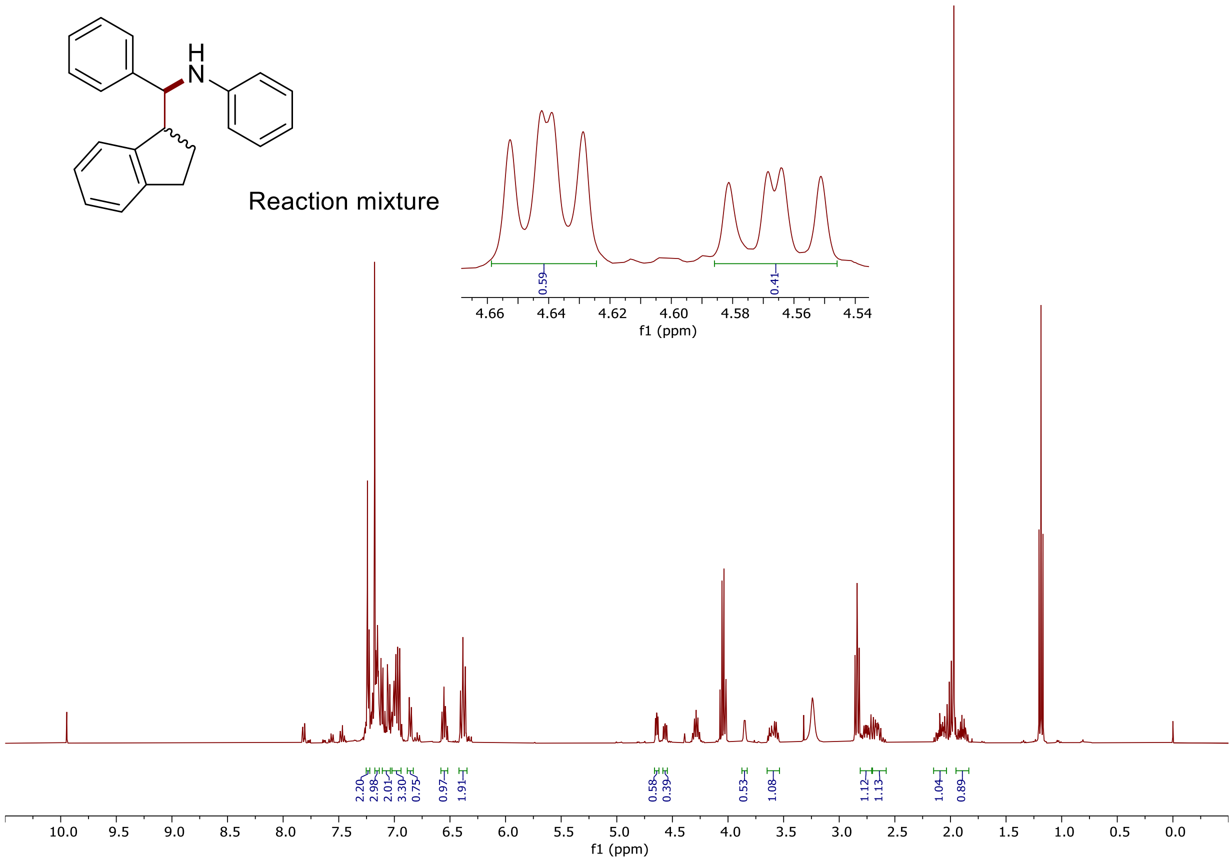

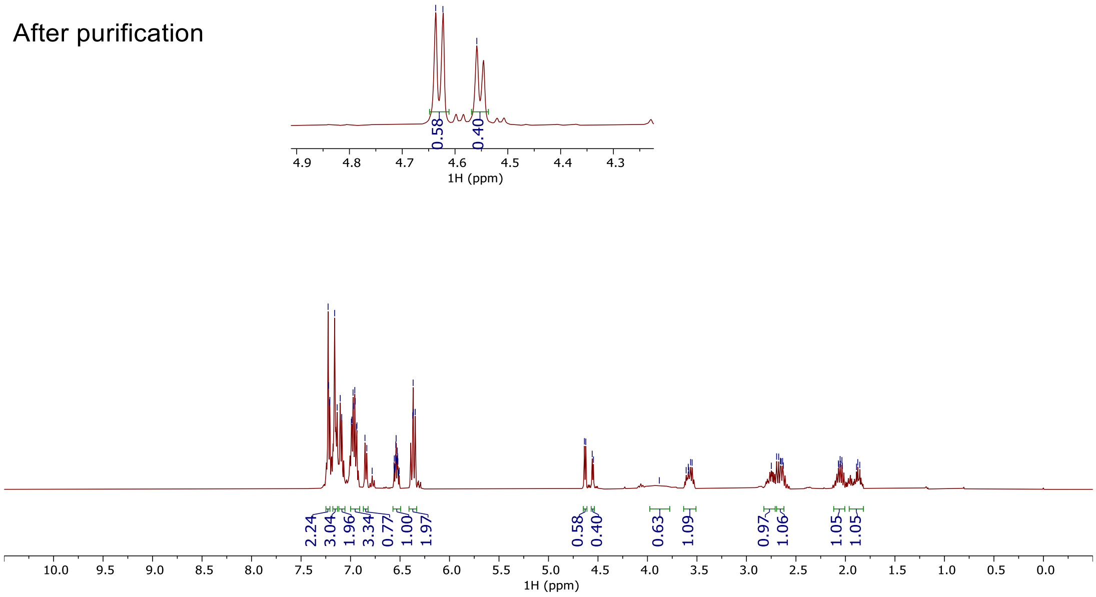

Product 7

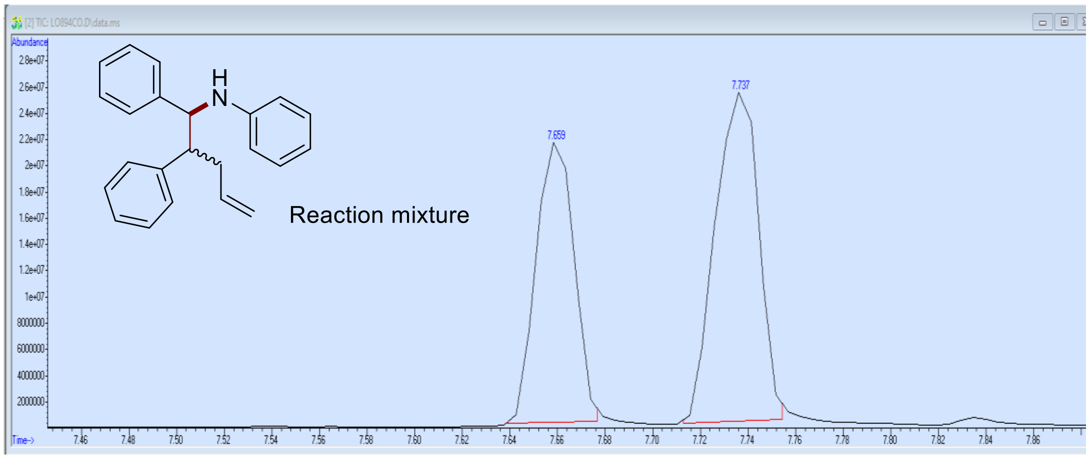

peak #	R.T. min	first scan	max scan	last scan	PK TY	peak height	corr. area	corr. % max.	% of total
1	7.659	877	881	885	M	21539821	237130982	74.23%	42.606%
2	7.737	892	896	900	M2	25061432	319433645	100.00%	57.394%

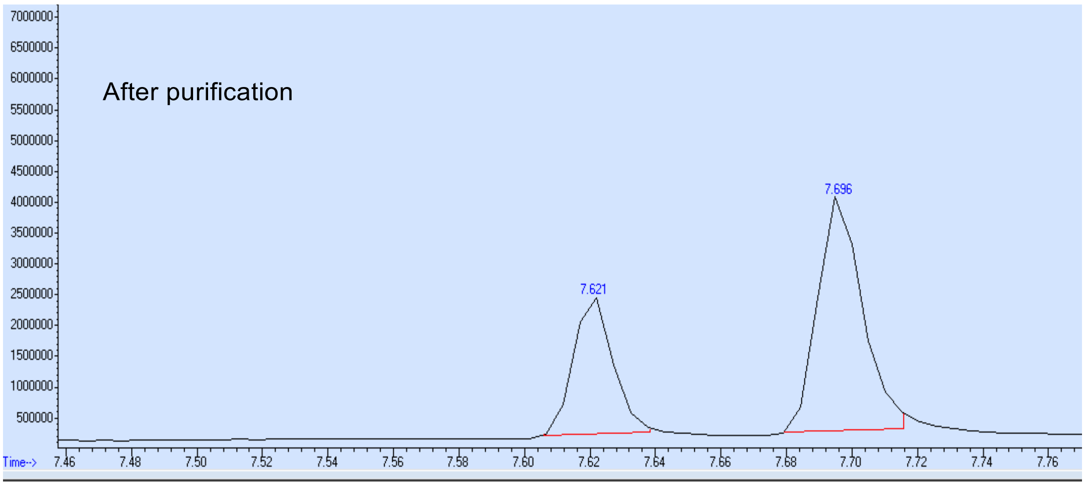

peak #	R.T. min	first scan	max scan	last scan	PK TY	peak height	corr. area	corr. % max.	% of total
1	7.621	871	874	877	M	2253158	18774790	52.34%	34.357%
2	7.696	885	888	892	M	3829737	35871054	100.00%	65.643%

Product 8

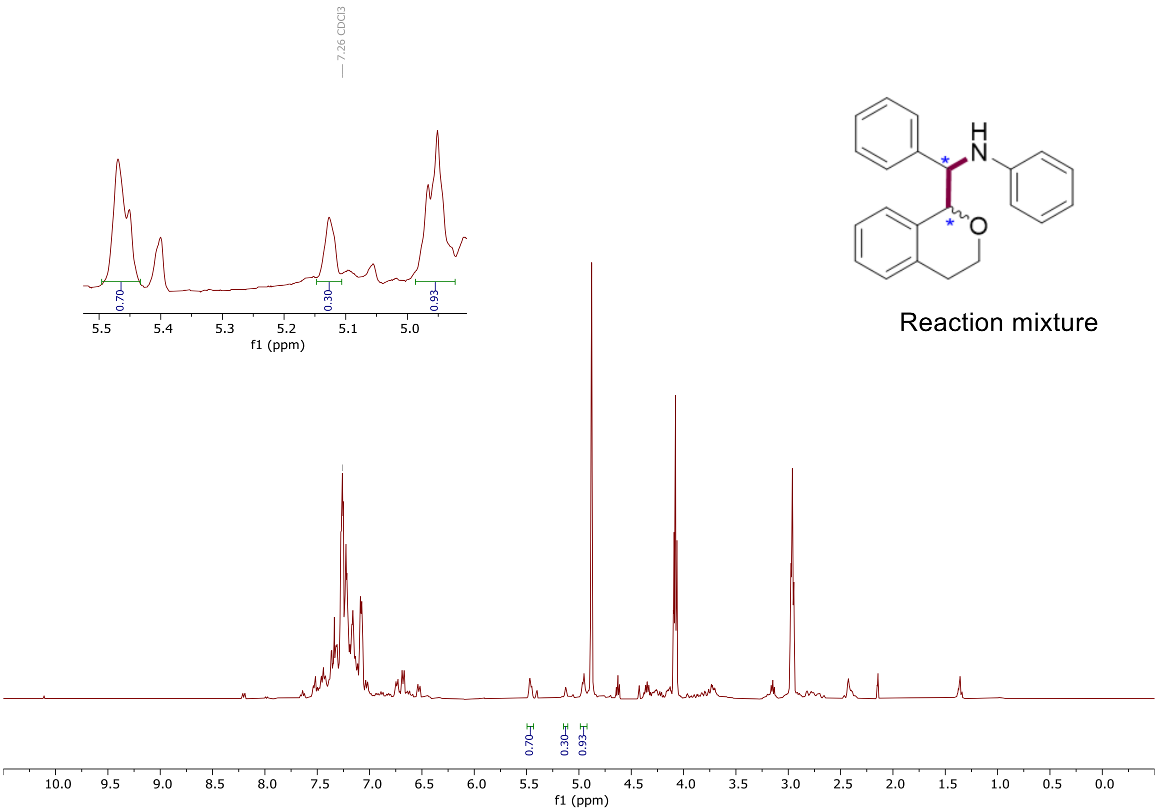

After purification

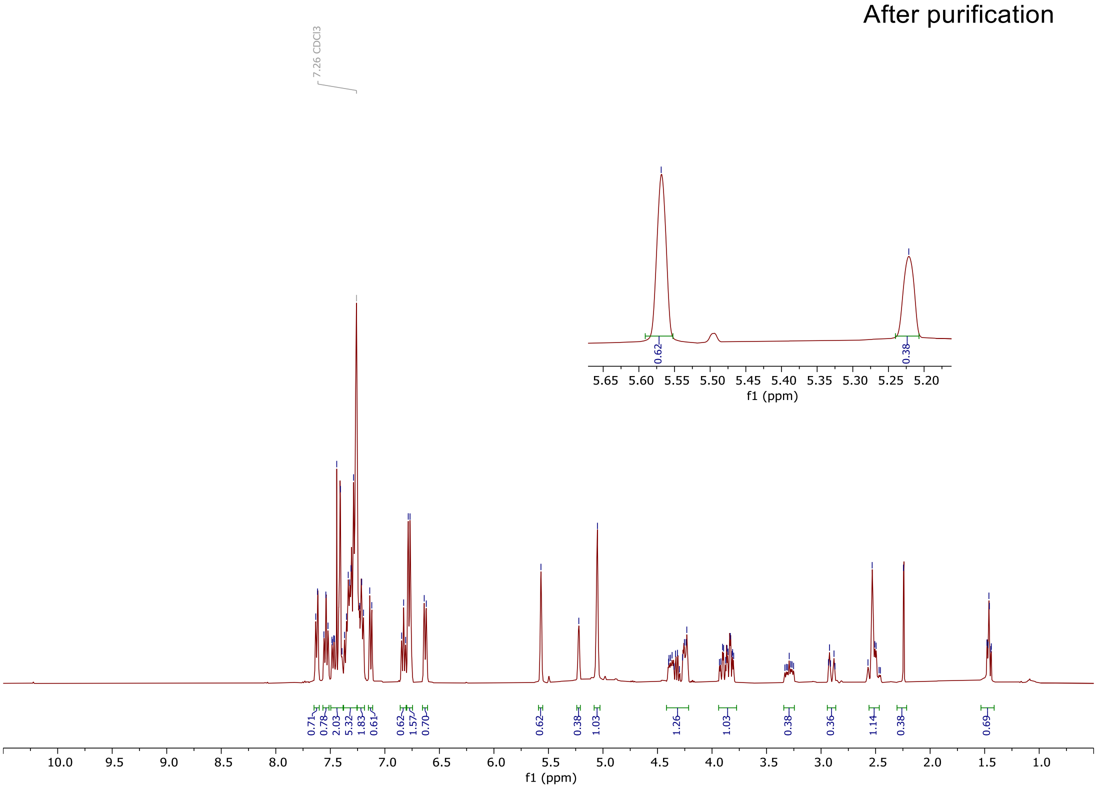

Product 9

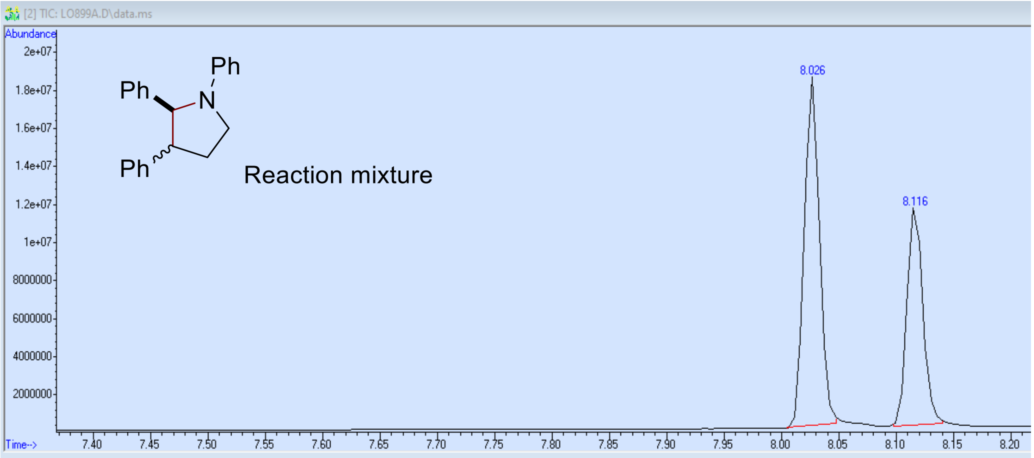

peak #	R.T. min	first scan	max scan	last scan	PK TY	peak height	corr. area	corr. % max.	% of total
1	8.026	948	952	956	M	18308808	171483300	100.00%	60.811%
2	8.116	966	969	974	M	11537132	110511279	64.44%	39.189%

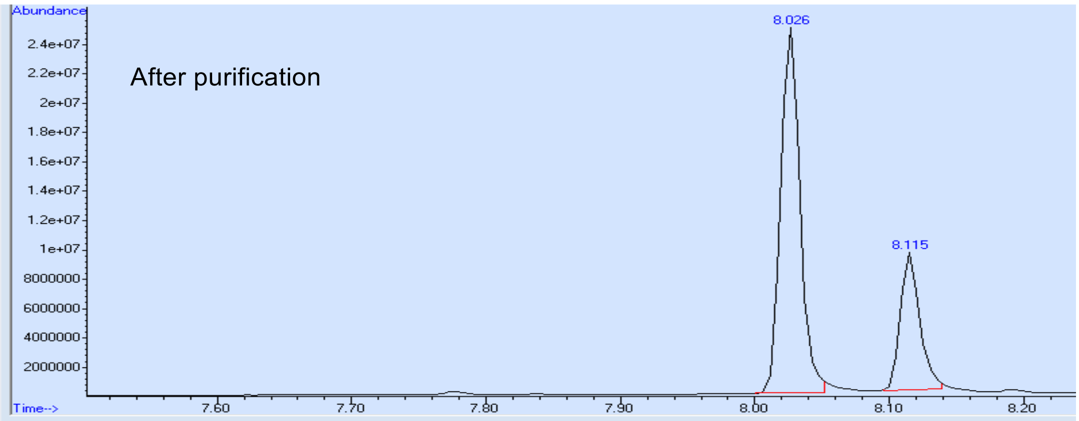

peak #	R.T. min	first scan	max scan	last scan	PK TY	peak height	corr. area	corr. % max.	% of total
1	8.026	947	952	957	M	24939598	253598982	100.00%	72.774%
2	8.115	965	969	974	M	9395917	94875219	37.41%	27.226%

Product 33

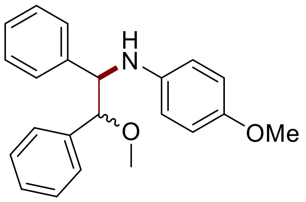

Reaction mixture

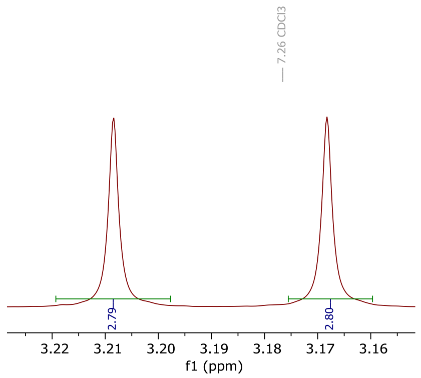

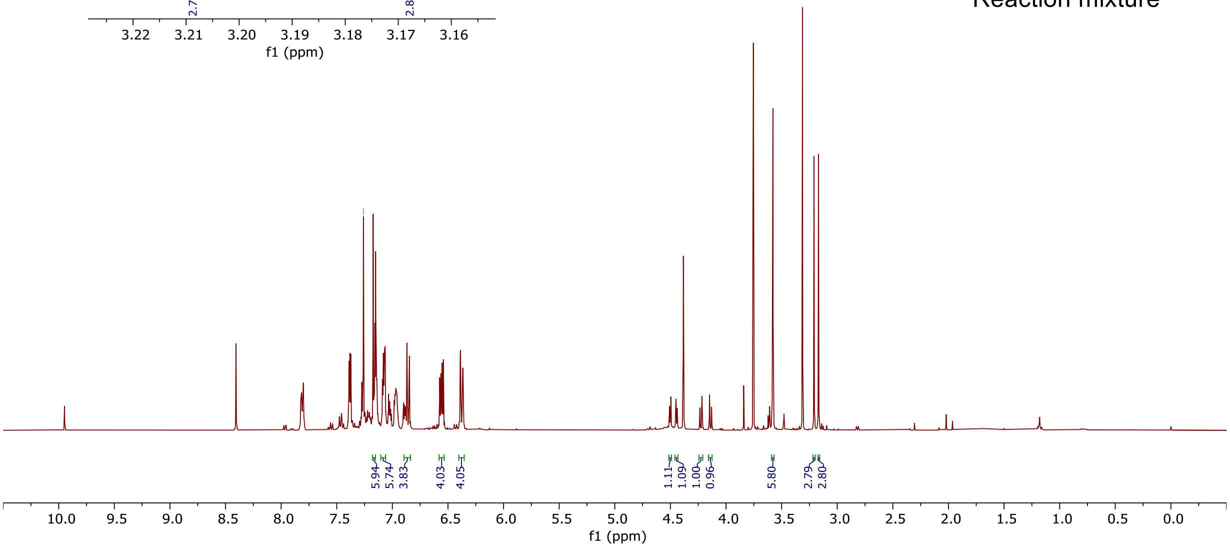

After purification

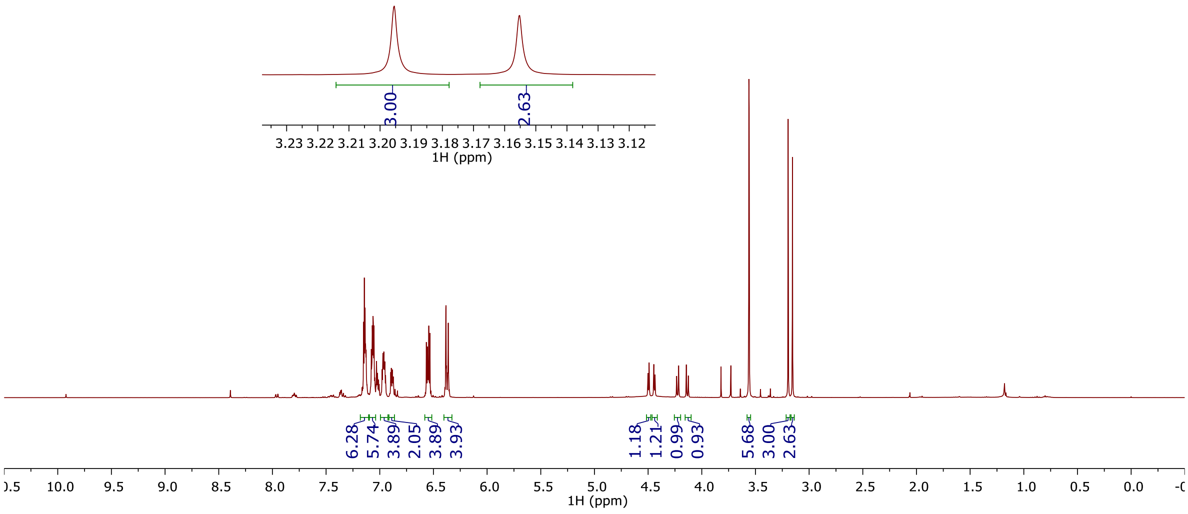

ANALYSIS OF UNDISTILLED ANILINE

A solution of undistilled aniline (0.30 mmol) and TFA (0.30 mmol) in 7:3 DCE/HFIP (3.0 mL) was irradiated with blue light (450 nm) without deoxygenation. Pictures at different times are shown below:

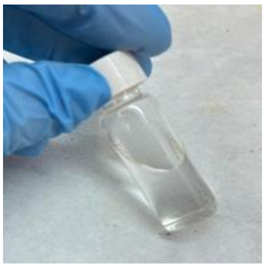

Before irradiation

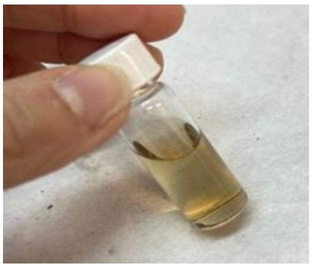

After 30 min

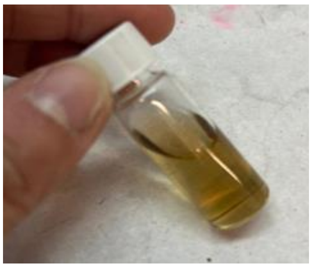

After 2 h

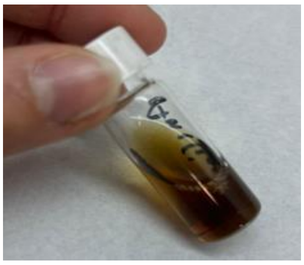

After 16 h

The same experiment was run, but after purging the solution with Ar for 5 min. Pictures at different times are shown below:

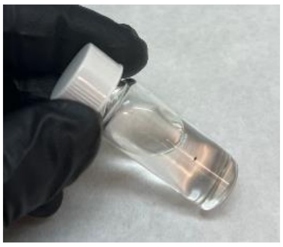

Before irradiation

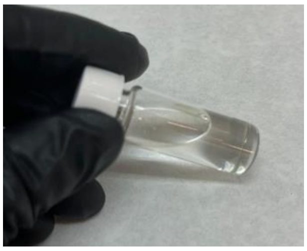

After 30 min

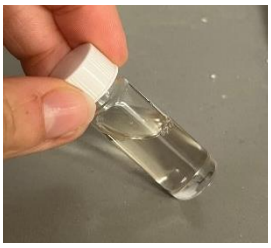

After 2 h

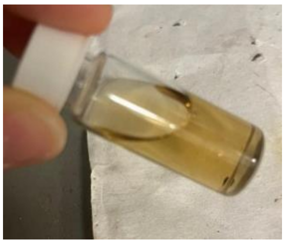

After 16 h

As shown before, the aniline solution in the presence of TFA becomes brown upon irradiation, which is much more pronounced in the presence of aerobic O_2. This oxidative, light-promoted degradation of aniline (likely a polymerization) might be catalyzed by impurities in undistilled aniline.

A sample of undistilled aniline (0.40 mL) was mixed with concentrated HNO_3 (4 mL), and the resulting mixture was diluted with 15 mL of distilled water before analysis by ICP. The results shown below indicate the presence of traces of Cu, Fe and Ni, which might catalyze the oxidative degradation of undistilled aniline under the reaction conditions.

Table S6: ICP-MS analysis of undistilled aniline.

Target	56Fe [He] [ppb]	60Ni [He] [ppb]	63Cu [He] [ppb]	105Pd [He] [ppb]	118Sn [He] [ppb]
Background*	<0.821	<0.098	<0.364	<0.004	0.122562065
Sample	**29.576**	**14.997**	**83.514**	0.036	12.279

*Background solution with 2% HNO_3 suprapur, 2% HCl suprapur y H_2O MilliQ.

UNSUCCESSFUL SUBSTRATES USING GENERAL PROCEDURES A AND B.

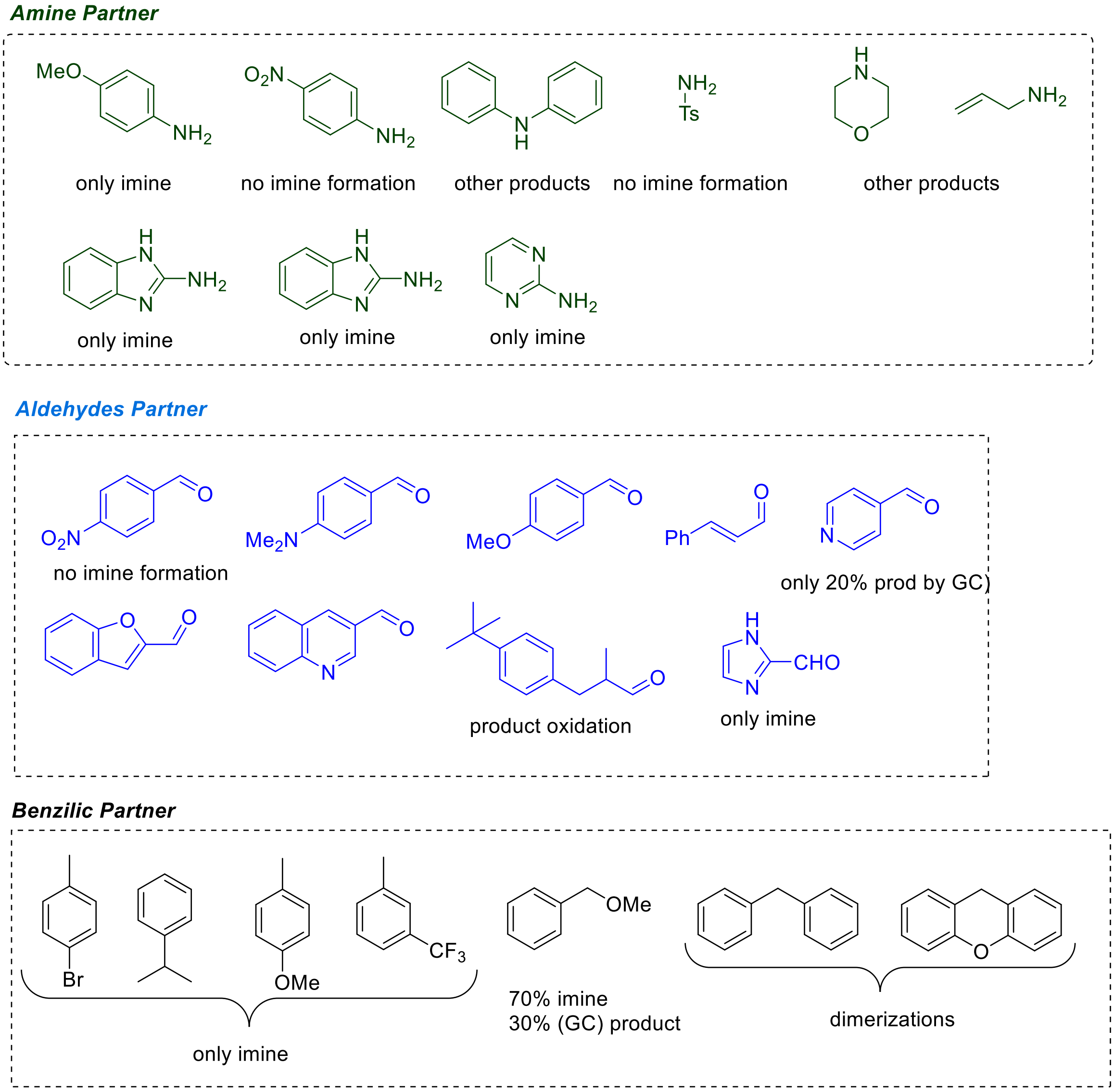

Figure S32: Unsuccessful substrates using GPA

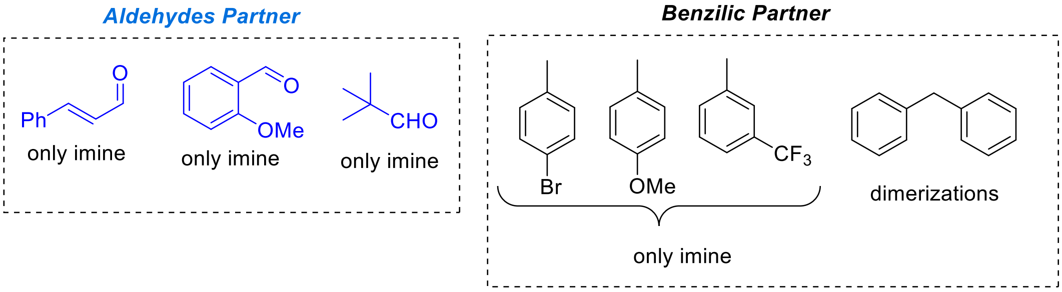

Figure S33: Unsuccessful substrates using GPB

CHARACTERIZATION OF PRODUCTS

***N*-(1,2-diphenylethyl)Aniline (1):**

Following GPA in 16 h. The product was obtained as white oil (72.9 mg, 0.27 mmol, 89%) after FC using a gradient from 0% to 5% of EtOAc in *n*-hexane as the eluent. The spectroscopy data matched previously reported literature values.[18]

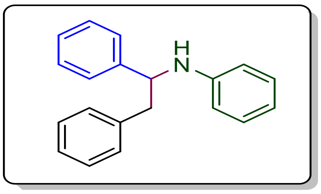

TLC: Rf = 0.41 (9:1 hexane/EtOAc, UV).

GC (Ti= 80 ºC): 7.347 min.

MS: *m/z* (%):273 ($M^{+\bullet}$, 3), 183 (32), 182 (100), 180 (12), 104 (17).

^{1}H NMR (400 MHz, $CDCl_3$): δ 7.29 – 7.08 (m, 8H), 7.05 – 6.98 (m, 2H), 6.98 – 6.87 (m, 2H), 6.52 (tt, *J* = 7.3, 1.1 Hz, 1H), 6.42 – 6.23 (m, 2H), 4.49 (dd, *J* = 8.2, 5.7 Hz, 1H), 4.01 (bs, 1 NH), 3.02 (dd, *J* = 14.0, 5.7 Hz, 1H), 2.90 (dd, *J* = 14.0, 8.2 Hz, 1H).

^{13}C NMR (101 MHz, $CDCl_3$): δ 147.4, 143.5, 137.8, 129.3, 129.1, 128.7, 128.7, 127.2, 126.8, 126.6, 59.3, 45.3.

***N*-(1-phenyl-2-(*p*-tolyl)ethyl)aniline (2)**

Following the GPA for the multicomponent reaction with *p*-xylene (1.5 mmol, 185 µL). The reaction was carried out over 24 h. The product was obtained as a colorless oil (81.8 mg, 0.285 mmol, 95%) after FC using 100% *n*-hexane as the eluent. The spectroscopic data matched those reported in the literature.[18]

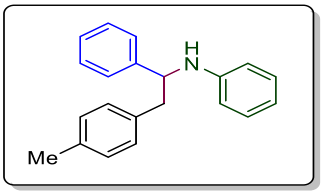

TLC: Rf = 0.56 (9:1 hexane/EtOAc, UV).

GC (Ti= 80 ºC): 7.627 min

MS: *m/z* (%): 287 ($M^{+\bullet}$, 1), 183 (15), 182 (100), 104 (7).

^{1}H NMR (400 MHz, $CDCl_3$) δ 7.29 – 7.22 (m, 4H), 7.20 – 7.17 (m, 1H), 7.03 – 6.94 (m, 6H), 6.56 (tt, *J* = 7.4, 1.1 Hz, 1H), 6.39 (dd, *J* = 8.7, 1.1 Hz, 2H), 4.50 (dd, *J* = 8.4, 5.5 Hz, 1H), 4.05 (bs, 1 NH), 3.04 (dd, *J* = 14.0, 5.6 Hz, 1H), 2.90 (dd, *J* = 14.0, 8.3 Hz, 1H), 2.25 (s, 3H).

^{13}C NMR (101 MHz, $CDCl_3$): δ 147.5, 143.7, 136.4, 134.7, 129.4, 129.2, 129.1, 128.7, 127.1, 126.6, 117.6, 113.8, 59.4, 44.9, 21.2.

***N*-(2-(4-chlorophenyl)-1-phenylethyl)aniline (3)**

Following the GPA for the multicomponent reaction with *p*-chlorotoluene (1.5 mmol, 185 µL). The reaction was carried out over 24 h. The product was obtained as a colorless oil (84.9 mg, 0.276 mmol, 92%) after FC using a gradient from 0% to 20% of EtOAc in *n*-hexane as the eluent. The spectroscopic data matched those reported in the literature.[18]

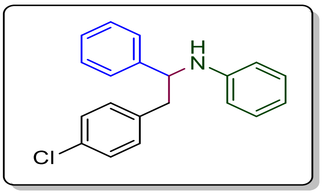

TLC: Rf = 0.43 (9:1 hexane/EtOAc, UV).

GC (Ti= 80 ºC): 8.054 min

MS: *m/z* (%): 309 ($M^{+\bullet}$, ^{37}Cl, 3), 307 ($M^{+\bullet}$, ^{35}Cl, 5), 183 (34), 182 (100), 180 (11), 104 (15).

^{1}H NMR (400 MHz, $CDCl_3$) δ 7.30 – 7.18 (m, 7H), 7.07 – 7.02 (m, 2H), 7.00 – 6.96 (m, 2H), 6.63 (tt, *J* = 7.3, 1.1 Hz, 1H), 6.46 (dd, *J* = 8.6, 1.1 Hz, 2H), 4.54 (t, 1H), 4.09 (bs, 1 NH), 3.06 (dd, *J* = 14.0, 6.3 Hz, 1H), 3.00 (dd, *J* = 14.0, 7.5 Hz, 1H).

^{13}C NMR (101 MHz, CDCl3): δ 147.1, 143.0, 136.2, 132.6, 130.7, 129.2, 128.74, 128.71, 127.3, 126.6, 117.8, 113.7, 59.2, 44.4.

_N_-(2-(3,5-dimethylphenyl)-1-phenylethyl)aniline (4)

Following GPA with mesitylene (180 mg, 1.5 mmol) in 16 h. The product was obtained as white oil (81.3 mg, 0.27 mmol, 90%) after FC using a gradient from 0% to 5% of EtOAc in *n*-hexane as the eluent. The spectroscopy data matched previously reported literature values.[19]

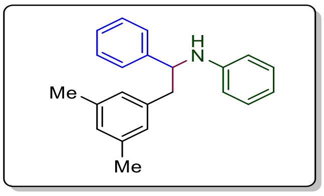

TLC: Rf = 0.64 (9:1 hexane/EtOAc, UV).

GC (Ti= 80 ºC): 7.747 min.

MS: *m/z* (%): 301 ($M^{+\bullet}$, 2), 183 (29), 182 (9), 119 (4), 105 (2).

^{1}H NMR (400 MHz, $CDCl_3$): δ 7.49 – 7.38 (m, 3H), 7.35 (d, *J* = 7.1 Hz, 1H), 7.15 (dd, *J* = 8.6, 7.3 Hz, 2H), 7.01 – 6.90 (m, 2H), 6.89 – 6.83 (m, 2H), 6.73 (s, 1H), 6.56 (dt, *J* = 7.7, 1.1 Hz, 2H), 4.66 (dd, *J* = 8.7, 5.3 Hz, 1H), 4.24 (bs, 1 NH), 3.18 (dd, *J* = 13.9, 5.2 Hz, 1H), 2.98 (dd, *J* = 13.9, 8.7 Hz, 1H), 2.39 (d, *J* = 0.7 Hz, 3H), 2.38 (d, *J* = 0.8 Hz, 3H).

^{13}C NMR (101 MHz, CDCl3): δ 147.5, 143.9, 138.1, 137.8, 137.7, 129.1, 128.6, 128.5, 127.1, 127.1, 127.0, 126.5, 117.5, 113.8, 59.4, 45.4, 21.4, 21.3.

_N_-(1,2-diphenylpropyl)aniline (5)

Following the GPA with ethylbenzene (159 mg, 1.5 mmol) in 16 h. The product was obtained as white oil (70.6 mg, 0.25 mmol, 82%, 53:47 dr according to GC-MS (48:52 dr before purification)) after FC using a gradient from 0% to 5% of EtOAc in *n*-hexane as the eluent. The spectroscopy data matched those previously reported in the literature.[20]

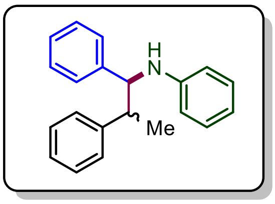

TLC: Rf = 0.58 (9:1 hexane/EtOAc, UV).

GC1 (Ti= 80 ºC): 7.332 min.

MS1: *m/z* (%): 287 ($M^{+\bullet}$, 1), 183 (16), 182 (100), 180 (6), 104 (7).

GC2 (Ti= 80 ºC): 7.415 min.

MS2: *m/z* (%): 287 ($M^{+\bullet}$, 1), 183 (15), 182 (100), 180 (8), 104 (9).

^{1}H NMR (400 MHz, $CDCl_3$): δ 7.46 – 7.36 (m, 6H), 7.36 – 7.25 (m, 10H), 7.23 – 7.16 (m, 4H), 7.10 (dd, *J* = 22.5, 1.4 Hz, 4H), 6.72 – 6.63 (m, 2H), 6.57 – 6.52 (m, 2H), 6.48 – 6.44 (m, 2H), 4.62 (d, *J* = 5.7 Hz,

1H), 4.45 (dd, J = 8.4, 3.6 Hz, 1H), 4.26 (d, J = 5.8 Hz, 1H), 4.09 (d, J = 3.9 Hz, 1H), 3.33 (dd, J = 7.3, 5.6 Hz, 1H), 3.18 – 3.05 (m, 1H), 1.43 (d, J = 7.2 Hz, 3H), 1.27 (d, J = 7.0 Hz, 3H).

^{13}C NMR (101 MHz, $CDCl_3$): δ 147.7, 147.4, 143.3, 142.9, 142.9, 141.6, 129.1, 129.0, 128.8, 128.5, 128.3, 128.2, 128.1, 127.8, 127.6, 127.5, 127.2, 127.1, 126.9, 126.8, 117.4 113.7, 63.9, 63.2, 47.5, 45.9, 19.7, 16.5.

***N*-((2,3-dihydro-1H-inden-1-yl)(phenyl)methyl)aniline (6) :**

Following the GPA with 2,3-dihydro-1H-indene (177 mg, 1.5 mmol) in 16 h. The product was obtained as white oil (79.8 mg, 0.27 mmol, 89%, 58:42 dr according to ^{1}H-NMR (59:41 dr before purification)) after FC using a gradient from 0% to 5% of EtOAc in *n*-hexane as the eluent.

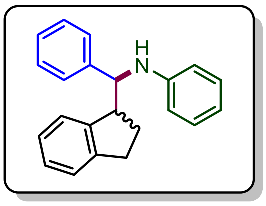

TLC: Rf = 0.62 (9:1 hexane/EtOAc, UV).

GC1 (Ti= 80 ºC): 8.074 min.

MS1: *m/z* (%): 299 ($M^{+\bullet}$, 1), 183 (26), 182 (100), 180 (8), 104 (13).

GC2 (Ti= 80 ºC): 8.100 min.

MS2: *m/z* (%): 299 ($M^{+\bullet}$, 1), 183 (21), 182 (100), 180 (6), 104 (10).

HRMS (EI-TOF): *m/z* calcd for $C_{22}H_{21}N$ 299.1664, found 299.1657.

^{1}H NMR (400 MHz, $CDCl_3$): δ 7.26 – 7.20 (m, 2H), 7.19 – 7.13 (m, 3H), 7.13 – 7.06 (m, 2H) 7.01 – 6.89 (m, 3.16H), 6.84 (d, J = 7.5 Hz, 0.84H), 6.54 (ddt, J = 8.5, 7.3, 3.0 Hz, 1H), 6.41 – 6.32 (m, 2H), 4.63 (d, J = 5.49 Hz, 0.58H), 4.55 (d, J = 5.17 Hz, 0.42H), 3.88 (bs, 1H), 3.58 (dd, J = 16.7, 7.4 Hz, 1H), 2.81–2.73 (m, 1H), 2.72 – 2.57 (m, 1H), 2.12 – 2.00 (m, 1H), 1.98 – 1.81 (m, 1H).

^{13}C NMR (101 MHz, CDCl3): δ 147.9, 147.5, 145.5, 145.2, 142.9, 142.8, 142.6, 141.9, 129.2, 129.1, 128.5, 128.2, 127.4, 127.3, 127.2, 127.1, 127.0, 126.3, 126.2, 125.4, 124.8, 124.8, 124.6, 117.6, 117.3, 113.8, 113.4, 61.6, 59.9, 52.5, 51.9, 31.6, 31.1, 29.8, 27.6.

***N*-(1,2-diphenylpent-4-en-1-yl)aniline (7) :**

Following the GPA with but-3-en-1-ylbenzene (198 mg, 1.5 mmol) in 16 h. The product was obtained as white oil (80.7 mg, 0.26 mmol, 86%, 35:65 dr according to GC-MS (43:57 dr before purification)) after FC using a gradient from 0% to 5% of EtOAc in *n*-hexane as the eluent.

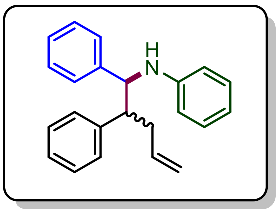

TLC: Rf = 0.64 (9:1 hexane/EtOAc, UV).

GC1 (Ti= 80 ºC): 7.731 min (diastereoisomer A).

MS1: *m/z* (%): 313 ($M^{+\bullet}$, 1), 182 (100), 183 (42), 152 (3).

GC2 (Ti= 80 ºC): 7.663 min (diastereoisomer B).

MS2: *m/z* (%): *m/z* (%): 313 ($M^{+\bullet}$, 1), 182 (100), 183 (36), 165 (4).

HRMS (EI-TOF): *m/z* calcd for $C_{23}H_{23}N$ 313.1826, found 313.1818.

^{1}H NMR (400 MHz, $CDCl_3$): δ 7.32 – 7.04 (m, 7H), 7.02 – 6.82 (m, 6H), 6.62 – 6.47 (m, 1H), 6.45 – 6.38 (m, 1.26H), 6.35 – 6.24 (m, 0.74H), 5.68 – 5.57 (m, 0.63H), 5.54 – 5.41 (m, 0.37H), 4.96 (dq, J =

17.1, 1.6 Hz, 0.63H), 4.89 (ddd, *J* = 10.2, 2.1, 1.1 Hz, 0.63H), 4.83 (s, 0.37H), 4.82 – 4.75 (m, 0.37H), 4.55 (d, *J* = 5.9 Hz, 0.63H), 4.45 (d, *J* = 7.1 Hz, 0.37H), 3.10 (dt, *J* = 9.4, 5.7 Hz, 0.63H), 2.93 (dt, *J* = 8.4, 6.6 Hz, 0.37H), 2.59 (dddt, *J* = 14.2, 7.0, 5.6, 1.3 Hz, 0.74H), 2.49 – 2.27 (m, 1.26H).

^{13}C NMR (101 MHz, $CDCl_3$): δ 147.4, 147.0, 142.7, 140.7, 140.2, 140.1, 129.3, 129.2, 129.1, 128.9, 128.7, 128.7, 128.5, 128.4, 128.1, 127.9, 127.7, 127.5, 127.5, 127.3, 127.1, 127.1, 126.9, 126.8, 61.8, 61.6, 53.1, 51.6, 37.0, 35.9.

_N_-(2-(4-chlorophenyl)-1-phenylethyl)aniline (8)

Following the GPA for the multicomponent reaction with isochromane (1.5 mmol, 201 mg). The reaction was carried out over 24 h. The product was obtained as a colorless oil (46.4 mg, 0.147 mmol, 51%, 62:38 dr according to ^{1}H-NMR (70:30 dr before purification)) after FC using a gradient from 0% to 20% of EtOAc in *n*-hexane as the eluent.

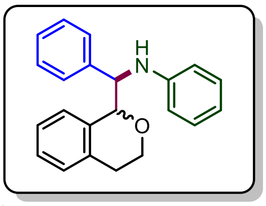

TLC: Rf = 0.39 (9:1 hexane/EtOAc, UV).

GC (Ti= 80 ºC): 8.343 min

MS: *m/z* (%): 315 ($M^{+\bullet}$, 1), 183 (38), 182 (100), 180 (10), 104 (16).

HRMS (ESI-TOF): *m/z* calcd for $C_{22}H_{22}NO^+$ 316.1696, found 316.1699.

^{1}H NMR (400 MHz, $CDCl_3$) δ 7.63 (d, *J* = 6.6 Hz, 0.76H), 7.54 (t, *J* = 7.5 Hz, 0.76H), 7.50 – 7.39 (m, 2H), 7.37 – 7.28 (m, 5H), 7.27 – 7.18 (m, 1.86H), 7.13 (d, *J* = 7.4 Hz, 0.62H), 6.83 (t, *J* = 7.3 Hz, 0.62H), 6.78 (d, *J* = 6.6 Hz, 1.62H), 6.63 (d, *J* = 7.6 Hz, 0.76H), 5.57 (s, 0.62H), 5.22 (s, 0.38H), 5.05 (s, 1H), 4.42 – 4.20 (m, 1.24H), 3.97 – 3.76 (m, 1H), 3.39 – 3.16 (m, 0.38H), 2.96 – 2.84 (m, 0.38H), 2.65 – 2.44 (m, 1H), 2.24 (s, 0.38H), 1.52 – 1.43 (m, 0.62H).

^{13}C NMR (101 MHz, $CDCl_3$): δ 147.4 (C), 147.1 (C), 141.3 (C), 138.5 (C), 135.8 (C), 135.1 (C), 134.7 (C), 134.5 (C), 129.3 (CH), 129.09 (CH), 129.05 (CH), 128.7 (CH), 128.6 (CH), 128.5 (CH), 127.7 (CH), 127.5 (CH), 127.4 (CH), 127.0 (CH), 126.9 (CH), 126.6 (CH), 126.4 (CH), 126.1 (CH), 125.7 (CH), 125.1 (CH), 117.4 (CH), 117.1 (CH), 113.6 (CH), 113.5 (CH), 80.4 (CH), 78.9 (CH), 64.2 (CH_2), 63.9 (CH_2), 62.8 (CH), 61.4 (CH), 29.2 (CH_2), 29.1 (CH_2).

1,2,3-triphenylpyrrolidine (9) :

Following the GPA with (3-chloropropyl)benzene (231 mg, 1.5 mmol) in 16 h. The product was obtained as white oil (80.7 mg, 0.27 mmol, 90%, 73:27 dr according to GC-MS (61:39 dr before purification)) after FC using a gradient from 0% to 5% of EtOAc in *n*-hexane as the eluent.

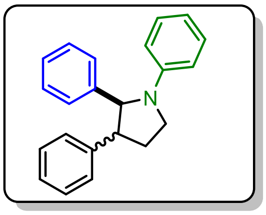

TLC: Rf = 0.54 (9:1 hexane/EtOAc, UV).

GC1 (Ti= 80 ºC): 8.020 min (diastereoisomer A).

MS1: *m/z* (%): 299 ($M^{+\bullet}$, 61), 194 (100), 195 (37), 300 (14).

GC2 (Ti= 80 ºC): 8.105 min (diastereoisomer B).

MS2: *m/z* (%): *m/z* (%): 299 ($M^{+\bullet}$, 53), 194 (100), 195 (33), 300 (17).

HRMS (EI-TOF): *m/z* calcd for $C_{22}H_{21}N$ 299.1674, found 299.1692.

^{1}H NMR (400 MHz, $CDCl_3$): δ 7.33 – 7.19 (m, 2H), 7.17 – 6.95 (m, 9H), 6.71 (d, *J* = 2.2 Hz, 1H), 6.63 – 6.52 (m, 1H), 6.48 – 6.25 (m, 2H), 4.80 (d, *J* = 3.7 Hz, 0.29H), 4.74 (d, *J* = 3.7 Hz, 0.71H), 3.86 – 3.66 (m, 1H), 3.66 – 3.5 (m, 1H), 3.35 – 3.19 (m, 0.71H), 2.39 (dd, *J* = 12.4, 7.2 Hz, 1 H), 2.16 (d, *J* = 6.1 Hz, 0.29H), 2.05 – 1.88 (m, 1H).

^{13}C NMR (101 MHz, $CDCl_3$): δ 146.7, 146.5, 129.1, 129.0, 128.9, 128.7, 128.7, 128.6, 128.5, 128.0, 127.7, 127.6, 127.4, 127.2, 126.9, 126.7, 126.6, 125.9, 116.0, 115.9, 112.7, 111.9, 69.5, 67.4, 54.8, 50.7, 48.1, 47.9.

***N*-(1-(4-bromophenyl)-2-phenylethyl)Aniline (10):**

Following the GPA with 4-bromobenzaldehyde (60.4 mg, 0.33 mmol) in 16 h. The product was obtained as white oil (77.9 mg, 0.22 mmol, 74%) after FC using a gradient from 0% to 5% of EtOAc in *n*-hexane as the eluent. The spectroscopy data matched previously reported literature values.[18]

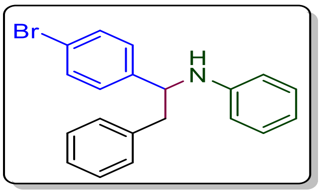

TLC: Rf = 0.54 (9:1 hexane/EtOAc, UV).

GC (Ti= 80 ºC): 8.385 min.

MS: *m/z* (%): 351 ($M^{+\bullet}$, 1), 263 (20), 262 (98), 261 (23), 181 (13).

^{1}H NMR (400 MHz, $CDCl_3$): δ 7.39 – 7.31 (m, 2H), 7.24 – 7.20 (m, 2H), 7.18 – 7.15 (m, 1H), 7.13 – 7.09 (m, 2H), 7.05 – 6.95 (m, 4H), 6.57 (tt, *J* = 7.3, 1.1 Hz, 1H), 6.38 – 6.22 (m, 2H), 4.47 (t, *J*= 7.2 Hz, 1H), 4.03 (bs, 1 NH), 3.01 (dd, *J* = 13.9, 5.9 Hz, 1H), 2.92 (dd, *J* = 13.9, 8.0 Hz, 1H).

^{13}C NMR (101 MHz, $CDCl_3$): δ 146.9, 142.5, 137.2, 131.7, 129.2, 129.1, 128.6, 128.3, 126.9, 120.7, 117.8, 113.6, 58.8, 45.0.

***N*-(1-(4-chlorophenyl)-2-phenylethyl)aniline (11)**

Following the GPA for the multicomponent with *p*-chlorobenzaldehyde (0.33 mmol, 50.6 mg). The reaction was carried out over 3 h. The product was obtained as a colorless oil (65.5 mg, 0.213 mmol, 71%) after FC using a gradient from 0% to 20% of EtOAc in *n*-hexane as the eluent. The spectroscopic data matched those reported in the literature.[18]

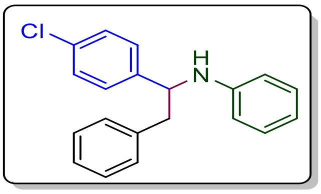

TLC: Rf = 0.6 (9:1 hexane/EtOAc, UV).

GC (Ti= 80 ºC): 8.032 min

MS: *m/z* (%): 309 ($M^{+\bullet}$, ^{37}Cl, 1), 307 ($M^{+\bullet}$, ^{35}Cl, 4), 218 (87), 217 (49), 216 (100), 214 (12), 180 (19), 179 (10), 104 (34).

^{1}H NMR (400 MHz, $CDCl_3$): δ 7.32 –7.21 (m, 7H), 7.13 – 7.02 (m, 4H), 6.65 (tt, *J* = 7.4, 1.1 Hz, 1H), 6.43 (dd, *J* = 8.7, 1.1 Hz, 2H), 4.56 (t, *J* = 7.0 Hz, 1H), 4.11 (bs, 1 NH), 3.09 (dd, *J* = 13.9, 6.0 Hz, 1H), 3.01 (dd, *J* = 13.9, 7.9 Hz, 1H).

^{13}C NMR (101 MHz, CDCl3): δ 147.4, 142.4, 137.6, 133.1, 129.7, 129.1, 129.2, 129.1, 128.3, 127.3, 118.2, 114.1, 59.1, 45.5.

***N*-(2-phenyl-1-(*p*-tolyl)ethyl)aniline (12)**

Following the GPA for the multicomponent reaction with *p*-methylbenzaldehyde (0.33 mmol, 42 μL). The reaction was carried out over 3 h. The product was obtained as a colorless oil (77.6 mg, 0.27 mmol, 90%) after FC using a gradient from 0% to 5% of EtOAc in *n*-hexane as the eluent. The spectroscopic data matched those reported in the literature.[18]

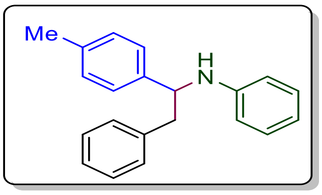

TLC: Rf = 0.73 (9:1 hexane/EtOAc, UV).

GC (Ti= 80 ºC): 7.627 min

MS: *m/z* (%): 281 ($M^{+\bullet}$, 5), 207 (10), 197 (44), 196 (100), 194 (12), 104 (36).

^{1}H NMR (400 MHz, $CDCl_3$) δ 7.46 – 7.39 (m, 3H), 7.37 – 7.34 (m, 3H), 7.29 – 7.23 (m, 3H), 7.21 – 7.15 (m, 2H), 6.78 – 6.73 (tt, *J* = 7.4, 1.1 Hz, 1H), 6.60 (dd, *J* = 8.7, 1.1 Hz, 2H), 4.70 (dd, *J* = 8.3, 5.6 Hz, 1H), 4.22 (bs, 1 NH), 3.26 (dd, *J* = 14.0, 5.7 Hz, 1H), 3.13 (dd, *J* = 14.0, 8.3 Hz, 1H), 2.46 (s, 3H).

^{13}C NMR (101 MHz, CDCl3): δ 147.5, 140.6, 138.0, 136.7, 129.5, 129.3, 129.1, 128.7, 126.8, 126.5, 117.5, 113.8, 59.1, 45.3, 21.2.

***N*-(1-(4-(tert-butyl)phenyl)-2-phenylethyl)aniline (13)**

Following the GPA for the multicomponent reaction with *p*-*tert*-butylbenzaldehyde (0.33 mmol, 62 μL). The reaction was carried out over 3 h. The product was obtained as a colorless oil, 91.9 mg (0.279 mmol, 93%), after FC using a gradient from 0% to 5% of EtOAc in *n*-hexane as the eluent. The spectroscopic data matched those reported in the literature.[18]

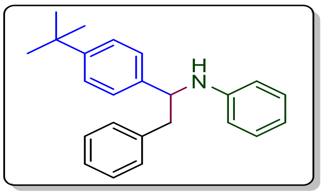

TLC: Rf = 0.73 (9:1 hexane/EtOAc, UV).

GC (Ti= 80 ºC): 8.244 min

MS: *m/z* (%): 328 ($M^{+\bullet}$, 1), 239 (41), 238 (100), 223 (19), 222 (15), 208 (16).

^{1}H NMR (400 MHz, $CDCl_3$) δ 7.34 – 7.28 (m, 2H), 7.28 – 7.27 (m, 2H), 7.26 – 7.18 (m, 3H), 7.17 – 7.13 (m, 2H), 7.08 – 7.02 (m, 2H), 6.62 (tt, *J* = 7.3, 1.1 Hz, 1H), 6.47 (dd, *J* = 8.7, 1.1 Hz, 2H), 4.57 (dd, *J* = 8.6, 5.4 Hz, 1H), 3.15 (dd, *J* = 14.2, 5.4 Hz, 1H), 2.98 (dd, *J* = 14.2, 8.6 Hz, 1H), 1.31 (s, 9H).

^{13}C NMR (101 MHz, CDCl3): δ 150.0, 147.6, 140.6, 138.1, 129.3, 129.1, 128.7, 126.8, 126.1, 125.6, 117.5, 113.7, 58.9, 45.3, 34.6, 31.5.

***N*-(2-phenyl-1-(4-(trifluoromethyl)phenyl)ethyl)Aniline (14):**

Following the GPA with 4-(trifluoromethyl)benzaldehyde (57.4 mg, 0.33 mmol) in 16 h. The product was obtained as white oil (55.2 mg, 0.16 mmol, 54%) after FC using a gradient from 0% to 5% of EtOAc in *n*-hexane as the eluent. The spectroscopy data matched previously reported literature values.[21]

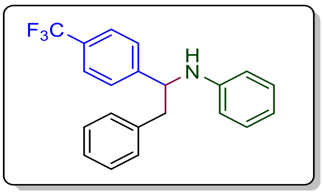

TLC: Rf = 0.51 (9:1 hexane/EtOAc, UV).

GC (Ti= 80 ºC): 7.160 min.

MS: *m/z* (%): 341 ($M^{+\bullet}$, 1), 251 (15), 250 (100), 248 (8), 207 (11).

^{1}H NMR (400 MHz, CDCl3): δ 7.56 (d, *J* = 7.8 Hz, 2H), 7.44 (d, *J* = 1.5 Hz, 2H), 7.29 (ddt, *J* = 7.9, 6.2, 1.4 Hz, 2H), 7.25 (d, *J* = 2.7 Hz, 1H), 7.11 (d, *J* = 7.0 Hz, 2H), 7.09 – 7.02 (m, 2H), 6.69 – 6.63 (m, 1H), 6.42 (d, *J* = 7.6 Hz, 2H), 4.64 (dd, *J* = 14.9, 4.9 Hz, 1H), 4.03 (bs, 1 NH), 3.11 (dd, *J* = 14.0, 5.4 Hz, 1H) 3.04 (dd, *J* = 14.0, 8.4 Hz, 1H).

^{13}C NMR (101 MHz, $CDCl_3$): δ 147.8, 146.9, 137.1, 129.3, 129.3, 129.2, 128.9, 127.2, 126.9, 125.73 (q, $J_{C\text{-}F}$= 4.10 Hz), 118.1, 113.8, 59.1, 45.1.

^{19}F NMR (282 MHz, $CDCl_3$): δ -62.31.

4-(2-phenyl-1-(phenylamino)ethyl)benzonitrile (15)

Following the GPA with *p*-cyanobenzaldehyde (0.33 mmol, 47.2 mg) in 24 h. The reaction was carried out over 24 h. The product was obtained as a brown oil (36.6 mg, 0.123 mmol, 41%) after FC using a gradient from 0% to 50% of EtOAc in *n*-hexane as the eluent. The spectroscopic data matched those reported in the literature.[22]

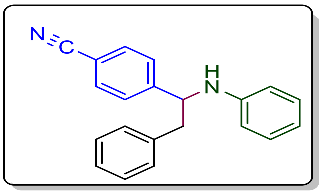

TLC: Rf = 0.23 (9:1 hexane/EtOAc, UV).

GC (Ti= 80 ºC): 8.551 min

MS: *m/z* (%): 298 ($M^{+\bullet}$, 1), 208 (17), 207 (100)

^{1}H NMR (400 MHz, $CDCl_3$): δ 7.62 – 7.56 (m, 2H), 7.45 – 7.38 (m, 2H), 7.32 – 7.21 (m, 3H), 7.10 – 7.03 (m, 4H), 6.67 (tt, *J* = 7.4, 1.1 Hz, 1H), 6.39 (dd, *J* = 8.7, 1.0 Hz, 2H), 4.63 (t, *J* = 6.9 Hz, 1H), 4.16 (bs, 1 NH), 3.10 (dd, *J* = 13.9, 6.1 Hz, 1H), 3.03 (dd, *J* = 13.9, 7.9 Hz, 1H).

^{13}C NMR (101 MHz, $CDCl_3$): δ 149.3, 146.7, 136.7, 132.6, 129.3, 129.3, 128.9, 127.4, 127.3, 119.0, 118.3, 113.8, 111.1, 59.3, 44.9.

***N*-(1-(4-chloropyridin-2-yl)-2-phenylethyl)aniline (16):**

Following the GPA with 4-chloropicolinaldehyde (46 mg, 0.33 mmol) and 2 equiv. TFA in 16 h. The product was obtained as a green solid (28 mg, 0.09 mmol, 30%) after FC using a gradient from 0% to 20% of EtOAc in *n*-hexane as the eluent.

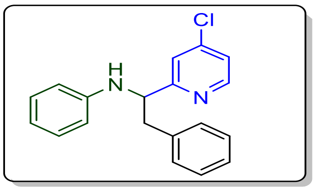

TLC: Rf = 0.61 (9:1 hexane/EtOAc, UV).

GC (Ti= 80 ºC): 7.773 min

MS: *m/z* (%):308 ($M^{+•}$, 5), 217 (62), 207 (100).

HRMS (EI-QTOF): *m/z* calcd for $C_{19}H_{15}ClN_2$ [-H_2] 306.0924 observed 306.0905.

^{1}H NMR (400 MHz, $CDCl_3$): δ 8.51 (dd, *J* = 5.3, 0.6 Hz, 1H), 7.30 – 7.20 (m, 6H), 7.18 (dd, *J* = 5.3, 2.0 Hz, 1H), 7.13 – 7.07 (m, 4H), 6.68 (tt, *J* = 7.3, 1.1 Hz, 1H), 6.52 – 6.47 (m, 2H), 4.71 (t, *J* = 6.9 Hz, 1H), 4.37 (s, 1H), 3.25 (dd, *J* = 13.9, 5.7 Hz, 1H), 3.06 (dd, *J* = 13.9, 8.0 Hz, 1H).

^{13}C NMR (101 MHz, $CDCl_3$): δ 164.3, 150.3, 146.7, 144.8, 137.1, 129.2, 129.1, 128.6, 126.8, 122.6, 121.7, 117.9, 113.6, 60.3, 42.8.

_N_-(3,3-dimethyl-1-phenylbutan-2-yl)Aniline (17)

Following the GPA with pivalaldehyde (25.8 mg, 0.33 mmol) in 16 h. The product was obtained as white oil (61.5 mg, 0.24 mmol, 81%) after FC using a gradient from 0% to 5% of EtOAc in *n*-hexane as the eluent.

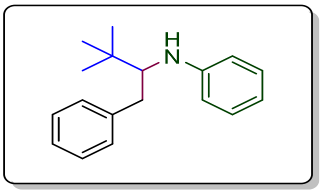

TLC: Rf = 0.72 (9:1 hexane/EtOAc, UV).

GC (Ti= 80 ºC): 5.837 min.

MS: *m/z* (%): 253 ($M^{+•}$, 9), 197 (13), 196 (85), 163 (13), 162 (100).

HRMS (EI-TOF): *m/z* calcd for $C_{18}H_{23}N$ 253.1823, found 253.1813.

^{1}H NMR (400 MHz, $CDCl_3$): δ 7.24 – 7.19 (m, 4H), 7.14 (dt, *J* = 5.5, 3.9 Hz, 1H), 7.01 (ddd, *J* = 8.4, 6.5, 0.9 Hz, 2H), 6.59 – 6.49 (m, 1H), 6.37 (dt, *J* = 7.6, 0.9 Hz, 2H), 3.50 (bs, 1 NH), 3.43 (dd, *J* = 10.1, 3.1 Hz, 1H), 3.13 (dd, *J* = 13.9, 2.5 Hz, 1H), 2.51 (dd, *J* = 13.8, 9.8 Hz, 1H), 1.06 (s, 9H).

^{13}C NMR (101 MHz, $CDCl_3$): δ 149.5, 140.4, 129.3, 128.9, 128.3, 125.9, 116.3, 112.8, 64.3, 38.4, 36.6, 27.1.

_N_-(1-cyclohexyl-3-phenylpropan-2-yl)aniline (18)

Following the GPA for the multicomponent reaction with cyclohexanecarboxaldehyde (0.33 mmol, 44 µL). The reaction was carried out over 24 h. The product was obtained as a colorless oil (67.9 mg, 0.243 mmol, 81%) after FC using *n*-hexane as the eluent.

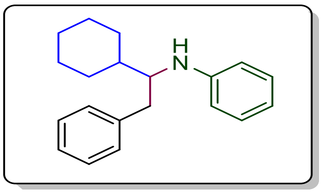

TLC: Rf = 0.74 (9:1 hexane/EtOAc, UV).

GC (Ti= 80 ºC): 7.342 min

MS: *m/z* (%): 279 ($M^{+•}$, 8), 196 (35), 189 (32), 188 (100), 106 (34)

HRMS (EI-TOF): *m/z* calcd for $C_{20}H_{25}N$ 279.1987, found 279.1971.

^{1}H NMR (400 MHz, $CDCl_3$): δ 7.32 – 7.26 (m, 2H), 7.23 – 7.17 (m, 3H), 7.19 – 7.10 (m, 2H), 6.64 (td, *J* = 7.3, 1.1 Hz, 1H), 6.56 (dd, *J* = 7.6, 1.1 Hz, 2H), 3.51 (q, *J* = 5.9 Hz, 1H), 2.89 (dd, *J* = 13.9, 5.9 Hz, 1H), 2.78 (dd, *J* = 14.0, 6.8 Hz, 1H), 1.92 – 1.65 (m, 5H), 1.56 – 1.48 (m, 1H), 1.29 – 1.06 (m, 6H).

^{13}**C NMR** (101 MHz, CDCl3): δ 148.3, 139.5, 129.41, 129.40, 128.4, 126.2, 116.7, 113.1, 59.1, 40.5, 37.5, 30.1, 28.6, 26.7, 26.55, 26.50.

***N*-(4-methyl-1-phenylpentan-2-yl)aniline (19)**

Following the GPA for the multicomponent reaction with 3-methylbutanal (0.33 mmol, 39 μL). The reaction was carried out over 3 h. The product was obtained as a brown oil (51.3 mg, 0.192 mmol, 64%) after FC using a gradient from 0% to 5% of EtOAc in *n*-hexane as the eluent. The spectroscopic data matched those reported in the literature.[23]

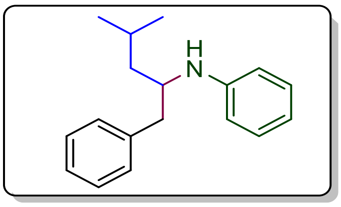

TLC: Rf = 0.76 (9:1 hexane/EtOAc, UV).

GC (Ti= 80 ºC): 6.034 min

MS: *m/z* (%): 253 ($M^{+•}$, 6), 163 (15), 162 (100), 120 (15), 106 (15)

^{1}**H NMR** (400 MHz, $CDCl_3$): δ 7.41 – 7.34 (m, 3H), 7.33 – 7.22 (m, 4H), 6.77 (t, *J* = 6.8 Hz, 1H), 6.71 (d, *J* = 7.7 Hz, 2H), 3.82 (p, *J* = 6.1 Hz, 1H), 3.50 (bs, 1 NH), 2.98 – 2.86 (m, 2H), 1.89 (hept, *J* = 6.4 Hz, 1H), 1.47 – 1.34 (m, 2H), 1.02 (d, *J* = 6.7 Hz, 3H), 0.94 (d, *J* = 6.5 Hz, 3H).

^{13}**C NMR** (101 MHz, CDCl3): δ 147.7, 138.6, 129.8, 129.5, 128.3, 126.3, 117.0, 113.2, 51.5, 43.7, 40.4, 24.5, 23.4, 22.3.

4-Chloro-*N*-(1,2-diphenylethyl)aniline (20):

Following the GPA with 4-chloroaniline (38.1 mg, 0.30 mmol) in 16 h. The product was obtained as white oil (81.0 mg, 0.26 mmol, 88%) after FC using a gradient from 0% to 5% of EtOAc in *n*-hexane as the eluent. The spectroscopy data matched previously reported literature values.[18]

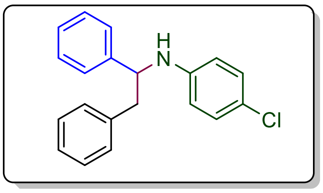

TLC: Rf = 0.62 (9:1 hexane/EtOAc, UV).

GC (Ti= 80 ºC): 8.151 min.

MS: *m/z* (%):307 ($M^{+•}$, 2), 218 (37), 216 (100), 180 (5), 138 (11).

^{1}**H NMR** (400 MHz, $CDCl_3$): δ 7.29 – 7.07 (m, 8H), 7.00 (t, *J* = 3.3 Hz, 2H), 6.93 – 6.77 (m, 2H), 6.42 – 6.08 (m, 2H), 4.45 (s, 1H), 4.10 – 3.98 (m, 1H), 3.04 (dd, *J* = 14.0, 5.6 Hz, 1H), 2.90 (dd, J = 14.0, 8.2 Hz, 1H).

^{13}**C NMR** (101 MHz, $CDCl_3$): δ 145.8, 142.9, 137.5, 129.2, 128.9, 128.7, 128.6, 127.3, 126.9, 126.4, 122.2, 114.8, 59.3, 45.1.

4-Bromo-*N*-(1,2-diphenylethyl) (21):

Following the GPA with 4-bromoaniline (51 mg, 0.30 mmol) in 16 h. The product was obtained as a green oil (93 mg, 0.26 mmol, 88%) after FC using a gradient from 0% to 20% of EtOAc in *n*-hexane as the eluent.

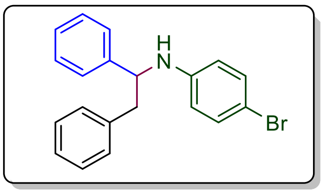

TLC: Rf = 0.39 (9:1 hexane/EtOAc, UV).

GC (Ti= 80 ºC): 8.504 min

MS: *m/z* (%): 351 ($M^{+\bullet}$, 9), 353 (9), 262 (95), 260 (100).

HRMS (ESI-QTOF): *m/z* calcd for $[M+H]^+$ for $C_{20}H_{19}BrN^+$ 352.0695 observed 352.0700.

^{1}H NMR (400 MHz, $CDCl_3$): δ 7.33 – 7.20 (m, 8H), 7.17 – 7.05 (m, 4H), 6.33 (d, *J* = 8.8 Hz, 2H), 4.54 (dd, *J* = 8.2, 5.7 Hz, 1H), 4.14 (bs, 1 NH), 3.14 (dd, *J* = 14.0, 5.7 Hz, 1H), 3.00 (dd, *J* = 14.0, 8.2 Hz, 1H).

^{13}C NMR (101 MHz, $CDCl_3$): δ 146.2, 142.8, 137.4, 131.7, 129.1, 128.6, 128.6, 127.2, 126.8, 126.3, 115.2, 109.2, 59.2, 45.0.

4-Fluoro-*N*-(1,2-diphenylethyl)aniline (22):

Following the GPA with 4-fluoroaniline (29 µL, 0.30 mmol) in 16 h. The product was obtained as white oil (79 mg, 0.27 mmol, 90%) after FC using a gradient from 0% to 20% of EtOAc in *n*-hexane as the eluent. The spectroscopy data matched previously reported literature values.[18]

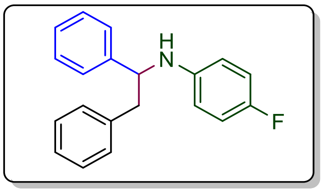

TLC: Rf = 0.55 (9:1 hexane/EtOAc, UV).

GC (Ti= 80 ºC): 7.319 min

MS: *m/z* (%): 291 ($M^{+\bullet}$, 2), 207 (20), 201 (18), 200 (100).

^{1}H NMR (400 MHz, $CDCl_3$): δ 7.29 – 7.25 (m, 4H), 7.25 – 7.16 (m, 4H), 7.08 (d, *J* = 6.7 Hz, 2H), 6.70 (t, *J* = 8.8 Hz, 2H), 6.33 (dd, *J* = 9.0, 4.4 Hz, 2H), 4.48 (dd, *J* = 5.1, 2.6 Hz, 1H), 3.96 (bs, 1 NH), 3.08 (dd, *J* = 14.0, 5.6 Hz, 1H), 2.95 (dd, *J* = 14.0, 8.3 Hz, 1H).

^{13}C NMR (101 MHz, $CDCl_3$): δ 155.9 (d, $J_{F,C}$= 225 Hz), 143.7, 143.3, 137.6, 129.2, 128.6, 128.6, 127.2, 126.8, 126.5, 115.6 (d, $J_{F,C}$= 23 Hz), 114.5 (d, $J_{F,C}$= 8.2 Hz), 59.8, 45.2.

^{19}F NMR (377 MHz, $CDCl_3$): δ -127.82.

3-Fluoro-*N*-(1,2-diphenylethyl) (23):

Following the GPA with 3-fluoroaniline (30 µL, 0.30 mmol) in 16 h. The product was obtained as a white oil (70 mg, 0.24 mmol, 80%) after FC using a gradient from 0% to 20% of EtOAc in *n*-hexane as the eluent. The spectroscopy data matched previously reported literature values. [24]

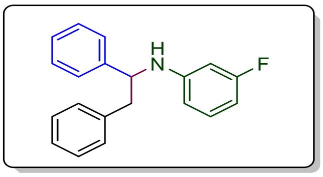

TLC: Rf = 0.47 (9:1 hexane/EtOAc, UV).

GC (Ti= 80 ºC): 7.331 min

MS: *m/z* (%): 291 ($M^{+\bullet}$, 2), 200 (100), 122 (19).

^{1}H NMR (400 MHz, $CDCl_3$): δ 7.29 – 7.18 (m, 8H), 7.07 (d, *J* = 6.6 Hz, 2H), 6.96 – 6.88 (m, 1H), 6.29 – 6.21 (m, 1H), 6.21 – 6.16 (m, 1H), 6.09 (dt, *J* = 11.7, 2.3 Hz, 1H), 4.53 (dd, *J* = 8.1, 5.8 Hz, 1H), 4.19 (bs, 1 NH), 3.10 (dd, *J* = 14.0, 5.7 Hz, 1H), 2.97 (dd, *J* = 14.0, 8.2 Hz, 1H).

^{13}C NMR (101 MHz, $CDCl_3$): δ 163.9 (d, $J_{F,C}$ = 264 Hz), 149.0 (d, $J_{F,C}$= 11.5 Hz), 142.8, 137.3, 130.0 (d, $J_{F,C}$= 10.8 Hz), 129.1, 128.68, 128.61, 127.2, 126.8, 126.3, 109.4 (d, $J_{F,C}$= 2.3 Hz), 104.0 (d, J =21.9 Hz), 100.5 (d, $J_{F,C}$= 25 Hz), 59.1, 45.0.
^{19}F NMR (377 MHz, $CDCl_3$): δ -112.94.

3-Trifluoro-*N*-(1,2-diphenylethyl) (24):
Following the GPA with 3-fluoroaniline (38 µL, 0.30 mmol) in 16 h. The product was obtained as a white oil (74 mg, 0.22 mmol, 72%) after FC using a gradient from 0% to 20% of EtOAc in *n*-hexane as the eluent.

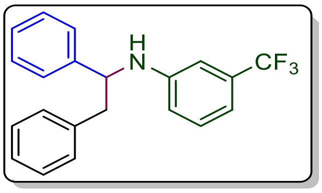

TLC: Rf = 0.42 (9:1 hexane/EtOAc, UV).
GC (Ti= 80 ºC): 7.072 min
MS: *m/z* (%): 341 ($M^{+\bullet}$, 4), 252 (19), 250 (100).
HRMS (ESI-QTOF): *m/z* calcd for $[M+H]^+$ for $C_{21}H_{19}F_3N^+$ 342.1464 observed 342.1467.
^{1}H NMR (400 MHz, $CDCl_3$): δ 7.30 – 7.16 (m, 8H), 7.10 – 7.02 (m, 3H), 6.80 (dd, J = 7.7 Hz, 1H), 6.65 (t, 1H), 6.51 (dd, J = 8.2, 2.1 Hz, 1H), 4.57 (t, J = 7.8 Hz 1H), 4.27 (bs, 1 NH), 3.12 (dd, J = 14.0, 5.7 Hz, 1H), 2.99 (dd, J = 14.0, 8.2 Hz, 1H).
^{13}C NMR (101 MHz, $CDCl_3$): δ 147.3, 142.5, 137.2, 129.4, 129.1, 131.3 (q, $J_{F,C}$= 36.6 Hz), 128.7, 128.6, 127.3, 126.9, 126.3, 122.8 (d, $J_{F,C}$), 116.2, 113.9 (q, $J_{F,C}$= 3.9 Hz), 110.1 (q, $J_{F,C}$= 3.9 Hz), 59.1, 44.9.
^{19}F NMR (377 MHz, $CDCl_3$): δ -62.95.

2-Chloro,4-methyl-*N*-(1,2-diphenylethyl) (25):
Following the GPA with 2-Chloro-4-methylaniline (37 µL, 0.30 mmol) in 16 h. The product was obtained as a colorless oil (76 mg, 0.24 mmol, 79%) after FC using a gradient from 0% to 5% of EtOAc in *n*-hexane as the eluent.

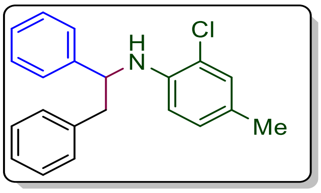

TLC: Rf = 0.85 (9:1 hexane/EtOAc, UV).
GC (Ti= 80 ºC): 8.032 min
MS: *m/z* (%): 321 ($M^{+\bullet}$, 4), 232 (53), 230 (100), 152 (17).
HRMS (ESI-QTOF): *m/z* calcd for $[M+H]^+$ for $C_{21}H_{21}ClN^+$ 322.1357 observed 322.1319.
^{1}H NMR (400 MHz, $CDCl_3$): δ 7.29 – 7.23 (m, 8H), 7.15 – 7.09 (m, 2H), 7.02 (dd, J = 2.1, 0.8 Hz, 1H), 6.71 (ddd, J = 8.3, 2.1, 0.8 Hz, 1H), 6.26 (d, J = 8.3 Hz, 1H), 4.71 (d, J = 4.8 Hz, 1H), 4.56 (dt, J = 8.0, 5.2 Hz, 1H), 3.16 (dd, J = 13.8, 5.6 Hz, 1H), 3.06 (dd, J = 13.8, 8.0 Hz, 1H), 2.13 (s, 3H).
^{13}C NMR (101 MHz, $CDCl_3$): δ 142.9, 140.7, 137.4, 129.3, 129.3, 128.5, 128.5, 128.0, 127.1, 126.8, 126.7, 126.3, 119.2, 112.7, 59.4, 45.2, 20.0.

N-Methylaniline-N-(1,2-diphenylethyl) (26):

Following the GPA with *N*-methylaniline (33 µL, 0.30 mmol) in 16 h. The product was obtained as red oil (77 mg, 0.26 mmol, 89%) after FC using a gradient from 0% to 5% of EtOAc in *n*-hexane as the eluent. The spectroscopy data matched previously reported literature values.[25]

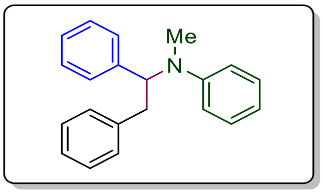

TLC: Rf = 0.85 (9:1 hexane/EtOAc, UV).

GC (Ti= 80 ºC): 7.487 min

MS: *m/z* (%): 287 ($M^{+\bullet}$, 3), 196 (100), 180 (15).

^{1}H NMR (400 MHz, $CDCl_3$): δ 7.30 – 7.09 (m, 13H), 6.65 (d, *J* = 6.7 Hz, 2H), 5.28 – 5.19 (m, 1H), 3.31 (qd, *J* = 14.3, 7.6 Hz, 2H), 2.71 (s, 3H).

^{13}C NMR (101 MHz, $CDCl_3$): δ 150.4, 141.1, 139.2, 129.0, 128.4, 128.3, 127.3, 127.1, 126.2, 116.9, 113.5, 63.6, 37.8, 32.5.

N-(1,2-diphenylethyl)-4-Methylaniline (27):

Following the GPA with *p*-toluidine (32.1 mg, 0.30 mmol) in 16 h. The product was obtained as white oil (71.5 mg, 0.25 mmol, 84%) after FC using a gradient from 0% to 5% of EtOAc in *n*-hexane as the eluent. The spectroscopy data matched previously reported literature values.[18]

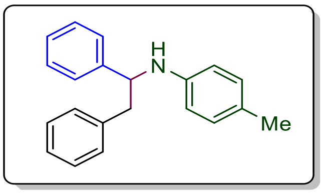

TLC: Rf = 0.64 (9:1 hexane/EtOAc, UV).

GC (Ti= 80 ºC): 7.653 min.

MS: *m/z* (%):287 ($M^{+\bullet}$, 8), 197 (48), 196 (100), 194 (13), 118 (15).

^{1}H NMR (400 MHz, $CDCl_3$): δ 7.37 – 7.07 (m, 8H), 7.06 – 6.97 (m, 2H), 6.81 – 6.68 (m, 2H), 6.28 (d, *J* = 8.4 Hz, 2H), 4.46 (dd, *J* = 8.3, 5.6 Hz, 1H), 3.90 (bs, 1 NH), 3.02 (dd, *J* = 14.0, 5.6 Hz, 1H), 2.89 (dd, *J* = 14.0, 8.3 Hz, 1H), 2.06 (s, 3H).

^{13}C NMR (101 MHz, $CDCl_3$): δ 145.1, 143.7, 137.9, 129.6, 128.7, 128.6, 127.1, 126.8, 126.7, 126.6, 113.8, 59.5, 45.3, 20.5.

4-Trifluoromethoxy-N-(1,2-diphenylethyl)aniline (28):

Following the GPA with 4-trifluoromethoxyaniline (41 µL, 0.30 mmol) in 16 h. The product was obtained as a brown oil (98 mg, 0.27 mmol, 91%) after FC using a gradient from 0% to 10% of EtOAc in *n*-hexane as the eluent.

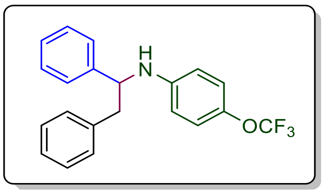

TLC: Rf = 0.71 (9:1 hexane/EtOAc, UV).

GC (Ti= 80 ºC): 7.181 min

MS: *m/z* (%):357 ($M^{+•}$, 2), 267 (35), 266 (100), 207 (36), 188 (25).

HRMS (ESI-QTOF): *m/z* calcd for $[M+H]^+$ for $C_{21}H_{19}F_3NO^+$ 358.1413 observed 358.1437.

^{1}H NMR (400 MHz, $CDCl_3$): δ 7.30 – 7.25 (m, 4H), 7.25 – 7.14 (m, 4H), 7.07 (d, J = 6.7 Hz, 2H), 6.84 (d, J = 8.3 Hz, 2H), 6.35 (d, J = 9.0 Hz, 2H), 4.49 (dd, J = 8.3, 5.7 Hz, 1H), 4.13 (bs, 1 NH), 3.09 (dd, J = 14.0, 5.6 Hz, 1H), 2.95 (dd, J = 14.0, 8.4 Hz, 1H).

^{13}C NMR (101 MHz, $CDCl_3$): δ 146.0, 142.9, 140.5, 137.4, 129.1, 128.7, 128.6, 127.3, 126.8, 126.3, 122.1, 119.3 (q, $J_{F\text{-}C}$= 256 Hz), 113.8, 59.4, 45.1.

^{19}F NMR (377 MHz, $CDCl_3$): δ -58.45.

Methyl 3-((1,2-diphenylethyl)amino)thiophene-2-carboxylate (29):

Following the GPA with methyl 3-amino-2-thiophenecarboxylate (47 mg, 0.30 mmol) and 2 equiv. TFA in 48 h. The product was obtained as a greenish oil (44 mg, 0.13 mmol, 43%) after FC using a gradient from 0% to 30% of EtOAc in *n*-hexane as the eluent.

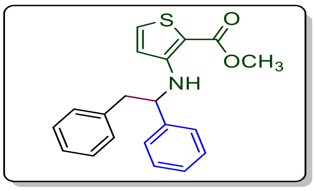

TLC: Rf = 0.60 (9:1 hexane/EtOAc, UV).

GC (Ti= 80 ºC): 8.307 min

MS: *m/z* (%):337 ($M^{+•}$, 4), 246 (89), 214 (100), 207 (56).

HRMS (EI-QTOF): *m/z* calcd for $C_{19}H_{16}NOS^+$ 306.0942 observed 306.0951.

^{1}H NMR (400 MHz, $CDCl_3$): δ 7.33 – 7.22 (m, 9H), 7.17 – 7.10 (m, 3H), 6.30 (d, J = 5.5 Hz, 1H), 4.67 (q, J = 6.7 Hz, 1H), 3.84 (s, 3H), 3.13 (d, J = 6.9 Hz, 2H).

^{13}C NMR (101 MHz, $CDCl_3$): δ 165.3, 142.9, 137.4, 131.8, 129.4, 128.6, 128.4, 127.3, 126.7, 126.4, 117.2, 61.1, 51.1, 45.2.

4-Methoxy-*N*-(1,2-diphenylethyl) (30):

Following the GPB for 16 h. The product was obtained as white oil (81 mg, 0.27 mmol, 89%) after FC using a gradient from 0% to 5% of EtOAc in *n*-hexane as the eluent. The spectroscopy data matched previously reported literature values.[18]

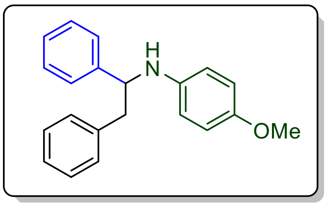

TLC: Rf = 0.48 (9:1 hexane/EtOAc, UV).

GC (Ti= 80 ºC): 8.125 min

MS: *m/z* (%): 303 ($M^{+•}$, 15), 212 (100).

^{1}**H NMR** (400 MHz, $CDCl_3$): δ 7.36 – 7.19 (m, 8H), 7.16 – 7.10 (m, 2H), 6.70 – 6.61 (m, 2H), 6.44 – 6.38 (m, 2H), 4.52 (dd, *J* = 8.3, 5.6 Hz, 1H), 3.86 (bs, 1 NH), 3.67 (s, 3H), 3.13 (dd, *J* = 13.9, 5.6 Hz, 1H), 3.00 (dd, *J* = 13.9, 8.3 Hz, 1H).

^{13}**C NMR** (101 MHz, $CDCl_3$): δ 152.1, 143.7, 141.5, 137.8, 129.2, 128.55, 128.52, 127.0, 126.6, 126.5, 114.9, 114.6, 60.0, 55.7, 45.2.

4-methoxy-*N*-(1-phenyl-2-(p-tolyl)ethyl)aniline (31):

Following the GPB with *p*-xylene (180 µL, 1.5 mmol) for 24 h. The product was obtained as white oil (71 mg, 0.22 mmol, 75%) after FC using a gradient from 0% to 5% of EtOAc in *n*-hexane as the eluent. The spectroscopy data matched previously reported literature values.[18]

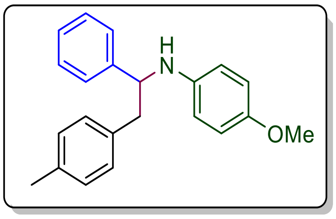

TLC: Rf = 0.75 (9:1 hexane/EtOAc, UV).

GC (Ti= 80 ºC): 8.40 min.

MS: *m/z* (%): 317 ($M^{+•}$, 7), 213 (28), 212 (100).

^{1}**H NMR** (400 MHz, $CDCl_3$): 7.35 – 7.28 (m, 4H), 7.23 – 7.21 (m, 1H), 7.10 – 7.06 (m, 2H), 7.04 – 7.00 (m, 2H), 6.65-6.63 (m, 2H), 6.43 – 6.39 (m, 2H), 4.48 (dd, *J* = 8.5, 5.3 Hz, 1H), 3.87 (bs, 1 NH), 3.67 (s, 3H), 3.09 (dd, *J* = 14.0, 5.5 Hz, 1H), 2.94 (dd, *J* = 14.0, 8.5 Hz, 1H), 2.32 (s, 3H).

^{13}**C NMR** (101 MHz, $CDCl_3$): δ 152.2, 144.0, 141.8, 136.3, 134.8, 129.3, 129.2, 128.6, 127.1, 126.6, 115.0, 114.8, 60.2, 55.8, 45.0, 21.2.

***N*-(2-(4-chlorophenyl)-1-phenylethyl)-4-methoxyaniline (32):**

Following the GPB with 1-chloro-4-methylbenzene (189 mg, 1.5 mmol) for 24 h. The product was obtained as white oil (43.5 mg, 0.13 mmol, 43%) after FC using a gradient from 0% to 5% of EtOAc in *n*-hexane as the eluent.

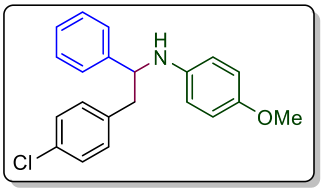

TLC: Rf = 0.32 (9:1 hexane/EtOAc, UV).

GC (Ti= 80 ºC): 8.836 min.

MS: *m/z* (%): 337 ($M^{+•}$, 2), 212 (100), 213 (18), 207 (5).

HRMS (ESI-TOF): *m/z* calcd for $[M+H]^+$ for $C_{21}H_{21}ClNO^+$ 338.1306, found 338.1309.

^{1}**H NMR** (400 MHz, $CDCl_3$): δ 7.43 – 7.01 (m, 7H), 6.93 (d, *J* = 8.4 Hz, 2H), 6.58 (d, *J* = 8.9 Hz, 2H), 6.35 (d, *J* = 9.0 Hz, 2H), 4.40 (dd, *J* = 7.6, 6.2 Hz, 1H), 3.59 (s, 3H), 3.13 – 2.78 (m, 2H).

^{13}**C NMR** (101 MHz, $CDCl_3$): δ 152.2, 143.1, 141.2, 136.3, 132.5, 130.6, 128.6, 128.6, 127.2, 126.5, 114.9, 114.7, 60.0, 55.7, 44.3.

***N*-2-methoxy-1,2-diphenylethylaniline (33):**

Following the GPB with (methoxymethyl)benzene (183 mg, 1.5 mmol) for 24 h. The product was obtained as white oil (76.3 mg, 0.25 mmol, 84%, 53:47 dr according to ^{1}H-NMR (50:50 dr before purification)) after FC using a gradient from 0% to 5% of EtOAc in *n*-hexane as the eluent.

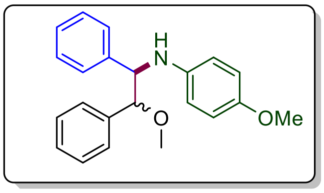

TLC: Rf = 0.28 (9:1 hexane/EtOAc, UV).

GC (Ti= 80 ºC): 8.208 min.

MS: *m/z* (%): 333 ($M^{+•}$, 2), 212 (100), 213 (22), 197 (5).

HRMS (ESI-TOF): *m/z* calcd for $[M+H]^+$ for $C_{22}H_{24}NO_2^+$ 334.1802, found 334.1814.

^{1}H NMR (400 MHz, $CDCl_3$): δ 7.14 (dt, *J* = 4.1, 2.7 Hz, 6.28H), 7.10 – 7.00 (m, 6H), 6.98 – 6.93 (m, 4H), 6.91 – 6.86 (m, 2H), 6.58 – 6.51 (m, 4H), 6.41 – 6.33 (m, 4H), 4.50 (d, *J* = 4.4 Hz, 1.18H), 4.44 (d, *J* = 4.4 Hz, 1.21H), 4.22 (d, *J* = 7.5 Hz, 0.93H), 4.13 (d, *J* = 7.5 Hz, 0.93H), 3.56 (d, *J* = 1.2 Hz, 5.68H), 3.20 (s, 3H), 3.16 (s, 2.63H).

^{13}C NMR (101 MHz, $CDCl_3$): δ 152.2, 152.1, 142.1, 141.2, 140.7, 139.1, 138.4, 137.9, 128.3, 128.1, 127.9, 127.8, 127.7, 127.5, 127.1, 127.0, 115.5, 115.2, 114.7, 114.6, 87.9, 86.8, 65.6, 63.7, 55.7, 55.7.

***N*-(1-(4-(tert-butyl)phenyl)-2-phenylethyl)-4-methoxyaniline (34):**

Following the GPB with *p-tert*-butylbenzaldehyde (60 µL, 0.36 mmol) in 72 h. The product was obtained as yellow oil (72 mg, 0.2 mmol, 69%) after FC using a gradient from 0% to 20% of EtOAc in *n*-hexane as the eluent.

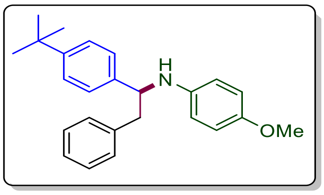

TLC: Rf = 0.44 (9:1 hexane/EtOAc, UV).

GC (Ti= 80 ºC): 9.108 min

MS: *m/z* (%): 359 ($M^{+•}$, 2), 269 (30), 268 (100), 253 (15), 238 (13), 134 (10)

HRMS (EI-QTOF): *m/z* calcd for $C_{25}H_{29}NO$ 359.2249 observed 359.2237.

^{1}H NMR (400 MHz, $CDCl_3$): 7.29 – 7.25 (m, 2H), 7.24 – 7.19 (m, 4H), 7.18 – 7.13 (m, 1H), 7.12 – 7.08 (m, 2H), 6.62 – 6.57 (m, 2H), 6.40 – 6.33 (m, 2H), 4.43 (dd, *J* = 8.7, 5.2 Hz, 1H), 3.78 (bs, 1 NH), 3.61 (s, 3H), 3.07 (dd, *J* = 14.1, 5.2 Hz, 1H), 2.90 (dd, *J* = 14.1, 8.7 Hz, 1H), 1.26 (s, 9H).

^{13}C NMR (101 MHz, $CDCl_3$): δ 152.4, 142.9, 141.2, 137.4, 131.7, 129.3, 128.7, 128.4, 126.9, 120.8, 115.0, 114.8, 59.7, 55.8, 45.2.

***N*-(1-(4-bromophenyl)-2-phenylethyl)-4-methoxyaniline (35):**

Following the GPB with *p*-bromobenzaldehyde (66.6 mg, 0.36 mmol) in 24 h. The product was obtained as white oil (88 mg, 0.23 mmol, 77%) after FC using a gradient from 0% to 20% of EtOAc in *n*-hexane as the eluent. The spectroscopy data matched previously reported literature values.[26]

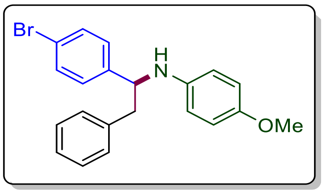

TLC: Rf = 0.33 (9:1 hexane/EtOAc, UV).

GC (Ti= 80 ºC): 9.215 min

MS: *m/z* (%): 383 ($M^{+•}$, 4), 382 (1), 381(M^{+}, 4), 292 (98), 293 (33), 292 (100), 211 (11), 196 (10), 134 (10).

^{1}H NMR (400 MHz, $CDCl_3$): 7.27 (dd, *J* = 8.5, 2.1 Hz, 2H), 7.16 – 7.07 (m, 3H), 7.04 (dd, *J* = 8.5, 2.0 Hz, 2H), 6.96 (d, *J* = 6.5 Hz, 2H), 6.55 – 6.48 (m, 2H), 6.27 – 6.21 (m, 2H), 4.33 (t, J= 6.98 Hz, 1H), 3.72 (bs, 1 NH), 3.52 (s, 3H), 2.92 (dd, *J* = 13.9, 5.9 Hz, 1H), 2.83 (dd, *J* = 13.9, 8.0 Hz, 1H).

^{13}C NMR (101 MHz, $CDCl_3$): δ 152.4, 142.9, 141.2, 137.4, 131.7, 129.3, 128.7, 128.4, 126.9, 120.8, 115.0, 114.8, 59.7, 55.8, 45.2.

4-Methoxy-*N*-(2-phenyl-1-(pyridin-4-yl)ethyl)aniline (36):

Following the GPB with pyridine-4-carbaldehyde (32 µL, 0.36 mmol) for 24 h. The product was obtained as a white oil (28 mg, 0.09 mmol, 31%) after FC using a gradient from 0% to 70% EtOAc in *n*-hexane as the eluent.

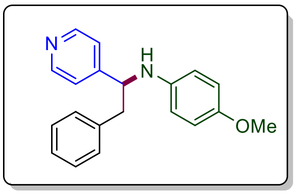

TLC: Rf = 0.30 (6:4 hexane/EtOAc, UV).

GC (Ti= 80 ºC): 8.551 min.

MS: *m/z* (%): 304 ($M^{+•}$, 4), 281 (30), 213 (100).

HRMS (EI-QTOF): *m/z* calcd for $C_{20}H_{20}N_2O$ 304.1576 observed 304.1579.

^{1}H NMR (400 MHz, $CDCl_3$): δ 8.51 (s, 2H), 7.32 – 7.20 (m, 7H), 7.13 – 7.07 (m, 2H), 6.65 (d, *J* = 9.0 Hz, 2H), 6.36 (d, *J* = 9.0 Hz, 2H), 4.49 (dd, *J* = 8.0, 5.9 Hz, 1H), 3.67 (s, 3H), 3.09 (dd, *J* = 13.9, 5.8 Hz, 1H), 3.00 (dd, *J* = 13.9, 8.1 Hz, 1H).

^{13}C NMR (101 MHz, $CDCl_3$): δ 152.93, 152.45, 150.01, 140.75, 136.72, 129.19, 128.73, 127.09, 121.85, 114.91, 114.73, 59.25, 55.68, 44.47.

***N*-(1-cyclohexyl-2-phenylethyl)-4-methoxyaniline (37):**

Following GPB with cyclohexanecarbaldehyde (37 mg, 0.33 mmol) in 24 h. The product was obtained as white oil (74.1 mg, 0.24 mmol, 80%) after FC using a gradient from 0% to 5% of EtOAc in *n*-hexane as the eluent.

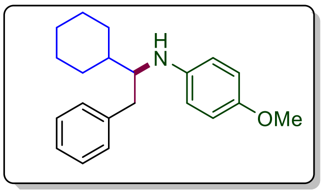

TLC: Rf = 0.45 (9:1 hexane/EtOAc, UV).

GC (Ti= 80 ºC): 8.130 min.

MS: *m/z* (%): 309 ($M^{+•}$,, 10), 218 (100), 219 (19), 136 (17).

HRMS (ESI-TOF): *m/z* calcd for $[M+H]^{+}$ for $C_{21}H_{27}NO^{+}$ 310.2165, found 310.2172.

^{1}H NMR (400 MHz, $CDCl_3$): δ 7.22 – 7.15 (m, 2H), 7.13 – 7.06 (m, 3H), 6.69 – 6.62 (m, 2H), 6.44 – 6.39 (m, 2H), 3.65 (s, 3H), 3.30 (td, *J* = 6.4, 4.7 Hz, 1H), 2.76 (dd, *J* = 13.9, 6.0 Hz, 1H), 2.66 (dd, *J* =

13.9, 6.8 Hz, 1H), 1.83 – 1.74 (m, 1H), 1.73 – 1.63 (m, 3H), 1.61 – 1.54 (m, 1H), 1.39 (ddd, *J* = 11.1, 6.9, 3.4 Hz, 1H), 1.20 – 0.95 (m, 5H).

^{13}C NMR (101 MHz, $CDCl_3$): δ 151.6, 142.5, 139.6, 129.3, 128.3, 126.0, 114.9, 114.4, 60.2, 55.8, 40.4, 37.4, 29.9, 28.4, 26.6, 26.4, 26.4.

POST FUNCTIONALIZATION

1,2-diphenylethan-1-amine (38) :

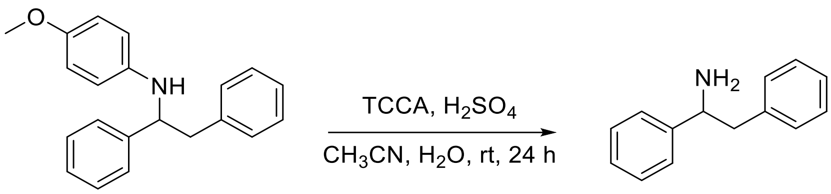

Using a reported protocol,[27] 4-Methoxy-*N*-(1,2-diphenylethyl) (152 mg, 0.50 mmol) was added to a 50 mL round-bottom flask with trichloroisocyanuric acid (TCCA, 58 mg, 0.25 mmol) and H_2SO_4 (1M, 0.5 mL, 0.50 mmol) in CH_3CN (5 mL) and H_2O (5 mL). The reaction mixture was stirred for 24 h at room temperature. At this point, another 2 mL of H_2O was added, and the reaction mixture was washed with DCM (3 x 20 mL). The organic phase was discarded, and KOH (110 mg) was added to the aqueous phase, which was then extracted with EtOAc (3 x 15 mL). The combined organic layers were dried over $MgSO_4$ and concentrated under vacuum. The product was obtained pure, without further purification, as a brown oil (72 mg, 0.36 mmol, 73%). The spectroscopy data matched previously reported literature values.[28]

TLC: Rf = 0.2 (EtOAc, UV).

GC (Ti= 80 ºC): 6.500 min

MS: *m/z* (%): 106 ($M^{+\bullet}$, 100).

^{1}H NMR (400 MHz, $CDCl_3$): δ 7.29 – 7.11 (m, 8H), 7.11 – 7.06 (m, 2H), 4.10 (dd, *J* = 8.9, 5.0 Hz, 1H), 2.93 (dd, *J* = 13.3, 5.0 Hz, 1H), 2.74 (dd, *J* = 13.3, 8.9 Hz, 1H), 1.51 (s, 2H).

^{13}C NMR (101 MHz, $CDCl_3$): δ 145.7, 139.1, 129.4, 128.47, 128.46, 127.1, 126.49, 126.43, 57.6, 46.5.

***N*,*N*-dimethyl-1,2-diphenylethan-1-amine (Lefetamine) (39) :**

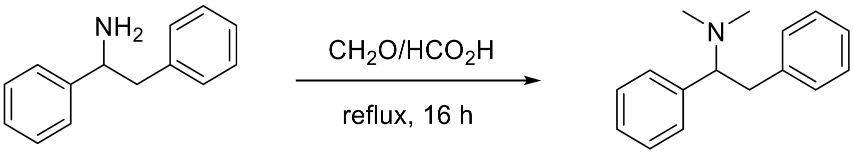

Using a reported protocol,[28] 1,2-diphenylethan-1-amine (39 µL, 0.2 mmol) was added to a Pyrex tube with formaldehyde 37% (113 µL, 1.2 mmol) and formic acid 97% (87 µL, 2 mmol). The reaction mixture was stirred at reflux for 16 h. At this point, NaOH (1 M, aq.) was added until the pH reached 10, and the reaction mixture was extracted with DCM (3 × 5 mL). The combined organic layers were dried over $MgSO_4$ and concentrated under vacuum. The product was obtained as a white oil (36 mg, 0.16 mmol, 80%) after FC using hexane:EtOAc:Et_3N (100:20:5) as the eluent. The spectroscopy data matched previously reported literature values. [28]

TLC: Rf = 0.25 (3:7 hexane: EtOAc, UV).

GC (Ti= 80 ºC): 6.541 min

MS: *m/z* (%): 225 ($M^{+\bullet}$, 3), 207 (11), 134 (100).

^{1}H NMR (400 MHz, $CDCl_3$): δ 7.19 – 7.09 (m, 3H), 7.09 – 6.94 (m, 5H), 6.87 (d, *J* = 6.7 Hz, 2H), 3.38 (dd, *J* = 9.7, 5.0 Hz, 1H), 3.23 (dd, *J* = 13.3, 5.0 Hz, 1H), 2.88 (dd, *J* = 13.3, 9.7 Hz, 1H), 2.18 (s, 6H).

^{13}C NMR (101 MHz, $CDCl_3$): δ 139.6, 139.5, 129.3, 128.8, 127.9, 127.8, 127.1, 125.8, 72.7, 42.9, 40.0.

1-(1,2-diphenylethyl)piperidine (Diphenidine) (40) :

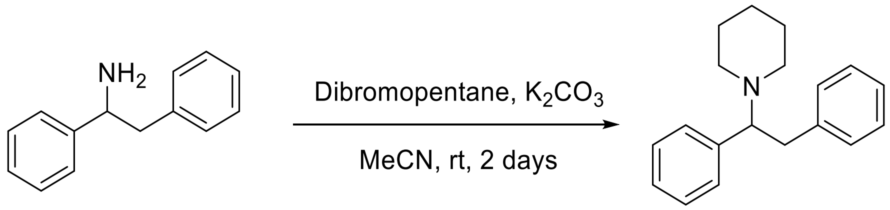

Using a reported protocol, [28] 1,2-diphenylethan-1-amine (39 µL, 0.2 mmol) was added to a Pyrex tube with K_2CO_3 (165 mg, 1.2 mmol) in anhydrous acetonitrile (0.5 mL). Then, 1,5-dibromopentane (82 µL, 0.3 mmol) was added dropwise to the mixture. The reaction mixture was stirred at room temperature for 2 days. Once the reaction was complete, the salts were removed by filtration, and the resulting solution was concentrated under vacuum and then purified by FC. The product was obtained as a white oil (46 mg, 0.17 mmol, 87%) after FC using Hexane:EtOAc:Et_3N (100:20:5) as the eluent. The spectroscopy data matched previously reported literature values.[28]

TLC: Rf = 0,25 (3:7 hexane : EtOAc, UV).

GC (Ti= 80 ºC): 8.022 min

MS: *m/z* (%):265 ($M^{+\bullet}$, 2), 174 (100).

^{1}H NMR (400 MHz, $CDCl_3$): δ 7.40 – 7.30 (m, 3H), 7.30 – 7.16 (m, 5H), 7.13 (d, *J* = 7.3 Hz, 2H), 3.72 (dd, *J* = 9.4, 5.2 Hz, 1H), 3.44 (dd, *J* = 13.3, 5.2 Hz, 1H), 3.13 (dd, *J* = 13.3, 9.4 Hz, 1H), 2.72 – 2.35 (m, 4H), 1.79 – 1.59 (m, 4H), 1.58 – 1.43 (m, 2H).

^{13}C NMR (101 MHz, $CDCl_3$): δ 140.03, 139.4, 129.4, 128.9, 127.8, 127.7, 126.8, 125.6, 72.4, 51.4, 39.2, 26.4, 24.7.

***N*-(1,2-diphenylethyl)-2-(4-isobutylphenyl)propanamide (41) :**

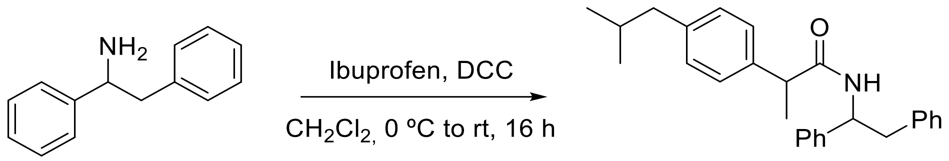

Using a reported protocol,[29] 2-(4-isobutylphenyl)propionic acid (ibuprofen, 206 mg, 1 mmol) was added to a 10 mL round-bottom flask containing *N,N'*-dicyclohexylcarbodiimide (DCC, 103 mg, 0.5 mmol) in dichloromethane (DCM, 0.83 mL) at room temperature for 1 h. The 2-(4-isobutylphenyl)propanoic peroxyanhydride formed was filtered and washed with DCM. The liquid was reduced under vacuum.
In the second part, this anhydride was dissolved in DCM (1 mL) and added to a Pyrex tube with 1,2-diphenylethan-1-amine (39 µL, 0.2 mmol) in DCM (1 mL) at 0 ºC. The resulting mixture was allowed to warm to room temperature and stirred for 16 h. After this time, the solvent was removed under vacuum, and the residue was purified. The product was obtained as a white solid (52 mg, 0.13 mmol, 67%) after FC using a gradient from 0% to 20% of EtOAc in *n*-hexane as the eluent.

TLC: Rf = 0.25 (3:7 hexane: EtOAc, UV).

GC (Ti= 80 ºC): 9.490 min

MS: *m/z* (%):385 ($M^{+\bullet}$, 6), 294 (97), 186 (100).

HRMS (ESI-QTOF): *m/z* calcd for $[M+H]^+$ for $C_{27}H_{32}NO^+$ 386.2478, found 386.2495.

^{1}H NMR (400 MHz, $CDCl_3$): δ 7.46 – 7.31 (m, 6H), 7.24 (dd, *J* = 9.8, 3.5 Hz, 5H), 7.14 (d, *J* = 7.1 Hz, 1H), 7.05 (d, *J* = 5.4 Hz, 1H), 6.99 (dd, *J* = 7.3, 1.9 Hz, 1H), 5.84 – 5.69 (m, 1H), 5.48 – 5.33 (m, 1H), 3.71 – 3.56 (m, 1H), 3.26 – 3.13 (m, 1H), 3.12 – 2.95 (m, 1H), 2.66 (dd, *J* = 7.1, 4.3 Hz, 2H), 2.05 (dq, *J* = 13.5, 6.8 Hz, 1H), 1.61 (dd, *J* = 11.3, 7.2 Hz, 3H), 1.16 – 1.02 (m, 6H).

^{13}C NMR (101 MHz, $CDCl_3$): δ 173.6 (d, *J* = 6.6 Hz), 141.7 (d, *J* = 4.4 Hz), 140.7 (d, *J* = 6.0 Hz), 138.6 – 137.8 (m), 136.9, 129.7, 129.6, 129.3, 129.2, 128.4, 128.38, 128.30, 128.2, 127.4, 127.2, 127.1, 126.54, 126.50, 126.3, 126.1, 54.0, 46.77, 46.74, 45.08, 45.05, 42.8, 42.5, 30.2, 22.3, 18.07, 18.00.

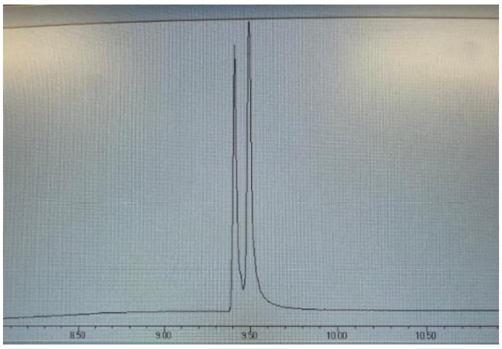

Figure S34: GC-MS trace shows a 1:1 d.r. for product **41**.

DIFFERENT ACCEPTORS OF BENZYL RADICALS

Diethyl 2-(1-phenylpropan-2-yl)malonate (42)

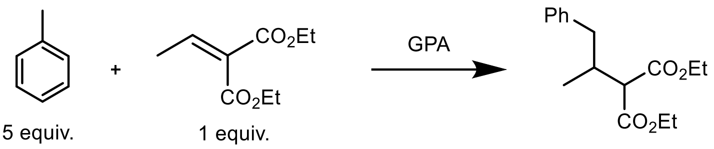

In a two-dram vial equipped with a magnetic stirring bar, diethyl 2-ethylidenemalonate (0.30 mmol, 56 µL), and toluene (5 equiv, 1.50 mmol, 158 µL) were added, followed by 9-(2-chlorophenyl)acridine (**A1**, 2.5 mmol%, 2.2 mg, 0.0075 mmol). A mixture of 1,2-dichloroethane (DCE, 2.1 mL) and 1,1,1,3,3,3-hexafluoro-2-propanol (HFIP, 0.9 mL) was then added to the vial, followed by trifluoroacetic acid (TFA, 1.1 equiv, 0.33 mmol, 26 µL). The vial was sealed and placed in the PhotoRedOx Box Duo photoreactor. The reaction mixture was irradiated with blue LEDs (λ = 450 nm) for 16 hours at room temperature (approximately 25–30 °C, controlled by a fan). After completion, the reaction mixture was concentrated under reduced pressure, and the resulting residue was dissolved in ethyl acetate (EtOAc). The mixture was then quenched by adding K_2CO_3 (approximately 40 mg), stirred for 30 minutes, and filtered. The crude mixture was concentrated under reduced pressure. The resulting residue was purified by FC using a gradient from 0% to 40% EtOAc in n-hexane as the eluent, affording the desired product as a white oil (42 mg, 0.15 mmol, 50%). The spectroscopic data matched those reported in the literature.[30]

TLC: Rf = 0.30 (9:1 hexane: EtOAc, UV).

GC (Ti= 80 ºC): 7.020 min

MS: *m/z* (%):278 ($M^{+\bullet}$, 3), 187 (23), 160 (65), 118 (100).

^{1}H NMR (400 MHz, $CDCl_3$): δ 7.31 – 7.27 (m, 2H), 7.20 – 7.17 (m, 3H), 4.26 – 4.15 (m, 4H), 3.29 (d, *J* = 7.4 Hz, 1H), 2.84 (dd, *J* = 13.1, 5.0 Hz, 1H), 2.61 – 2.49 (m, 1H), 2.43 (dd, *J* = 13.1, 9.1 Hz, 1H), 1.28 (td, *J* = 7.0, 2.9 Hz, 6H), 0.96 (d, *J* = 6.7 Hz, 3H).

^{13}C NMR (101 MHz, $CDCl_3$): δ 168.9, 168.7, 139.9, 129.3, 128.3, 126.2, 61.27, 61.21, 56.9, 40.6, 35.4, 16.7, 14.1.

Ethyl 2-methylene-4-phenylbutanoate (43)

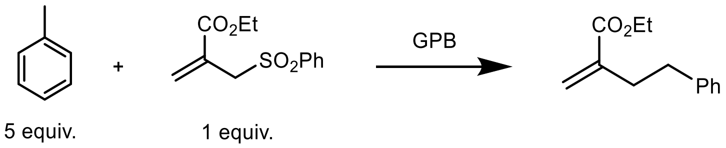

In a two-dram vial equipped with a magnetic stirring bar, ethyl 2-((phenylsulfonyl)methyl)acrylate (0.30 mmol, 76 µL) and toluene (5 equiv, 1.50 mmol, 158 µL) were added in acetonitrile (3 mL). Then, **A1** (2.5 mmol%, 2.2 mg, 0.0075 mmol) and TsOH (1.1 equiv., 57 mg, 0.33 mmol) were added, and a precipitate formed. The reaction mixture was irradiated with blue LEDs (λ = 450 nm) for 16 hours at room

temperature (approximately 25–30 °C, controlled by a fan). Once finished, EtOAc (10 mL) was added to the reaction mixture, which was then washed with sat. aq. K_2CO_3 (3 x 5 mL). The combined organic phases were concentrated under reduced pressure, and the resulting residue was purified by FC using a gradient from 0% to 10% EtOAc in *n*-hexane as the eluent, affording a green oil (19 mg, 0.09 mmol, 32%). The spectroscopic data matched those reported in the literature. [31]

TLC: Rf = 0.40 (9:1 hexane: EtOAc, UV).

GC (Ti= 80 ºC): 8.489 min

MS: *m/z* (%):204 ($M^{+\bullet}$, 37), 104 (37), 101 (100).

^{1}H NMR (400 MHz, $CDCl_3$): δ 7.29 – 7.26 (m, 3H), 7.21 – 7.18 (m, 2H), 6.15 (dd, *J* = 1.4, 0.6 Hz, 1H), 5.50 (q, *J* = 1.4 Hz, 1H), 4.22 (q, *J* = 7.1 Hz, 2H), 2.80 (dd, *J* = 9.4, 6.3 Hz, 2H), 2.62 (dddd, *J* = 9.5, 6.6, 1.4, 0.7 Hz, 2H), 1.31 (t, *J* = 7.1 Hz, 3H).

^{13}C NMR (101 MHz, $CDCl_3$): δ 167.1, 141.4, 140.1, 128.4, 128.3, 125.9, 125.1, 60.6, 34.9, 33.9, 14.2.

1-Benzylisoquinoline (44)

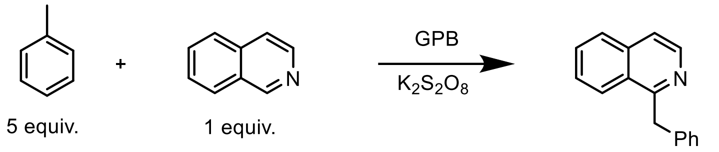

In a two-dram vial equipped with a magnetic stirring bar, isoquinoline (0.3 mmol, 35 µL) and toluene (5 equiv, 1.50 mmol, 158 µL) were added in acetonitrile (3 mL). Then, **A1** (2.5 mmol%, 2.2 mg, 0.0075 mmol), TsOH (1.1 equiv., 57 mg, 0.33 mmol) and K2S2O8 (2 equiv., 0.6 mmol, 162 mg) were added, and a precipitate formed. The reaction mixture was irradiated with blue LEDs (λ = 450 nm) for 16 hours at room temperature (approximately 25–30 °C, controlled by a fan). Once finished, EtOAc (10 mL) was added to the reaction mixture, which was then washed with sat. aq. K_2CO_3 (3 x 5 mL). The combined organic phases were concentrated under reduced pressure, and the resulting residue was purified by FC using a gradient from 0% to 10% EtOAc in *n*-hexane as the eluent, affording a green oil (25 mg, 0.114 mmol, 38%). The spectroscopic data matched those reported in the literature.[32]

TLC: Rf = 0.20 (9:1 hexane: EtOAc, UV).

GC (Ti= 80 ºC): 6.294 min

MS: *m/z* (%):219 ($M^{+\bullet}$, 30), 218 (100), 217 (37). 216 (11).

^{1}H NMR (400 MHz, $CDCl_3$): δ 8.50 (d, *J* = 5.7 Hz, 1H), 8.15 (dd, *J* = 8.5, 1.0 Hz, 1H), 7.81 (d, *J* = 1.1 Hz, 1H), 7.64 (ddd, *J* = 8.2, 6.9, 1.2 Hz, 1H), 7.56 (d, *J* = 0.9 Hz, 1H), 7.53 (ddd, *J* = 8.3, 6.9, 1.3 Hz, 1H), 7.30 – 7.26 (m, 3H), 7.26 – 7.22 (m, 1H), 7.17 (ddt, *J* = 6.7, 5.9, 1.9 Hz, 1H), 4.68 (s, 2H).

^{13}C NMR (101 MHz, $CDCl_3$): δ 160.2, 142.0, 139.5, 136.6, 129.9, 128.6, 128.5, 127.4, 127.2, 126.3, 125.8, 119.8, 42.1.

REFERENCES

(1) Laze, L.; Quevedo-Flores, B.; Bosque, I.; Gonzalez-Gomez, J. C. Alkanes in Minisci-Type Reaction under Photocatalytic Conditions with Hydrogen Evolution. *Org. Lett.* **2023**, *25* (48), 8541–8546. https://doi.org/10.1021/acs.orglett.3c02619.

(2) Ramirez, N. P.; Lana-Villarreal, T.; Gonzalez-Gomez, J. C. Direct Decarboxylative Allylation and Arylation of Aliphatic Carboxylic Acids Using Flavin-Mediated Photoredox Catalysis. *Eur J Org Chem* **2020**, *2020* (10), 1539–1550. https://doi.org/10.1002/ejoc.201900888.

(3) Pitzer, L.; Schäfers, F.; Glorius, F. Rapid Assessment of the Reaction-Condition-Based Sensitivity of Chemical Transformations. *Angew Chem Int Ed* **2019**, *58* (25), 8572–8576. https://doi.org/10.1002/anie.201901935.

(4) Zhao, Y.; Truhlar, D. G. The M06 Suite of Density Functionals for Main Group Thermochemistry, Thermochemical Kinetics, Noncovalent Interactions, Excited States, and Transition Elements: Two New Functionals and Systematic Testing of Four M06-Class Functionals and 12 Other Functionals. *Theor Chem Account* **2008**, *120* (1–3), 215–241. https://doi.org/10.1007/s00214-007-0310-x.

(5) Frisch, M. J. et al. Gaussian 16, Revision C.01, 2016.

(6) Weigend, F.; Ahlrichs, R. Balanced Basis Sets of Split Valence, Triple Zeta Valence and Quadruple Zeta Valence Quality for H to Rn: Design and Assessment of Accuracy. *Phys. Chem. Chem. Phys.* **2005**, *7* (18), 3297–3305. https://doi.org/10.1039/B508541A.

(7) Rappoport, D.; Furche, F. Property-Optimized Gaussian Basis Sets for Molecular Response Calculations. *The Journal of Chemical Physics* **2010**, *133* (13), 134105. https://doi.org/10.1063/1.3484283.

(8) Marenich, A. V.; Cramer, C. J.; Truhlar, D. G. Universal Solvation Model Based on Solute Electron Density and on a Continuum Model of the Solvent Defined by the Bulk Dielectric Constant and Atomic Surface Tensions. *J. Phys. Chem. B* **2009**, *113* (18), 6378–6396. https://doi.org/10.1021/jp810292n.

(9) Grimme, S. Supramolecular Binding Thermodynamics by Dispersion-Corrected Density Functional Theory. *Chemistry – A European Journal* **2012**, *18* (32), 9955–9964. https://doi.org/10.1002/chem.201200497.

(10) Luchini, G.; Alegre-Requena, J. V.; Funes-Ardoiz, I.; Paton, R. S. GoodVibes: Automated Thermochemistry for Heterogeneous Computational Chemistry Data. *F1000Res* **2020**, *9*, 291. https://doi.org/10.12688/f1000research.22758.1.

(11) Marcus, R. A. On the Theory of Oxidation-Reduction Reactions Involving Electron Transfer. I. *The Journal of Chemical Physics* **1956**, *24* (5), 966–978. https://doi.org/10.1063/1.1742723.

(12) Marcus, R. A. Electron Transfer Reactions in Chemistry: Theory and Experiment (Nobel Lecture). *Angewandte Chemie International Edition in English* **1993**, *32* (8), 1111–1121. https://doi.org/10.1002/anie.199311113.

(13) de Aguirre, A.; Funes-Ardoiz, I.; Maseras, F. Four Oxidation States in a Single Photoredox Nickel-Based Catalytic Cycle: A Computational Study. *Angewandte Chemie International Edition* **2019**, *58* (12), 3898–3902. https://doi.org/10.1002/anie.201814233.

(14) Pliego Jr, J. R.; Riveros, J. M. Hybrid Discrete-Continuum Solvation Methods. *WIREs Computational Molecular Science* **2020**, *10* (2), e1440. https://doi.org/10.1002/wcms.1440.

(15) Ortuño, M. A.; Lledós, A. How Acid Can Become a Dihydrogen Complex in Water? A DFT Study. *Journal of Organometallic Chemistry* **2021**, *949*, 121957. https://doi.org/10.1016/j.jorganchem.2021.121957.

(16) Weigend, F.; Furche, F.; Ahlrichs, R. Gaussian Basis Sets of Quadruple Zeta Valence Quality for Atoms H–Kr. *The Journal of Chemical Physics* **2003**, *119* (24), 12753–12762. https://doi.org/10.1063/1.1627293.

(17) Álvarez-Moreno, M.; de Graaf, C.; López, N.; Maseras, F.; Poblet, J. M.; Bo, C. Managing the Computational Chemistry Big Data Problem: The ioChem-BD Platform. *J. Chem. Inf. Model.* **2015**, *55* (1), 95–103. https://doi.org/10.1021/ci500593j.

(18) Weindl, C.; Helmbrecht, S. L.; Hintermann, L. Rapid C–H Transformation: Addition of Diarylmethanes to Imines in Seconds by Catalytic Use of Base. *J. Org. Chem.* **2023**, *88* (7), 4155–4161. https://doi.org/10.1021/acs.joc.2c02658.

(19) Shi, A.; Xie, P.; Wang, Y.; Qiu, Y. Photoelectrocatalytic Cl-Mediated C(Sp3)–H Aminomethylation of Hydrocarbons by BiVO4 Photoanodes. *Nature Communications* **2025**, *16* (1), 2322. https://doi.org/10.1038/s41467-025-57567-2.

(20) Dörfler, J.; Doye, S. Aminopyridinato Titanium Catalysts for the Hydroaminoalkylation of Alkenes and Styrenes. *Angew Chem Int Ed* **2013**, *52* (6), 1806–1809. https://doi.org/10.1002/anie.201206027.

(21) Wang, Y.-Z.; Liu, S.-D.; Cheng, L.; Liu, L.; Li, C.-J. Asymmetric Addition of Hydrazones as Alkyl Carbanion Equivalents with Aryl Imines in Water. *Org. Chem. Front.* **2023**, *10* (12), 3021–3026. https://doi.org/10.1039/D3QO00475A.

(22) Zhang, H.-H.; Yu, S. Radical Alkylation of Imines with 4-Alkyl-1,4-Dihydropyridines Enabled by Photoredox/Brønsted Acid Cocatalysis. *J. Org. Chem.* **2017**, *82* (19), 9995–10006. https://doi.org/10.1021/acs.joc.7b01425.

(23) Fan, R.; Pu, D.; Qin, L.; Wen, F.; Yao, G.; Wu, J. Efficient Three-Component One-Pot Benzylation and Allylation of Aldehydes and Amines for Synthesis of Homobenzylamines and Homoallylamines. *J. Org. Chem.* **2007**, *72* (8), 3149–3151. https://doi.org/10.1021/jo062616y.

(24) Hikawa, H.; Izumi, K.; Ino, Y.; Kikkawa, S.; Yokoyama, Y.; Azumaya, I. Palladium-Catalyzed Benzylic C-H Benzylation via Bis-Benzylpalladium(II) Complexes in Water: An Effective Pathway

for the Direct Construction of N-(1,2-Diphenylethyl)Anilines. *Adv Synth Catal* **2015**, *357* (5), 1037–1048. https://doi.org/10.1002/adsc.201401017.

(25) Xie, L.-G.; Dixon, D. J. Tertiary Amine Synthesis via Reductive Coupling of Amides with Grignard Reagents. *Chem. Sci.* **2017**, *8* (11), 7492–7497. https://doi.org/10.1039/C7SC03613B.

(26) Li, G.; Chen, R.; Wu, L.; Fu, Q.; Zhang, X.; Tang, Z. Alkyl Transfer from C=C Cleavage. *Angew Chem Int Ed* **2013**, *52* (32), 8432–8436. https://doi.org/10.1002/anie.201303696.

(27) Rubanov, Z. M.; Levin, V. V.; Dilman, A. D. Dual Acridine/Decatungstate Photocatalysis for the Decarboxylative Radical Addition of Carboxylic Acids to Azomethines. *Org. Lett.* **2024**, *26* (15), 3174–3178. https://doi.org/10.1021/acs.orglett.4c00778.

(28) Wang, Z.; Zheng, Z.; Xu, X.; Mao, J.; Walsh, P. J. One-Pot Aminobenzylation of Aldehydes with Toluenes. *Nat Commun* **2018**, *9* (1), 3365. https://doi.org/10.1038/s41467-018-05638-y.

(29) Alberca, S.; Velázquez, M.; Trujillo-Sierra, J.; Iglesias-Sigüenza, J.; Fernández, R.; Lassaletta, J. M.; Monge, D. Pd(II)-Catalyzed Asymmetric Addition of Arylboronic Acids to Aliphatic *N* -Carbamoyl Hydrazones. *Adv Synth Catal* **2022**, *364* (14), 2373–2379. https://doi.org/10.1002/adsc.202200430.

(30) Patehebieke, Y.; Charaf, R.; Bryce-Rogers, H. P.; Ye, K.; Ahlquist, M.; Hammarström, L.; Wallentin, C.-J. β-Scission of Secondary Alcohols via Photosensitization: Synthetic Utilization and Mechanistic Insights. *ACS Catal.* **2024**, *14* (1), 585–593. https://doi.org/10.1021/acscatal.3c05150.

(31) Zhang, J.; Li, Y.; Xu, R.; Chen, Y. Donor–Acceptor Complex Enables Alkoxyl Radical Generation for Metal-Free C(Sp^3)–C(Sp^3) Cleavage and Allylation/Alkenylation. *Angew Chem Int Ed* **2017**, *56* (41), 12619–12623. https://doi.org/10.1002/anie.201707171.

(32) Ballav, T.; Barman, S.; Ganesh, V. Aryne Alder-Ene Reaction Enables Arylation of Conformationally Locked Styrenes. *Org. Lett.* **2025**, *27* (16), 4107–4111. https://doi.org/10.1021/acs.orglett.5c00511.
